# Pesticides Curbing Soil Fertility: Effect of Complexation of Free Metal Ions

**DOI:** 10.3389/fchem.2017.00043

**Published:** 2017-07-04

**Authors:** Sukhmanpreet Kaur, Vijay Kumar, Mohit Chawla, Luigi Cavallo, Albert Poater, Niraj Upadhyay

**Affiliations:** ^1^Department of Chemistry, Lovely Professional UniversityJalandhar, India; ^2^Regional Ayurveda Research Institute for Drug DevelopmentGwalior, India; ^3^Kaust Catalysis Center, Physical Sciences and Engineering, King Abdullah University of Science and TechnologyThuwal, Saudi Arabia; ^4^Departament de Química, Facultat de Ciències, Institut de Química Computacional i Catàlisi, Universitat de GironaGirona, Spain; ^5^Department of Chemistry, Dr. Harisingh Gour VishwavidyalayaSagar, India

**Keywords:** organophosphate, carbamate, pesticide, complex, soil, complexation, DFT calculations

## Abstract

Researchers have suggested that the reason behind infertility is pernicious effect of broad spectrum pesticides on non target, beneficial microorganism of soil. Here, studying the chelating effect of selective organophosphate and carbamate pesticides with essential metal ions, at all possible combinations of three different pH (4 ± 0.05, 7 ± 0.05 and 9 ± 0.05) and three different temperatures (15 ± 0.5°C, 30 ± 0.5°C and 45 ± 0.5°C), shows very fast rate of reaction which further increases with increase of pH and temperature. Carbonyl oxygen of carbamate and phosphate oxygen of organophosphate were found to be common ligating sites among all the complexes. Formed metal complexes were found to be highly stable and water insoluble on interaction with essential metal ions in solvent medium as well as over silica. Density functional theory (DFT) calculations not only reinforced the experimental observations, but, after a wide computational conformational analysis, unraveled the nature of the high stable undesired species that consist of pesticides complexed by metal ions from the soil. All in all, apart from the direct toxicity of pesticides, the indirect effect by means of complexation of free metal ions impoverishes the soil.

## Introduction

Pesticides are integral part of modern agriculture (David, 1998; Chen et al., 2014), which increased tremendous chemical pressure on agricultural soil and in turn has affected entire ecosystem (Karpouzas et al., 2014; Kumar et al., 2015c; Kienzler et al., 2016; Rivera-Becerril et al., 2017). Environmental Protection Agencies (Gallegos Saliner et al., 2006; Balderacchi et al., 2014) are very cautious and place several checks before passing any chemical to act as a pesticide (Krupadam et al., 2003; Gallegos-Saliner et al., 2008). Nevertheless, few checks like effect on micronutrients (Senesi, 1992) that affect the soil fertility are still not completely properly addressed (Piccolo et al., 1992; Shirzadi et al., 2008). One of such factors that affect soil fertility is metal depauperating, naturally operated by microorganisms and other natural chelating agents that are prevailed in the natural environment (Tullberg, 2010). Recent studies have alarmed the excess usage of pesticides could possibly cause pernicious effect on micro flora that potentially degrading the soil fertility (David, 1998; Glover-Amengor and Tetteh, 2008; Wasim et al., 2009). Another study highlighted the alarming decrease in the percentage of world soil metal ions (zinc, 49%, iron, 12%, manganese, 5%, copper, 3%, boron, 33% and molybdenum, 11%; Long et al., 2004; Alloway, 2008; Schroeder et al., 2013) leading to deterioration in soil quality (Racke et al., 1997). However, the explanation for the latter fact has still to be unfolded and it might be the possible that similar to former case, pesticides might hold the prime reason for worsened soil quality (Prado and Airoldi, 2002; Sharma et al., 2011). Studies on metal- chelation system in plant are only focused on its usage in phytoremediation system. On the contrary, pesticides have the similar ligating sites and can act as potential agents for complexation. In this context, one of the overlooked property of the pesticides, which relates to its ligating ability or chelating effect (Gupta et al., 2011, 2012) with the most representative natural trace metal ions present in soil have been analyzed in the present work.

**Figure 1:**
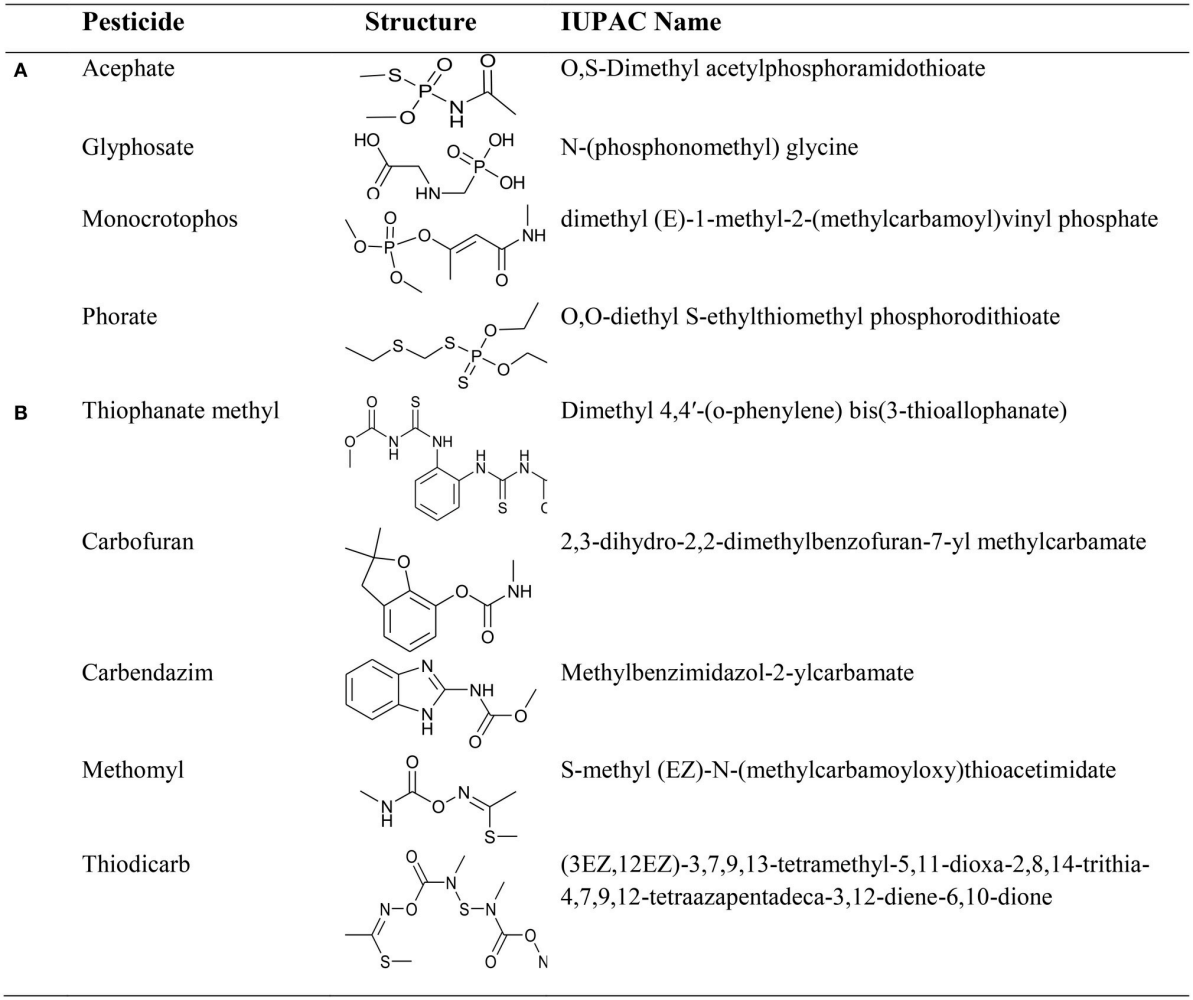
Structures of **(A)** organophosphate and **(B)** carbamate pesticides used in our studies.

Considering the chelating effect of pesticides as the foundation of our study, we have examined the interaction of first row essential trace transition metal ions (Mn^2+^, Fe^3+^, Co^2+^, Ni^2+^, Cu^2+^, and Zn^2+^) with organophosphate pesticides (acephate, glyphosate, monocrotophos, and phorate) and carbamate pesticides (carbendazim, carbofuran, methomyl, thiodicarb, and thiophanate methyl) (see Figure 1) in terms of physical and chemical aspects. The theoretical DFT calculations have been carried out to reinforce and explain some of the questions that are practically impossible to be addressed by chemical techniques, giving not only the affinity of the different studied pesticides to coordinate metal ions, but to go deeper into detail about coordination chemistry, missing from just an experimental point of view.

## Materials and methods

### Materials and reagents

Active components of technical grade of pesticides were received from Gautami Ltd. India. Acquired purity of the pesticides was obtained through recrystallization and comparison with the IR and NMR data of the standard samples before experimentation. Analytical grade metal salts and solvents were purchased from LobaChemie, India. Distilled water used for experimentation was obtained by using Milli-Q water purification system from Millipore, (Bedford, MA, USA).

Analytical Instruments: UV-visible spectrophotometer (Shimadzu-1800), FTIR spectrophotometer (Shimadzu-8400s), NMR spectrophotometer (Bruker Avance III, 400 MHz), Mass spectrometer (Waters, Q-TOF Micromass), Surface Electron Microscopy (JEOL Model JSM - 6390LV) and TG Analyser (Perkin Elmer STA 6000) were used for characterization of the samples. The energy-dispersive X-ray analysis (EDAX) plots of the samples were recorded with an FEI Quanta 200 FEG instrument.

Individual pesticide was passed in proper mobile phase (generally, mili-Q water) over well-dried metal salt adsorbed silica. On diffusion of pesticide solution with silica, evident color change was observed highlighting the metal pesticides interaction. Expected changes on the surface of silica were analyzed by Field-Emission Scanning Electron Microscopy and Energy Dispersive X-ray Spectroscopy for each of the products. Metal-pesticide complexes were synthesized using 1.0 mM of metal ion and known amount of pesticides (ratio obtained by applying Job's method, as summarized in Table S1) dissolved in 50.0 mL of solvent (water was taken as solvent for organophosphates and methanol for carbamates) in a round bottom flask with continuous stirring. Reaction progress or product formation was monitored by using the UV-visible spectrophotometer. Product precipitated after the completion of reaction was filtered out by using vacuum through G4 glass crucible. Products were washed thoroughly with water followed by ethanol and dried in a hot air oven at 60°C for 12 h and then kept in a desiccator for 72 h before characterization (spectral data are included in the Supporting Information). The progress of reaction was investigated UV-vis spectrophotometrically for all the possible combinations of three different temperatures (at 15 ± 0.5°C, 30 ± 0.5°C, and 45 ± 0.5°C) and three distinct pH values (4 ± 0.05°C, 7 ± 0.05°C, and 9 ± 0.05°C) using same synthetic methodology as stated above. For the purpose of finding chemical stability of metal-pesticide complex, the complex was added into 100.0 mL pH adjusted deionized water (pH was adjusted by the use of HCl and NaOH for the sake of obtaining four different pH values 3.0, 5.0, 7.0, and 9.0). After every 24 h 5.0 mL samples were taken out from the mixture and digested with nitric acid. Atomic Absorption Spectroscopic analyses of such samples were performed in triplicate. In none of the case metal leaching was observed. Thermal stability of metal complexes is determined by applying thermogravimetric analysis (TGA/DTA) between ambient room temperature to 850°C with a heating rate of 10°C/min. Photolytic behavior of complexes was analyzed by passing UV radiation. After every 5 min, complex was exposed with UV- radiation of the λ_max_ (300–450 nm) of the metal-pesticide complex in trifluoroacetic acid was passed and percentage of absorbance obtained with respect to initial absorbance after 8 h of experimentation is tabulated in Table S2.

### Computational details

The density functional calculations were performed at the GGA (Generalized Gradient Approximation) level with the Gaussian09 set of programs (Frisch et al., 2009), with the M06L correlation-exchange functional (Zhao and Truhlar, 2008). The electronic configuration of the molecular systems was described by the standard basis set triple zeta valence plus polarization (TZVP keyword in Gaussian) of Ahlrichs and co-workers (Schaefer et al., 1992). All open-shell species were treated using the unrestricted formalism. The geometry optimizations were performed without symmetry constraints, and the nature of the extrema was checked by analytical frequency calculations. Furthermore, connections between minima and transition states were confirmed by calculation of the intrinsic reaction paths. The Gibbs energies discussed throughout the text contain thermal, ZPE, and entropic corrections. Solvent effects were estimated in single point calculations on the gas phase optimized structures with triple zeta valence of the Dunning's correlation consistent basis sets (cc-pVTZ keyword in Gaussian) (Kendall et al., 1992) using the M06L functional. Solvation energies were evaluated with the polarizable conductor calculation model CPCM using water as solvent (Barone and Cossi, 1998). Therefore, all reported Gibbs energies are M06L/cc-pVTZ//M06L/TZVP electronic energies in solvent with added thermal, ZPE, and entropic corrections obtained at the M06L/TZVP level of theory.

%VBur Calculations: The buried volume calculations were performed with the SambVca package developed by Cavallo et al. (Jacobsen et al., 2009) The radius of the sphere around the origin placed two Å below the metal center was set to 3.5 Å, while for the atoms we adopted the Bondi radii scaled by 1.17, and a mesh of 0.1 Å was used to scan the sphere for buried voxels. The steric maps were evaluated with a development version of the SambVca package (Poater et al., 2009).

## Results and discussion

Soil transfers the metal nutrients to the plant system by using some biosynthetic chelates. Simultaneously, the pesticides that are employed on the same soil surface could act as potential competitors to chelate the metal ions (Kumar et al., 2015a). Unfortunately, soil is a complex phase (having inorganic, organic and biological components; along with their inter component interactions) and it is difficult to see impact of pesticides on the essential metal ions present in soil. Thus, in order to investigate the impact of pesticides in presence of metal ions, simulative method was used. Bearing in mind two third parts of soil consist of the silica, metal ions (separately each single metal ion) were adsorbed on silica. To point out that in the silica most of the free metal ions are adsorbed over. Thus, once packed in a column the silica, the metal ions are part of the stationary phase. Pesticide dissolved in the solvent acted as the mobile phase, when passed over metal adsorbed silica gel. The color change (see Figures S1–S3 for further details) was a clear evidence of the formation of metal complexes (discussed *vide infra*). Those latter species moved down with the mobile phase, showing weak interaction of silica with the metal complex compared to silica-metal interaction. Probably this is due to conversion from dipole-ion interaction (silica-metal interaction) to weaker dipole-induced dipole interaction (silica- metal complex).

**Figure 2:**
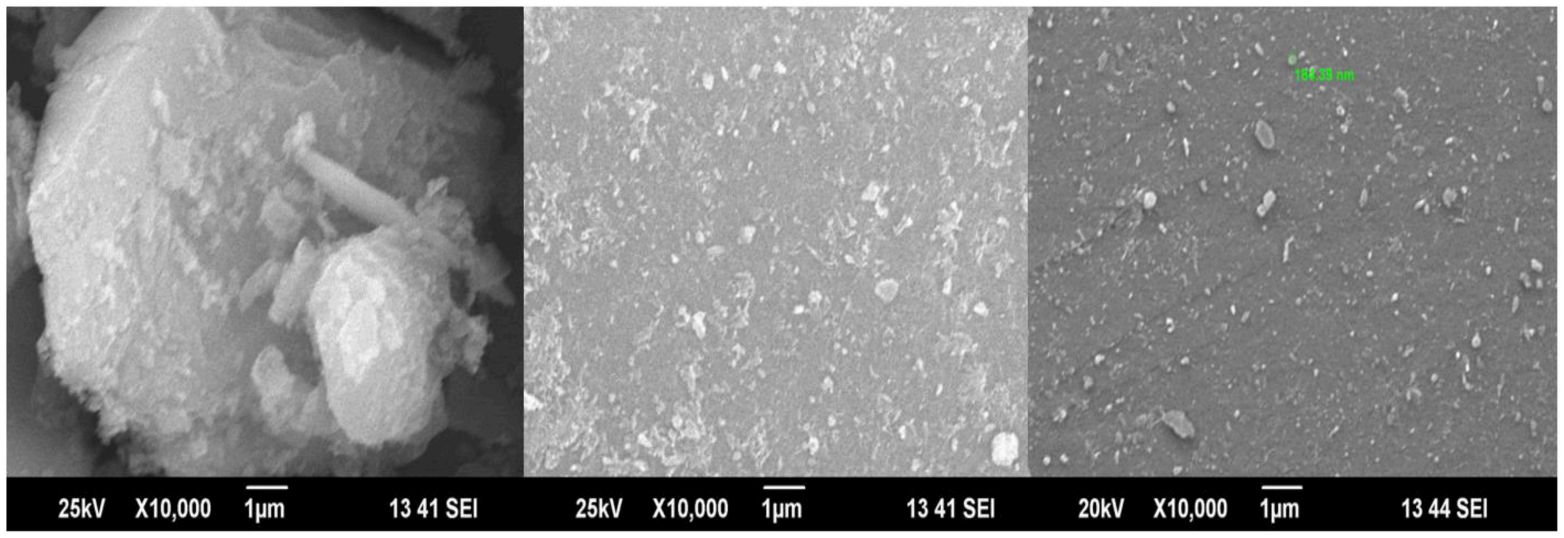
SEM picture for iron(III) adsorbed silica **(Right)**, SEM picture for iron(III) adsorbed silica after passing monocrotophos in a mobile phase **(Middle)**, SEM picture of silica leached Fe(III) complex of monocrotophos, when passed through Fe(III) adsorbed silica **(Left)**.

Next, the morphology of the silica surface was checked by using SEM with EDX analysis. Figure 2 shows SEM- EDX picture of silica alone; silica + cobalt(II) before passing monocrotophos over it and then after passing monocrotophos on iron(III) impregnated silica, where two different kinds of surface morphologies were obtained, the first surface morphology indicated monocrotophos adsorbed over silica at a place no cobalt(II) were observed, while the other kind of surface morphology was observed after interaction of monocrotophos with Fe(III) on silica surface (obtained from EDX analysis). Details of similar analysis of thiophanate methyl with Co(II) ion is given in the Supplementary Material.

Pesticide may also interact with metal nutrients in a soluble media; their interaction with the pesticides in liquid medium was therefore checked (wherever possible, water was chosen as a solvent, depending on the solubility of the pesticide; Gomez et al., 2012). Both the pesticide and metal ion were mixed in a solvent (the solvent in which column was performed) in a ratio obtained after applying Job's spectrophotometric method (Job, 1964) (given in Supplementary Material) and stirred on a magnetic stirrer. Most of the pesticides quickly formed complexes with metal ions (see Supplementary Material). The color of the compounds was found identical to the compound extracted from the column. Characterization of the formed complexes obtained through both methods was evaluated using UV-vis and IR spectra and were found similar.

**Figure 3:**
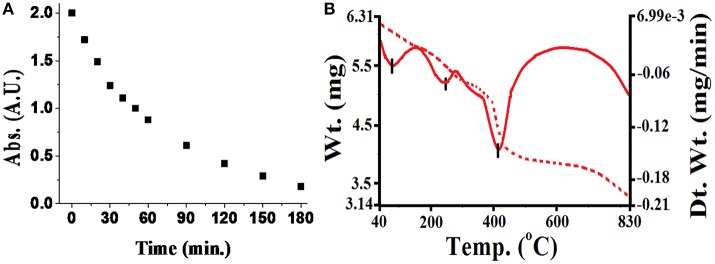
**(A)** Rate of complex formation of monocrotophos with iron at room temperature (absorbance were taken at λ_max_ equal to 218 nm with concentration of 300 ppm). **(B)** TGA/DSC behavior of monocrotophos−iron complex.

During investigation of the reaction(s) through UV-vis spectrophotometric technique, it was observed that pesticides quickly form complexes with metal ions adsorbed on silica. The rate of such fast reactions was extremely difficult to determine on the silica surface. However, the reaction rate in a liquid medium was found slower as well as informative to examine the reaction process (although, some carbamate pesticides like thiophanate methyl with Cu(II), methomyl with Fe(III) reacts quickly). For that purpose, changes observed in absorbance at λ_max_ of the pesticide in presence of the metal ion in liquid medium was evaluated in Figure 3. Attained results (Table 1) exhibited altered interactive behavior pattern such as thiophanate methyl was found to interact quickly with Cu(II) and Ni(II) metal ions, whereas acephate steadily initiated interaction with metal ions (compiled in tabular form in Table S2). Such an interaction could be explained on the basis of HSAB principle. The cases, where N/S is also a donor site/coordinating site in addition to “O” (e.g., thiophanate methyl; Gupta et al., 2011) showed more reactivity toward Cu(II) ion, and show intermediate behavior between hard and soft bases. On the other hand, pesticides having only “O” as donor site (either one or more than one) react faster with Fe(III) (Gupta et al., 2012). Unlike other pesticides, carbofuran with almost all the essential metal ions react very slowly, because of unavailability of strong chelation sites in it. Thus, the formation of stable chelate is another reason for metal-pesticide complexation. But, carbofuran (having only “O” as donor site) at least reacts with Fe(III) and Mn(II) and with other metal ions reactivity was too low to be determined. On characterization of products, phosphoryl oxygen in organophosphate and carbonyl oxygen in carbamate ligands was found to be common (see Supplementary Material). This indicated that every organophosphate or carbamate has high probability of interaction with essential metal ions of soil, differentiated only by the rate of interaction.

**Table 1:** Interactive analysis of the interaction of pesticides with a selection of metal ions.

**Name of the complexes**	**% of ligands left with time (h)**					
	**1**	**2**	**3**	**5**	**8**	**24**
**METAL**−**PESTICIDE COMPLEX**						
Cu(II)−Carbendazim	75	57	28	26	24	12
Fe(II)−Carbendazim	77	42	20	19	16	08
Mn(II)−Carbofuran	63	56	53	47	37	19
Fe(II)−Carbofuran	76	63	61	41	32	24
Fe(II)−Thoidicarb	80	63	34	30	25	06
Zn(II)−Thoidicarb	79	61	33	29	24	5
Fe(II)−Methomyl	57	45	43	38	28	13
Cu(II)−Methomyl	80	73	72	68	55	42
Fe(II)−Thiophanate methyl	92	86	82	77	68	58
Cu(II)−Thiophanate methyl	17	16	15	15	09	05
Fe(II)−Acephate	75	59	48	33	21	16
Cu(II)−Acephate	68	51	38	24	14	08
Fe(II)−Glyphosate	35	19	15	14	09	06
Cu(II)−Glyphosate	45	31	21	18	14	11
Fe(II)−Monocrotophos	59	37	24	15	12	09
Cu(II)−Monocrotophos	68	46	33	26	17	12
Fe(II)−Phorate	64	43	32	26	18	13
Cu(II)−Phorate	42	28	18	12	10	06

**Figure 4:**
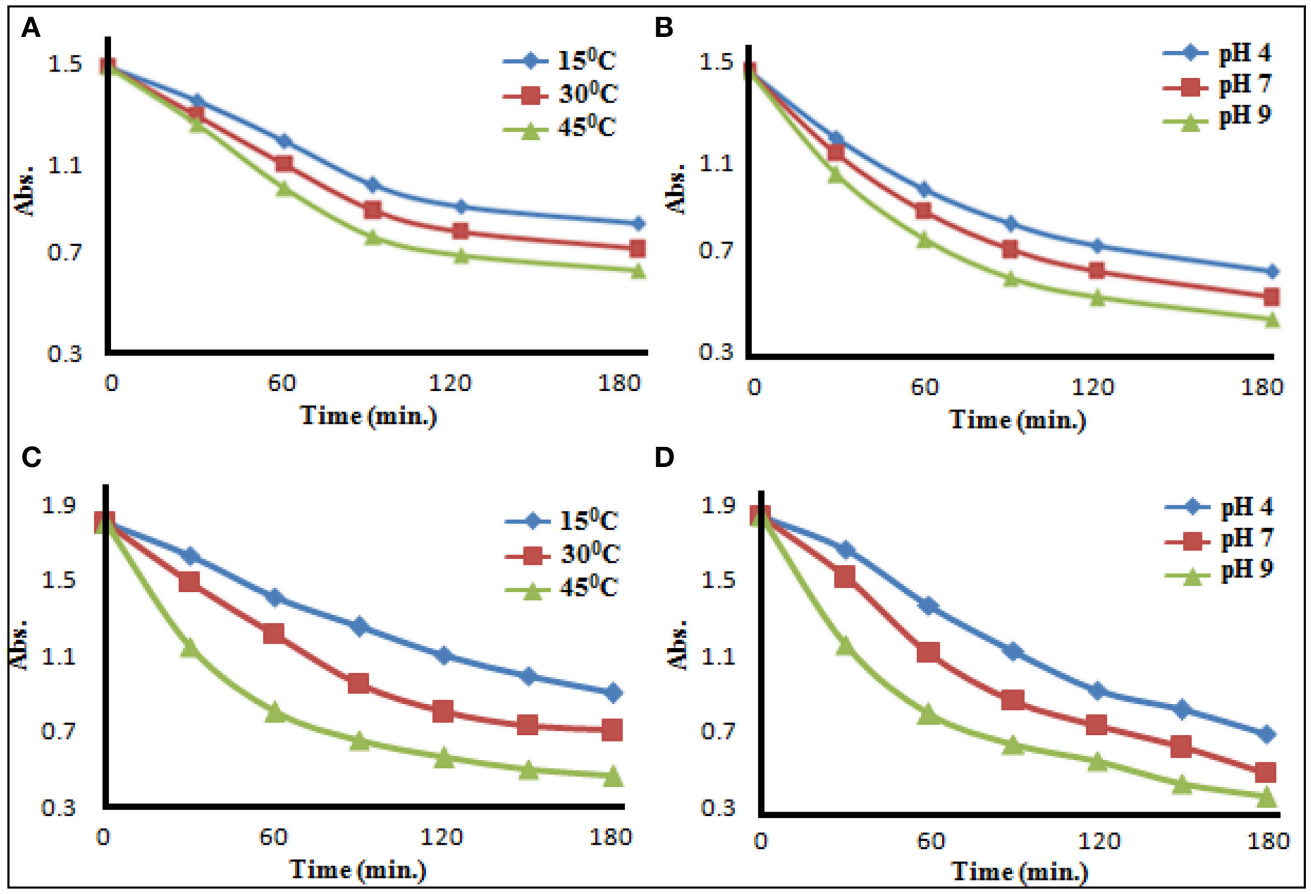
Effect of temperature **(A)** and pH **(B)** on glyphosate−copper(II) complexation, and Effect of temperature **(C)** and pH **(D)** on carbendazim −copper(II) complexation.

In the environmental conditions, soil may have a pH variation from 2 to 12 and temperature variation from 0 to 50°C and rate of metal-pesticide interaction might be influenced by effect of temperature and pH. Therefore, above stated reactions was carried out at all possible combinations of three different temperatures (15 ± 0.5°C, 30 ± 0.5°C, and 45 ± 0.5°C) and three pH values (4 ± 0.05, 7 ± 0.05, and 9 ± 0.05). In most of the cases, formed product was found insoluble in the medium of the reaction and therefore λ_max_ of the n→π^*^ electronic transition of pesticide was chosen for the observation of rate of conversion of pesticide to pesticide-metal complex. With increase of temperature, rate of reaction was found to increase. At 25°C, when reaction was performed at different pH, rate of the reaction was found to increase with the increase of pH (representative results are showcased in Figures 4 and Supplementary Material). Thus, the rate of formation of metal–pesticide complex is expected to be high in summer days as well as in neutral to basic soil. Organophosphate itself creates acidic environment in soil/water medium, the rate of formation of metal-pesticide complex was expectedly obtained lower than that of carbamate pesticides. Almost all the complexes formed/synthesized (between pesticide and metal ion at different pH and temperature) were found insoluble in all tested laboratory solvents. Since, most of the methods of recovery, residue or half life time analysis of the pesticide in a specific media are dependent on the solubility of the pesticides (Chapalamadugu and Chaudhry, 1995; Maier and Griepink, 1995; Pehkonen and Zhang, 2002; Andreu and Pico, 2004; Kumar et al., 2013, 2015b), all these methods are now in a serious doubt in real world sample, where the presence of metal ions is possible/probable.

In order to analyse the site of interaction, synthesized metal-pesticide complexes were characterized by using UV-vis, IR, NMR spectrophotometric and Mass spectrometric techniques (See Supplementary Material). Result evaluated through the comparative IR spectra of organophosphates with its corresponding metal complexes depicted, 20–30 cm^−1^ down shift of P = O band (comes originally in between 1,250 and 1,050 cm^−1^), along with, remarkable decrease in intensity and broadening in the peaks (Heineke et al., 1994; Murugavel et al., 2008). P-NMR up field shift up from a range of 25–65 to 0–10 ppm was observed (Sala et al., 2004). Such changes occurred near the P = O bonds in case of organophosphate pesticides, representing the active involvement of this group in complex formation with metal ions. Since neurotoxicity of organophosphate (most of the time) is based on removal of the alkoxy group from phosphate and the metal ion interaction with the phosphate oxygen may therefore affect the neurotoxicity of the organophosphate.

In case of carbamate, participation of carbamate oxygen and formation of imine was found common. That might be a resultant due to existence of keto-imine tautomerization in carbamates. In IR spectra of carbamates, generally N-H stretching band comes in between 3,150 and 3,400 cm^−1^ and N-H bending band was observed around 1,600 cm^−1^. The N-H stretching band(s) disappear on complex formation, while at around 1,650 cm^−1^ a medium intensity was observed in place of N-H bending band. The band at 1,650 cm^−1^ is an indication of formation of imine (C = N) group. Also, the keto group of uncomplexed carbamate that was observed at about 1,750 cm^−1^ disappeared on complex formation, which may indicate the participation of carbonyl oxygen in complex formation. The evidence of keto-imine tautomerization was also obtained from ^1^H-NMR spectral study of carbamate and its formed product. N-H moiety of carbamate, which initially was observed at around 10 ppm, was found to be disappeared on complex formation. The formation of a stable chelate species seems to be always a determining factor for complex formation. For example, carbendazim, methomyl, thiodicarb, and thiophanate methyl quickly form stable complexes with essential metal ions, while carbofuran because of unavailability of second legating site (except having carbonyl oxygen) do not easily form the complexes with the same metal ions (detailed spectroscopic data are tabulated in the Supplementary Material).

Pesticides may be degraded by the effect of temperature, pH of the soil/medium, UV radiation or bacterial degradation (Chapalamadugu and Chaudhry, 1995; Maier and Griepink, 1995; Pehkonen and Zhang, 2002; Andreu and Pico, 2004; Kumar et al., 2015b). Chemical stability of the formed complexes was measured below pH 3.0 by using UV-vis spectrophotometric method. It was observed that there were initially small but sharp dip in decomposition curve, but after some time, it remains almost constant when irradiated with the UV radiation exactly equal to the λ_max_ of the pesticide-metal complex (see Supplementary Material). Thermal stability of the formed complexes was determined by using (TG/DTA) technique and found extremely stable (see Table 2). In most of the cases, pesticide-metal chelate was found non-decomposable before 500°C in air medium and until 850°C of temperature in nitrogen (inert) atmosphere (see Table S3).

**Table 2:** Summary of thermal analysis for metal−organophosphate complexes.

**Sample**	**Stage**	**T_i_ (°C)**	**T_p_ (DTG_max_) (°C)**	**T_f_ (°C)**	**Mass loss (%)**
Fe(II)−A	1st	37	86	100	8
	2nd	101	222	300	21
	3rd	301	347	500	15
	4th	501	725	700	6
Fe(II)−G	1st	37	84	100	7
	2nd	101	250	300	22
	3rd	301	415	500	15
	4th	501	475	700	7
Fe(II)−M	1st	37	86	100	6
	2nd	101	263	300	18
	3rd	301	370	500	15
	4th	501	512	700	11
Fe(II)−P	1st	37	–	100	1
	2nd	101	186	300	56
	3rd	301	330	500	5
	4th	501	–	700	2

*A, acephate; G, glyphosate; M, monocrotophos; P, phorate; Ti, initial temperature, Tp, peak temperature; Tf, final temperature*.

To quantify the capacity of complexation of pesticides by different metal ions, we envisaged DFT calculations for two organophosphates, glyphosate and phorate, depicted in Figure 4. Bearing the aqueous media in the soil, thus with water in excess, the metal ions must be surrounded by water molecules and thus the metal-aqua complexes have been studied to be as the energy reference for each of the studied metal ions. In all the cases metal complexed with six water molecules (see Table 3) turn out to be an ideal recipe, since the metal-metal interaction is rather unusual (Poater et al., 2006), and thus water will be the most feasible agent to interact with the metal ions. According to calculations iron and also nickel are more prone to coordinate six water molecules. Actually, the complexes with five and four water molecules are located more than 5 and 10 kcal/mol higher in energy, respectively, whereas for the other metal ions decoordination of a water molecule from the metal ion seems to be more facile, being even in equilibrium in particular for copper, manganese and zinc, where the decoordination costs around only 1 kcal/mol. To unravel the nature of the driving force for dissociation of either one or two water molecules, analysis by Mayer Bond Orders (MBO) was carried out, see Table 4, confirming that the strength of the M-O bonds is a priori responsible for the affinity to decoordinate water molecules, and thus more prone to recoordinate potentially pesticides as ligands. The MBOs for Fe(III) and Ni(II) are 0.446, 0.284, respectively, in correlation with the highest binding energies, whereas the MBO for Co(II) is only 0.231, and consequently, even lower than for Cu(II) and Zn(II).

**Table 3:** Complexation by water molecules of metal ions (in parentheses the ground state multiplicities for each metal ion, energies in kcal/mol).

**Metal**	6 ^*^ H_2_O	5 ^*^ H_2_O	4 ^*^ H_2_O
Co^II^ (quadruplet)	0.0	3.4	4.2
Cu^II^ (doublet)	0.0	1.3	6.2
Fe^III^ (sextuplet)	0.0	9.6	22.3
Mn^II^ (sextuplet)	0.0	0.8	2.5
Ni^II^ (triplet)	0.0	5.7	10.7
Zn^II^ (singlet)	0.0	1.3	2.2

**Table 4:** Mayer Bond Order (MBO) of the weakest M−O bond for each water complexed species.

**Metal**	6 ^*^ H_2_O	5 ^*^ H_2_O	4 ^*^ H_2_O
Co^II^ (quadruplet)	0.231	0.294	0.353
Cu^II^ (doublet)	0.240	0.365	0.397
Fe^III^ (sextuplet)	0.446	0.474	0.589
Mn^II^ (sextuplet)	0.208	0.228	0.309
Ni^II^ (triplet)	0.284	0.302	0.367
Zn^II^ (singlet)	0.269	0.281	0.403

For all species the ground state multiplicity has been checked and, for the sake of consistency, the corresponding state for metal ion is maintained whatever pesticide ligands surround it afterwards (Manrique et al., 2015). Anyway, and fortunately no multiplicity crossing is observed (Poater et al., 2014; Gil-Sepulcre et al., 2016).

As expected, replacing the water molecules by the organophosphate pesticides, glyphosate and phorate, included in Figure 1 results in a thermodynamic preference for the latter species as ligands with respect to the coordinated water molecule. Going into details, for the organophosphate pesticide phorate the results are collected in Table 5, whereas in Table 6 the values for the organophosphate pesticide glyphosate are reported with the screening of the most stable geometry around the metal for each combination (see Table S5 for all the other isomers). Both pesticides are demonstrated to strongly coordinate to the metal ions, and thermodynamically even more stable when dealing with two or three ligand molecules. However, coordination of only one pesticide as a ligand to a metal ion is favored energetically in all the studied cases, as well. With all the above results, it seems reasonable to point out that pesticides bind very strongly to metal ions, thus having the potential to devoid the soil of metal ions. Unexpectedly, Sala et al. (2004) although coordination of the phorate to metal ions is favored over coordination of water, this preference is weak, specifically for Mn(II). Bearing the low concentration of pesticide with respect to water molecules, some free metal ions might be still left in the soil. However, for a more strongly coordinating pesticide, confirmed by high exothermicity when bonded to the respective metal ions, i.e., pesticide glyphosate, the presence of that pesticide would “kill” the soil completely.

**Table 5:** Exchange of water ligands by pesticides (P = phorate) in metal ions (in parentheses the ground state for each metal ion, energies in kcal/mol relative to the corresponding M(OH2)_6_ reference).

**Complex**	**Co^II^**	**Cu^II^**	**Fe^III^**	**Mn^II^**	**Ni^II^**	**Zn^II^**
6 ^*^ H_2_O	0.0	0.0	0.0	0.0	0.0	0.0
1 ^*^ H_2_O+ 1 ^*^ P	1.6	−6.9	8.7	7.9	3.7	−3.1
2 ^*^ H_2_O+ 1 ^*^ P	−0.4	−12.1	−1.5	3.5	3.7	−5.2
3 ^*^ H_2_O+ 1 ^*^ P	−2.6	−15.1	−6.0	3.1	−1.9	−1.9
4 ^*^ H_2_O+ 1 ^*^ P	−5.7	−14.6	−9.3	−0.9	−6.3	−3.7
2 ^*^ P	−11.4	−25.0	−6.9	−0.9	−6.6	−14.3
1 ^*^ H_2_O+ 2 ^*^ P	−7.7	−25.3	−13.5	−1.9	−9.1	−8.2
2 ^*^ H_2_O+ 2 ^*^ P	−6.9	−19.2	−6.1	2.0	−10.2	−4.8
3 ^*^ P	−9.3	−23.2	−18.0	1.3	−12.6	−2.8

**Table 6:** Exchange of water ligands by pesticides (P = glyphosate) in metal ions (in parentheses the ground state for each metal ion, energies in kcal/mol relative to the corresponding M(OH2)_6_ reference).

**Complex**	**Co^II^**	**Cu^II^**	**Fe^III^**	**Mn^II^**	**Ni^II^**	**Zn^II^**
6 ^*^ H_2_O	0.0	0.0	0.0	0.0	0.0	0.0
1 ^*^ P	−5.5	−4.7	−4.5	−5.6	0.7	−9.5
1 ^*^ H_2_O+ 1 ^*^ P	−10.5	−20.3	−10.6	−10.0	−1.8	−12.3
3 ^*^ H_2_O+ 1 ^*^ P	−17.1	−21.5	−27.1	−13.4	−17.5	−15.3
2 ^*^ P	−32.5	−41.0	−50.4	−28.5	−34.4	−33.0

**Figure 5:**
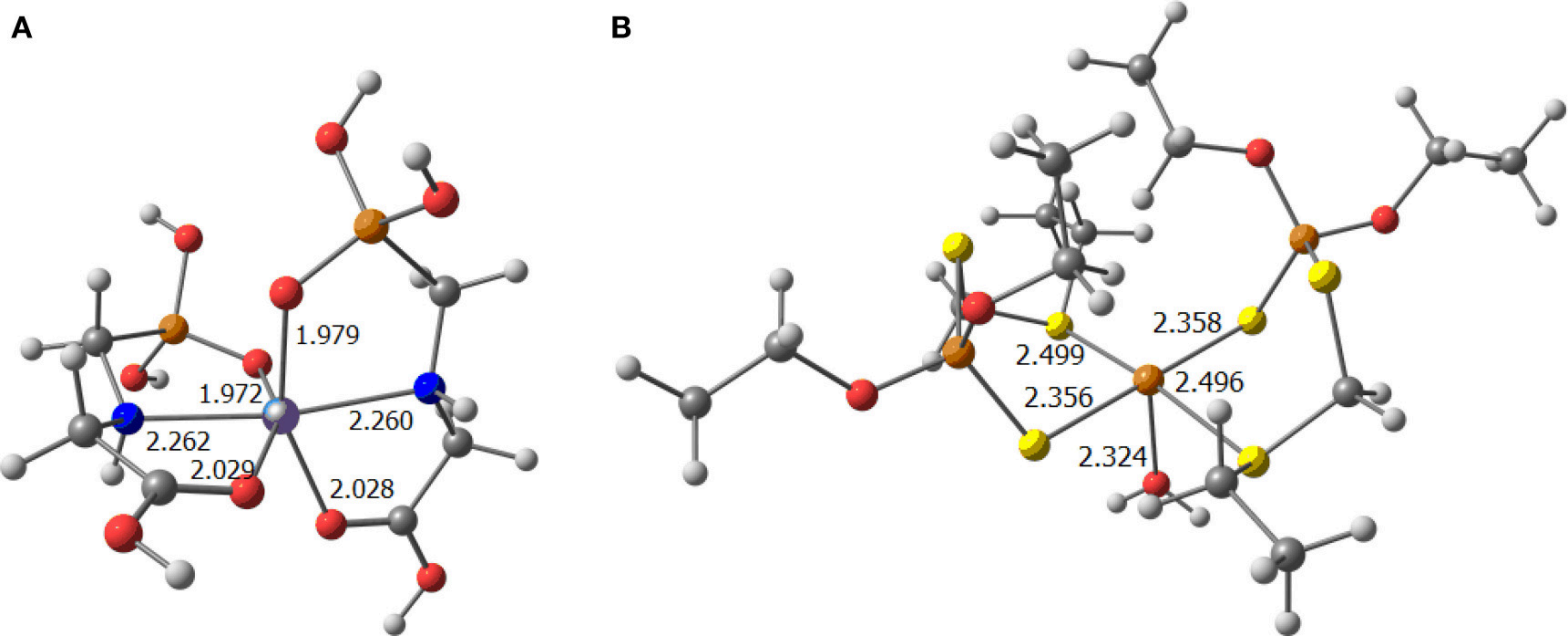
Optimized complexes bearing **(A)** Fe(III) as a metal and two glyphosate ligands; and **(B)** Cu(II) as metal and two phorate ligands and a water molecule (selected distances in Å).

Analysing the performance of each metal ion, iron is the metal ion more prone to exchange the water ligands by the pesticide molecules (see Figure 5A).

Further, to point out that copper becomes competitive with phorate because of its known affinity for sulfur based ligands. The two pesticide moieties bonded to copper, displayed in the same plane of the metal in Figure 5B, reveal that this metal prefers the square planar geometry because the fifth ligand, i.e., the water ligand, is not strongly bonded to copper. All these results reinforce the experimental observations, that the presence of more pesticides in the soil leads to the formation of strong complexes with the metal ions, that once formed, are nearly impossible to be degraded, thus devoiding the soil of the essential metal ions, which in-turn is required by the plant source.

Moreover, a deeper structural analysis helps to understand how the environment changes when the metal center is exchanged. To study the steric properties around the metal, we calculated the %VBur on the position of the metal due to the ligands bonded to it, using SambVca2 package developed by Cavallo et al. (Poater et al., 2009) where the buried volume is the measure of the occupation of the first coordination sphere of the metal (Heineke et al., 1994). It also realized a more detailed analysis to assess %VBur in individual quadrants around the metal center and represent steric contour maps (Figure 5). Splitting the total %VBur into quadrant contributions quantifies any asymmetry, in the way the ligand wraps around the metal (Falivene et al., 2016). This analysis shows how to change the shape of the center of the complexes exchanging either the ligands around the metals, or the nature of the metal itself (Ahmed et al., 2013; Poater et al., 2013).

**Figure 6:**
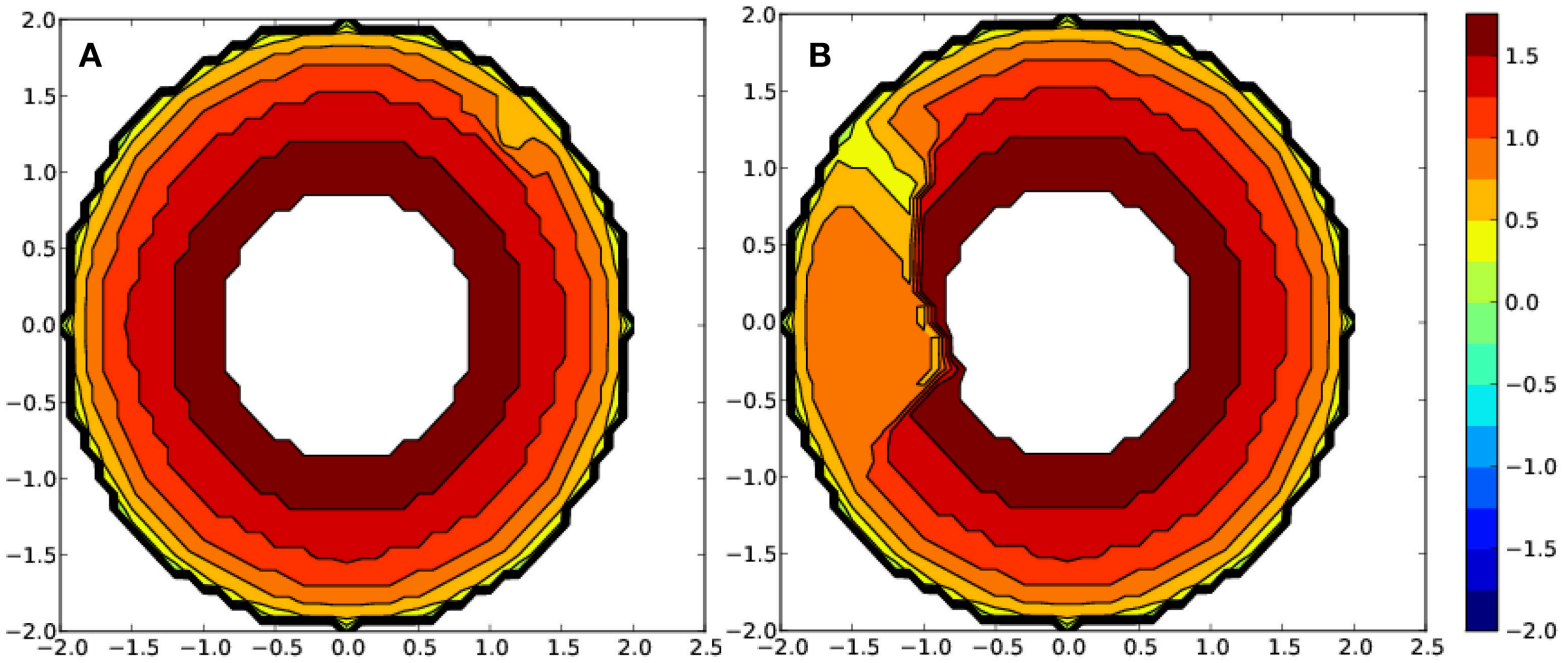
Steric maps for metal complexes with two glyphosate ligands bearing as a metal **(A)** Fe and **(B)** Mn. The metal is at the origin and the isocontour curves of the steric maps are given in (Å).

As shown in Figure 6 for the complex bearing two glyphosate pesticides as ligands, a quite different steric environment based on the metal center is appreciated. The metal environment for the Fe-based complex is significantly more sterically congested than for the Mn-based one. In detail, the calculated percent buried volume (%VBur) for Fe is 5.0% higher (see Table S4 for further details). Moreover, one quadrant for corresponding Mn complex is specially occupied, whereas three are about 7–9% less occupied. The more sterically hindered iron center favors its stability bearing two ligands around the metal, rationalizing the 21.9 kcal/mol of further stabilization that means the substitution of the six water molecules. On the other hand, the complexes bearing three phorate ligands can also be rationalized through the steric maps, where for copper the %Bur is 2.8% higher, rationalizing the stabilization by 24.5 kcal/mol with respect to the Mn counterpart, which shows a more distorted hexacoordinated geometry, with some weaker Metal-P bonds. Overall, the more sterically hindered metal environment, together with a higher degree of symmetry around it, the higher the stability of the complexes product of the interaction of the metals with the pesticide ligands is. Thus, it is more facile to allocate the ligands around the metal.

Analysis of the Natural Bond Order (NBO) charges in Table 7 shows that that the exchange of water ligands by the tridentate glyphosate stabilizes the high positive charge on the metal, bearing the oxygen (O_2_) next to the phosphorous nearly twice more negative charge than the contribution of the two coordinating atoms, O_1_ and N.

**Table 7:** Natural Bond Order (NBO) charges on the atoms of the pesticide (P = glyphosate) bonded to the corresponding metal (O1 = oxygen bonded to the carbon, O2 = oxygen bonded to the phosphorous), and charge on that metal (in e).

**Complex**	**Atom**	**Co^II^**	**Cu^II^**	**Fe^III^**	**Mn^II^**	**Ni^II^**	**Zn^II^**
3 ^*^ H_2_O+ 1 ^*^ P	M	1.928	0.867	2.808	3.072	1.384	1.359
	O_1_	−0.264	−0.256	−0.219	−0.299	−0.262	−0.631
	O_2_	−0.510	−0.482	−0.355	−0.518	−0.492	−1.093
	N	−0.264	−0.200	−0.213	−0.297	−0.254	−0.632
2 ^*^ P	M	1.866	0.822	2.747	3.023	1.324	1.304
	O_1_	−0.268	−0.278	−0.220	−0.278	−0.257	−0.608
	O_2_	−0.501	−0.524	−0.420	−0.524	−0.490	−1.098
	N	−0.271	−0.216	−0.229	−0.294	−0.255	−0.636
6 ^*^ H_2_O	M	2.010	0.990	2.890	3.114	1.485	1.425

## Conclusions

Through the investigation of nine studied compounds, it was found that both the carbamates and organophosphate pesticides form quick and stable complexes with essential metal ions of soil/water medium. These complexes were insoluble in water (as well as in most of the laboratory solvents) at almost all pH, their transfer to plant/animal body is therefore not possible. Decomposition of the formed product seems to be difficult (on ground of effect of change of pH, temperature and UV radiation). To reinforce the experimental observations, the theoretical studies have been carried out to explain the high stability of the metals complexed with pesticides. Further, biochemical and agricultural aspects are yet to be opened up to support the fact that pesticide could lead to the depression or disappearance of the trace metal ions for plant/animal body. It would be unwise to underestimate the significance of the pesticide impact on soil fertility.

## Author contributions

NU initiated the project and designed all the experimental work. VK did entire experimental work on organophosphate pesticide and SK on carbamate pesticides. MC and AP the calculations; LC, NU, MC, and AP wrote the paper and planned the paper.

### Conflict of interest statement

The authors declare that the research was conducted in the absence of any commercial or financial relationships that could be construed as a potential conflict of interest.
