# Supplementary material for: Pesticides Curbing Soil Fertility: Effect of Complexation of Free Metal Ions
Source: Front Chem. 2017 Jul 4;5:43. doi: 10.3389/fchem.2017.00043 (PMC5495828; doi:10.3389/fchem.2017.00043)
Supplement: Supplementary file 1 [file Table1.docx]

| **Pesticides** | **% Remained after 8hrs Decomposition of Metal Complexes** | | | | | |
| --- | --- | --- | --- | --- | --- | --- |
|  | Mn(II) | Fe(II) | Co(II) | Ni(II) | Cu(II) | Zn(II) |
| Metal-Pesticide complex | | | | | | |
| Acephate^a^ | 46 | 51 | 51 | 49 | 53 | 48 |
| Carbendazim^b^ | 61 | 64 | 62 | 58 | 65 | 52 |
| Carbofuran^b^ | 56 | 59 | 54 | 55 | 60 | 58 |
| Glyphosate^a^ | 47 | 49 | 51 | 48 | 55 | 48 |
| Methomyl^b^ | 54 | 61 | 57 | 59 | 61 | 56 |
| Monocrotophos^a^ | 46 | 47 | 45 | 50 | 52 | 47 |
| Phorate^a^ | 46 | 51 | 49 | 47 | 53 | 48 |
| Thiodicarb^b^ | 52 | 56 | 49 | 50 | 52 | 51 |
| Thiophanate methyl^b^ | 50 | 58 | 53 | 51 | 56 | 52 |
