# Supplementary material for: Pesticides Curbing Soil Fertility: Effect of Complexation of Free Metal Ions
Source: Front Chem. 2017 Jul 4;5:43. doi: 10.3389/fchem.2017.00043 (PMC5495828; doi:10.3389/fchem.2017.00043)
Supplement: Supplementary file 3 [file Table3.docx]

| **Sample** | **Stage** | **T_i_(°C)** | **T_p_(DTG_max_) (°C)** | **T_f_(°C)** | **Mass loss (%)** |
| --- | --- | --- | --- | --- | --- |
| Fe(II)-Cf | 1st | 39 | 126 | 200 | 5 |
|  | 2^nd^ | 201 | 248 | 350 | 14 |
|  | 3^rd^ | 401 | 432 | 450 | 32 |
|  | 4^th^ | 680 | 758 | 790 | 38 |
| Fe(II)-Cz | 1st | 39 | -- | 200 | 5 |
|  | 2^nd^ | 201 | 239, 343 | 400 | 9, 23 |
|  | 3^rd^ | 401 | 475 | 600 | 23 |
|  | 4^th^ | 601 | --- | 850 | 18 |
| Fe(II)-TC | 1st | 39 | 82 | 200 | 7 |
|  | 2^nd^ | 201 | 299 | 400 | 26 |
|  | 3^rd^ | 421 | 478 | 600 | 15 |
|  | 4^th^ | 601 | --- | 800 | 11 |
| Cu(II)-TM | 1st | 39 | ---- | 200 | 2 |
|  | 2^nd^ | 201 | 266, 317 | 400 | 9 |
|  | 3^rd^ | 401 | 498 | 800 | 23 |
|  |  |  |  |  |  |
