# Supplementary material for: Pesticides Curbing Soil Fertility: Effect of Complexation of Free Metal Ions
Source: Front Chem. 2017 Jul 4;5:43. doi: 10.3389/fchem.2017.00043 (PMC5495828; doi:10.3389/fchem.2017.00043)
Supplement: Supplementary file 4 [file Table4.docx]

**S6: Theoretical detail:**

**Supplementary Table 4:** Total %VBur and quadrant %VBur (numbers in brackets) values for species 2* P (P = glyphosate) for the considered metals.

| Complex 2*P | Co^II^ | Cu^II^ | Fe^III^ | Mn^II^ | Ni^II^ | Zn^II^ |
| --- | --- | --- | --- | --- | --- | --- |
| **total %VBur** | 97.9 | 98.2 | 95.5 | 90.5 | 98.4 | 97.1 |
| **quadrant3 %VBur** | 97.1 | 97.9 | 94.7 | 96.1 | 97.8 | 95.6 |
| **quadrant4 %VBur** | 97.6 | 98.1 | 95.0 | 88.3 | 98.7 | 96.7 |
| **quadrant4 %VBur** | 99.2 | 99.3 | 92.7 | 98.1 | 97.7 | 99.1 |
| **quadrant4 %VBur** | 97.6 | 97.7 | 99.6 | 89.5 | 99.2 | 96.9 |
