# Supplementary material for: Pesticides Curbing Soil Fertility: Effect of Complexation of Free Metal Ions
Source: Front Chem. 2017 Jul 4;5:43. doi: 10.3389/fchem.2017.00043 (PMC5495828; doi:10.3389/fchem.2017.00043)
Supplement: Supplementary file 5 [file Table5.docx]

**Supplementary Table 5:** xyz coordinate data sets and absolute energies in a.u. for DFT optimized complexes.

Supporting Information

H2O

3

H2O SCF Done: -76.4414656037 A.U.

O 0.053688 0.053688 0.000000

H 1.007106 -0.060794 0.000000

H -0.060794 1.007106 0.000000

M-4OH2pq-CoIIq

13

M-4OH2pq-CoIIq SCF Done: -1687.97683133 A.U.

Co -5.114537 2.242907 -1.549936

O -4.606422 0.666742 -2.696381

H -4.596159 -0.262159 -2.426147

H -4.315865 0.697317 -3.618722

O -6.517882 2.767784 -2.915622

H -6.622813 3.639934 -3.320116

H -7.223061 2.206510 -3.266524

O -5.637910 3.820633 -0.400868

H -6.503894 3.975731 0.001367

H -5.074763 4.568255 -0.156675

O -3.703208 1.721307 -0.195058

H -3.844310 1.582218 0.751675

H -2.762354 1.576218 -0.365520

M-4OH2pq-CuIId

13

M-4OH2pq-CuIId SCF Done: -1945.66771231 A.U.

Cu -5.117201 2.246045 -1.550652

O -4.599426 0.694959 -2.679266

H -4.948994 -0.203894 -2.601348

H -3.962814 0.712428 -3.407778

O -6.492654 2.727084 -2.837268

H -7.065954 3.503364 -2.781642

H -6.711616 2.232798 -3.638622

O -5.626483 3.797972 -0.419205

H -6.319166 3.802986 0.256484

H -5.234923 4.681896 -0.460950

O -3.746969 1.761244 -0.260111

H -3.576622 2.215289 0.576174

H -3.120356 1.031227 -0.354344

M-4OH2pq-FeIIId

13

M-4OH2pq-FeIIId SCF Done: -1568.17592187 A.U.

Fe -5.117221 2.246363 -1.550654

O -4.623204 0.748522 -2.634336

H -5.216909 0.020739 -2.928137

H -3.728930 0.564545 -3.001728

O -6.539903 2.639108 -2.742129

H -7.135974 3.417358 -2.695268

H -6.729695 2.185043 -3.591427

O -5.612130 3.743410 -0.466307

H -6.506629 3.926392 -0.098947

H -5.018960 4.471162 -0.171368

O -3.693684 1.854504 -0.359918

H -3.503562 2.309015 0.489055

H -3.096377 1.077236 -0.407363

M-4OH2pq-FeIIIq

13

M-4OH2pq-FeIIIq SCF Done: -1568.21820537 A.U.

Fe -5.117183 2.246389 -1.550648

O -4.626221 0.759960 -2.626458

H -5.009744 -0.143429 -2.562663

H -3.948059 0.773155 -3.338717

O -6.459683 2.713560 -2.808424

H -7.068049 3.481441 -2.738737

H -6.662301 2.232610 -3.640492

O -5.608151 3.732821 -0.474829

H -6.286309 3.719601 0.237433

H -5.224661 4.636224 -0.538621

O -3.774611 1.779297 -0.292920

H -3.571992 2.260333 0.539098

H -3.166212 1.011435 -0.362551

M-4OH2pq-NiII

Ni -5.128391 2.269149 -1.535190

O -4.736002 0.795460 -2.683156

H -4.699307 -0.117000 -2.359746

H -4.163292 0.863920 -3.461577

O -6.571295 2.791801 -2.666848

H -6.452366 3.496733 -3.322892

H -7.087242 2.089745 -3.094579

O -5.630580 3.640447 -0.324327

H -6.508570 4.045360 -0.400611

H -4.998468 4.330328 -0.068356

O -3.729331 1.885258 -0.301961

H -3.936299 1.625072 0.608842

H -2.882034 1.487125 -0.548127

M-4OH2pq-NiIIt

13

M-4OH2pq-NiIIt SCF Done: -1813.51327322 A.U.

Ni -5.118870 2.245695 -1.551367

O -4.545333 0.718592 -2.708281

H -4.351879 -0.182231 -2.414664

H -4.486421 0.726334 -3.673369

O -6.560949 2.733303 -2.847056

H -6.779027 3.623947 -3.154230

H -7.104147 2.113046 -3.352207

O -5.599897 3.826948 -0.428063

H -6.491897 4.163406 -0.266710

H -4.981084 4.416496 0.024072

O -3.757909 1.713176 -0.189145

H -3.865205 1.756045 0.770705

H -2.880561 1.348639 -0.368212

M-4OH2td-FeIIId

13

M-4OH2td-FeIIId SCF Done: -1568.17690725 A.U.

Fe -5.004186 2.337980 -1.666220

O -4.715233 0.513279 -2.093281

H -5.424107 -0.143275 -2.277691

H -3.902805 0.003958 -1.873754

O -6.709147 2.481168 -2.462729

H -7.434722 3.094500 -2.210206

H -6.953003 2.088767 -3.331743

O -5.513580 3.160579 -0.020662

H -6.427026 3.198664 0.340986

H -4.914168 3.350499 0.735089

O -3.183628 2.818844 -1.540360

H -2.795433 3.540481 -0.997294

H -2.484871 2.521876 -2.166517

M-4OH2td-FeIIIq

13

M-4OH2td-FeIIIq SCF Done: -1568.21284414 A.U.

Fe -5.037546 2.228057 -1.504319

O -5.249172 0.335466 -1.512910

H -5.147587 -0.266764 -0.742932

H -5.469254 -0.225222 -2.289338

O -6.009355 2.620131 -3.094461

H -5.937097 3.437132 -3.635728

H -6.686336 2.050758 -3.522605

O -5.299951 2.526314 0.358094

H -5.760493 1.928629 0.987818

H -5.012622 3.315543 0.868224

O -3.583271 3.428615 -1.770808

H -2.974178 3.770559 -1.079578

H -3.295048 3.818104 -2.625838

M-4OH2pq-FeIIIs

13

M-4OH2pq-FeIIIs SCF Done: -1568.27574194 A.U.

Fe -5.111560 2.244593 -1.548377

O -5.390939 0.360846 -1.985421

H -5.882396 -0.299742 -1.448329

H -5.094307 -0.107632 -2.797128

O -5.510124 3.316379 -3.134315

H -4.981318 4.067665 -3.484035

H -6.259310 3.191254 -3.758181

O -6.304166 2.766709 -0.091018

H -6.193138 2.564742 0.864612

H -7.117393 3.313712 -0.166456

O -3.262531 2.541151 -0.991703

H -2.909687 3.318700 -0.504538

H -2.506309 1.925018 -1.113638

M-4OH2td-MnIIs

13

M-4OH2td-MnIIs SCF Done: -1456.27646710 A.U.

Mn -5.036074 2.226295 -1.513734

O -4.753350 0.159726 -1.538241

H -5.065537 -0.474347 -0.877589

H -4.283390 -0.362161 -2.203542

O -6.459858 2.833463 -2.909522

H -6.942957 3.671540 -2.924301

H -6.789140 2.325976 -3.664706

O -5.742724 2.833130 0.355547

H -6.635640 2.726265 0.711960

H -5.238939 3.295150 1.040196

O -3.183470 3.086309 -1.929939

H -2.319141 2.807853 -1.596265

H -3.011691 3.838120 -2.514247

13

M-4OH2td-NiIIt SCF Done: -1813.51897738 A.U.

Ni -5.170131 1.976394 -1.222336

O -4.732683 0.191164 -1.966736

H -5.264121 -0.615099 -1.905411

H -3.893218 -0.047290 -2.382842

O -6.459624 2.590951 -2.580448

H -6.970301 3.411413 -2.542193

H -6.602005 2.194875 -3.451170

O -5.649669 3.210887 0.252220

H -6.385319 3.124732 0.874788

H -5.123585 3.973148 0.529166

O -3.407633 2.835208 -1.627719

H -2.574579 2.720974 -1.150275

H -3.229041 3.399964 -2.391426

M-4OH2td-ZnII

13

M-4OH2td-ZnII SCF Done: -2084.58609583 A.U.

Zn -5.035691 2.228163 -1.507162

O -4.792848 0.257382 -1.547306

H -5.115866 -0.368283 -0.884942

H -4.311530 -0.254110 -2.211479

O -6.377542 2.793132 -2.855235

H -6.867396 3.626485 -2.867479

H -6.694853 2.265424 -3.600657

O -5.691202 2.801867 0.278107

H -6.583434 2.690769 0.633141

H -5.174586 3.270999 0.947262

O -3.277898 3.063083 -1.897315

H -2.416563 2.782846 -1.559496

H -3.122502 3.809562 -2.491823

M-5OH2tp-CoIIq

16

M-5OH2pir-CoIIq SCF Done: -1764.47931626 A.U.

Co -4.966768 2.378680 -1.666651

O -6.333235 1.202166 -0.638931

H -7.267027 1.083599 -0.852968

H -6.155862 0.663744 0.142393

O -4.383541 0.727898 -2.899899

H -4.759910 -0.157926 -2.832660

H -3.737501 0.688212 -3.615629

O -5.860729 3.222502 -3.228958

H -6.300604 4.083193 -3.233846

H -5.916618 2.858742 -4.122665

O -5.471166 4.099278 -0.489280

H -6.127366 4.096474 0.218177

H -5.121741 4.998146 -0.523076

O -3.204835 2.289052 -0.747988

H -2.895146 2.881079 -0.049605

H -2.493954 1.662497 -0.938411

M-5OH2tp-CuIId

16

M-5OH2pir-CuIId SCF Done: -2022.16145084 A.U.

Cu -4.855724 2.473833 -1.747296

O -6.289070 1.271060 -0.662572

H -7.215065 1.124365 -0.890213

H -6.124630 0.740253 0.126132

O -4.132218 0.971707 -2.889104

H -4.602666 0.128827 -2.946791

H -3.584225 1.052963 -3.681321

O -6.208274 2.991090 -3.106736

H -6.610588 3.869576 -3.063449

H -6.193496 2.724822 -4.035283

O -5.338499 4.184746 -0.790100

H -5.877506 4.151279 0.012019

H -4.838612 5.011826 -0.770507

O -3.403130 2.033340 -0.466587

H -2.998940 2.652694 0.154968

H -2.723363 1.394955 -0.723160

M-5OH2tp-FeIIIq

16

M-5OH2tpir-FeIIIq SCF Done: -1644.75922811 A.U.

Fe -4.964399 2.193461 -1.397401

O -6.073287 1.079921 -0.243542

H -7.040695 1.161347 -0.141121

H -5.775038 0.304555 0.270541

O -4.267409 0.633706 -2.253174

H -4.620248 -0.273965 -2.171371

H -3.570497 0.628076 -2.938486

O -6.365433 2.613204 -2.801457

H -7.006970 3.348111 -2.787802

H -6.525750 2.126636 -3.631450

O -5.327013 3.652498 -0.207106

H -5.829351 3.566008 0.627654

H -4.804344 4.477115 -0.154638

O -3.512155 3.241598 -2.170219

H -2.556530 3.061704 -2.073519

H -3.617849 4.078873 -2.661008

M-5OH2pir-FeIIIs

16

M-5OH2pir-FeIIIs SCF Done: -1644.81539880 A.U.

Fe -5.006116 2.345266 -1.632682

O -6.311540 1.226617 -0.659720

H -7.250560 1.088055 -0.897300

H -6.144490 0.702376 0.149419

O -4.227242 1.029316 -2.960044

H -4.465760 0.086043 -3.044923

H -3.549001 1.202313 -3.641779

O -6.177861 3.037429 -3.115955

H -6.665475 3.883414 -3.125504

H -6.294730 2.643854 -4.001807

O -5.223338 4.142904 -0.725389

H -5.850591 4.355569 -0.007657

H -4.731815 4.966675 -0.911683

O -3.365088 2.060029 -0.502547

H -3.070695 2.607677 0.250309

H -2.661702 1.399801 -0.652737

M-5OH2tp-MnIId

16

M-5OH2tp-MnIId SCF Done: -1532.64808750 A.U.

Mn -4.871354 2.463296 -1.736731

O -6.188773 1.328234 -0.754772

H -7.085452 1.582411 -0.503571

H -6.036065 0.432805 -0.428123

O -4.202822 0.840922 -2.857960

H -4.727242 0.061388 -3.078588

H -3.473245 0.853898 -3.490647

O -6.085192 3.093185 -3.192493

H -6.653157 3.873128 -3.140785

H -6.283218 2.645283 -4.024986

O -5.440253 4.168996 -0.684333

H -5.814445 4.179836 0.205288

H -5.114292 5.064200 -0.842940

O -3.292257 2.147913 -0.554319

H -3.058177 2.627586 0.250636

H -2.670060 1.414257 -0.645675

M-5OH2tp-MnIIq

16

M-5OH2tp-MnIIq SCF Done: -1532.69340278 A.U.

Mn -4.849623 2.478567 -1.744276

O -6.265522 1.208651 -0.625046

H -7.195185 1.056259 -0.834577

H -6.093504 0.691655 0.171621

O -4.288113 0.979447 -3.012493

H -4.832684 0.208107 -3.215604

H -3.418935 0.819173 -3.401648

O -6.203657 3.039214 -3.135459

H -6.643052 3.899656 -3.159833

H -6.235125 2.685161 -4.034246

O -5.298024 4.174104 -0.699080

H -6.147433 4.347504 -0.273495

H -4.714048 4.908408 -0.470378

O -3.234811 2.163646 -0.580736

H -2.993454 2.660041 0.211790

H -2.582835 1.457743 -0.676536

M-5OH2pir-MnIIs

16

M-5OH2pir-MnIIs SCF Done: -1532.77310666 A.U.

Mn -5.022445 2.347278 -1.644181

O -6.404665 1.130301 -0.574106

H -7.334186 0.972949 -0.784918

H -6.223470 0.616419 0.223652

O -4.260531 0.820283 -3.001219

H -4.545019 -0.102495 -3.021114

H -3.596859 0.902489 -3.697962

O -6.080066 3.194275 -3.283794

H -6.543218 4.041455 -3.296724

H -6.173263 2.822778 -4.170127

O -5.347233 4.213119 -0.565450

H -5.977774 4.346531 0.153832

H -4.918715 5.068602 -0.697155

O -3.194888 2.157464 -0.558325

H -2.886664 2.724101 0.160062

H -2.487007 1.521789 -0.722470

M-5OH2tp-NiIIt

16

M-5OH2pir-NiIIt SCF Done: -1890.02480169 A.U.

Ni -4.897412 2.437725 -1.717278

O -6.242766 1.319372 -0.703551

H -7.157195 1.159291 -0.967174

H -6.064212 0.782748 0.078391

O -4.275747 0.918135 -2.957280

H -4.611290 0.013805 -2.927140

H -3.607100 0.948505 -3.653171

O -6.148215 3.077145 -3.161793

H -6.634537 3.910767 -3.158536

H -6.245785 2.687036 -4.039063

O -5.295505 4.136081 -0.626573

H -5.964661 4.187780 0.066972

H -4.908442 5.016136 -0.714358

O -3.299332 2.102421 -0.536380

H -3.026337 2.646314 0.212581

H -2.617468 1.434077 -0.675646

M-5OH2tp-ZnII

16

M-5OH2pir-ZnII SCF Done: -2161.07936001 A.U.

Zn -5.000843 2.352512 -1.644657

O -6.344781 1.205250 -0.630161

H -7.269240 1.058196 -0.866152

H -6.170972 0.688227 0.166384

O -4.304977 0.862946 -2.939029

H -4.607332 -0.053820 -2.933594

H -3.644115 0.928646 -3.639667

O -6.029522 3.148926 -3.203730

H -6.477170 4.003834 -3.211685

H -6.105859 2.775230 -4.090126

O -5.350533 4.125310 -0.588605

H -5.997432 4.223355 0.121114

H -4.935809 4.989638 -0.702005

O -3.257013 2.172931 -0.614663

H -2.953219 2.750169 0.096457

H -2.547189 1.545985 -0.799877

M-6OH2-CoIIq

19

M-6OH2-CoIIq SCF Done: -1840.97167227 A.U.

Co -5.119389 2.245256 -1.550144

O -6.554286 1.045962 -0.479127

H -7.467769 0.904660 -0.752927

H -6.419120 0.503388 0.305879

O -4.592039 0.637977 -2.704871

H -4.965798 -0.250897 -2.664408

H -3.927061 0.640962 -3.404521

O -6.687568 2.725057 -2.923374

H -7.248905 3.507711 -2.881895

H -6.833234 2.330220 -3.790587

O -5.645537 3.852065 -0.394075

H -6.311352 3.849039 0.304781

H -5.274139 4.741837 -0.436320

O -3.551862 1.764825 -0.176609

H -3.402256 2.164507 0.687705

O -3.684805 3.444459 -2.621573

H -2.770500 3.583535 -2.349356

H -3.820511 3.988756 -3.405288

H -2.994237 0.979369 -0.214676

M-6OH2-CuIId

19

M-6OH2-CuIId SCF Done: -2098.64510103 A.U.

Cu -5.119890 2.244722 -1.549963

O -6.529105 1.025537 -0.447402

H -7.440024 0.911252 -0.743167

H -6.433538 0.472253 0.336150

O -4.558331 0.561199 -2.739935

H -4.956895 -0.317164 -2.731302

H -3.888218 0.553771 -3.433817

O -6.639549 2.630707 -2.764744

H -7.045939 3.507716 -2.762479

H -6.645282 2.306185 -3.674857

O -5.680632 3.929478 -0.361574

H -6.348203 3.937296 0.334760

H -5.281347 4.807504 -0.371393

O -3.600393 1.859608 -0.334733

H -3.595350 2.183770 0.575512

O -3.710304 3.464975 -2.652170

H -2.800590 3.582621 -2.354036

H -3.804306 4.013941 -3.438941

H -3.192468 0.983316 -0.337296

M-6OH2-FeIIId

19

M-6OH2-CuIId SCF Done: -2098.64510103 A.U.

Cu -5.119890 2.244722 -1.549963

O -6.529105 1.025537 -0.447402

H -7.440024 0.911252 -0.743167

H -6.433538 0.472253 0.336150

O -4.558331 0.561199 -2.739935

H -4.956895 -0.317164 -2.731302

H -3.888218 0.553771 -3.433817

O -6.639549 2.630707 -2.764744

H -7.045939 3.507716 -2.762479

H -6.645282 2.306185 -3.674857

O -5.680632 3.929478 -0.361574

H -6.348203 3.937296 0.334760

H -5.281347 4.807504 -0.371393

O -3.600393 1.859608 -0.334733

H -3.595350 2.183770 0.575512

O -3.710304 3.464975 -2.652170

H -2.800590 3.582621 -2.354036

H -3.804306 4.013941 -3.438941

H -3.192468 0.983316 -0.337296

M-6OH2-FeIIIq

19

M-6OH2-FeIIIq SCF Done: -1721.28332551 A.U.

Fe -5.119571 2.245715 -1.550213

O -6.387653 1.156361 -0.592414

H -7.312137 1.054049 -0.886089

H -6.243392 0.619253 0.207401

O -4.592230 0.605682 -2.729730

H -4.977346 -0.287061 -2.704572

H -3.906222 0.577563 -3.418580

O -6.659052 2.651180 -2.807624

H -7.111301 3.513554 -2.822529

H -6.713374 2.284722 -3.708193

O -5.645665 3.883984 -0.368484

H -6.332285 3.911085 0.319802

H -5.262604 4.777609 -0.394308

O -3.579810 1.838776 -0.293639

H -3.523017 2.208506 0.605431

O -3.851825 3.335903 -2.507358

H -2.926175 3.434966 -2.216265

H -3.995958 3.872033 -3.307849

H -3.130748 0.974805 -0.276173

M-6OH2-FeIIIs

19

M-6OH2-FeIIIs SCF Done: -1721.34651113 A.U.

Fe -5.119177 2.245475 -1.550298

O -6.476656 1.084365 -0.536926

H -7.410379 0.942432 -0.771673

H -6.312190 0.558366 0.265208

O -4.602518 0.636057 -2.705787

H -4.970448 -0.263428 -2.653803

H -3.937745 0.620771 -3.416562

O -6.569434 2.755008 -2.910801

H -7.179452 3.511118 -2.853613

H -6.784859 2.291088 -3.738783

O -5.636846 3.853784 -0.393637

H -6.302367 3.867668 0.316455

H -5.269918 4.753756 -0.444464

O -3.668922 1.735897 -0.189607

H -3.454600 2.198951 0.639150

O -3.762112 3.406342 -2.563707

H -2.828605 3.548971 -2.328487

H -3.926305 3.931362 -3.366548

H -3.057832 0.980704 -0.247503

M-6OH2-MnIIs

19

M-6OH2-MnIIs SCF Done: -1609.26520684 A.U.

Mn -5.119501 2.245248 -1.549805

O -6.583294 1.017558 -0.456755

H -7.504563 0.867941 -0.700558

H -6.429002 0.485520 0.333002

O -4.564372 0.526935 -2.791701

H -4.915883 -0.370317 -2.749164

H -3.902970 0.519703 -3.494091

O -6.683864 2.782720 -3.001965

H -7.284983 3.535527 -2.952904

H -6.896305 2.328647 -3.826094

O -5.675190 3.963542 -0.308283

H -6.337595 3.970959 0.393160

H -5.322758 4.860486 -0.349740

O -3.554293 1.707313 -0.098857

H -3.344683 2.157697 0.728013

O -3.655868 3.473297 -2.642842

H -2.735463 3.625467 -2.397371

H -3.809619 4.003286 -3.434079

H -2.950160 0.957157 -0.151351

M-6OH2-NiIIt

19

M-6OH2-NiIIt SCF Done: -1966.52168411 A.U.

Ni -5.119507 2.245306 -1.549999

O -6.494751 1.081387 -0.519075

H -7.414780 0.934741 -0.766282

H -6.321369 0.552965 0.268197

O -4.595095 0.615773 -2.720919

H -4.960446 -0.274490 -2.667494

H -3.932376 0.612997 -3.420930

O -6.587295 2.758980 -2.922900

H -7.185680 3.511967 -2.860000

H -6.792951 2.296921 -3.743411

O -5.644833 3.874990 -0.379863

H -6.305940 3.876922 0.321672

H -5.279719 4.765359 -0.433143

O -3.651263 1.731571 -0.177485

H -3.445035 2.193430 0.642996

O -3.744004 3.408807 -2.581015

H -2.824315 3.556250 -2.333025

H -3.917170 3.937003 -3.368487

H -3.053837 0.977807 -0.240223

M-6OH2-ZnII

19

M-6OH2-ZnII SCF Done: -2237.56971568 A.U.

Zn -5.119515 2.245374 -1.549920

O -6.513013 1.064331 -0.505320

H -7.434832 0.917605 -0.746796

H -6.344281 0.536132 0.283431

O -4.587736 0.594751 -2.736315

H -4.950684 -0.297040 -2.687217

H -3.925699 0.588226 -3.437329

O -6.608039 2.766594 -2.943233

H -7.208899 3.518261 -2.883952

H -6.817135 2.307531 -3.764870

O -5.652602 3.896202 -0.364568

H -6.312855 3.901588 0.338134

H -5.290235 4.788234 -0.413557

O -3.630322 1.723872 -0.157159

H -3.420466 2.182669 0.664424

O -3.725526 3.425945 -2.594671

H -2.804091 3.573434 -2.352212

H -3.893775 3.953719 -3.383808

H -3.030662 0.971259 -0.216451

P3-1lligand+1OH2-CoIId

22

P3-1lligand+1OH2-CoIId SCF Done: -2350.23893538 A.U.

C 0.400108 3.201572 0.432692

H 0.340243 2.814244 1.450706

H -0.199466 2.565273 -0.219497

P 2.101156 3.206242 -0.240828

N -0.092287 4.606916 0.322670

C -1.574361 4.766400 0.325099

H -1.991254 4.852569 1.329685

H -2.039439 3.893170 -0.139585

C -1.905306 5.963257 -0.520945

O -1.067967 6.436184 -1.313062

O -3.098650 6.420303 -0.383265

H 0.278953 5.130945 1.116621

H -3.277909 7.168499 -0.983300

O 2.991932 3.490090 1.037065

O 2.462828 1.839623 -0.929167

O 2.103190 4.300629 -1.321539

H 3.936109 3.671450 0.913267

H 2.859203 1.137475 -0.392889

Co 0.611317 5.466934 -1.326593

O 1.241175 6.291735 -2.977019

H 2.116114 6.086968 -3.329277

H 0.973339 7.158371 -3.304573

P3-1lligand+1OH2-CoIIq

22

P3-1lligand+1OH2-CoIIq SCF Done: -2350.25168874 A.U.

C 0.387974 3.459733 0.519363

H 0.470429 3.181825 1.571540

H -0.441430 2.893193 0.089895

P 1.855032 2.979054 -0.452726

N 0.135892 4.917334 0.324883

C -1.290991 5.275457 0.470265

H -1.379645 6.284145 0.882338

H -1.837071 4.620970 1.153314

C -1.951128 5.285563 -0.882855

O -1.297972 5.285641 -1.939122

O -3.239545 5.316795 -0.845242

H 0.669291 5.425178 1.024087

H -3.627498 5.365839 -1.738694

O 3.063661 3.357806 0.503883

O 1.832892 1.444807 -0.804965

O 1.771973 3.761426 -1.769337

H 3.963885 3.256733 0.159502

H 2.192830 0.818698 -0.160832

Co 0.678214 5.336248 -1.781317

O 1.544259 7.082597 -2.166310

H 1.761430 7.386450 -3.058554

H 1.906545 7.733358 -1.552848

P3-1lligand+1OH2-CuIId

22

P3-1lligand+1OH2-CuIId SCF Done: -2607.95787751 A.U.

C 0.393788 3.186144 0.420377

H 0.333670 2.789162 1.435088

H -0.205680 2.554496 -0.236468

P 2.102685 3.190317 -0.241863

N -0.100835 4.586594 0.327406

C -1.573685 4.769122 0.341110

H -1.975926 4.876069 1.349965

H -2.059422 3.895858 -0.101677

C -1.909711 5.963557 -0.511720

O -1.086883 6.444578 -1.306899

O -3.108594 6.408937 -0.360366

H 0.297957 5.120636 1.100108

H -3.301697 7.155945 -0.956733

O 2.970235 3.477470 1.054262

O 2.462316 1.804791 -0.896106

O 2.130672 4.266229 -1.330397

H 3.911756 3.678345 0.942317

H 2.867852 1.122720 -0.341385

Cu 0.626085 5.476010 -1.376822

O 1.304141 6.383184 -2.972591

H 2.146916 6.154970 -3.382925

H 0.943389 7.163721 -3.408412

P3-1lligand+1OH2-FeIIIq

22

P3-1lligand+1OH2-FeIIIq SCF Done: -2230.61056788 A.U.

C 0.419762 3.183171 0.486061

H 0.415859 2.873643 1.534646

H -0.230192 2.512697 -0.079789

P 2.093066 3.119064 -0.265880

N -0.065507 4.593825 0.337367

C -1.556244 4.771366 0.428934

H -1.888899 4.865046 1.466869

H -2.067953 3.901234 0.004473

C -1.896126 5.972819 -0.397183

O -1.030668 6.394678 -1.226422

O -3.038230 6.488460 -0.246825

H 0.358189 5.151592 1.086830

H -3.245466 7.249062 -0.833020

O 3.077384 3.402625 0.904685

O 2.319406 1.855890 -1.133272

O 1.979473 4.322235 -1.299671

H 4.048347 3.387726 0.820098

H 2.844269 1.083388 -0.856955

Fe 0.611987 5.486763 -1.383488

O 1.181959 6.454469 -2.960874

H 1.709359 7.271337 -2.965193

H 1.129253 6.127759 -3.875126

P3-1lligand+1OH2-FeIIIs

22

P3-1lligand+1OH2-FeIIIs SCF Done: -2230.63252945 A.U.

C 0.402231 3.437533 0.608083

H 0.491213 3.172771 1.664805

H -0.406208 2.839195 0.180636

P 1.910514 2.995433 -0.336432

N 0.085359 4.897376 0.433167

C -1.370344 5.198591 0.565706

H -1.513288 6.149548 1.091177

H -1.906220 4.449027 1.154435

C -1.966466 5.335333 -0.805666

O -1.196602 5.461332 -1.804274

O -3.229275 5.349481 -0.880532

H 0.591857 5.424033 1.146688

H -3.600121 5.482108 -1.779426

O 3.102234 3.211641 0.642173

O 1.780365 1.621749 -1.043787

O 1.872532 4.078797 -1.495634

H 4.024946 2.982884 0.428556

H 2.170205 0.793936 -0.711957

Fe 0.716132 5.484166 -1.646158

O 1.569365 7.075542 -2.430384

H 2.504926 7.135590 -2.697869

H 1.135674 7.892785 -2.737039

P3-1lligand+1OH2-FeIIIo

22

P3-1lligand+1OH2-FeIIIo SCF Done: -2230.45332390 A.U.

C 0.337001 3.553478 0.649658

H 0.527935 3.290253 1.693038

H -0.554736 3.010374 0.328715

P 1.688852 2.991039 -0.440055

N 0.119809 5.031112 0.491040

C -1.329918 5.453673 0.663865

H -1.336301 6.485090 1.025118

H -1.838479 4.844518 1.415903

C -2.019382 5.398422 -0.650422

O -1.276105 5.764506 -1.745923

O -2.455264 4.155722 -1.098089

H 0.677365 5.517846 1.196662

H -3.181003 4.233216 -1.764331

O 3.012297 3.100651 0.368508

O 1.247952 1.607258 -0.981426

O 1.657939 4.096919 -1.593205

H 3.865161 2.700064 0.123166

H 1.820625 0.958444 -1.428011

Fe 0.701839 5.651460 -1.554662

O 1.653267 7.242267 -2.216695

H 2.569912 7.255457 -2.549110

H 1.280264 8.127080 -2.387477

P3-1lligand+1OH2-MnIId

22

P3-1lligand+1OH2-MnIId SCF Done: -2118.43277926 A.U.

C 0.394283 3.203778 0.435639

H 0.325086 2.800343 1.447086

H -0.201890 2.578481 -0.230281

P 2.100709 3.195849 -0.223107

N -0.095848 4.610169 0.337989

C -1.578990 4.750328 0.345417

H -1.997742 4.818428 1.350746

H -2.031179 3.875230 -0.128406

C -1.936285 5.950275 -0.485966

O -1.110167 6.443239 -1.273765

O -3.139328 6.382779 -0.336084

H 0.264108 5.117748 1.148972

H -3.334716 7.133648 -0.926752

O 2.981013 3.482347 1.062797

O 2.465308 1.823311 -0.900062

O 2.118037 4.283291 -1.305473

H 3.929923 3.642526 0.946973

H 2.825280 1.114896 -0.346696

Mn 0.659801 5.554778 -1.356634

O 1.314765 6.379962 -3.074060

H 2.120259 6.077527 -3.512199

H 1.096603 7.249919 -3.429868

P3-1lligand+1OH2-MnIIq

22

P3-1lligand+1OH2-MnIIq SCF Done: -2118.47455832 A.U.

C 0.404472 3.198570 0.483568

H 0.393558 2.861966 1.520893

H -0.231039 2.531920 -0.101278

P 2.068846 3.142996 -0.275686

N -0.087892 4.599986 0.328215

C -1.573507 4.759726 0.342009

H -1.991716 4.815419 1.348080

H -2.032059 3.896831 -0.148156

C -1.905598 5.979789 -0.469756

O -1.089951 6.437982 -1.291359

O -3.080786 6.470333 -0.271222

H 0.291536 5.145178 1.102654

H -3.257924 7.235571 -0.848830

O 3.006132 3.586372 0.925116

O 2.422430 1.706520 -0.809892

O 2.003777 4.092064 -1.478995

H 3.945588 3.739382 0.743026

H 2.825260 1.071369 -0.200550

Mn 0.633345 5.470027 -1.405274

O 1.350055 6.451791 -2.980512

H 2.215373 6.266995 -3.368061

H 0.859129 7.008065 -3.597728

P3-1lligand+1OH2-MnIIs

22

P3-1lligand+1OH2-MnIIs SCF Done: -2118.53598175 A.U.

C 0.341368 3.379291 0.489377

H 0.308483 2.965572 1.499570

H -0.437009 2.884677 -0.096811

P 1.913223 3.004896 -0.360924

N 0.108340 4.850600 0.458833

C -1.315455 5.201744 0.635519

H -1.399174 6.149877 1.173266

H -1.874206 4.471732 1.225338

C -1.966310 5.394711 -0.709222

O -1.305413 5.540952 -1.746040

O -3.257397 5.421488 -0.669313

H 0.636586 5.268107 1.220221

H -3.641218 5.591861 -1.548612

O 2.992275 3.197959 0.786565

O 1.912379 1.531962 -0.924186

O 2.014974 3.973398 -1.536448

H 3.928378 3.144313 0.542816

H 2.167409 0.814330 -0.327048

Mn 0.765711 5.521306 -1.698563

O 1.604076 7.115392 -2.728729

H 2.509986 7.143250 -3.064253

H 1.162024 7.901433 -3.075091

P3-1lligand+1OH2-NiII

22

P3-1lligand+1OH2-NiII SCF Done: -2475.78711892 A.U.

C 0.396690 3.188371 0.409848

H 0.325410 2.814166 1.431994

H -0.194414 2.547091 -0.244728

P 2.103117 3.220173 -0.246784

N -0.097224 4.592892 0.285871

C -1.580854 4.761892 0.302619

H -1.985928 4.828031 1.313611

H -2.052010 3.902736 -0.181378

C -1.882635 5.980235 -0.517083

O -1.021262 6.431487 -1.296128

O -3.057468 6.481574 -0.374677

H 0.291418 5.135533 1.059864

H -3.210349 7.243892 -0.963902

O 2.971321 3.511804 1.045567

O 2.504019 1.865468 -0.935213

O 2.081863 4.316759 -1.321370

H 3.910371 3.726361 0.936754

H 2.935478 1.181190 -0.402682

Ni 0.567249 5.418277 -1.328821

O 1.155319 6.199587 -2.968403

H 2.057036 6.006571 -3.258354

H 0.951883 7.114760 -3.200339

P3-1lligand+1OH2-NiIIt

22

P3-1lligand+1OH2-NiIIt SCF Done: -2475.79033564 A.U.

C 0.377463 3.264016 0.411036

H 0.314805 2.792264 1.393168

H -0.278395 2.721300 -0.271941

P 2.060724 3.173870 -0.301722

N -0.032888 4.697784 0.435650

C -1.499035 4.923720 0.533273

H -1.768343 5.410979 1.472198

H -2.049070 3.980779 0.518543

C -1.969916 5.775442 -0.616464

O -1.206055 6.169462 -1.511248

O -3.230440 6.042939 -0.580725

H 0.409177 5.135365 1.241574

H -3.508665 6.590454 -1.337847

O 2.988946 3.346660 0.972282

O 2.298900 1.792663 -1.020411

O 2.136933 4.289215 -1.347085

H 3.942489 3.453807 0.836351

H 2.628161 1.049409 -0.494566

Ni 0.698703 5.596071 -1.326247

O 1.502120 6.635067 -2.813779

H 2.275672 6.313616 -3.293918

H 1.077745 7.313967 -3.351858

P3-1lligand+1OH2-ZnII

22

P3-1lligand+1OH2-ZnII SCF Done: -2746.84722358 A.U.

C 0.373750 3.242411 0.556305

H 0.362993 2.856143 1.576564

H -0.266769 2.606116 -0.055412

P 2.042228 3.160755 -0.172727

N -0.087485 4.650700 0.458096

C -1.550011 4.846048 0.457045

H -1.949606 5.109172 1.437945

H -2.058465 3.925302 0.160011

C -1.928024 5.906633 -0.547730

O -1.137969 6.338230 -1.399010

O -3.154082 6.293919 -0.448481

H 0.298121 5.161180 1.250297

H -3.384991 6.952481 -1.129019

O 2.984069 3.505307 1.055025

O 2.169320 1.659689 -0.630166

O 2.119875 4.189472 -1.309979

H 3.937820 3.589343 0.917106

H 2.930781 1.370438 -1.151495

Zn 0.706844 5.549098 -1.396334

O 1.421015 6.356878 -3.063728

H 2.271382 6.103489 -3.443895

H 1.068234 7.096042 -3.574152

P3-1lligand+3OH2-CoIIq

28

P3-1lligand+3OH2-CoIIq SCF Done: -2503.22959887 A.U.

C 0.395640 3.215278 0.623992

H 0.369818 2.932970 1.678192

H -0.213251 2.505340 0.061534

P 2.080254 3.170225 -0.074327

N -0.088866 4.583121 0.353333

C -1.541734 4.771365 0.456765

H -1.905892 4.888006 1.479860

H -2.054430 3.901358 0.037849

C -1.910913 5.962953 -0.384745

O -1.151729 6.404479 -1.248021

O -3.088463 6.450594 -0.126860

H 0.365265 5.218925 1.007557

H -3.293786 7.197532 -0.715487

O 2.949484 3.958758 1.005228

O 2.683756 1.706647 -0.069792

O 1.987526 3.850844 -1.430718

H 3.816900 3.585589 1.215546

H 2.699154 1.236118 -0.913799

Co 0.616475 5.358058 -1.628270

O 1.212691 6.297665 -3.338825

H 0.973412 6.073928 -4.244639

H 1.874447 6.996407 -3.385823

O -0.571640 3.941376 -2.806798

H -0.143307 3.197624 -3.245255

O 1.817096 6.794149 -0.484522

H 1.614479 7.722886 -0.325513

H 2.746942 6.681249 -0.256392

H -1.366712 4.125109 -3.319793

P3-1lligand+3OH2-CuIId

28

P3-1lligand+3OH2-CuIId SCF Done: -2760.91374899 A.U.

C 0.415511 3.255438 0.515127

H 0.379046 2.964521 1.566355

H -0.205440 2.570488 -0.064076

P 2.108931 3.188366 -0.171653

N -0.057617 4.635224 0.277071

C -1.512247 4.853014 0.371153

H -1.862890 4.975358 1.397880

H -2.035204 3.989158 -0.046910

C -1.860939 6.049107 -0.474200

O -1.098346 6.476682 -1.345937

O -3.027689 6.555454 -0.217512

H 0.425476 5.282921 0.902851

H -3.228617 7.299624 -0.812234

O 2.975782 3.839400 0.990043

O 2.644274 1.703533 -0.276024

O 2.067147 3.961397 -1.486120

H 3.889084 3.532333 1.080854

H 2.603398 1.280854 -1.144563

Cu 0.583932 5.333024 -1.546011

O 1.203146 6.032348 -3.313277

H 2.118548 5.880331 -3.576812

H 0.939238 6.887585 -3.671519

O -0.743344 3.683598 -2.585919

H -0.225536 3.116284 -3.168783

O 1.726150 6.877703 -0.143290

H 1.527143 7.820487 -0.103023

H 2.667096 6.822223 0.061092

H -1.543421 3.862096 -3.094290

P3-1lligand+3OH2-FeIIIs

28

P3-1lligand+3OH2-FeIIIs SCF Done: -2383.66720717 A.U.

C 0.390430 3.178558 0.646222

H 0.372505 2.883601 1.698066

H -0.235579 2.477146 0.090359

P 2.064773 3.102424 -0.080835

N -0.081317 4.564716 0.414243

C -1.537878 4.780882 0.530265

H -1.875590 4.950226 1.555743

H -2.082583 3.904438 0.165364

C -1.897256 5.943545 -0.352080

O -1.108170 6.322871 -1.249650

O -3.038353 6.471426 -0.128933

H 0.380238 5.175301 1.089850

H -3.266708 7.204652 -0.734395

O 3.154464 3.792741 0.824751

O 2.294788 1.590323 -0.382780

O 1.955697 4.041715 -1.321697

H 3.596367 3.294694 1.532381

H 3.144888 1.246040 -0.705001

Fe 0.629669 5.379106 -1.624492

O 1.206242 6.309506 -3.372810

H 1.038740 6.006403 -4.277140

H 1.756904 7.102323 -3.449568

O -0.455898 4.043555 -2.824440

H -0.080865 3.241513 -3.219125

O 1.715601 6.876863 -0.650730

H 1.445181 7.792535 -0.482808

H 2.667299 6.827579 -0.470172

H -1.280973 4.223866 -3.300313

P3-1lligand+3OH2-MnIIs

28

P3-1lligand+3OH2-MnIIs SCF Done: -2271.51347938 A.U.

C 0.366973 3.176935 0.667083

H 0.327094 2.855990 1.710445

H -0.244651 2.491713 0.076864

P 2.060181 3.105886 -0.005717

N -0.094711 4.557143 0.430448

C -1.543054 4.763641 0.534057

H -1.906956 4.897875 1.555378

H -2.068597 3.891485 0.134923

C -1.932054 5.943601 -0.320966

O -1.206168 6.390069 -1.207588

O -3.113471 6.414179 -0.038272

H 0.362291 5.165169 1.107200

H -3.342416 7.150623 -0.631176

O 2.912261 3.866958 1.107716

O 2.610317 1.619089 0.003456

O 2.038727 3.794785 -1.360907

H 3.799393 3.523385 1.281163

H 2.711915 1.193022 -0.857718

Mn 0.675779 5.371970 -1.694458

O 1.307446 6.373687 -3.518610

H 1.158254 6.086395 -4.425802

H 1.869706 7.153727 -3.576085

O -0.539922 3.993397 -2.953111

H -0.198723 3.183541 -3.349694

O 1.849877 6.914307 -0.593996

H 1.593514 7.820233 -0.385787

H 2.779294 6.838849 -0.347559

H -1.359682 4.190896 -3.421010

P3-1lligand+3OH2-NiIIt

28

P3-1lligand+3OH2-NiIIt SCF Done: -2628.78060816 A.U.

C 0.380566 3.193874 0.519310

H 0.339696 2.837119 1.550180

H -0.230924 2.537054 -0.101097

P 2.082843 3.178631 -0.149239

N -0.086851 4.585676 0.349932

C -1.539071 4.806510 0.440177

H -1.907228 4.902773 1.463292

H -2.065177 3.959339 -0.007504

C -1.889315 6.030929 -0.370339

O -1.143112 6.486441 -1.240765

O -3.052917 6.530097 -0.077878

H 0.376485 5.170724 1.044027

H -3.259238 7.293531 -0.645020

O 2.908874 3.933064 0.987316

O 2.674118 1.710640 -0.170961

O 2.048424 3.911674 -1.483076

H 3.774073 3.565196 1.214913

H 2.748125 1.278371 -1.032163

Ni 0.569029 5.353231 -1.518671

O 1.202584 6.091405 -3.353095

H 2.075053 5.878672 -3.703121

H 0.920730 6.905874 -3.783347

O -0.619354 3.925833 -2.558522

H -0.083387 3.324047 -3.090712

O 1.717300 6.747339 -0.405537

H 1.556677 7.696458 -0.467003

H 2.673708 6.646001 -0.326673

H -1.299097 4.248048 -3.164148

P3-1lligand+3OH2-ZnII

28

P3-1lligand+3OH2-ZnII SCF Done: -2899.82199796 A.U.

C 0.387999 3.191985 0.621662

H 0.362700 2.870400 1.664598

H -0.228204 2.506368 0.037747

P 2.076992 3.139401 -0.068116

N -0.083188 4.572118 0.400419

C -1.531558 4.781471 0.481053

H -1.908124 4.922398 1.496332

H -2.053014 3.910505 0.075263

C -1.899551 5.964571 -0.381341

O -1.154019 6.414149 -1.250557

O -3.084805 6.436239 -0.122754

H 0.369222 5.176361 1.083521

H -3.301497 7.175601 -0.716909

O 2.930038 3.876329 1.059419

O 2.629464 1.655057 -0.099560

O 2.042927 3.854955 -1.410205

H 3.826541 3.546973 1.211539

H 2.731082 1.249799 -0.970754

Zn 0.638731 5.343653 -1.597696

O 1.211834 6.289487 -3.348578

H 1.187718 5.918458 -4.236529

H 1.787310 7.060423 -3.384886

O -0.555264 3.987740 -2.782388

H -0.184037 3.206963 -3.208835

O 1.778493 6.862443 -0.575368

H 1.503791 7.766796 -0.386015

H 2.718413 6.811138 -0.367138

H -1.327379 4.236769 -3.303649

P3-1lligand-CoIId

19

P3-1lligand-CoIId SCF Done: -2273.72532788 A.U.

C 0.387773 3.138302 0.322740

H 0.140375 2.576657 1.224479

H -0.075884 2.650539 -0.535582

P 2.190612 3.273639 0.014737

N -0.091214 4.557428 0.394294

C -1.555302 4.781341 0.176857

H -2.136007 4.667327 1.093599

H -1.933846 4.047642 -0.539656

C -1.705246 6.147910 -0.431686

O -0.724233 6.701051 -0.971425

O -2.878092 6.660640 -0.377555

H 0.149507 4.919727 1.320403

H -2.930749 7.528307 -0.823393

O 2.727806 3.532332 1.470853

O 2.855672 1.952954 -0.500651

O 2.305906 4.453025 -0.978935

H 3.682289 3.490004 1.639157

H 3.024939 1.857174 -1.450427

Co 0.864997 5.656694 -0.911993

P3-1lligand-CoIIq

19

P3-1lligand-CoIIq SCF Done: -2273.74014286 A.U.

C 0.381124 3.165428 0.337166

H 0.150984 2.552829 1.210290

H -0.082721 2.707578 -0.537844

P 2.182501 3.276274 0.023648

N -0.097182 4.568785 0.467908

C -1.542336 4.797933 0.226158

H -2.139945 4.757575 1.138536

H -1.939015 4.027776 -0.440776

C -1.704702 6.125740 -0.466047

O -0.732904 6.689759 -1.003710

O -2.899867 6.591735 -0.478922

H 0.124161 4.890657 1.410706

H -2.971159 7.433210 -0.968599

O 2.755523 3.469920 1.475732

O 2.806279 1.957451 -0.549747

O 2.331313 4.488382 -0.926037

H 3.707896 3.362981 1.625352

H 2.964053 1.891516 -1.503574

Co 1.005304 5.837159 -0.904425

P3-1lligand-CoIIs

19

P3-1lligand-CoIIs SCF Done: -2273.59389665 A.U.

C 0.367551 3.188958 0.500457

H 0.248136 2.680019 1.459210

H -0.160021 2.609764 -0.259468

P 2.117499 3.258804 -0.003702

N -0.139095 4.577704 0.515643

C -1.563774 4.768123 0.138735

H -2.250310 4.438879 0.922231

H -1.760483 4.164687 -0.757318

C -1.737984 6.231831 -0.156467

O -0.568773 6.743519 -0.818881

O -2.864762 6.526357 -0.778120

H 0.025299 4.999058 1.430941

H -3.002688 7.484567 -0.869519

O 2.943544 3.370600 1.327681

O 2.356565 1.959259 -0.837181

O 2.242627 4.560033 -0.870014

H 3.904011 3.503909 1.321649

H 3.231274 1.701372 -1.166375

Co 0.910691 5.825245 -0.963689

P3-1lligand-CuIId

19

P3-1lligand-CuIId SCF Done: -2531.44542982 A.U.

C 0.383007 3.133187 0.324991

H 0.162506 2.530640 1.208265

H -0.085453 2.667959 -0.543272

P 2.186970 3.284667 0.007367

N -0.114690 4.529878 0.460133

C -1.557108 4.792801 0.216953

H -2.146593 4.740618 1.134549

H -1.963111 4.040473 -0.464255

C -1.688979 6.140230 -0.447748

O -0.709922 6.701766 -0.980462

O -2.871430 6.633559 -0.442952

H 0.146686 4.892685 1.378580

H -2.927613 7.486285 -0.916573

O 2.725474 3.504060 1.468985

O 2.843343 1.972509 -0.540934

O 2.327495 4.484376 -0.958939

H 3.681066 3.471051 1.633578

H 3.007866 1.895826 -1.493359

Cu 0.899791 5.690120 -0.909092

P3-1lligandFAC+3OH2-CoIIq

28

P3-1lligandFAC+3OH2-CoIIq SCF Done: -2503.22978672 A.U.

Co 0.436911 5.322668 -1.600168

C 2.052648 5.178865 -4.189239

H 1.339771 4.564073 -4.739720

H 2.770073 5.586472 -4.904598

P 2.854570 4.089775 -2.969795

N 1.299970 6.233810 -3.462277

C 2.118898 7.409634 -3.101349

H 1.568387 8.328661 -3.315181

H 3.048174 7.467165 -3.671752

C 2.422949 7.399544 -1.631177

O 1.828992 6.660356 -0.846183

O 3.330569 8.260925 -1.277464

H 0.547379 6.543609 -4.068148

H 3.469762 8.243970 -0.315222

O 3.589432 2.886923 -3.695433

O 4.042909 5.031249 -2.460011

O 1.809896 3.699523 -1.941339

H 3.172358 2.019061 -3.613209

H 4.793267 4.588452 -2.039565

O 0.066691 4.436005 0.206483

H -0.445546 4.741831 0.962657

O -1.033868 6.901261 -1.259979

H -1.972753 6.947509 -1.470571

O -0.871531 4.136789 -2.720610

H -1.832982 4.173029 -2.785931

H -0.636526 3.200198 -2.724036

H 0.634590 3.718521 0.511628

H -0.882100 7.600778 -0.614739

P3-1lligandFAC+3OH2-CuIId

28

P3-1lligandFAC+3OH2-CuIId SCF Done: -2760.91053245 A.U.

Cu 0.471641 5.394604 -1.604982

C 1.992140 5.138389 -4.139604

H 1.253756 4.545680 -4.680212

H 2.681972 5.567558 -4.869193

P 2.842351 4.011025 -2.979530

N 1.273696 6.191924 -3.373778

C 2.104378 7.368947 -3.019713

H 1.560127 8.288865 -3.245316

H 3.031683 7.405023 -3.593488

C 2.403971 7.368302 -1.551399

O 1.796627 6.642099 -0.756350

O 3.316729 8.216817 -1.195844

H 0.483117 6.502931 -3.930250

H 3.450312 8.209979 -0.231872

O 3.612503 2.905774 -3.816167

O 3.997368 4.982726 -2.438292

O 1.835949 3.534349 -1.961203

H 3.262128 2.006520 -3.758704

H 4.784132 4.553208 -2.074226

O 0.000983 4.463445 0.123867

H -0.214983 4.939187 0.934949

O -1.106357 7.084349 -1.553301

H -1.981588 7.115945 -1.956893

O -0.773895 4.127363 -2.643380

H -1.695039 4.018103 -2.372604

H -0.367534 3.251094 -2.558867

H 0.650021 3.788234 0.364486

H -1.143295 7.748219 -0.855059

P3-1lligandFAC+3OH2-FeIIIs

28

P3-1lligandFAC+3OH2-FeIIIs SCF Done: -2383.66338343 A.U.

Fe 0.478023 5.306465 -1.557879

C 2.135710 5.162635 -4.240468

H 1.433760 4.549691 -4.808414

H 2.863544 5.574356 -4.943491

P 2.925521 4.099884 -2.987059

N 1.377441 6.221969 -3.513659

C 2.195627 7.409594 -3.151851

H 1.711483 8.329831 -3.492418

H 3.179165 7.399378 -3.627044

C 2.354322 7.497503 -1.664053

O 1.673710 6.772799 -0.902620

O 3.194294 8.370691 -1.258093

H 0.626780 6.527908 -4.129122

H 3.261127 8.440300 -0.285598

O 3.547435 2.775547 -3.518041

O 4.125499 4.963303 -2.443140

O 1.794606 3.947980 -1.923673

H 3.039820 1.959168 -3.646822

H 4.947141 4.520606 -2.170411

O 0.197120 4.359687 0.237238

H -0.383605 4.604888 0.972874

O -1.067875 6.658373 -1.117485

H -2.026129 6.517138 -1.145529

O -1.006707 4.297540 -2.636033

H -1.765235 4.613860 -3.148150

H -1.129041 3.340762 -2.538740

H 0.776216 3.650291 0.555288

H -0.936859 7.498513 -0.652536

P3-1lligandFAC+3OH2-MnIIs

28

P3-1lligandFAC+3OH2-MnIIs SCF Done: -2271.51536348 A.U.

Mn 0.395902 5.347920 -1.537025

C 2.171831 5.159720 -4.228981

H 1.472134 4.508303 -4.754586

H 2.913272 5.507917 -4.952734

P 2.945095 4.146569 -2.930340

N 1.409789 6.259486 -3.592218

C 2.244962 7.424277 -3.240960

H 1.767580 8.348063 -3.576001

H 3.224166 7.401914 -3.724970

C 2.434118 7.541401 -1.752637

O 1.773992 6.903173 -0.935313

O 3.342865 8.416804 -1.428088

H 0.713835 6.564562 -4.263088

H 3.413489 8.497775 -0.461845

O 3.632519 2.858223 -3.548180

O 4.167417 5.072770 -2.486912

O 1.897221 3.873777 -1.865367

H 3.151784 2.026573 -3.441655

H 4.896148 4.635569 -2.024714

O 0.047823 4.097931 0.217952

H -0.475199 4.258494 1.010851

O -1.177266 6.757398 -0.906988

H -2.134599 6.655358 -0.948404

O -1.171379 4.260287 -2.667976

H -1.829141 4.511626 -3.325666

H -1.383083 3.352199 -2.420743

H 0.695297 3.421465 0.448558

H -1.017680 7.561104 -0.398899

P3-1lligandFAC+3OH2-NiIIt

28

P3-1lligandFAC+3OH2-NiIIt SCF Done: -2628.78108153 A.U.

Ni 0.478879 5.327491 -1.652375

C 2.098929 5.202276 -4.195415

H 1.421718 4.585127 -4.787200

H 2.831704 5.643925 -4.873808

P 2.875258 4.109448 -2.963276

N 1.296421 6.220219 -3.468689

C 2.068674 7.417704 -3.072239

H 1.463714 8.313137 -3.230912

H 2.976074 7.549295 -3.665050

C 2.412758 7.372122 -1.609430

O 1.865350 6.599495 -0.822464

O 3.307864 8.249730 -1.263136

H 0.544526 6.513439 -4.083571

H 3.472545 8.218525 -0.305129

O 3.587017 2.869808 -3.643005

O 4.076321 5.022809 -2.445775

O 1.794448 3.759084 -1.950517

H 3.127923 2.020953 -3.586444

H 4.809211 4.569815 -2.005245

O -0.033291 4.416586 0.144484

H -0.224209 4.876843 0.969360

O -0.944344 6.897414 -1.444056

H -1.814861 6.937853 -1.856199

O -0.984417 4.175763 -2.616702

H -1.837011 4.059500 -2.178348

H -0.698979 3.278692 -2.830718

H 0.535192 3.674026 0.383529

H -0.984525 7.489582 -0.684596

P3-1lligandFAC+3OH2-ZnII

28

P3-1lligandFAC+3OH2-ZnII SCF Done: -2899.82453386 A.U.

Zn 0.454647 5.344708 -1.598102

C 2.090611 5.169717 -4.184650

H 1.378657 4.531674 -4.709495

H 2.810194 5.547124 -4.914446

P 2.897570 4.124315 -2.931281

N 1.338622 6.248043 -3.497368

C 2.168989 7.411155 -3.131327

H 1.665721 8.338668 -3.414213

H 3.130484 7.420016 -3.648943

C 2.403763 7.472160 -1.644671

O 1.773486 6.796487 -0.834233

O 3.312376 8.344588 -1.315305

H 0.609983 6.561748 -4.128548

H 3.414441 8.390128 -0.349343

O 3.550387 2.842649 -3.595430

O 4.142443 5.031150 -2.513611

O 1.877049 3.843685 -1.841969

H 3.064331 2.013256 -3.494517

H 4.883772 4.580819 -2.085026

O 0.051896 4.287485 0.153561

H -0.389259 4.588587 0.955049

O -1.075782 6.774032 -1.236322

H -2.028995 6.632330 -1.239456

O -0.962844 4.174689 -2.688172

H -1.744174 4.404713 -3.202506

H -1.105853 3.273391 -2.375013

H 0.734597 3.663166 0.427508

H -0.924218 7.560174 -0.699095

P3-1lligand-FeIIIq

19

P3-1lligand-FeIIIq SCF Done: -2154.06002656 A.U.

C 0.393980 3.185826 0.375178

H 0.171763 2.677619 1.320021

H -0.095010 2.644725 -0.438291

P 2.194813 3.249191 0.004403

N -0.108706 4.602824 0.415093

C -1.592339 4.787105 0.181351

H -2.147893 4.677138 1.119472

H -1.963048 4.020729 -0.508170

C -1.770910 6.135275 -0.455369

O -0.736634 6.687086 -0.958828

O -2.924152 6.632134 -0.485976

H 0.127124 5.009214 1.329347

H -3.023622 7.495601 -0.949841

O 2.924270 3.325013 1.367132

O 2.523074 2.133027 -1.005458

O 2.232919 4.659341 -0.767633

H 3.889639 3.305879 1.511954

H 3.313958 1.561879 -1.029611

Fe 0.890080 5.803083 -0.888959

P3-1lligand-FeIIIs

19

P3-1lligand-FeIIIs SCF Done: -2154.07584247 A.U.

C 0.377901 3.224230 0.413902

H 0.197151 2.604356 1.298221

H -0.125487 2.756032 -0.435459

P 2.164967 3.266074 0.004073

N -0.125260 4.618596 0.603741

C -1.573021 4.838274 0.315568

H -2.157144 4.953373 1.235083

H -2.005225 3.984170 -0.216076

C -1.732544 6.061832 -0.546838

O -0.694834 6.629608 -1.018086

O -2.909153 6.453005 -0.765978

H 0.062443 4.904370 1.569424

H -3.010499 7.240496 -1.347036

O 2.948622 3.219764 1.340564

O 2.378571 2.178082 -1.069114

O 2.301252 4.740733 -0.630133

H 3.905790 3.064497 1.448900

H 3.229011 1.804184 -1.366968

Fe 1.066765 6.051012 -0.697972

P3-1lligand-MnIIs

19

P3-1lligand-MnIIs SCF Done: -2042.02223559 A.U.

C 0.382774 3.202820 0.318382

H 0.144423 2.526142 1.140937

H -0.083235 2.809582 -0.587125

P 2.186039 3.278993 0.010042

N -0.084537 4.598334 0.546193

C -1.526783 4.840547 0.312363

H -2.100094 4.909270 1.238721

H -1.973332 4.017497 -0.251726

C -1.718498 6.099832 -0.496895

O -0.771003 6.687970 -1.052114

O -2.939702 6.491128 -0.576341

H 0.124024 4.839751 1.513244

H -3.040088 7.290615 -1.126891

O 2.764978 3.394014 1.470355

O 2.734590 1.947356 -0.615849

O 2.413277 4.521874 -0.879084

H 3.709812 3.233172 1.616156

H 2.982550 1.944728 -1.551914

Mn 1.094110 5.959064 -0.892639

P3-1lligand-NiII

19

P3-1lligand-NiII SCF Done: -2399.26497755 A.U.

C 0.384009 3.129028 0.318049

H 0.123245 2.581772 1.225314

H -0.072275 2.636980 -0.541211

P 2.188210 3.278315 0.030053

N -0.094279 4.549848 0.375171

C -1.563093 4.775401 0.170552

H -2.128313 4.658778 1.097150

H -1.948242 4.043914 -0.544153

C -1.698161 6.142817 -0.431366

O -0.696275 6.682843 -0.947647

O -2.862657 6.672499 -0.398028

H 0.166684 4.939766 1.285381

H -2.897206 7.543402 -0.839905

O 2.713811 3.495501 1.495117

O 2.885662 1.989811 -0.520419

O 2.281614 4.485088 -0.933495

H 3.667819 3.463129 1.669389

H 3.020268 1.904854 -1.477042

Ni 0.828487 5.618943 -0.897094

P3-1lligand-NiIIt

19

P3-1lligand-NiIIt SCF Done: -2399.28303253 A.U.

C 0.339539 3.296555 0.259523

H 0.050218 2.484849 0.929161

H -0.143929 3.132933 -0.705663

P 2.144435 3.347773 -0.037601

N -0.069385 4.637039 0.789280

C -1.501032 4.967379 0.562365

H -1.909457 5.479719 1.437400

H -2.117408 4.078153 0.415468

C -1.626156 5.897462 -0.614685

O -0.629224 6.433030 -1.139251

O -2.829488 6.108071 -1.006792

H 0.122175 4.660153 1.789271

H -2.877945 6.748133 -1.742758

O 2.716767 3.165654 1.415603

O 2.656130 2.147028 -0.905608

O 2.393473 4.731182 -0.689369

H 3.658829 2.968743 1.537137

H 2.864174 2.307315 -1.838602

Ni 1.057587 6.001520 -0.319063

P3-1lligand-ZnII

19

P3-1lligand-ZnII SCF Done: -2670.33527056 A.U.

C 0.370535 3.189693 0.366555

H 0.202930 2.506007 1.199819

H -0.126379 2.777685 -0.513463

P 2.151509 3.300037 -0.046470

N -0.131155 4.567049 0.629666

C -1.549756 4.840483 0.316134

H -2.167326 4.934627 1.210730

H -1.988725 4.025591 -0.265538

C -1.680246 6.103835 -0.509356

O -0.707132 6.691567 -1.027778

O -2.889175 6.507612 -0.647801

H 0.036029 4.788098 1.608386

H -2.959997 7.306816 -1.204381

O 2.848991 3.316540 1.369654

O 2.429017 2.001332 -0.878730

O 2.396224 4.606446 -0.848379

H 3.809909 3.416272 1.442618

H 3.267910 1.878593 -1.347473

Zn 0.986144 5.834404 -0.718376

P3-2lligand180-CoIId

37

P3-2lligand180-CoIId SCF Done: -3165.46738021 A.U.

C 0.362876 3.176731 0.252761

H 0.202738 2.763200 1.249695

H -0.262000 2.633826 -0.457896

P 2.097154 3.015746 -0.304229

N 0.036393 4.620208 0.159569

C -1.407299 4.937779 0.222004

H -1.802138 4.945239 1.239114

H -1.962206 4.177063 -0.333192

C -1.637331 6.252794 -0.474164

O -0.832303 6.710961 -1.281734

O -2.772340 6.814963 -0.158480

H 0.512208 5.112800 0.916869

H -2.900689 7.634453 -0.665256

O 2.876961 3.433448 1.018232

O 2.485864 1.489180 -0.515169

O 2.260123 3.894996 -1.529440

H 3.807954 3.178454 1.066917

H 2.481690 1.187345 -1.433180

Co 0.814839 5.412341 -1.481582

C 2.087366 7.598769 -2.818258

H 2.755808 7.992751 -3.585658

H 1.215992 8.250636 -2.742116

P 2.871196 7.543509 -1.167106

N 1.615772 6.222185 -3.106285

C 0.666553 6.121146 -4.236907

H 1.159412 6.121839 -5.210360

H -0.002155 6.985279 -4.215826

C -0.187275 4.894080 -4.049515

O -0.322885 4.351902 -2.957525

O -0.777940 4.510324 -5.150291

H 2.426473 5.634847 -3.306689

H -1.352966 3.745672 -4.979472

O 4.297945 6.938719 -1.534012

O 3.195853 9.015785 -0.660791

O 1.971894 6.729420 -0.260316

H 5.001104 7.074774 -0.885400

H 2.578852 9.368962 -0.006632

P3-2lligand180-CoIIq

37

P3-2lligand180-CoIIq SCF Done: -3165.48035483 A.U.

C 0.298991 3.094651 0.369965

H 0.150488 2.623404 1.343798

H -0.333993 2.584084 -0.358454

P 2.020740 2.914025 -0.208990

N -0.007766 4.537325 0.335070

C -1.436743 4.874949 0.329919

H -1.884995 4.930992 1.323838

H -1.989192 4.104674 -0.215287

C -1.653762 6.167809 -0.418214

O -0.839016 6.636779 -1.208377

O -2.814917 6.708290 -0.159794

H 0.433834 4.986306 1.134734

H -2.941679 7.511855 -0.691564

O 2.832985 3.308984 1.099998

O 2.381366 1.386805 -0.455732

O 2.212028 3.805691 -1.430423

H 3.763672 3.049517 1.125910

H 2.384865 1.106236 -1.380232

Co 0.923506 5.413189 -1.435610

C 2.179764 7.671566 -2.962483

H 2.867119 8.096742 -3.696836

H 1.296835 8.311329 -2.909420

P 2.913631 7.650372 -1.290470

N 1.746089 6.292224 -3.257392

C 0.763113 6.163013 -4.340963

H 1.207483 6.067095 -5.333441

H 0.133636 7.056460 -4.372281

C -0.157688 4.998638 -4.066638

O -0.293023 4.486994 -2.958565

O -0.817721 4.623234 -5.129810

H 2.563876 5.732407 -3.488040

H -1.431286 3.901638 -4.912545

O 4.373263 7.098050 -1.596307

O 3.150391 9.128908 -0.759092

O 2.029927 6.798848 -0.387019

H 5.036348 7.224416 -0.904634

H 2.501324 9.444624 -0.116942

P3-2lligand180-CuIId

37

P3-2lligand180-CuIId SCF Done: -3423.16922503 A.U.

C 0.359493 3.158652 0.311362

H 0.188659 2.764536 1.314741

H -0.270592 2.608543 -0.389337

P 2.093561 2.942845 -0.228579

N 0.037902 4.600094 0.196697

C -1.403983 4.908812 0.265249

H -1.784323 4.921150 1.288045

H -1.957589 4.133141 -0.269762

C -1.684149 6.214994 -0.436487

O -0.913724 6.713346 -1.244946

O -2.847921 6.713962 -0.105272

H 0.521317 5.108073 0.937979

H -3.023703 7.526681 -0.608157

O 2.867046 3.360958 1.098768

O 2.417427 1.394859 -0.397048

O 2.302805 3.788351 -1.465735

H 3.795929 3.100334 1.156024

H 2.438325 1.079582 -1.310257

Cu 0.872336 5.404566 -1.482065

C 2.127576 7.629699 -2.891060

H 2.800257 8.009850 -3.661924

H 1.260550 8.289308 -2.832198

P 2.909045 7.611361 -1.238301

N 1.651407 6.253758 -3.168518

C 0.684687 6.155924 -4.280371

H 1.164915 6.153317 -5.260260

H 0.023930 7.026033 -4.251428

C -0.186135 4.938290 -4.095335

O -0.318102 4.375505 -3.016533

O -0.798386 4.592842 -5.197968

H 2.456082 5.661586 -3.375101

H -1.385535 3.835137 -5.037710

O 4.350523 7.042719 -1.600609

O 3.185920 9.099236 -0.751222

O 2.030818 6.783805 -0.323304

H 5.038934 7.150130 -0.931044

H 2.558190 9.440149 -0.100658

P3-2lligand180-FeIIIq

37

P3-2lligand180-FeIIIq SCF Done: -3045.92074541 A.U.

C 0.327452 3.083982 0.286691

H 0.198501 2.678364 1.291801

H -0.305941 2.524186 -0.403307

P 2.060355 2.988856 -0.304914

N -0.043749 4.528944 0.215058

C -1.505904 4.825848 0.233859

H -1.915907 4.822211 1.245710

H -2.038388 4.057119 -0.333060

C -1.722373 6.141237 -0.462183

O -0.861038 6.605377 -1.234206

O -2.854805 6.695468 -0.216471

H 0.395027 5.016010 1.002125

H -2.992750 7.525001 -0.711261

O 2.883316 3.472417 0.945176

O 2.554061 1.535490 -0.611445

O 2.081113 3.960604 -1.511583

H 3.774553 3.119932 1.093835

H 2.440075 1.151530 -1.493366

Fe 0.792643 5.434150 -1.392384

C 2.215458 7.633823 -2.831631

H 2.940856 7.934210 -3.590830

H 1.395931 8.354677 -2.829205

P 2.936557 7.570198 -1.147461

N 1.661498 6.278517 -3.097150

C 0.730762 6.213330 -4.251423

H 1.261540 6.178451 -5.205398

H 0.104224 7.109148 -4.265568

C -0.173243 5.023441 -4.073429

O -0.314503 4.500568 -2.960973

O -0.772345 4.658069 -5.154615

H 2.439996 5.637875 -3.272049

H -1.384940 3.912745 -5.016491

O 4.316029 6.823700 -1.295484

O 3.286036 8.991026 -0.587040

O 1.901430 6.732277 -0.376612

H 5.118062 7.263675 -0.972865

H 2.633903 9.509676 -0.094179

P3-2lligand180-FeIIIs

37

P3-2lligand180-FeIIIs SCF Done: -3045.96023800 A.U.

C 0.359410 3.087075 0.578325

H 0.444343 2.761987 1.617658

H -0.372547 2.447830 0.081276

P 1.949788 2.921934 -0.303561

N -0.043630 4.502587 0.432519

C -1.468230 4.783405 0.693516

H -1.682150 5.011508 1.740161

H -2.081330 3.914429 0.439351

C -1.894782 5.916177 -0.193670

O -1.181651 6.290654 -1.141709

O -3.037632 6.426708 0.097567

H 0.515848 5.063074 1.076029

H -3.308313 7.134071 -0.516438

O 3.032216 3.424939 0.720799

O 2.352768 1.444419 -0.638925

O 1.784857 3.857529 -1.529154

H 3.886295 2.967252 0.758789

H 2.020253 1.006938 -1.435984

Fe 0.609477 5.418245 -1.590716

C 2.392142 7.671816 -2.979087

H 3.262736 7.947016 -3.578663

H 1.624157 8.432980 -3.128901

P 2.791285 7.603733 -1.195909

N 1.835611 6.343656 -3.320593

C 1.067703 6.287729 -4.580140

H 1.683346 6.071971 -5.456070

H 0.585631 7.249794 -4.774659

C -0.016493 5.260376 -4.425393

O -0.315395 4.808064 -3.306100

O -0.599485 4.926085 -5.520713

H 2.608511 5.681029 -3.390641

H -1.325084 4.288320 -5.388969

O 4.154885 6.830539 -1.040662

O 3.053695 9.010471 -0.557033

O 1.630725 6.770882 -0.605118

H 4.914813 7.310210 -0.675308

H 2.329722 9.616699 -0.344202

P3-2lligand180-FeIIIo

37

P3-2lligand180-FeIIIo SCF Done: -3045.78370153 A.U.

C 0.300264 3.027092 0.433717

H 0.205616 2.599598 1.434109

H -0.336446 2.456054 -0.245193

P 2.014735 2.918073 -0.187149

N -0.076097 4.460940 0.363547

C -1.527507 4.732936 0.427364

H -1.886540 4.924906 1.440897

H -2.095270 3.871339 0.064581

C -1.843093 5.894306 -0.471939

O -1.011747 6.312992 -1.301544

O -3.021905 6.380303 -0.323240

H 0.371856 4.943833 1.143414

H -3.217847 7.113133 -0.936172

O 2.896105 3.353908 1.039119

O 2.476912 1.470015 -0.561862

O 2.043898 3.945787 -1.351546

H 3.783685 2.976389 1.142382

H 2.320634 1.111111 -1.447650

Fe 0.824698 5.463443 -1.476073

C 2.422986 7.686672 -3.000135

H 3.239560 7.940341 -3.679020

H 1.655756 8.457726 -3.095290

P 2.960826 7.644974 -1.252299

N 1.817433 6.362372 -3.285767

C 0.822866 6.376649 -4.387194

H 1.283363 6.569688 -5.359301

H 0.108556 7.186544 -4.195499

C 0.104289 5.056895 -4.365615

O 0.054338 4.392890 -3.160758

O -1.234259 5.150178 -4.667064

H 2.565182 5.710782 -3.530730

H -1.582998 4.280196 -4.947405

O 4.310247 6.839203 -1.172861

O 3.291455 9.054911 -0.657566

O 1.820686 6.851396 -0.564389

H 5.103968 7.299739 -0.857766

H 2.597291 9.674814 -0.390431

P3-2lligand180-MnIIq

37

P3-2lligand180-MnIIq SCF Done: -2933.68904658 A.U.

C 0.242330 3.082646 0.312083

H 0.058025 2.599407 1.273081

H -0.356527 2.581086 -0.449729

P 1.992061 2.970129 -0.201797

N -0.086341 4.529891 0.299900

C -1.535535 4.845951 0.248249

H -2.026073 4.823979 1.223066

H -2.034372 4.101453 -0.377477

C -1.706262 6.183085 -0.420356

O -0.824770 6.682751 -1.125331

O -2.867601 6.732977 -0.203129

H 0.310496 4.953337 1.138856

H -2.940981 7.577270 -0.679669

O 2.710475 3.420384 1.143448

O 2.437879 1.461850 -0.412247

O 2.181033 3.859162 -1.424728

H 3.663243 3.268323 1.202468

H 2.475055 1.165016 -1.331272

Mn 0.847253 5.448910 -1.310748

C 2.207082 7.606898 -2.911851

H 2.864485 7.944099 -3.715807

H 1.360108 8.292726 -2.849566

P 3.038226 7.638058 -1.287086

N 1.683218 6.235288 -3.115920

C 0.732322 6.148291 -4.241309

H 1.221439 6.135208 -5.217265

H 0.084979 7.029334 -4.225740

C -0.164558 4.947744 -4.073284

O -0.349176 4.400268 -2.996061

O -0.739873 4.599470 -5.198148

H 2.474683 5.626068 -3.326006

H -1.346562 3.855482 -5.048888

O 4.457388 7.010637 -1.648066

O 3.373003 9.139176 -0.877006

O 2.165753 6.877122 -0.311943

H 5.176865 7.198527 -1.030927

H 2.784723 9.520124 -0.212124

P3-2lligand180-MnIIs

37

P3-2lligand180-MnIIs SCF Done: -2933.76048594 A.U.

C 0.129145 2.921347 0.360104

H -0.171948 2.386119 1.263502

H -0.404682 2.490869 -0.489237

P 1.910077 2.736024 0.022947

N -0.131678 4.374255 0.395751

C -1.549499 4.744393 0.311498

H -2.070644 4.730711 1.271206

H -2.074732 4.033398 -0.332018

C -1.683047 6.098875 -0.338235

O -0.787732 6.615617 -0.997072

O -2.859890 6.635023 -0.144529

H 0.241198 4.740422 1.269507

H -2.922613 7.489484 -0.602647

O 2.513279 3.044356 1.463414

O 2.280531 1.217101 -0.259688

O 2.280251 3.696322 -1.093355

H 3.445214 2.829142 1.602101

H 2.455122 1.003278 -1.185431

Mn 1.084082 5.437388 -1.328724

C 2.215992 7.840442 -3.083874

H 2.824037 8.278523 -3.878630

H 1.324853 8.458362 -2.957180

P 3.092718 7.838756 -1.485331

N 1.795799 6.447497 -3.335543

C 0.769977 6.295809 -4.373672

H 1.164480 6.269217 -5.391743

H 0.083413 7.146194 -4.332840

C -0.053587 5.062596 -4.096859

O -0.086972 4.512140 -3.001939

O -0.742751 4.676603 -5.139024

H 2.616581 5.915645 -3.619129

H -1.288735 3.904054 -4.918470

O 4.532724 7.329168 -1.929255

O 3.329395 9.323245 -0.970179

O 2.316288 6.954824 -0.525590

H 5.241815 7.408782 -1.277372

H 2.745033 9.606150 -0.254787

P3-2lligand180-NiIIt

37

P3-2lligand180-NiIIt SCF Done: -3291.03541743 A.U.

C 0.281857 3.155122 0.341642

H 0.106874 2.725135 1.329480

H -0.335557 2.620715 -0.382487

P 2.022394 2.966649 -0.185127

N -0.025429 4.597388 0.252975

C -1.454286 4.942388 0.247516

H -1.901014 4.990631 1.242260

H -2.006421 4.179692 -0.308423

C -1.647608 6.244646 -0.489954

O -0.815855 6.699001 -1.271479

O -2.797566 6.806620 -0.232468

H 0.427203 5.078506 1.028671

H -2.907276 7.617526 -0.756977

O 2.784710 3.359010 1.155337

O 2.384251 1.434197 -0.403657

O 2.244211 3.847204 -1.402266

H 3.715372 3.104587 1.212005

H 2.403376 1.144353 -1.325100

Ni 0.871881 5.414022 -1.465601

C 2.181624 7.623897 -2.924210

H 2.874472 8.017130 -3.670355

H 1.317334 8.287983 -2.872114

P 2.929402 7.577708 -1.256802

N 1.709359 6.252728 -3.208526

C 0.732896 6.132028 -4.301158

H 1.183943 6.086406 -5.294006

H 0.075267 7.005451 -4.293931

C -0.142069 4.927073 -4.056142

O -0.259329 4.399153 -2.953345

O -0.776722 4.536910 -5.128287

H 2.514686 5.665607 -3.419366

H -1.360754 3.786802 -4.926517

O 4.373241 6.999817 -1.593231

O 3.210817 9.048861 -0.724352

O 2.031019 6.744513 -0.359605

H 5.046078 7.092173 -0.905796

H 2.571112 9.380497 -0.080932

P3-2lligand180-ZnII

37

P3-2lligand180-ZnII SCF Done: -3562.07640457 A.U.

C 0.242945 3.064839 0.340113

H 0.013198 2.612892 1.306705

H -0.348125 2.559160 -0.425366

P 2.000371 2.847128 -0.106619

N -0.040544 4.514257 0.274019

C -1.465959 4.861500 0.212230

H -1.956745 4.880420 1.186905

H -1.991767 4.115443 -0.389608

C -1.636696 6.186529 -0.491507

O -0.774830 6.680704 -1.207119

O -2.813272 6.714785 -0.272276

H 0.365889 4.959843 1.095325

H -2.910896 7.545190 -0.767123

O 2.705511 3.181195 1.279108

O 2.330880 1.314831 -0.363687

O 2.302743 3.762624 -1.279708

H 3.638799 2.943052 1.360624

H 2.399795 1.058709 -1.292825

Zn 1.028679 5.392066 -1.403685

C 2.174919 7.712518 -2.948200

H 2.823632 8.136643 -3.716660

H 1.292557 8.347614 -2.855230

P 2.989881 7.688892 -1.314248

N 1.734006 6.330149 -3.233289

C 0.721034 6.210358 -4.289991

H 1.138530 6.181326 -5.297951

H 0.055781 7.076853 -4.252402

C -0.136850 4.992584 -4.041654

O -0.195114 4.421176 -2.959844

O -0.824037 4.649166 -5.099784

H 2.548797 5.781355 -3.502292

H -1.392438 3.885884 -4.904433

O 4.445755 7.186588 -1.710057

O 3.209684 9.167939 -0.777364

O 2.172753 6.798752 -0.395238

H 5.133382 7.271983 -1.036297

H 2.581245 9.457182 -0.102898

P3-2lligand-CoIId

37

P3-2lligand-CoIId SCF Done: -3165.46784445 A.U.

C 0.214387 3.372915 0.073350

H 0.188635 2.850436 1.031749

H -0.534821 2.928474 -0.581780

P 1.817167 3.138599 -0.771621

N -0.078454 4.822309 0.194951

C -1.508737 5.109097 0.462158

H -1.770257 5.017774 1.517124

H -2.117508 4.386516 -0.085947

C -1.853667 6.472549 -0.078880

O -1.175553 7.025574 -0.935561

O -2.952906 6.960208 0.434718

H 0.471225 5.205836 0.963103

H -3.163082 7.818918 0.030595

O 2.935017 2.905675 0.350411

O 1.579957 1.720243 -1.449253

O 2.094285 4.321340 -1.677675

H 3.719880 3.459693 0.254381

H 2.359201 1.158021 -1.551765

Co 0.492822 5.775392 -1.453313

C 1.121708 5.765213 -4.240953

H 1.116009 6.262581 -5.212446

H 2.028605 5.165519 -4.165959

P -0.259044 4.574455 -4.075283

N 1.094383 6.712333 -3.098252

C 2.408082 7.345983 -2.821168

H 2.595380 8.230636 -3.430708

H 3.197229 6.624157 -3.044072

C 2.497929 7.648869 -1.351409

O 1.782197 7.079034 -0.529619

O 3.415397 8.525023 -1.049807

H 0.419371 7.450208 -3.293216

H 3.461067 8.656197 -0.087574

O -1.424464 4.886570 -5.120645

O 0.412435 3.254091 -4.644418

O -0.725241 4.584373 -2.627658

H -2.244818 5.214142 -4.730670

H -0.184883 2.636695 -5.088623

P3-2lligand-CoIIq

37

P3-2lligand-CoIIq SCF Done: -3165.48022887 A.U.

C 0.141191 3.293760 0.150427

H 0.133529 2.682418 1.055784

H -0.605589 2.891040 -0.534934

P 1.736885 3.122406 -0.724597

N -0.147908 4.721583 0.377329

C -1.569855 5.034920 0.588301

H -1.885318 4.980109 1.631509

H -2.184895 4.316896 0.039590

C -1.884567 6.395845 0.015927

O -1.178134 6.957468 -0.818301

O -2.999641 6.891954 0.476903

H 0.374795 5.041917 1.188082

H -3.187901 7.750694 0.061557

O 2.886181 2.782030 0.331710

O 1.492989 1.780670 -1.539411

O 2.029933 4.379911 -1.535418

H 3.639729 3.385371 0.317781

H 2.274415 1.237871 -1.710066

Co 0.508077 5.808063 -1.392794

C 1.207317 5.763021 -4.282723

H 1.227044 6.166271 -5.298016

H 2.102086 5.154711 -4.145546

P -0.193860 4.603091 -4.083324

N 1.165297 6.787350 -3.222949

C 2.455907 7.435300 -2.939743

H 2.647171 8.323870 -3.543786

H 3.268674 6.736023 -3.152362

C 2.550138 7.770659 -1.471147

O 1.841994 7.250032 -0.611874

O 3.483676 8.643868 -1.210920

H 0.495700 7.507000 -3.481519

H 3.539409 8.807137 -0.254045

O -1.353992 4.957567 -5.122388

O 0.450139 3.260110 -4.635211

O -0.672161 4.604139 -2.635966

H -2.197656 5.201884 -4.721345

H -0.161868 2.638690 -5.052251

P3-2lligand-CuIId

37

P3-2lligand-CuIId SCF Done: -3423.16971169 A.U.

C 0.167838 3.333008 0.081735

H 0.115012 2.784154 1.024340

H -0.573777 2.917199 -0.600804

P 1.786062 3.098605 -0.733084

N -0.112034 4.778326 0.240970

C -1.531742 5.074640 0.527758

H -1.778940 4.966276 1.584925

H -2.156851 4.366025 -0.020395

C -1.880122 6.453029 0.023558

O -1.204001 7.041970 -0.807024

O -2.988345 6.909288 0.547889

H 0.456441 5.150079 1.000779

H -3.206233 7.778752 0.171862

O 2.874130 2.831073 0.409857

O 1.546597 1.697090 -1.443674

O 2.091645 4.297602 -1.605461

H 3.666939 3.378183 0.343363

H 2.323032 1.131334 -1.548317

Cu 0.489890 5.787161 -1.431393

C 1.176227 5.743875 -4.246482

H 1.216490 6.217072 -5.229597

H 2.070795 5.133692 -4.120729

P -0.233773 4.582062 -4.120032

N 1.125201 6.722130 -3.135479

C 2.413431 7.404624 -2.883778

H 2.550982 8.288860 -3.507570

H 3.231007 6.719986 -3.120725

C 2.533236 7.745938 -1.420489

O 1.841267 7.217516 -0.559776

O 3.468598 8.627418 -1.186258

H 0.419261 7.427951 -3.337465

H 3.545718 8.796060 -0.232023

O -1.348274 4.947554 -5.204860

O 0.433523 3.253070 -4.676918

O -0.745148 4.589034 -2.691789

H -2.195695 5.226952 -4.835632

H -0.159455 2.648060 -5.143017

P3-2lligandFAC-CoIId

37

P3-2lligandFAC-CoIId SCF Done: -3165.46954245 A.U.

C -0.350746 3.035354 -0.102900

H 0.661733 2.643839 -0.024515

H -0.988046 2.545026 0.635545

P -0.931223 2.740237 -1.813424

N -0.291443 4.511732 0.077909

C -1.617988 5.114462 0.378324

H -1.779801 5.234108 1.450203

H -2.410706 4.449630 0.024280

C -1.789894 6.426572 -0.340550

O -1.060475 6.781795 -1.255816

O -2.817904 7.104696 0.101935

H 0.343630 4.707168 0.848325

H -2.929686 7.921249 -0.413053

O -0.113469 1.547910 -2.497604

O -2.437771 2.251556 -1.595707

O -0.751578 4.023236 -2.598723

H -0.444528 0.658448 -2.310008

H -2.985544 2.182069 -2.390543

Co 0.475989 5.316827 -1.587993

C 1.931001 5.093418 -4.044283

H 1.191943 4.434592 -4.501794

H 2.552625 5.522598 -4.832459

P 2.898734 4.070084 -2.885812

N 1.195962 6.130396 -3.271622

C 2.002807 7.334218 -2.933880

H 1.407778 8.232187 -3.112776

H 2.899168 7.420129 -3.549677

C 2.359268 7.312766 -1.476999

O 1.805641 6.549912 -0.685155

O 3.263483 8.188442 -1.140115

H 0.401832 6.423194 -3.834411

H 3.421690 8.163565 -0.181443

O 3.531050 2.834884 -3.663964

O 4.148164 5.030753 -2.611101

O 2.022329 3.729880 -1.703516

H 3.051406 2.003166 -3.552679

H 4.928845 4.614348 -2.220770

P3-2lligandFAC-CoIIq

37

P3-2lligandFAC-CoIIq SCF Done: -3165.48563023 A.U.

C -0.420113 2.941416 -0.004157

H 0.593003 2.551947 0.089625

H -1.071658 2.386698 0.676072

P -0.935628 2.693138 -1.742904

N -0.382576 4.393994 0.256779

C -1.703737 4.996561 0.506516

H -1.913732 5.143390 1.567508

H -2.498983 4.341104 0.138517

C -1.848746 6.306050 -0.224945

O -1.073704 6.685745 -1.099599

O -2.901023 6.978414 0.151540

H 0.215285 4.553491 1.060870

H -2.982987 7.798496 -0.364122

O -0.053451 1.539514 -2.425376

O -2.434505 2.159490 -1.597492

O -0.754809 3.984270 -2.511798

H -0.349535 0.636470 -2.244845

H -2.949804 2.103312 -2.414690

Co 0.482906 5.358799 -1.545509

C 1.988485 5.180976 -4.135461

H 1.244622 4.532202 -4.600647

H 2.660578 5.544269 -4.917048

P 2.886368 4.148665 -2.933934

N 1.276274 6.260386 -3.418295

C 2.114440 7.425379 -3.083039

H 1.584119 8.350351 -3.319888

H 3.046118 7.457178 -3.652747

C 2.427727 7.463528 -1.610533

O 1.867572 6.752931 -0.779468

O 3.333626 8.353293 -1.309658

H 0.509005 6.565771 -4.005894

H 3.476482 8.378825 -0.348591

O 3.387458 2.801319 -3.612294

O 4.218205 4.995034 -2.706595

O 2.003874 3.942976 -1.712310

H 2.804745 2.041834 -3.474754

H 4.948378 4.537226 -2.267608

P3-2lligandFAC-CuIId

37

P3-2lligandFAC-CuIId SCF Done: -3423.17093646 A.U.

C -0.357436 3.024195 -0.101453

H 0.667542 2.665365 -0.022882

H -0.980413 2.504174 0.629107

P -0.917046 2.705497 -1.816515

N -0.348465 4.494379 0.099904

C -1.678763 5.066594 0.410651

H -1.866780 5.108919 1.484489

H -2.457890 4.425102 -0.010456

C -1.844146 6.430546 -0.214826

O -1.115747 6.853161 -1.098415

O -2.872702 7.071714 0.280232

H 0.300181 4.710924 0.852886

H -2.985678 7.925192 -0.170607

O -0.080068 1.507806 -2.465631

O -2.422467 2.211188 -1.602603

O -0.739668 3.969798 -2.630632

H -0.402148 0.618741 -2.261746

H -2.963165 2.126369 -2.400941

Cu 0.422628 5.359248 -1.609586

C 1.940140 5.075439 -4.048573

H 1.204426 4.401199 -4.488391

H 2.569899 5.471025 -4.848421

P 2.917918 4.083944 -2.865097

N 1.201553 6.151903 -3.338295

C 2.007692 7.354641 -3.020853

H 1.411051 8.250657 -3.204449

H 2.897006 7.432395 -3.648395

C 2.391269 7.363064 -1.566470

O 1.859886 6.629826 -0.738390

O 3.306282 8.249150 -1.285816

H 0.417340 6.425978 -3.922820

H 3.488552 8.259063 -0.331176

O 3.556534 2.856865 -3.654108

O 4.154580 5.071444 -2.621513

O 2.068945 3.735254 -1.669751

H 3.107923 2.013382 -3.508733

H 4.935513 4.680302 -2.206499

P3-2lligandFAC-FeIIId

37

P3-2lligandFAC-FeIIId SCF Done: -3045.93064022 A.U.

C -0.391152 2.910413 -0.110936

H 0.560301 2.377809 -0.077381

H -1.056095 2.477301 0.638279

P -1.034327 2.743786 -1.807215

N -0.125037 4.359311 0.129240

C -1.304215 5.133508 0.610354

H -1.049180 5.699844 1.509635

H -2.140206 4.487488 0.888216

C -1.742390 6.103197 -0.443712

O -1.069167 6.288235 -1.479807

O -2.837072 6.722387 -0.186988

H 0.612254 4.419944 0.828245

H -3.086162 7.368131 -0.874838

O -0.635130 1.397732 -2.509331

O -2.599155 2.912322 -1.622167

O -0.349482 3.881116 -2.602237

H -1.148941 0.592843 -2.338321

H -3.184989 2.805427 -2.388654

Fe 0.564532 5.223846 -1.596687

C 2.044502 5.136116 -4.084271

H 1.341262 4.474303 -4.590713

H 2.684361 5.606247 -4.833201

P 2.981075 4.114102 -2.898127

N 1.258290 6.129202 -3.295110

C 2.020055 7.353337 -2.912789

H 1.568110 8.249676 -3.344392

H 3.045017 7.327863 -3.291052

C 2.051825 7.495513 -1.421848

O 1.471814 6.680672 -0.672955

O 2.711151 8.509531 -0.992717

H 0.459974 6.404499 -3.863374

H 2.715178 8.585535 -0.020106

O 3.061738 2.598025 -3.308883

O 4.398223 4.811095 -2.830362

O 2.157718 4.176799 -1.588869

H 3.736004 2.307794 -3.943069

H 5.103592 4.419492 -2.290625

P3-2lligandFAC-FeIIIq

37

P3-2lligandFAC-FeIIIq SCF Done: -3045.92430172 A.U.

C -0.379007 2.931946 -0.148237

H 0.586268 2.422511 -0.134177

H -1.024962 2.463975 0.596765

P -1.022560 2.778339 -1.845884

N -0.150498 4.381185 0.131467

C -1.312618 5.107451 0.711054

H -0.989346 5.700194 1.569717

H -2.075323 4.419155 1.080844

C -1.910924 6.042701 -0.306976

O -1.350698 6.255152 -1.386826

O -3.019169 6.578569 0.077306

H 0.633868 4.447597 0.778095

H -3.378208 7.208292 -0.574183

O -0.631559 1.431658 -2.549703

O -2.583669 2.964691 -1.666351

O -0.311087 3.905153 -2.637892

H -1.171281 0.637378 -2.410447

H -3.166750 2.892461 -2.439037

Fe 0.524879 5.319530 -1.647567

C 2.113827 5.101297 -4.099245

H 1.384368 4.465213 -4.600520

H 2.779452 5.531185 -4.850838

P 3.018417 4.073103 -2.873790

N 1.374785 6.158483 -3.349998

C 2.222777 7.320636 -2.941776

H 2.037460 8.196877 -3.566417

H 3.283119 7.081339 -3.067134

C 1.988211 7.641721 -1.498083

O 1.298156 6.897346 -0.767101

O 2.563851 8.708507 -1.081439

H 0.614000 6.490170 -3.944023

H 2.419629 8.882554 -0.132020

O 2.910497 2.535770 -3.213584

O 4.499089 4.615851 -2.983317

O 2.299681 4.323426 -1.545959

H 3.521589 2.152277 -3.861354

H 5.198011 4.220749 -2.438143

P3-2lligandFAC-FeIIIs

37

P3-2lligandFAC-FeIIIs SCF Done: -3045.96039698 A.U.

C -0.546399 2.716983 -0.345945

H 0.377397 2.160598 -0.512880

H -1.177421 2.128997 0.324227

P -1.315255 2.950507 -1.983440

N -0.202297 4.049417 0.213825

C -1.300958 4.712950 0.953316

H -0.993629 4.965788 1.971022

H -2.177601 4.069369 1.058510

C -1.700767 5.976906 0.249526

O -1.082436 6.384866 -0.748673

O -2.708406 6.580562 0.771429

H 0.592652 3.919431 0.834801

H -2.934399 7.412175 0.314735

O -1.076459 1.738655 -2.955888

O -2.843008 3.187271 -1.641008

O -0.597477 4.165785 -2.605261

H -1.625664 0.943154 -2.876258

H -3.483815 3.288031 -2.362791

Fe 0.509291 5.379399 -1.531060

C 2.282849 4.873245 -4.024132

H 1.668751 4.058867 -4.412537

H 2.973461 5.180956 -4.812514

P 3.152750 4.211752 -2.564408

N 1.386227 5.973443 -3.585716

C 2.033841 7.303540 -3.505929

H 1.491173 8.040300 -4.103249

H 3.053121 7.292701 -3.899890

C 2.061323 7.772061 -2.081522

O 1.506579 7.123227 -1.178188

O 2.688157 8.878619 -1.894507

H 0.613091 6.013609 -4.245071

H 2.668986 9.174979 -0.965728

O 3.572147 2.705505 -2.721401

O 4.385859 5.192900 -2.397315

O 2.126002 4.268926 -1.414764

H 4.368142 2.477247 -3.226219

H 5.048470 5.011721 -1.711870

P3-2lligandFAC-MnIId

37

P3-2lligandFAC-MnIId SCF Done: -2933.67628809 A.U.

C -0.400821 2.949822 -0.030254

H 0.597463 2.524273 0.056967

H -1.057576 2.454683 0.689138

P -0.971183 2.688669 -1.746907

N -0.299485 4.416607 0.169408

C -1.601249 5.057191 0.472216

H -1.750441 5.232234 1.538876

H -2.425754 4.409716 0.159415

C -1.736939 6.342239 -0.292521

O -0.967690 6.653668 -1.201245

O -2.752841 7.068001 0.082476

H 0.330015 4.575464 0.951085

H -2.822981 7.867279 -0.466259

O -0.140260 1.515262 -2.453446

O -2.475635 2.182029 -1.553971

O -0.792085 3.982741 -2.511275

H -0.457640 0.620335 -2.268620

H -3.012938 2.120958 -2.356301

Mn 0.498318 5.291618 -1.585509

C 1.991472 5.167001 -4.129819

H 1.280464 4.506017 -4.627279

H 2.652245 5.597817 -4.885237

P 2.896607 4.145925 -2.929488

N 1.221539 6.180298 -3.365875

C 1.989716 7.404561 -3.028060

H 1.371427 8.285573 -3.211435

H 2.890053 7.525339 -3.634094

C 2.344081 7.394197 -1.571173

O 1.827948 6.602689 -0.780951

O 3.212553 8.304332 -1.226987

H 0.431779 6.452422 -3.942942

H 3.375469 8.272429 -0.269539

O 3.546902 2.865853 -3.609041

O 4.145373 5.081483 -2.591129

O 1.944926 3.831315 -1.785022

H 3.019886 2.058637 -3.535295

H 4.891560 4.655763 -2.146680

P3-2lligandFAC-MnIIs

37

P3-2lligandFAC-MnIIs SCF Done: -2933.76441585 A.U.

C -0.525254 2.861375 -0.151797

H 0.487621 2.465984 -0.083861

H -1.193272 2.211649 0.420063

P -0.983403 2.850487 -1.923674

N -0.504037 4.258307 0.319339

C -1.832563 4.807325 0.637102

H -2.104649 4.705433 1.689540

H -2.606268 4.270604 0.079286

C -1.921967 6.251263 0.218060

O -1.111126 6.782362 -0.534242

O -2.961403 6.861303 0.717107

H 0.074792 4.291814 1.152456

H -3.002262 7.778312 0.396602

O -0.061564 1.789008 -2.706456

O -2.477497 2.282900 -1.901476

O -0.795586 4.224749 -2.522664

H -0.390164 0.879526 -2.693342

H -2.972259 2.354437 -2.729983

Mn 0.465725 5.595600 -1.395250

C 2.044934 4.974865 -4.025546

H 1.293581 4.265811 -4.376940

H 2.730002 5.183692 -4.851811

P 2.920952 4.149164 -2.659967

N 1.347645 6.178997 -3.524368

C 2.205236 7.370684 -3.413792

H 1.681545 8.247913 -3.799925

H 3.126592 7.288247 -3.994909

C 2.543581 7.655206 -1.974315

O 1.981231 7.104656 -1.032959

O 3.467800 8.566518 -1.839721

H 0.588705 6.375500 -4.167014

H 3.621281 8.754706 -0.898671

O 3.548145 2.776780 -3.163097

O 4.188841 5.102080 -2.474069

O 1.990268 4.027976 -1.466747

H 3.008089 1.995510 -2.986407

H 4.920988 4.743701 -1.953324

P3-2lligandFAC-NiIIt

37

P3-2lligandFAC-NiIIt SCF Done: -3291.04052795 A.U.

C -0.373064 2.973452 -0.039059

H 0.638163 2.580807 0.054059

H -1.024164 2.436072 0.654345

P -0.907196 2.716918 -1.772492

N -0.331186 4.431602 0.208846

C -1.653983 5.033428 0.472533

H -1.834181 5.204890 1.534949

H -2.450716 4.360685 0.142087

C -1.822885 6.321788 -0.289711

O -1.055719 6.686699 -1.175196

O -2.883351 6.987790 0.077892

H 0.279805 4.600561 1.001885

H -2.981784 7.793797 -0.456414

O -0.022089 1.568153 -2.461262

O -2.394748 2.158110 -1.600666

O -0.761288 4.013426 -2.535810

H -0.321242 0.664351 -2.289500

H -2.925343 2.099766 -2.407756

Ni 0.490863 5.361663 -1.540951

C 1.935028 5.157425 -4.093844

H 1.181737 4.510712 -4.546156

H 2.585313 5.535794 -4.885744

P 2.858835 4.123312 -2.912040

N 1.233042 6.230356 -3.352152

C 2.070161 7.402452 -3.025313

H 1.527395 8.322856 -3.250444

H 2.990408 7.438216 -3.612004

C 2.404089 7.430355 -1.557076

O 1.856852 6.706636 -0.730225

O 3.308798 8.323186 -1.260631

H 0.445480 6.532500 -3.915267

H 3.466083 8.341663 -0.301802

O 3.369360 2.789481 -3.609377

O 4.184325 4.985184 -2.701338

O 1.988932 3.903818 -1.687547

H 2.794832 2.023245 -3.474906

H 4.927718 4.533295 -2.278686

P3-2lligandFAC-ZnII

37

P3-2lligandFAC-ZnII SCF Done: -3562.07984367 A.U.

C -0.418983 2.935026 -0.078738

H 0.591732 2.540145 0.012392

H -1.081835 2.356340 0.568646

P -0.915009 2.759455 -1.834311

N -0.385830 4.375039 0.255936

C -1.710320 4.961433 0.528986

H -1.919551 5.055629 1.595726

H -2.501172 4.320833 0.127977

C -1.857767 6.306635 -0.136927

O -1.072128 6.737961 -0.972976

O -2.925436 6.943053 0.260329

H 0.205885 4.490605 1.072677

H -3.009948 7.791360 -0.206540

O -0.013382 1.631762 -2.540742

O -2.406709 2.196838 -1.719326

O -0.745591 4.078823 -2.547980

H -0.333600 0.725967 -2.429009

H -2.922011 2.186266 -2.538209

Zn 0.501658 5.437286 -1.468711

C 1.942372 5.090500 -4.056539

H 1.178127 4.422088 -4.455557

H 2.589941 5.405004 -4.878202

P 2.873271 4.122939 -2.825210

N 1.257107 6.221279 -3.389682

C 2.112053 7.395281 -3.143902

H 1.584417 8.309543 -3.423195

H 3.029820 7.379642 -3.735649

C 2.463088 7.514118 -1.682028

O 1.928066 6.848273 -0.802470

O 3.373788 8.422386 -1.460061

H 0.474089 6.493984 -3.973102

H 3.544737 8.505156 -0.506949

O 3.399592 2.771626 -3.475539

O 4.186856 5.011925 -2.652021

O 2.011058 3.942430 -1.590064

H 2.833311 2.004679 -3.315378

H 4.932583 4.593134 -2.200426

P3-2lligand-FeIIIs

37

P3-2lligand-FeIIIs SCF Done: -3045.96445086 A.U.

C 0.155631 3.215423 0.202483

H 0.109710 2.624507 1.119597

H -0.534444 2.785325 -0.525745

P 1.820992 3.182856 -0.550811

N -0.187617 4.641880 0.402022

C -1.620043 4.919508 0.635348

H -1.927263 4.799255 1.676119

H -2.231349 4.234042 0.041272

C -1.897785 6.311955 0.142249

O -1.106538 6.873785 -0.638230

O -2.989085 6.835213 0.568839

H 0.334662 4.987153 1.208441

H -3.152282 7.727534 0.209276

O 2.779791 3.037440 0.691934

O 1.992745 2.020281 -1.601093

O 1.933062 4.509739 -1.332297

H 3.740091 2.985788 0.567342

H 2.203827 1.128806 -1.282381

Fe 0.485943 5.859187 -1.378808

C 1.219652 5.724703 -4.389142

H 1.280391 6.145260 -5.395135

H 2.065781 5.051018 -4.241983

P -0.302633 4.738713 -4.156667

N 1.230348 6.750713 -3.320394

C 2.540861 7.383009 -3.061936

H 2.766679 8.215700 -3.731016

H 3.339143 6.646219 -3.189858

C 2.557717 7.820153 -1.623910

O 1.738214 7.351827 -0.811598

O 3.470392 8.670681 -1.323295

H 0.571703 7.486965 -3.577879

H 3.469636 8.915138 -0.378791

O -1.338585 5.434486 -5.119002

O -0.092197 3.209643 -4.476570

O -0.621293 4.834912 -2.648391

H -2.243049 5.093245 -5.196513

H -0.133876 2.913588 -5.399213

P3-2lligand-MnIIs

37

P3-2lligand-MnIIs SCF Done: -2933.76489774 A.U.

C 0.142902 3.223919 0.004212

H 0.036899 2.463359 0.782118

H -0.473743 2.939314 -0.850234

P 1.854193 3.315443 -0.617786

N -0.245288 4.582208 0.424523

C -1.692457 4.777898 0.589042

H -2.075891 4.476803 1.566052

H -2.222799 4.179378 -0.156244

C -2.036125 6.218184 0.307600

O -1.294673 6.970615 -0.316956

O -3.204872 6.566771 0.772236

H 0.209160 4.789268 1.310946

H -3.398174 7.490170 0.537384

O 2.705315 3.087930 0.716256

O 2.142421 2.127680 -1.641392

O 2.059782 4.633981 -1.333173

H 3.658635 3.235182 0.647417

H 2.287501 1.255243 -1.251088

Mn 0.458550 6.105556 -1.247512

C 1.267238 5.571189 -4.242015

H 1.461571 5.792949 -5.294560

H 2.014843 4.858851 -3.887436

P -0.350635 4.762661 -4.010995

N 1.295718 6.749155 -3.357203

C 2.637230 7.303754 -3.128263

H 2.970814 7.998970 -3.901152

H 3.363416 6.487106 -3.102438

C 2.678905 7.960245 -1.771985

O 1.861349 7.715891 -0.889935

O 3.679040 8.786434 -1.631178

H 0.713897 7.476450 -3.766833

H 3.685274 9.157972 -0.732519

O -1.260809 5.530950 -5.077583

O -0.264697 3.218750 -4.398644

O -0.767693 4.879088 -2.559923

H -2.211458 5.359494 -5.032430

H -0.256406 3.016838 -5.344041

P3-2lligand-NiIIt

37

P3-2lligand-NiIIt SCF Done: -3291.03691684 A.U.

C 0.180678 3.321327 0.146709

H 0.181875 2.746228 1.074990

H -0.579042 2.901379 -0.513340

P 1.762205 3.120255 -0.750335

N -0.104528 4.758576 0.332548

C -1.525476 5.075018 0.565750

H -1.813698 5.041328 1.617322

H -2.147759 4.342302 0.045983

C -1.848621 6.421993 -0.032139

O -1.142783 6.963313 -0.878066

O -2.964226 6.923605 0.423080

H 0.437026 5.108588 1.119236

H -3.158408 7.772891 -0.008221

O 2.932911 2.767721 0.279084

O 1.490331 1.765430 -1.533574

O 2.043928 4.371432 -1.568305

H 3.659351 3.403531 0.294312

H 2.265118 1.212089 -1.701482

Ni 0.513032 5.785960 -1.400912

C 1.161298 5.763567 -4.262699

H 1.154375 6.202513 -5.262691

H 2.072674 5.174287 -4.158170

P -0.212070 4.567921 -4.065605

N 1.117707 6.758616 -3.172384

C 2.400231 7.436181 -2.907843

H 2.547571 8.339498 -3.501690

H 3.223477 6.761662 -3.155788

C 2.517494 7.742422 -1.435577

O 1.831431 7.188846 -0.581217

O 3.440857 8.626768 -1.173521

H 0.419407 7.465147 -3.390555

H 3.512731 8.771236 -0.214925

O -1.377854 4.870510 -5.115098

O 0.468154 3.242911 -4.618006

O -0.690382 4.581401 -2.621834

H -2.211049 5.160105 -4.722632

H -0.129032 2.609094 -5.038140

P3-2lligand-ZnII

37

P3-2lligand-ZnII SCF Done: -3562.07756517 A.U.

C 0.132543 3.268519 0.102036

H 0.069565 2.632554 0.987345

H -0.576238 2.896351 -0.639235

P 1.773887 3.141690 -0.678883

N -0.161504 4.690905 0.360026

C -1.583345 4.987117 0.590193

H -1.886103 4.897591 1.634635

H -2.197930 4.281671 0.025736

C -1.907738 6.365200 0.067045

O -1.187457 6.968928 -0.720193

O -3.041833 6.821549 0.524259

H 0.364492 4.992753 1.176654

H -3.235148 7.695382 0.144762

O 2.847382 2.911148 0.484989

O 1.646339 1.764158 -1.459123

O 2.039107 4.388883 -1.504097

H 3.635626 3.465152 0.422040

H 2.455830 1.246043 -1.558161

Zn 0.500773 5.854463 -1.370943

C 1.198690 5.736948 -4.289866

H 1.245730 6.137729 -5.304637

H 2.073100 5.103990 -4.134647

P -0.243289 4.631331 -4.095921

N 1.175401 6.771594 -3.237781

C 2.473581 7.416600 -2.987747

H 2.657621 8.290436 -3.614611

H 3.278285 6.707865 -3.197855

C 2.586618 7.786146 -1.528858

O 1.862420 7.313369 -0.659796

O 3.550245 8.635697 -1.298878

H 0.505623 7.491916 -3.496231

H 3.614584 8.828267 -0.348218

O -1.377586 5.023887 -5.151516

O 0.350521 3.261852 -4.638821

O -0.718137 4.678387 -2.654318

H -2.213407 5.307396 -4.760313

H -0.279316 2.672182 -5.074804

P3-lligand1

18

P3-lligand1 SCF Done: -891.562664720 A.U.

C 0.446750 3.288786 0.014047

H 0.093838 2.724543 0.882137

H 0.041755 2.771444 -0.872579

P 2.246115 3.060323 -0.039296

N 0.069256 4.675506 0.136676

C -1.357389 4.876863 0.171592

H -1.767875 4.620044 1.152313

H -1.915876 4.258396 -0.555686

C -1.687468 6.305049 -0.152706

O -0.944938 7.074470 -0.704352

O -2.947161 6.615243 0.215164

H 0.475405 5.238185 -0.603888

H -3.097682 7.528588 -0.070167

O 2.593374 4.181899 -1.160436

O 2.868405 3.745263 1.304270

O 2.709687 1.663146 -0.251367

H 3.542202 4.298663 -1.278989

H 3.232973 3.047321 1.860667

P3-lligand2

18

P3-lligand2 SCF Done: -891.564305849 A.U.

C 0.468688 3.245065 -0.067529

H 0.076991 2.660345 0.768215

H 0.105816 2.774312 -0.994730

P 2.280179 3.142923 -0.019158

N 0.110747 4.638870 0.095435

C -1.312725 4.858316 0.178977

H -1.696536 4.558842 1.158106

H -1.897678 4.280814 -0.559600

C -1.632144 6.304993 -0.063679

O -0.884194 7.099694 -0.569464

O -2.889862 6.601146 0.322840

H 0.496733 5.205575 -0.654338

H -3.034944 7.530476 0.091530

O 2.698434 3.416285 1.531121

O 2.490985 1.541107 -0.115232

O 2.982877 4.005298 -1.007755

H 2.829460 4.363247 1.659970

H 3.408512 1.325389 -0.315778

P1-1LIGAND2222+1H2O-CoIIq

34

P1-1LIGAND2222+1H2O-CoIIq SCF Done: -3422.20049850 A.U.

P -2.001456 1.529659 0.422317

O -1.386163 2.628916 1.352223

O -1.085061 0.259429 0.521986

C -1.428226 -1.120817 0.070617

H -2.495439 -1.263240 0.253002

H -1.231242 -1.143437 -1.002603

C 0.098594 2.790618 1.564905

H 0.561817 2.810779 0.575781

H 0.422785 1.896366 2.093989

C 0.295838 4.048812 2.338418

H -0.212090 4.010656 3.299361

H 1.361782 4.171369 2.528787

H -0.045837 4.924096 1.788736

C -0.572599 -2.073411 0.837202

H -0.775775 -2.023693 1.904528

H -0.792620 -3.085927 0.500684

H 0.484835 -1.886985 0.664139

S -3.966419 1.301641 0.779596

S -1.807562 2.225445 -1.627442

C -2.357737 3.979289 -1.378917

H -1.805019 4.593544 -2.088685

H -2.111653 4.295357 -0.365671

S -4.156687 4.189379 -1.637483

C -4.222481 4.429090 -3.482783

H -5.278104 4.286601 -3.714801

H -3.659542 3.612963 -3.940820

C -3.727242 5.792308 -3.889159

H -3.824144 5.888988 -4.970267

H -4.308376 6.588564 -3.429459

H -2.675698 5.947016 -3.649709

Co -4.296440 1.833085 -1.386889

O -4.883095 0.634901 -2.874837

H -4.321216 0.195681 -3.524923

H -5.775770 0.283433 -2.990237

P1-1LIGAND2222+1H2O-CoIIq

34

P1-1LIGAND2222+1H2O-CoIIq SCF Done: -3422.20049850 A.U.

P -2.001456 1.529659 0.422317

O -1.386163 2.628916 1.352223

O -1.085061 0.259429 0.521986

C -1.428226 -1.120817 0.070617

H -2.495439 -1.263240 0.253002

H -1.231242 -1.143437 -1.002603

C 0.098594 2.790618 1.564905

H 0.561817 2.810779 0.575781

H 0.422785 1.896366 2.093989

C 0.295838 4.048812 2.338418

H -0.212090 4.010656 3.299361

H 1.361782 4.171369 2.528787

H -0.045837 4.924096 1.788736

C -0.572599 -2.073411 0.837202

H -0.775775 -2.023693 1.904528

H -0.792620 -3.085927 0.500684

H 0.484835 -1.886985 0.664139

S -3.966419 1.301641 0.779596

S -1.807562 2.225445 -1.627442

C -2.357737 3.979289 -1.378917

H -1.805019 4.593544 -2.088685

H -2.111653 4.295357 -0.365671

S -4.156687 4.189379 -1.637483

C -4.222481 4.429090 -3.482783

H -5.278104 4.286601 -3.714801

H -3.659542 3.612963 -3.940820

C -3.727242 5.792308 -3.889159

H -3.824144 5.888988 -4.970267

H -4.308376 6.588564 -3.429459

H -2.675698 5.947016 -3.649709

Co -4.296440 1.833085 -1.386889

O -4.883095 0.634901 -2.874837

H -4.321216 0.195681 -3.524923

H -5.775770 0.283433 -2.990237

P1-1LIGAND2222+1H2O-CuIId

34

P1-1LIGAND2222+1H2O-CuIId SCF Done: -3679.90361236 A.U.

P -1.832104 1.900046 0.494701

O -1.296143 2.731131 1.708671

O -0.999139 0.567656 0.448623

C -1.287723 -0.626837 -0.387932

H -2.278339 -0.979585 -0.094533

H -1.311192 -0.296637 -1.428804

C 0.171349 2.876886 2.010357

H 0.645499 3.242667 1.096983

H 0.535307 1.873810 2.229056

C 0.294109 3.818934 3.158767

H -0.221264 3.442797 4.039676

H 1.350048 3.920580 3.407667

H -0.089148 4.807637 2.915802

C -0.211913 -1.627941 -0.123648

H -0.195275 -1.925245 0.922180

H -0.406237 -2.516811 -0.722337

H 0.767182 -1.244205 -0.401753

S -3.837955 1.736649 0.860579

S -1.439183 2.840683 -1.348270

C -2.612990 4.228180 -1.202445

H -2.170046 5.071750 -1.732090

H -2.767835 4.522667 -0.165121

S -4.278795 3.965076 -1.926794

C -3.869503 3.576848 -3.686363

H -4.840177 3.334593 -4.118689

H -3.253377 2.675091 -3.693404

C -3.203689 4.740013 -4.383271

H -3.104082 4.490333 -5.439553

H -3.790860 5.653663 -4.314448

H -2.199918 4.933843 -4.008235

Cu -4.827741 1.865537 -1.085346

O -6.164052 1.187299 -2.387132

H -6.168392 0.282652 -2.724787

H -7.084466 1.480715 -2.376524

P1-1LIGAND2222+1H2O-FeIIIs

34

P1-1LIGAND2222+1H2O-FeIIIs SCF Done: -3302.60765608 A.U.

P -1.750877 1.228186 -0.029086

O -1.253715 2.558112 0.581005

O -0.779657 0.061243 0.202034

C -0.911050 -1.498510 0.300485

H -1.937980 -1.675048 0.618733

H -0.754619 -1.818377 -0.727294

C -0.406571 2.728580 1.921729

H 0.323467 1.923619 1.873475

H -1.146026 2.554913 2.700450

C 0.160278 4.091957 1.891061

H -0.605626 4.864366 1.887019

H 0.726642 4.210937 2.819658

H 0.861446 4.237613 1.072045

C 0.124865 -1.954936 1.254724

H -0.045829 -1.590664 2.265226

H 0.060863 -3.045910 1.286078

H 1.129749 -1.701587 0.924760

S -3.738562 0.779071 0.343378

S -1.823516 1.722118 -2.201034

C -2.033795 3.558993 -2.136543

H -1.766264 3.929815 -3.126832

H -1.366771 3.967622 -1.378952

S -3.781033 3.984862 -1.737667

C -4.402352 4.828257 -3.315869

H -5.479818 4.849521 -3.146831

H -4.175521 4.157542 -4.145315

C -3.794475 6.196156 -3.446356

H -4.238063 6.649717 -4.337810

H -4.027740 6.842002 -2.602977

H -2.717619 6.178229 -3.607688

Fe -4.218974 1.603984 -1.712689

O -5.775170 0.885322 -2.734000

H -5.800202 0.604485 -3.663478

H -6.633523 0.638287 -2.349856

P1-1LIGAND2222+1H2O-MnIIs

34

P1-1LIGAND2222+1H2O-MnIIs SCF Done: -3190.47092485 A.U.

P -2.008528 1.378050 0.390748

O -1.464351 2.550093 1.281208

O -0.975327 0.202932 0.510613

C -1.187024 -1.218925 0.109410

H -2.208081 -1.483695 0.388932

H -1.083865 -1.247517 -0.976469

C 0.005657 2.797582 1.491756

H 0.474081 2.811974 0.504655

H 0.378454 1.939959 2.048555

C 0.131091 4.088186 2.227219

H -0.379312 4.051409 3.186934

H 1.187300 4.276175 2.417469

H -0.256343 4.926768 1.650801

C -0.157928 -2.040022 0.812312

H -0.271020 -1.983415 1.892528

H -0.283990 -3.080929 0.516508

H 0.850325 -1.735794 0.540631

S -3.922514 0.975477 0.768039

S -1.841208 2.069969 -1.684659

C -2.298961 3.852234 -1.435378

H -1.706684 4.427687 -2.145064

H -2.030486 4.146478 -0.422064

S -4.074382 4.215617 -1.693218

C -4.103614 4.577162 -3.521071

H -5.166910 4.554597 -3.760457

H -3.619249 3.745331 -4.036536

C -3.481291 5.909693 -3.842893

H -3.564106 6.084419 -4.915393

H -3.985241 6.726132 -3.330734

H -2.420462 5.948715 -3.597866

Mn -4.417502 1.746953 -1.451400

O -5.764564 0.648313 -2.602064

H -6.184959 -0.174522 -2.319434

H -6.147050 0.863390 -3.462037

P1-1LIGAND2222+1H2O-NiII

34

P1-1LIGAND2222+1H2O-NiII SCF Done: -3818.79595290 A.U.

P -1.823185 1.317295 0.279616

O -1.957826 2.333054 1.477699

O -0.721708 0.266528 0.649080

C -0.538496 -1.087946 0.054409

H 0.255418 -1.500703 0.670730

H -1.457818 -1.642271 0.245667

C -0.752459 2.859288 2.206964

H -0.019929 3.159753 1.453859

H -0.354852 2.018180 2.772332

C -1.212539 3.986816 3.068047

H -1.968574 3.662031 3.779397

H -0.360992 4.360999 3.635177

H -1.607591 4.815481 2.481811

C -0.181835 -1.008735 -1.394934

H 0.702169 -0.393368 -1.557201

H 0.041829 -2.009398 -1.762338

H -1.006896 -0.624324 -1.998587

S -3.630600 0.424295 -0.013010

S -1.064685 2.390700 -1.382418

C -1.999174 3.941510 -1.212017

H -1.401263 4.711949 -1.696618

H -2.131678 4.218744 -0.167144

S -3.681616 4.041848 -1.985116

C -3.330943 3.486699 -3.731547

H -4.320767 3.460508 -4.187509

H -2.939554 2.469705 -3.679362

C -2.398992 4.438158 -4.431579

H -2.311533 4.128038 -5.472711

H -2.768725 5.461786 -4.423926

H -1.393171 4.421754 -4.013663

Zn -4.623383 2.158991 -1.002861

O -6.625785 2.097930 -1.073484

H -7.162266 1.379930 -0.712944

H -7.228625 2.805249 -1.334235

P1-1LIGAND2222+1H2O-NiIIt

34

P1-1LIGAND2222+1H2O-NiIIt SCF Done: -3547.74389207 A.U.

P -1.935394 1.536861 0.441204

O -1.270949 2.667714 1.294311

O -1.001216 0.279485 0.529955

C -1.373835 -1.129445 0.198165

H -2.415262 -1.263294 0.495985

H -1.286792 -1.214611 -0.886106

C 0.214827 2.764236 1.551499

H 0.700336 2.798972 0.574303

H 0.492042 1.841302 2.057794

C 0.437304 3.988262 2.370821

H -0.092535 3.936694 3.319208

H 1.502550 4.065406 2.587567

H 0.139536 4.892252 1.843209

C -0.432818 -2.028376 0.927839

H -0.528289 -1.918635 2.005621

H -0.673612 -3.060069 0.674268

H 0.599499 -1.846161 0.637876

S -3.882470 1.329289 0.812063

S -1.855134 2.179048 -1.654110

C -2.475211 3.914078 -1.402567

H -1.950222 4.556395 -2.108223

H -2.248222 4.231517 -0.385596

S -4.274173 4.007350 -1.677549

C -4.323600 4.327867 -3.503031

H -5.357817 4.110478 -3.769597

H -3.689069 3.581156 -3.983850

C -3.936913 5.746452 -3.834821

H -4.017489 5.886802 -4.912324

H -4.592472 6.469528 -3.354528

H -2.907964 5.977171 -3.560795

Ni -4.209607 1.676846 -1.415775

O -4.941308 0.977998 -3.183701

H -4.515079 0.302923 -3.725312

H -5.876683 0.964985 -3.422224

P1-1LIGAND2222+1H2O-ZnII

34

P1-1LIGAND2222+1H2O-ZnII SCF Done: -3818.79592516 A.U.

P -1.789657 1.319569 0.273690

O -1.916722 2.337498 1.470881

O -0.672418 0.280791 0.629207

C -0.486628 -1.073508 0.034568

H 0.319298 -1.478612 0.640243

H -1.398717 -1.634667 0.239986

C -0.705742 2.871642 2.185300

H 0.015916 3.175502 1.423111

H -0.296721 2.033342 2.746713

C -1.162000 3.997325 3.050758

H -1.908190 3.669068 3.770842

H -0.306022 4.376244 3.608023

H -1.567832 4.823638 2.468600

C -0.151467 -0.994325 -1.419846

H 0.726058 -0.373573 -1.595923

H 0.073272 -1.994188 -1.788820

H -0.987631 -0.616256 -2.012107

S -3.591512 0.406725 0.009600

S -1.067429 2.397049 -1.402647

C -2.010316 3.941087 -1.218411

H -1.428514 4.713999 -1.718311

H -2.122488 4.220963 -0.171913

S -3.710730 4.025372 -1.954788

C -3.395543 3.461146 -3.704792

H -4.397586 3.382641 -4.126754

H -2.957347 2.463444 -3.649113

C -2.532519 4.442066 -4.451013

H -2.462176 4.115929 -5.488548

H -2.951758 5.446441 -4.450322

H -1.514942 4.483109 -4.065235

Zn -4.618437 2.138343 -0.949479

O -6.621499 2.069146 -0.988286

H -7.149054 1.344656 -0.627529

H -7.230988 2.778866 -1.226100

P1-1LIGAND2222+2H2Opq-CoIIq

37

P1-1LIGAND2222+2H2Opq-CoIIq SCF Done: -3498.68934146 A.U.

P -1.921708 1.435239 -0.299769

O -2.342001 2.389886 0.917587

O -0.750984 0.530289 0.224688

C -0.195699 -0.649372 -0.512341

H -1.038792 -1.302227 -0.742293

H 0.232571 -0.267940 -1.440620

C -1.290827 3.046937 1.758996

H -0.564338 3.502564 1.082728

H -0.803915 2.250620 2.318969

C -1.982243 4.042639 2.631259

H -2.707520 3.562971 3.288271

H -1.247523 4.530528 3.270234

H -2.473341 4.823460 2.049485

C 0.816570 -1.293422 0.373497

H 0.369574 -1.646704 1.300025

H 1.232249 -2.155094 -0.147751

H 1.635730 -0.616278 0.604779

S -3.553577 0.415199 -0.880740

S -1.051632 2.653341 -1.787201

C -2.244668 4.029332 -1.836646

H -1.732701 4.814635 -2.392287

H -2.455698 4.407950 -0.836929

S -3.884532 3.783724 -2.646858

C -3.427901 2.836730 -4.170739

H -4.393090 2.612878 -4.625814

H -2.970864 1.900928 -3.844952

C -2.538247 3.639616 -5.083513

H -2.375895 3.072934 -5.999881

H -2.983514 4.593901 -5.358515

H -1.556797 3.823030 -4.647721

Co -4.848117 2.282249 -1.124616

O -6.791697 1.852603 -1.567337

H -7.168439 1.711391 -2.443591

H -7.396520 1.434306 -0.942413

O -5.130743 3.092780 0.705351

H -4.421063 2.974710 1.353280

H -5.807581 3.667520 1.079987

P1-1LIGAND2222+2H2Opq-CuIId

37

P1-1LIGAND2222+2H2Opq-CuIId SCF Done: -3756.38808137 A.U.

P -1.808388 2.046237 0.365359

O -1.366945 2.760557 1.691110

O -0.898116 0.761595 0.245550

C -1.011463 -0.257406 -0.819282

H -2.071707 -0.505865 -0.920855

H -0.663959 0.204693 -1.745458

C 0.067909 2.872082 2.110508

H 0.609470 3.327067 1.278385

H 0.426020 1.853990 2.257522

C 0.101310 3.696618 3.353001

H -0.471759 3.233647 4.153190

H 1.135512 3.777210 3.686007

H -0.275329 4.702565 3.181396

C -0.179594 -1.430573 -0.413275

H -0.534250 -1.867485 0.517454

H -0.236852 -2.191986 -1.189642

H 0.865139 -1.150816 -0.296440

S -3.798353 1.755807 0.616233

S -1.339118 3.170133 -1.345825

C -2.684325 4.390608 -1.251904

H -2.303341 5.310278 -1.695159

H -2.973619 4.601703 -0.223616

S -4.220033 3.966629 -2.155362

C -3.566747 3.482978 -3.813127

H -4.460454 3.172543 -4.353231

H -2.929194 2.608545 -3.670541

C -2.844017 4.619761 -4.493680

H -2.581328 4.306346 -5.503691

H -3.460808 5.512774 -4.574698

H -1.912795 4.880731 -3.992445

Cu -5.074311 1.978740 -1.268464

O -6.153409 0.316250 -0.648239

H -5.747434 -0.291946 -0.016835

H -7.087281 0.365371 -0.406998

O -6.434026 1.981482 -2.834677

H -7.083615 2.669466 -3.024236

H -6.798262 1.153528 -3.171430

P1-1LIGAND2222+2H2Opq-FeIIIs

37

P1-1LIGAND2222+2H2Opq-FeIIIs SCF Done: -3379.11559217 A.U.

P -1.726031 1.572546 -0.061821

O -1.933732 2.618117 1.074853

O -0.700424 0.490005 0.341418

C -0.144799 -0.707558 -0.451855

H -1.024916 -1.256493 -0.787514

H 0.378571 -0.265716 -1.299599

C -0.791352 3.045322 2.047663

H 0.071971 3.249174 1.414186

H -0.615536 2.159366 2.653346

C -1.281855 4.220350 2.806693

H -2.164341 3.988911 3.398674

H -0.492225 4.505440 3.505438

H -1.472350 5.081517 2.169054

C 0.733136 -1.471220 0.469647

H 0.186734 -1.866533 1.322747

H 1.132387 -2.319286 -0.089498

H 1.577650 -0.878195 0.812774

S -3.627830 0.690586 -0.341895

S -1.115449 2.482696 -1.851841

C -2.209116 3.932751 -1.809436

H -1.725669 4.683517 -2.437993

H -2.307393 4.342010 -0.804602

S -3.927666 3.776477 -2.484815

C -3.680711 3.072493 -4.210877

H -4.705317 2.998301 -4.577178

H -3.266560 2.070779 -4.084397

C -2.825025 3.980605 -5.049441

H -2.814691 3.567760 -6.060801

H -3.222952 4.991159 -5.117479

H -1.788165 4.017174 -4.719139

Fe -5.067643 2.187030 -1.132194

O -6.497459 1.206639 -2.164678

H -6.444473 0.290428 -2.479163

H -7.423088 1.479123 -2.269252

O -6.079338 3.121458 0.346677

H -6.205142 2.766884 1.240384

H -6.564670 3.960237 0.308521

P1-1LIGAND2222+2H2Opq-MnIIs

37

P1-1LIGAND2222+2H2Opq-MnIIs SCF Done: -3266.96561607 A.U.

P -1.930601 1.399376 -0.313233

O -2.351197 2.346576 0.916235

O -0.737194 0.525136 0.216318

C -0.163397 -0.657613 -0.495784

H -0.990884 -1.341986 -0.687109

H 0.234458 -0.294924 -1.444883

C -1.297748 3.014957 1.745085

H -0.570920 3.456239 1.059835

H -0.812636 2.226776 2.317796

C -1.986089 4.028650 2.599461

H -2.710595 3.563188 3.267639

H -1.249949 4.528755 3.227127

H -2.479243 4.797295 2.003249

C 0.886317 -1.242210 0.388534

H 0.468638 -1.579368 1.334576

H 1.320090 -2.106420 -0.113189

H 1.687041 -0.532142 0.582640

S -3.520909 0.331038 -0.888121

S -1.073484 2.642172 -1.784564

C -2.254168 4.031684 -1.802585

H -1.725905 4.824938 -2.330081

H -2.467329 4.382120 -0.793241

S -3.886751 3.829583 -2.634669

C -3.427373 2.924734 -4.186106

H -4.388778 2.760332 -4.673361

H -3.019100 1.957005 -3.890229

C -2.480884 3.722237 -5.043547

H -2.312085 3.183500 -5.975529

H -2.880351 4.703176 -5.294078

H -1.506394 3.850214 -4.573122

Mn -5.021270 2.186519 -1.148064

O -7.007064 1.734789 -1.682534

H -7.662904 2.315419 -2.087607

H -7.437046 0.876748 -1.576587

O -5.034841 3.084418 0.756572

H -4.248330 2.887777 1.293742

H -5.726597 3.423165 1.336021

P1-1LIGAND2222+2H2Opq-NiIIt

37

P1-1LIGAND2222+2H2Opq-NiIIt SCF Done: -3624.22839477 A.U.

P -1.957105 1.477017 -0.364167

O -2.407405 2.364610 0.896007

O -0.807589 0.544197 0.157685

C -0.213472 -0.595672 -0.610496

H -1.044732 -1.203353 -0.971061

H 0.313202 -0.158924 -1.460154

C -1.385310 2.976338 1.806125

H -0.594146 3.392972 1.179116

H -0.978437 2.158216 2.397476

C -2.086827 4.008427 2.626484

H -2.890948 3.571291 3.217705

H -1.379409 4.452688 3.325036

H -2.482499 4.818224 2.011672

C 0.689598 -1.334596 0.318429

H 0.143130 -1.743984 1.165131

H 1.138778 -2.165495 -0.224272

H 1.494722 -0.700661 0.683344

S -3.560132 0.483645 -1.041726

S -1.032920 2.775737 -1.740776

C -2.277546 4.103922 -1.797443

H -1.782934 4.931740 -2.304899

H -2.547961 4.435430 -0.795661

S -3.858554 3.804346 -2.685792

C -3.288223 2.947075 -4.223597

H -4.218326 2.720895 -4.745990

H -2.823345 2.009512 -3.914157

C -2.368651 3.816517 -5.041316

H -2.137759 3.298648 -5.971769

H -2.822604 4.771729 -5.298434

H -1.419364 4.000716 -4.539060

Ni -5.003677 2.236074 -1.422015

O -6.858324 1.446318 -1.120269

H -7.374189 0.865968 -1.692596

H -7.221428 1.355714 -0.230906

O -5.055380 3.072647 0.422771

H -4.239120 2.921482 0.935096

H -5.426587 3.924445 0.681086

P1-1LIGAND2222+2H2Opq-ZnII

37

P1-1LIGAND2222+2H2Opq-ZnII SCF Done: -3895.28773631 A.U.

P -2.093185 1.348079 0.230393

O -2.155974 2.473596 1.337177

O -1.071973 0.263983 0.722490

C -0.820055 -1.060822 0.079996

H -1.186738 -1.784168 0.805573

H -1.434022 -1.127404 -0.820463

C -0.915559 2.973917 2.010740

H -0.166517 3.152074 1.235120

H -0.571663 2.164692 2.652946

C -1.285460 4.208313 2.763852

H -2.056288 4.006040 3.504177

H -0.405670 4.575141 3.290919

H -1.628476 5.002370 2.101708

C 0.642707 -1.186352 -0.193215

H 1.225781 -1.080303 0.718993

H 0.840522 -2.177134 -0.600478

H 0.986703 -0.451473 -0.919448

S -3.939225 0.574568 -0.040834

S -1.199932 2.186829 -1.498735

C -2.027234 3.807724 -1.521953

H -1.356702 4.479732 -2.054737

H -2.161406 4.193600 -0.512189

S -3.672695 3.946687 -2.345274

C -3.337456 3.209761 -4.016106

H -4.317180 3.228633 -4.493718

H -3.046412 2.169323 -3.862529

C -2.307879 3.996375 -4.782440

H -2.228207 3.581846 -5.786850

H -2.580214 5.045780 -4.879426

H -1.316231 3.930793 -4.336263

Zn -4.888195 2.259267 -1.234442

O -6.215169 1.439080 -2.593752

H -6.142290 0.547422 -2.954146

H -7.149349 1.677500 -2.628043

O -6.409948 3.200979 -0.187317

H -6.724302 2.873146 0.664289

H -6.649581 4.134262 -0.229411

P1-1LIGAND2222+2H2Otd-CoIIq

37

P1-1LIGAND2222+2H2Otd-CoIIq SCF Done: -3498.69167798 A.U.

P -1.789695 1.255545 -0.632966

O -2.404977 2.111898 0.594189

O -0.775394 0.249772 0.013719

C 0.005358 -0.787631 -0.737179

H -0.717461 -1.375844 -1.304360

H 0.659514 -0.249118 -1.424637

C -1.497788 2.689204 1.637391

H -0.650924 3.145510 1.119322

H -1.133561 1.846368 2.222424

C -2.286953 3.669334 2.441439

H -3.071567 3.180573 3.017341

H -1.626988 4.154565 3.159255

H -2.723657 4.453094 1.820572

C 0.755809 -1.592662 0.268759

H 0.083524 -2.103408 0.954434

H 1.335018 -2.349773 -0.258557

H 1.450215 -0.977256 0.836434

S -3.402414 0.477084 -1.542196

S -0.683253 2.589373 -1.827983

C -1.667118 4.111552 -1.579340

H -1.154367 4.862612 -2.179926

H -1.632450 4.433977 -0.541174

S -3.435874 4.136101 -2.048702

C -3.392008 3.531905 -3.800928

H -4.443597 3.533001 -4.087605

H -3.045351 2.497647 -3.772865

C -2.555225 4.414329 -4.688667

H -2.655975 4.068125 -5.716986

H -2.873973 5.454491 -4.658163

H -1.495253 4.362340 -4.443613

Co -4.397552 2.392324 -0.780832

O -6.336947 2.259756 -1.743929

H -6.664320 1.454200 -2.161141

H -6.869054 2.977084 -2.107160

O -5.475430 2.587108 0.895769

H -5.164418 2.538586 1.806420

H -6.434548 2.480039 0.903538

P1-1LIGAND2222+2H2Otd-CuIId

37

P1-1LIGAND2222+2H2Otd-CuIId SCF Done: -3756.38834635 A.U.

P -1.819186 1.991125 0.399259

O -1.295616 2.843743 1.608557

O -1.032205 0.625164 0.457388

C -1.283891 -0.552147 -0.402828

H -2.340498 -0.807680 -0.294126

H -1.090502 -0.249211 -1.434317

C 0.153328 2.894472 1.990157

H 0.713633 3.153195 1.088833

H 0.422608 1.885990 2.301302

C 0.289086 3.906539 3.077377

H -0.306692 3.638518 3.946838

H 1.332971 3.944073 3.386294

H 0.002612 4.901254 2.742739

C -0.374903 -1.646075 0.055019

H -0.575900 -1.922982 1.087359

H -0.537384 -2.524565 -0.567763

H 0.669967 -1.358303 -0.038232

S -3.831108 1.915629 0.668203

S -1.277056 2.839134 -1.446947

C -2.407939 4.269185 -1.398125

H -1.912200 5.085777 -1.921542

H -2.604028 4.590172 -0.376047

S -4.037260 4.029455 -2.194829

C -3.551492 3.659521 -3.935631

H -4.507313 3.455055 -4.417708

H -2.959864 2.741143 -3.923986

C -2.821013 4.809243 -4.586743

H -2.672145 4.571806 -5.639694

H -3.386938 5.737388 -4.535089

H -1.833820 4.973462 -4.157765

Cu -4.859640 2.021759 -1.325080

O -5.666592 1.311692 -3.054855

H -6.538951 0.913056 -2.936319

H -5.237492 0.848928 -3.783160

O -6.756238 1.362027 -0.568842

H -6.822208 0.622041 0.047923

H -7.412783 2.002226 -0.265519

P1-1LIGAND2222+2H2Otd-FeIIId

37

P1-1LIGAND2222+2H2Otd-FeIIId SCF Done: -3379.04919930 A.U.

P -1.662758 1.458481 -0.133031

O -0.926673 2.445645 0.826375

O -1.470138 -0.060699 0.066973

C -0.287323 -0.977385 -0.332907

H -0.626193 -1.922232 0.081811

H -0.326839 -1.014427 -1.420378

C -0.558063 2.217444 2.304296

H 0.514689 2.034422 2.247640

H -1.071495 1.309126 2.622106

C -0.920529 3.435168 3.071501

H -1.997037 3.601889 3.097525

H -0.587794 3.289396 4.100623

H -0.419098 4.322749 2.691281

C 0.993859 -0.477013 0.229140

H 0.976961 -0.432148 1.316562

H 1.773445 -1.190775 -0.046053

H 1.292569 0.487333 -0.186489

S -3.738834 1.693479 0.357706

S -1.256485 2.075676 -2.070162

C -1.978064 3.748747 -1.896835

H -1.445348 4.397746 -2.593398

H -1.839674 4.154800 -0.895689

S -3.755755 3.905562 -2.308751

C -3.822546 3.555949 -4.144967

H -4.853251 3.817928 -4.386908

H -3.665728 2.483931 -4.278242

C -2.837882 4.398151 -4.915166

H -3.062566 4.263621 -5.975151

H -2.931630 5.461372 -4.700703

H -1.806175 4.080851 -4.775424

Fe -5.106336 2.475609 -1.147703

O -6.445655 2.029248 -2.572041

H -7.279073 1.576750 -2.368904

H -6.377388 2.043660 -3.536756

O -6.617698 2.524176 0.175299

H -7.459157 2.984694 0.036093

H -6.586989 2.282881 1.112826

P1-1LIGAND2222+2H2Otd-FeIIIs

37

P1-1LIGAND2222+2H2Otd-FeIIIs SCF Done: -3379.11550719 A.U.

P -1.693290 1.519614 -0.155397

O -1.948811 2.381678 1.118093

O -0.676597 0.391057 0.119928

C -0.108045 -0.714783 -0.792397

H -0.975897 -1.145706 -1.293119

H 0.523883 -0.187101 -1.506060

C -0.828119 2.694918 2.156789

H -0.009796 3.122500 1.577015

H -0.545051 1.720957 2.550017

C -1.411432 3.615679 3.161342

H -2.246845 3.166775 3.693702

H -0.633533 3.822622 3.899507

H -1.708612 4.570196 2.731330

C 0.625928 -1.664457 0.079742

H -0.027589 -2.152242 0.799094

H 1.045880 -2.438927 -0.564722

H 1.454173 -1.185268 0.596422

S -3.577370 0.684926 -0.629665

S -1.024563 2.691990 -1.763502

C -2.136631 4.111138 -1.550077

H -1.639657 4.950600 -2.040698

H -2.281324 4.367127 -0.501082

S -3.824327 4.043746 -2.314572

C -3.488511 3.575161 -4.104092

H -4.491131 3.567853 -4.533094

H -3.089831 2.559443 -4.091757

C -2.577612 4.573265 -4.762983

H -2.517684 4.303972 -5.819825

H -2.958094 5.591572 -4.711024

H -1.559541 4.542444 -4.377353

Fe -5.001107 2.277782 -1.244255

O -6.382712 1.465992 -2.469304

H -7.295053 1.772112 -2.595465

H -6.328389 0.590870 -2.884634

O -6.063888 2.981926 0.324665

H -6.191502 2.509225 1.161738

H -6.575974 3.803147 0.387793

P1-1LIGAND2222+2H2Otd-MnIId

37

P1-1LIGAND2222+2H2Otd-MnIId SCF Done: -3266.86635247 A.U.

P -1.866376 1.528933 0.358147

O -1.181103 2.738312 1.087336

O -0.930079 0.287709 0.606413

C -1.269809 -1.132941 0.323262

H -2.257148 -1.317767 0.749504

H -1.316712 -1.235220 -0.762876

C 0.273033 2.794231 1.477325

H 0.849152 2.735680 0.552642

H 0.464265 1.904825 2.074608

C 0.475238 4.068613 2.223755

H -0.139015 4.108376 3.120280

H 1.519139 4.125101 2.530850

H 0.262923 4.939853 1.607222

C -0.201382 -1.979199 0.932713

H -0.161018 -1.853809 2.012319

H -0.423671 -3.025038 0.725384

H 0.775380 -1.754611 0.509895

S -3.833223 1.365433 0.709481

S -1.905525 1.920005 -1.794005

C -2.228121 3.746065 -1.707782

H -1.774698 4.209061 -2.583305

H -1.773428 4.149355 -0.804236

S -4.029803 4.026523 -1.687625

C -4.337112 4.482296 -3.459339

H -5.413530 4.351781 -3.574421

H -3.841825 3.731949 -4.076812

C -3.893851 5.889113 -3.766593

H -4.124870 6.115122 -4.807116

H -4.404374 6.620945 -3.144332

H -2.819640 6.021596 -3.640814

Mn -4.262236 1.625191 -1.520597

O -4.687782 1.087912 -3.447395

H -5.592762 0.866330 -3.701727

H -4.112192 0.859524 -4.185632

O -6.362310 1.483090 -1.341029

H -6.773710 0.741635 -0.878469

H -6.870476 2.255833 -1.064931

P1-1LIGAND2222+2H2Otd-MnIIs

37

P1-1LIGAND2222+2H2Otd-MnIIs SCF Done: -3266.96563248 A.U.

P -1.959052 1.331800 -0.375426

O -2.453716 2.056659 0.970555

O -0.797067 0.369297 0.064591

C -0.197313 -0.687871 -0.805944

H -1.003745 -1.377073 -1.058885

H 0.156116 -0.195117 -1.713507

C -1.447359 2.574759 1.952686

H -0.652322 3.059577 1.382209

H -1.040206 1.701459 2.458893

C -2.161778 3.512232 2.870161

H -2.956366 3.008336 3.420060

H -1.458717 3.897323 3.607173

H -2.573689 4.369967 2.337027

C 0.902145 -1.332435 -0.030077

H 0.529009 -1.793136 0.881942

H 1.345623 -2.115851 -0.643302

H 1.685586 -0.621256 0.222287

S -3.504206 0.391397 -1.228955

S -1.003121 2.794136 -1.554730

C -2.201622 4.166312 -1.463889

H -1.635545 5.037651 -1.790160

H -2.526329 4.340314 -0.438386

S -3.733663 4.105577 -2.490692

C -3.101548 3.432935 -4.097253

H -4.005502 3.318739 -4.695925

H -2.694568 2.441365 -3.891803

C -2.101958 4.355978 -4.742144

H -1.825204 3.949055 -5.714373

H -2.506987 5.353246 -4.902026

H -1.183119 4.440367 -4.162549

Mn -4.982513 2.275348 -1.355222

O -6.943950 1.905613 -2.014376

H -7.557828 2.519184 -2.436476

H -7.397422 1.053775 -1.978389

O -5.066631 2.951384 0.646300

H -4.331945 2.640328 1.203883

H -5.782145 3.250430 1.218821

P1-1LIGAND2222+2H2Otd-NiIIt

37

P1-1LIGAND2222+2H2Otd-NiIIt SCF Done: -3624.23354047 A.U.

P -1.651680 1.224135 -0.744747

O -2.380137 2.014909 0.491849

O -0.546620 0.329846 -0.086251

C 0.126167 -0.848103 -0.732226

H -0.667436 -1.484560 -1.125888

H 0.721013 -0.450782 -1.555323

C -1.560761 2.639457 1.580414

H -0.700325 3.114727 1.103360

H -1.207738 1.811729 2.193039

C -2.418348 3.601372 2.332588

H -3.251140 3.097906 2.820837

H -1.821396 4.069357 3.114350

H -2.798316 4.399539 1.692902

C 0.950790 -1.514419 0.315303

H 0.337531 -1.887918 1.132401

H 1.458691 -2.365238 -0.137630

H 1.711421 -0.845578 0.711681

S -3.181242 0.326861 -1.641403

S -0.656467 2.697824 -1.866348

C -1.766025 4.128900 -1.584295

H -1.345543 4.916577 -2.210040

H -1.706789 4.462467 -0.550262

S -3.543882 4.001268 -1.969404

C -3.536931 3.407640 -3.719598

H -4.596826 3.257195 -3.926781

H -3.043905 2.434468 -3.722381

C -2.898160 4.397897 -4.658032

H -3.018899 4.036269 -5.678784

H -3.360531 5.381447 -4.599972

H -1.827487 4.500307 -4.486192

Ni -4.268908 2.158453 -0.668103

O -6.044927 1.861500 -1.664706

H -6.882150 2.159302 -1.288783

H -6.240522 1.087861 -2.206592

O -5.413473 3.233459 0.696885

H -5.655487 2.930441 1.580074

H -5.482214 4.195292 0.710155

P1-1LIGAND2222+2H2Otd-ZnII

37

P1-1LIGAND2222+2H2Otd-ZnII SCF Done: -3895.28830147 A.U.

P -1.886418 1.351098 -0.003169

O -1.958802 2.279180 1.272429

O -0.753297 0.293990 0.258513

C -0.447245 -0.869094 -0.621191

H -1.378025 -1.424137 -0.747638

H -0.133236 -0.466100 -1.585972

C -0.712820 2.773806 1.941294

H -0.035370 3.126145 1.159315

H -0.267584 1.907904 2.428912

C -1.114764 3.847725 2.896131

H -1.815003 3.476324 3.640937

H -0.227468 4.200919 3.420040

H -1.558535 4.701692 2.386004

C 0.623223 -1.665048 0.048196

H 0.295331 -2.038945 1.015485

H 0.864900 -2.522081 -0.578908

H 1.531040 -1.080532 0.180283

S -3.680125 0.465799 -0.287563

S -1.184494 2.524793 -1.620862

C -2.159855 4.035753 -1.336211

H -1.578159 4.854272 -1.756261

H -2.290545 4.222508 -0.271173

S -3.840483 4.141828 -2.089596

C -3.486772 3.733942 -3.865071

H -4.479056 3.704796 -4.315165

H -3.064935 2.727416 -3.887489

C -2.590900 4.756691 -4.511061

H -2.500348 4.520969 -5.570957

H -2.992643 5.764995 -4.430621

H -1.582057 4.747094 -4.099953

Zn -4.830303 2.170362 -1.253881

O -6.054347 1.412845 -2.738428

H -7.013008 1.521218 -2.731853

H -5.869189 0.610385 -3.240456

O -6.463686 2.752446 -0.114209

H -6.732742 2.270102 0.677457

H -6.800935 3.650746 -0.015209

P1-1LIGAND2222+4H2Ooh-CoIIq

43

P1-1LIGAND2222+4H2Ooh-CoIIq SCF Done: -3651.64195402 A.U.

P -1.840284 1.620642 0.337989

O -1.353545 2.682944 1.397540

O -0.925712 0.348268 0.561805

C -1.092901 -0.927741 -0.153808

H -2.157008 -1.179157 -0.137793

H -0.777432 -0.762902 -1.187798

C 0.094917 2.916813 1.666754

H 0.616143 2.926111 0.706069

H 0.439339 2.060999 2.245324

C 0.208351 4.211214 2.402252

H -0.349860 4.187777 3.335596

H 1.255508 4.389456 2.642872

H -0.142682 5.049536 1.802298

C -0.251115 -1.951583 0.538160

H -0.570148 -2.093671 1.568383

H -0.346205 -2.904086 0.019166

H 0.799070 -1.668942 0.530127

S -3.796442 1.299207 0.581076

S -1.302195 2.329542 -1.587279

C -2.108357 3.978512 -1.426705

H -1.505889 4.666456 -2.016938

H -2.057641 4.296366 -0.386450

S -3.836183 4.120346 -1.964491

C -3.627734 4.143586 -3.798415

H -4.645151 4.029788 -4.172402

H -3.072135 3.247792 -4.082676

C -2.985433 5.409512 -4.305243

H -2.933418 5.375505 -5.392892

H -3.554249 6.292838 -4.023443

H -1.964142 5.531449 -3.946225

Co -5.064394 2.052312 -1.293233

O -6.449854 0.400233 -0.853678

H -6.396904 -0.302277 -0.196549

H -7.232652 0.224976 -1.387240

O -4.116397 0.865718 -2.765432

H -4.257920 -0.088902 -2.757169

H -3.154662 1.002826 -2.789139

O -6.540107 2.293456 -2.906903

H -7.240955 2.954839 -2.888108

H -6.373258 2.090656 -3.833945

O -6.461941 3.238312 -0.248650

H -7.068168 2.814575 0.370822

H -6.277191 4.116117 0.106119

P1-1LIGAND2222+4H2Ooh-CuIId

43

P1-1LIGAND2222+4H2Ooh-CuIId SCF Done: -3909.33927547 A.U.

P -1.536787 1.885156 0.487606

O -0.854921 2.680151 1.661451

O -0.687149 0.556758 0.328282

C -1.099692 -0.597100 -0.481481

H -2.142673 -0.816728 -0.233068

H -1.041460 -0.297756 -1.531360

C 0.627086 2.789702 1.796829

H 1.016418 3.122778 0.831793

H 0.995931 1.785671 2.002295

C 0.900128 3.754504 2.902543

H 0.472033 3.411918 3.841997

H 1.977932 3.837347 3.035761

H 0.512318 4.745983 2.678121

C -0.178677 -1.730910 -0.160474

H -0.241688 -2.006221 0.889900

H -0.455111 -2.599917 -0.755349

H 0.852770 -1.478532 -0.395987

S -3.469252 1.716621 1.003531

S -1.277739 2.885137 -1.346795

C -2.439439 4.255705 -1.019153

H -2.052888 5.129295 -1.542616

H -2.481786 4.488061 0.043774

S -4.159158 4.004149 -1.576100

C -3.901623 3.849252 -3.393708

H -4.851970 3.462056 -3.759212

H -3.135456 3.085103 -3.542938

C -3.549388 5.167128 -4.039090

H -3.461888 5.022606 -5.115430

H -4.315964 5.919352 -3.864048

H -2.593983 5.560345 -3.694635

Cu -4.777753 1.818998 -0.904847

O -5.800702 0.115491 -0.211173

H -5.642008 -0.250358 0.668065

H -6.759621 0.167506 -0.317851

O -4.198518 0.318285 -2.549727

H -4.407102 -0.593516 -2.314116

H -3.508389 0.261583 -3.219277

O -6.453428 1.934819 -2.090785

H -6.892253 2.788888 -2.345123

H -6.335501 1.419358 -2.898371

O -7.380990 4.331813 -2.814844

H -7.735234 4.944390 -2.161171

H -7.951360 4.444552 -3.583473

P1-1LIGAND2222+4H2Ooh-FeIIIs

43

P1-1LIGAND2222+4H2Ooh-FeIIIs SCF Done: -3532.10077099 A.U.

P -1.754972 1.697624 0.319998

O -1.270806 2.746605 1.360909

O -1.052362 0.335230 0.591862

C -1.094729 -0.959783 -0.172147

H -2.134628 -1.289905 -0.143212

H -0.800401 -0.724998 -1.197221

C 0.185953 2.862757 1.849659

H 0.810814 2.810357 0.957123

H 0.336628 1.977860 2.464393

C 0.294566 4.144860 2.590670

H -0.377245 4.178609 3.445191

H 1.314243 4.218994 2.972607

H 0.121741 5.008914 1.952234

C -0.158405 -1.902821 0.501930

H -0.453947 -2.094177 1.530818

H -0.182346 -2.851198 -0.035136

H 0.865071 -1.535884 0.480242

S -3.813574 1.693404 0.627864

S -1.286435 2.309411 -1.643199

C -2.108754 3.948935 -1.530576

H -1.537410 4.622370 -2.169482

H -2.053009 4.324801 -0.509156

S -3.852154 4.056443 -2.067900

C -3.707153 4.135647 -3.923185

H -4.746932 4.089814 -4.245653

H -3.204093 3.222381 -4.247000

C -3.022584 5.393050 -4.390501

H -3.042391 5.398497 -5.481119

H -3.529144 6.292260 -4.046989

H -1.974242 5.442034 -4.100719

Fe -5.115791 2.046520 -1.243801

O -6.406627 0.386712 -0.876993

H -6.340334 -0.267657 -0.167647

H -7.214684 0.182322 -1.367615

O -4.123386 0.850570 -2.680583

H -4.343700 -0.062746 -2.911039

H -3.166831 0.962901 -2.814373

O -6.511662 2.371522 -2.882073

H -7.277337 2.960562 -2.837953

H -6.460867 2.050048 -3.791925

O -6.556252 3.241384 -0.243510

H -7.275427 2.879918 0.294007

H -6.395335 4.135276 0.090947

P1-1LIGAND2222+4H2Ooh-MnIId

43

P1-1LIGAND2222+4H2Ooh-MnIId SCF Done: -3419.82996844 A.U.

P -1.828774 1.675139 0.313030

O -1.291298 2.733206 1.351264

O -0.969244 0.368834 0.566986

C -1.186233 -0.908666 -0.128779

H -2.258026 -1.124788 -0.095172

H -0.879970 -0.769563 -1.169634

C 0.164207 2.906335 1.628327

H 0.686989 2.911992 0.668566

H 0.474758 2.029141 2.193835

C 0.323589 4.183418 2.385110

H -0.236075 4.164253 3.317710

H 1.376336 4.319440 2.629210

H 0.003265 5.043116 1.798841

C -0.369271 -1.951098 0.565734

H -0.678483 -2.068499 1.602031

H -0.502210 -2.906866 0.061346

H 0.689364 -1.702953 0.540004

S -3.807230 1.457915 0.539783

S -1.287635 2.314770 -1.636380

C -2.131982 3.941136 -1.519438

H -1.579684 4.626396 -2.159934

H -2.063180 4.306443 -0.496037

S -3.890383 3.991780 -2.011020

C -3.693771 4.035426 -3.851883

H -4.708236 3.889231 -4.222670

H -3.107169 3.162746 -4.146380

C -3.105038 5.328649 -4.355652

H -3.070124 5.307800 -5.444298

H -3.699588 6.188714 -4.055243

H -2.082500 5.484012 -4.013706

Mn -5.077435 2.104109 -1.338620

O -6.399604 0.547382 -0.750628

H -6.244959 -0.166544 -0.122312

H -7.156139 0.296300 -1.292853

O -4.108238 0.896192 -2.667661

H -4.221118 -0.060553 -2.599994

H -3.147665 1.063729 -2.681582

O -6.560629 2.274741 -2.837042

H -7.206089 2.991245 -2.803145

H -6.365105 2.120873 -3.768844

O -6.318952 3.288979 -0.241084

H -6.868586 2.861426 0.428624

H -6.034817 4.134080 0.129337

P1-1LIGAND2222+4H2Ooh-NiIIt

43

P1-1LIGAND2222+4H2Ooh-NiIIt SCF Done: -3777.19414054 A.U.

P -1.853888 1.798250 0.381131

O -1.264714 2.764353 1.478141

O -0.996702 0.467984 0.494900

C -1.306623 -0.775247 -0.220340

H -2.387125 -0.937792 -0.152042

H -1.031102 -0.623696 -1.268275

C 0.201051 2.901593 1.707560

H 0.690660 2.927384 0.730793

H 0.513570 2.001658 2.235341

C 0.413212 4.151782 2.495816

H -0.116538 4.116594 3.445303

H 1.476030 4.258142 2.708892

H 0.092435 5.034677 1.945462

C -0.521213 -1.879330 0.413467

H -0.799889 -2.013037 1.456407

H -0.719515 -2.811219 -0.113804

H 0.547301 -1.683595 0.357331

S -3.816129 1.658897 0.666827

S -1.340569 2.581392 -1.527293

C -2.306770 4.135554 -1.355608

H -1.745133 4.910355 -1.873366

H -2.360505 4.410775 -0.302891

S -4.009586 4.156673 -1.991184

C -3.745728 4.107809 -3.818247

H -4.761249 4.183053 -4.207833

H -3.361141 3.121355 -4.081348

C -2.874030 5.226568 -4.329117

H -2.884617 5.213671 -5.418316

H -3.229838 6.203496 -4.006531

H -1.833402 5.115531 -4.027082

Ni -5.050722 2.047585 -1.367641

O -6.095855 0.224131 -0.945676

H -5.996007 -0.328977 -0.162493

H -6.945704 0.005825 -1.344971

O -3.929234 0.929002 -2.735394

H -4.054384 -0.027567 -2.698065

H -2.972382 1.089131 -2.669919

O -6.493152 2.132916 -2.931550

H -7.242202 2.738943 -2.902488

H -6.310971 1.948605 -3.859600

O -6.562406 3.119166 -0.311328

H -7.054217 2.680845 0.393452

H -6.377950 4.006185 0.021326

P1-1LIGAND2222+4H2Ooh-ZnII

43

P1-1LIGAND2222+4H2Ooh-ZnII SCF Done: -4048.23444703 A.U.

P -1.854810 1.782344 0.407648

O -1.248482 2.758655 1.485957

O -0.990967 0.455890 0.508640

C -1.313054 -0.792234 -0.192908

H -2.393936 -0.947673 -0.117178

H -1.043297 -0.650710 -1.243734

C 0.220728 2.918891 1.671115

H 0.680851 2.945309 0.680130

H 0.561842 2.027098 2.195025

C 0.437405 4.177227 2.445282

H -0.063476 4.140343 3.410230

H 1.504303 4.301044 2.625780

H 0.086898 5.051772 1.899486

C -0.529651 -1.894956 0.445651

H -0.802422 -2.017778 1.491484

H -0.736254 -2.830472 -0.071944

H 0.539556 -1.705628 0.381192

S -3.805727 1.618823 0.757884

S -1.370723 2.553253 -1.512813

C -2.283142 4.141907 -1.336040

H -1.687062 4.898442 -1.841844

H -2.334168 4.410692 -0.281481

S -3.979372 4.224171 -1.985424

C -3.702298 4.155731 -3.810678

H -4.716245 4.204404 -4.208676

H -3.296677 3.173477 -4.057691

C -2.848638 5.284887 -4.327308

H -2.832117 5.248524 -5.415925

H -3.237558 6.258198 -4.033743

H -1.813181 5.209589 -3.997712

Zn -4.998346 2.040806 -1.304365

O -6.157156 0.227133 -0.914443

H -6.049944 -0.393085 -0.184633

H -6.961140 -0.021927 -1.383391

O -3.941713 0.888549 -2.770827

H -4.084154 -0.064250 -2.819937

H -2.981421 1.029030 -2.750769

O -6.469432 2.129778 -2.900354

H -7.239530 2.706903 -2.847098

H -6.310899 1.950010 -3.833382

O -6.643953 3.091436 -0.316538

H -7.201992 2.626625 0.318090

H -6.525575 3.977192 0.046988

P1-1LIGAND2222ax+3H2O-CoIIq

40

P1-1LIGAND2222ax+3H2O-CoIIq SCF Done: -3575.16879263 A.U.

P -1.895454 1.413713 -0.055548

O -0.439765 1.806609 0.409781

O -2.058985 -0.172280 -0.099581

C -0.882140 -1.089743 -0.275117

H -1.283393 -1.891048 -0.891445

H -0.121663 -0.555906 -0.845947

C -0.110581 2.374928 1.752672

H 0.859576 1.933946 1.969319

H -0.838599 1.996235 2.471725

C -0.077869 3.866469 1.680694

H -1.066640 4.280380 1.476776

H 0.246425 4.265098 2.641267

H 0.626804 4.211353 0.925601

C -0.397805 -1.559661 1.057358

H -1.188474 -2.051391 1.620133

H 0.402178 -2.283722 0.905992

H 0.007725 -0.744113 1.654333

S -3.416533 2.249442 0.903278

S -1.984081 1.929361 -2.113277

C -2.017390 3.766780 -1.904523

H -1.504239 4.201449 -2.760684

H -1.471115 4.038220 -1.002683

S -3.713004 4.424588 -1.792894

C -4.069862 4.699954 -3.588138

H -5.156185 4.777541 -3.627234

H -3.781096 3.790545 -4.118979

C -3.391557 5.932465 -4.128676

H -3.656638 6.054384 -5.178568

H -3.702504 6.829884 -3.598479

H -2.305267 5.865723 -4.079340

Co -4.510722 2.129072 -1.224414

O -6.531839 2.467022 -0.496333

H -6.875241 1.784660 0.093777

H -6.855792 3.301157 -0.137168

O -4.904799 0.046936 -0.870629

H -5.301091 -0.543536 -1.521086

H -4.205462 -0.456642 -0.427001

O -5.608908 1.828256 -2.997699

H -6.561393 1.981459 -2.968352

H -5.376634 1.598632 -3.903695

P1-1LIGAND2222ax+3H2O-CuIId

40

P1-1LIGAND2222ax+3H2O-CuIId SCF Done: -3832.86479040 A.U.

P -1.849942 2.062678 0.529326

O -1.296637 2.726844 1.839015

O -1.109033 0.666856 0.409888

C -1.428756 -0.332278 -0.623265

H -2.022497 -1.094845 -0.117958

H -2.053280 0.141988 -1.389806

C 0.152721 2.706546 2.204870

H 0.705173 3.102939 1.349905

H 0.422002 1.661426 2.352080

C 0.308851 3.535111 3.435732

H -0.273847 3.132982 4.261609

H 1.357458 3.525772 3.730198

H 0.016731 4.569028 3.265423

C -0.149033 -0.865421 -1.183800

H 0.467570 -1.303190 -0.401727

H -0.368106 -1.645049 -1.911756

H 0.423017 -0.085505 -1.683854

S -3.852329 2.081300 0.770693

S -1.230368 3.067222 -1.212202

C -2.508598 4.363898 -1.272561

H -2.059893 5.221750 -1.772289

H -2.817144 4.671280 -0.275444

S -4.029473 3.936558 -2.191392

C -3.335615 3.474822 -3.836952

H -4.216002 3.165381 -4.397706

H -2.694631 2.603434 -3.685417

C -2.609000 4.619818 -4.499740

H -2.328630 4.316497 -5.507989

H -3.232139 5.508225 -4.583995

H -1.687611 4.886363 -3.983842

Cu -4.853481 1.904949 -1.302171

O -6.983188 2.712955 -0.796215

H -7.563969 2.281063 -0.158785

H -7.161452 3.654308 -0.683181

O -5.371343 -0.053020 -0.813290

H -6.038404 -0.630174 -1.201149

H -5.149837 -0.411867 0.054574

O -5.930829 1.681262 -3.076824

H -6.853091 1.927506 -2.918105

H -5.915375 1.008801 -3.766683

P1-1LIGAND2222ax+3H2O-FeIIIs

40

P1-1LIGAND2222ax+3H2O-FeIIIs SCF Done: -3455.61460647 A.U.

P -1.861060 1.309827 -0.057537

O -0.558229 2.096790 0.256268

O -1.811970 -0.241507 0.048776

C -0.549275 -1.110710 -0.187161

H -0.949913 -1.911593 -0.802893

H 0.143060 -0.504198 -0.771305

C 0.015840 2.412745 1.656791

H 1.001016 1.954902 1.603041

H -0.601586 1.878653 2.380222

C 0.032382 3.886611 1.832370

H -0.971441 4.310741 1.851261

H 0.493680 4.105639 2.796907

H 0.632632 4.378977 1.069697

C -0.020233 -1.554845 1.127364

H -0.757366 -2.116357 1.696731

H 0.823132 -2.221350 0.937015

H 0.353187 -0.726546 1.727731

S -3.543282 1.977023 0.891682

S -2.215048 1.778774 -2.127247

C -2.169687 3.631669 -2.016365

H -1.731174 3.996706 -2.944920

H -1.534454 3.928228 -1.182409

S -3.846309 4.321051 -1.806946

C -4.284634 4.791863 -3.559022

H -5.363952 4.942842 -3.511996

H -4.078665 3.922223 -4.184576

C -3.548395 6.032986 -3.990902

H -3.874396 6.280702 -5.002316

H -3.769270 6.886642 -3.354338

H -2.468847 5.893092 -4.032981

Fe -4.661616 2.013275 -1.167670

O -6.526672 2.713749 -0.535735

H -7.235984 2.143724 -0.202205

H -6.745370 3.611094 -0.243316

O -5.221320 -0.011021 -0.972328

H -5.714444 -0.545168 -1.610972

H -5.037576 -0.578488 -0.209926

O -5.496753 1.646200 -3.129960

H -6.350976 1.990025 -3.427282

H -5.115044 1.183242 -3.888327

P1-1LIGAND2222ax+3H2O-MnIIs

40

P1-1LIGAND2222ax+3H2O-MnIIs SCF Done: -3343.44759555 A.U.

P -1.928215 1.299876 0.025955

O -0.503646 1.878328 0.404581

O -1.928187 -0.285384 0.038280

C -0.718941 -1.078207 -0.343067

H -1.130489 -1.901134 -0.923192

H -0.097072 -0.465033 -0.997572

C -0.166394 2.407834 1.757654

H 0.836993 2.025530 1.931315

H -0.845182 1.957873 2.483785

C -0.233492 3.900331 1.743226

H -1.250750 4.252060 1.565331

H 0.079430 4.285517 2.712928

H 0.434259 4.319045 0.991748

C -0.001128 -1.530112 0.887341

H -0.653361 -2.107565 1.539049

H 0.832006 -2.169346 0.597450

H 0.407546 -0.691136 1.448743

S -3.461895 2.024934 1.040162

S -2.074425 1.767610 -2.048331

C -2.016301 3.612256 -1.890241

H -1.494402 3.987680 -2.768420

H -1.429635 3.868570 -1.009991

S -3.650703 4.407251 -1.776314

C -3.985164 4.738928 -3.566931

H -5.054593 4.948934 -3.594997

H -3.816773 3.807652 -4.111251

C -3.169680 5.886069 -4.104843

H -3.428019 6.052695 -5.150186

H -3.362606 6.807750 -3.560122

H -2.098693 5.687846 -4.069595

Mn -4.737623 2.102671 -1.140414

O -6.706401 2.778129 -0.436887

H -7.308072 2.194157 0.040680

H -6.934727 3.671912 -0.154964

O -5.231349 -0.011679 -0.819776

H -5.661926 -0.643014 -1.407021

H -4.867111 -0.518462 -0.082884

O -5.734248 1.769371 -3.095222

H -6.641331 2.040988 -3.278067

H -5.387712 1.417488 -3.922720

P1-1LIGAND2222ax+3H2O-NiIIt

40

P1-1LIGAND2222ax+3H2O-NiIIt SCF Done: -3700.71112121 A.U.

P -1.781733 1.652893 -0.060302

O -0.400740 2.072387 0.580256

O -1.962457 0.075523 -0.074893

C -0.794269 -0.842294 -0.263799

H -1.171892 -1.594217 -0.953221

H 0.006601 -0.286677 -0.755077

C -0.198148 2.393753 2.023169

H 0.728729 1.878203 2.265631

H -1.010459 1.944201 2.597369

C -0.103644 3.874745 2.198516

H -1.042868 4.371494 1.955733

H 0.125185 4.096520 3.240179

H 0.693805 4.293912 1.587743

C -0.381112 -1.413488 1.053273

H -1.205100 -1.932343 1.538227

H 0.419332 -2.134680 0.891806

H -0.000899 -0.646485 1.725947

S -3.352121 2.539164 0.801723

S -1.541826 2.135325 -2.098695

C -1.970464 3.912250 -1.993914

H -1.400513 4.427028 -2.764608

H -1.675361 4.316841 -1.027180

S -3.732874 4.318396 -2.224332

C -3.988608 3.889024 -4.003093

H -5.038671 4.133603 -4.162158

H -3.877501 2.808732 -4.111646

C -3.088886 4.659229 -4.935767

H -3.386605 4.451816 -5.962912

H -3.161720 5.734128 -4.781279

H -2.044437 4.363218 -4.848441

Ni -4.945894 2.694103 -0.949814

O -6.286087 1.666809 0.273137

H -6.077812 1.414007 1.180830

H -7.242247 1.781308 0.220167

O -4.666079 1.008429 -2.078759

H -4.921000 0.175980 -1.657885

H -3.748409 0.892228 -2.379326

O -6.711635 3.045413 -2.087076

H -7.249672 3.841753 -2.163372

H -6.931919 2.485987 -2.840943

P1-1LIGAND2222ax+3H2Opir-CoIIq

40

P1-1LIGAND2222ax+3H2Opir-CoIIq SCF Done: -3575.16440357 A.U.

P -1.772166 1.529171 0.138665

O -1.693915 2.444915 1.421483

O -0.713482 0.379112 0.343544

C -0.549058 -0.791587 -0.552367

H -1.534583 -1.245636 -0.672446

H -0.207651 -0.413905 -1.518195

C -0.379949 2.869188 1.991460

H 0.285706 3.106517 1.158088

H 0.012340 2.001747 2.520079

C -0.635308 4.037872 2.884455

H -1.331131 3.784320 3.681400

H 0.304047 4.341079 3.344988

H -1.024749 4.892154 2.332450

C 0.439735 -1.710514 0.087031

H 0.087532 -2.062844 1.053914

H 0.581597 -2.577510 -0.556545

H 1.405872 -1.227719 0.215882

S -3.662267 0.815957 0.003989

S -1.091572 2.618413 -1.521679

C -2.177108 4.080664 -1.321002

H -1.617961 4.923401 -1.723215

H -2.360865 4.274484 -0.264711

S -3.801045 4.034227 -2.142246

C -3.321484 3.751222 -3.900790

H -4.281108 3.740812 -4.420120

H -2.876054 2.755355 -3.960430

C -2.410081 4.818877 -4.450221

H -2.269281 4.653135 -5.517597

H -2.826716 5.816064 -4.321666

H -1.420166 4.792635 -3.996332

Co -5.048359 2.089460 -1.280651

O -6.665959 0.577740 -0.771456

H -6.509851 -0.198504 -0.219963

H -7.620590 0.713630 -0.756613

O -5.544189 1.321227 -3.100435

H -6.098422 0.530467 -3.091695

H -5.171239 1.412590 -3.983493

O -6.266891 3.408655 -0.291241

H -6.824149 3.187294 0.463623

H -6.217965 4.370926 -0.338570

P1-1LIGAND2222ax+3H2Opir-CuIId

40

P1-1LIGAND2222ax+3H2Opir-CuIId SCF Done: -3832.86512880 A.U.

P -1.724494 2.063368 0.517519

O -1.006809 2.797263 1.706227

O -1.050912 0.630353 0.441419

C -1.520670 -0.455132 -0.429270

H -2.604505 -0.537853 -0.295420

H -1.314503 -0.159389 -1.461580

C 0.471381 2.713600 1.919945

H 0.945582 3.025596 0.986483

H 0.700261 1.664765 2.104899

C 0.802992 3.602242 3.071551

H 0.290200 3.286174 3.977268

H 1.875072 3.545019 3.256383

H 0.552440 4.640511 2.865842

C -0.795862 -1.702948 -0.036994

H -1.007313 -1.976253 0.994257

H -1.116786 -2.522848 -0.677805

H 0.279045 -1.586820 -0.156038

S -3.689433 2.178435 0.935498

S -1.259443 2.941031 -1.339718

C -2.333365 4.408778 -1.185278

H -1.855982 5.210078 -1.747915

H -2.423398 4.726387 -0.148027

S -4.032121 4.229048 -1.836221

C -3.677685 3.977151 -3.626909

H -4.611723 3.586146 -4.029867

H -2.920438 3.192833 -3.695608

C -3.254244 5.253747 -4.313879

H -3.115308 5.051413 -5.375502

H -4.003885 6.036249 -4.219346

H -2.306830 5.636042 -3.937268

Cu -4.833978 2.150647 -1.077873

O -5.930391 0.508229 -0.371751

H -5.760704 0.169871 0.517207

H -6.890221 0.579594 -0.456314

O -4.446310 0.731991 -2.719490

H -4.729528 -0.168260 -2.518995

H -3.762327 0.650637 -3.392392

O -6.658385 2.476248 -2.164865

H -6.802797 2.087149 -3.035168

H -7.285111 3.204000 -2.077633

P1-1LIGAND2222ax+3H2Opir-FeIIIs

40

P1-1LIGAND2222ax+3H2Opir-FeIIIs SCF Done: -3455.60967392 A.U.

P -1.644324 1.468797 -0.152011

O -0.616482 2.422218 0.533174

O -1.444413 -0.060081 0.022841

C -0.085424 -0.800068 -0.104163

H -0.183167 -1.319539 -1.054852

H 0.682938 -0.029566 -0.178206

C -0.218941 2.377741 2.014229

H 0.744744 1.870544 1.994140

H -0.952584 1.751541 2.525783

C -0.154957 3.773330 2.519186

H -1.130835 4.256705 2.516590

H 0.192203 3.741456 3.552787

H 0.555421 4.374516 1.955752

C 0.053574 -1.696206 1.070179

H -0.758231 -2.417220 1.129959

H 0.981823 -2.257761 0.946254

H 0.126521 -1.148514 2.007826

S -3.539699 1.872307 0.661220

S -1.535830 1.960949 -2.181674

C -1.997574 3.734955 -2.075305

H -1.479444 4.235898 -2.893817

H -1.647267 4.167054 -1.139247

S -3.780082 4.118034 -2.251341

C -4.034765 4.018916 -4.101117

H -5.119290 3.972803 -4.204348

H -3.615045 3.063958 -4.423105

C -3.436580 5.207207 -4.806176

H -3.659288 5.105505 -5.869765

H -3.865453 6.148036 -4.469153

H -2.352704 5.257805 -4.715931

Fe -4.965872 2.269197 -1.042305

O -6.645271 1.022656 -0.668624

H -6.662974 0.316021 -0.006684

H -7.529535 1.046119 -1.063096

O -4.657552 0.962853 -2.607468

H -5.223297 0.213165 -2.843346

H -3.799447 0.837783 -3.042639

O -6.260657 3.733568 -0.376754

H -7.050827 3.584062 0.162979

H -6.097898 4.688346 -0.374401

P1-1LIGAND2222ax+3H2Opir-MnIIs

40

P1-1LIGAND2222ax+3H2Opir-MnIIs SCF Done: -3343.44460727 A.U.

P -1.752022 1.359652 -0.077933

O -0.455535 2.005572 0.562470

O -1.645584 -0.215297 -0.162552

C -0.341964 -0.911731 -0.385793

H -0.593171 -1.694215 -1.098280

H 0.348693 -0.212096 -0.859870

C -0.304030 2.338201 2.005037

H 0.698755 1.988239 2.240980

H -1.023939 1.751453 2.578453

C -0.466594 3.811854 2.195400

H -1.478569 4.136562 1.951739

H -0.282916 4.063832 3.239041

H 0.247030 4.368287 1.589944

C 0.169121 -1.445408 0.913073

H -0.555901 -2.106643 1.382834

H 1.076522 -2.019704 0.729770

H 0.420430 -0.645238 1.607806

S -3.431468 1.884394 0.872892

S -1.602238 1.978949 -2.087396

C -1.961502 3.770405 -1.863298

H -1.336907 4.305246 -2.576119

H -1.670905 4.080868 -0.860455

S -3.693624 4.265430 -2.124121

C -3.840804 4.090162 -3.961207

H -4.914896 4.142644 -4.140492

H -3.516761 3.079776 -4.219014

C -3.087016 5.154804 -4.714907

H -3.252337 5.017966 -5.783180

H -3.423116 6.155193 -4.450781

H -2.011151 5.099893 -4.553460

Mn -4.962349 2.295809 -0.977277

O -6.537533 0.876214 -0.313754

H -6.349936 0.214463 0.362895

H -7.444942 0.715979 -0.597479

O -4.697702 0.995019 -2.680976

H -5.228469 0.241038 -2.962569

H -3.796234 0.851008 -3.004377

O -6.480455 3.752785 -0.439162

H -7.216242 3.593623 0.163148

H -6.412222 4.710103 -0.533660

P1-1LIGAND2222ax+3H2Opir-NiIIt

40

P1-1LIGAND2222ax+3H2Opir-NiIIt SCF Done: -3700.70881785 A.U.

P -2.009757 1.852596 0.608314

O -1.435120 2.808354 1.715782

O -1.299216 0.455488 0.841175

C -1.709290 -0.808657 0.217225

H -1.727533 -1.520728 1.039885

H -2.735650 -0.691679 -0.148043

C 0.001764 2.791358 2.127725

H 0.599173 2.919524 1.222127

H 0.194122 1.803721 2.544428

C 0.190032 3.893863 3.115952

H -0.438333 3.751957 3.992389

H 1.228446 3.893595 3.444492

H -0.023608 4.867879 2.680644

C -0.740619 -1.201760 -0.856558

H 0.270926 -1.279584 -0.463875

H -1.014928 -2.178267 -1.254352

H -0.726234 -0.492063 -1.685786

S -4.002700 1.915998 0.757138

S -1.289194 2.469098 -1.289597

C -2.200099 4.060417 -1.388466

H -1.566128 4.745430 -1.949102

H -2.339017 4.470790 -0.389564

S -3.833620 4.034656 -2.193305

C -3.360738 3.780054 -3.962273

H -4.293345 3.488706 -4.445632

H -2.690181 2.919341 -3.996705

C -2.760978 5.018404 -4.578267

H -2.540137 4.819189 -5.626324

H -3.443447 5.864475 -4.536437

H -1.823071 5.308379 -4.106346

Ni -4.916453 2.038566 -1.401278

O -6.138406 0.321422 -1.099472

H -6.102081 -0.179790 -0.276101

H -6.987353 0.115890 -1.509088

O -3.812214 0.803489 -2.608141

H -4.025937 -0.132124 -2.709167

H -2.846627 0.891669 -2.583264

O -6.594609 3.178549 -0.973280

H -7.289700 2.903976 -0.363784

H -6.576626 4.142912 -0.945701

P1-1LIGAND2222ax+3H2Opir-ZnII

40

P1-1LIGAND2222ax+3H2Opir-ZnII SCF Done: -3971.75852087 A.U.

P -1.871779 1.946373 0.475087

O -1.254590 2.698116 1.709246

O -1.133108 0.545943 0.415142

C -1.527721 -0.557961 -0.472622

H -2.612552 -0.677982 -0.384308

H -1.289205 -0.257840 -1.495841

C 0.215030 2.714966 1.972953

H 0.707497 3.020951 1.046707

H 0.499282 1.689577 2.205522

C 0.452929 3.662301 3.101432

H -0.079632 3.352337 3.997752

H 1.517661 3.671156 3.330759

H 0.155874 4.677355 2.846569

C -0.777741 -1.778491 -0.044010

H -1.020839 -2.054445 0.979578

H -1.045043 -2.610982 -0.693032

H 0.296636 -1.627046 -0.120957

S -3.854996 1.907669 0.812952

S -1.307438 2.864852 -1.332868

C -2.357003 4.365073 -1.244859

H -1.763820 5.190615 -1.632301

H -2.627719 4.596055 -0.215772

S -3.913818 4.288303 -2.201202

C -3.266098 3.973352 -3.903013

H -4.163470 3.759651 -4.485335

H -2.656864 3.067642 -3.851339

C -2.502507 5.146874 -4.461220

H -2.218585 4.928112 -5.489935

H -3.099546 6.056247 -4.467450

H -1.580549 5.340278 -3.914346

Zn -4.834978 2.206856 -1.263912

O -6.094403 0.279029 -0.776598

H -5.853031 -0.222672 0.012008

H -6.986609 -0.011459 -1.001956

O -4.528560 0.933021 -2.861102

H -4.967224 0.076770 -2.780982

H -4.325716 1.080392 -3.790627

O -6.758952 2.996112 -1.170882

H -7.402910 2.691359 -0.521530

H -6.976391 3.916632 -1.360337

P1-1LIGAND2222ax+3H2O-ZnII

40

P1-1LIGAND2222ax+3H2O-ZnII SCF Done: -3971.76216674 A.U.

P -1.710269 2.215931 0.008918

O -0.423570 2.554155 0.853865

O -1.756981 0.620364 -0.254462

C -0.533260 -0.100443 -0.723444

H -0.593510 -0.123005 -1.812512

H 0.335434 0.493301 -0.433775

C -0.249177 2.053351 2.253123

H 0.629868 1.412598 2.197145

H -1.114516 1.439357 2.513299

C -0.069275 3.220963 3.165654

H -0.961356 3.843829 3.201475

H 0.125103 2.855246 4.173303

H 0.777919 3.832342 2.863080

C -0.530002 -1.461076 -0.106045

H -1.411870 -2.034234 -0.387003

H 0.343716 -2.007904 -0.458297

H -0.477350 -1.408468 0.979776

S -3.421955 2.762910 0.863902

S -1.323436 3.069701 -1.869344

C -2.425195 4.526568 -1.836096

H -1.950570 5.271765 -2.471583

H -2.508625 4.933714 -0.830712

S -4.135856 4.279216 -2.444270

C -3.854236 3.529899 -4.112968

H -4.867431 3.392157 -4.491575

H -3.403197 2.547245 -3.964408

C -3.032841 4.414144 -5.014925

H -3.006974 3.975451 -6.011816

H -3.453655 5.414045 -5.104484

H -1.998854 4.498859 -4.682576

Zn -4.883249 2.468981 -1.030198

O -6.575045 3.165618 0.110633

H -6.842605 2.747829 0.938807

H -6.739276 4.108624 0.234757

O -4.235718 0.544224 -1.498348

H -4.829986 -0.213614 -1.528746

H -3.412530 0.260540 -1.053074

O -6.552231 1.834367 -2.214344

H -7.440854 2.124806 -1.978304

H -6.606595 1.434854 -3.089209

P1-1LIGAND2222eq+3H2O-CoIIq

40

P1-1LIGAND2222eq+3H2O-CoIIq SCF Done: -3575.16439184 A.U.

P -1.764175 1.538933 0.126344

O -1.675188 2.467292 1.399554

O -0.718769 0.378894 0.343033

C -0.560761 -0.796599 -0.547902

H -1.550234 -1.240368 -0.674027

H -0.208196 -0.425852 -1.512398

C -0.356140 2.881926 1.964626

H 0.310799 3.105845 1.128596

H 0.027830 2.014602 2.499448

C -0.597841 4.059970 2.849037

H -1.294736 3.819749 3.649171

H 0.345440 4.356910 3.305625

H -0.979404 4.914005 2.291196

C 0.413494 -1.724061 0.101350

H 0.050539 -2.069937 1.066589

H 0.550972 -2.594268 -0.538837

H 1.383644 -1.251025 0.235932

S -3.662006 0.844500 0.001420

S -1.070201 2.601507 -1.545249

C -2.138345 4.079482 -1.366225

H -1.569726 4.908247 -1.783988

H -2.317631 4.293109 -0.312964

S -3.764228 4.039102 -2.183747

C -3.293494 3.703522 -3.935656

H -4.253114 3.709064 -4.454911

H -2.876644 2.694468 -3.973424

C -2.352200 4.732864 -4.507215

H -2.215262 4.539502 -5.570459

H -2.740458 5.744187 -4.401425

H -1.363799 4.688370 -4.051457

Co -5.043188 2.143400 -1.262028

O -6.201276 3.512489 -0.267276

H -6.748102 3.319261 0.502826

H -6.134688 4.472233 -0.338252

O -5.604461 1.355264 -3.054123

H -6.180783 0.581535 -3.009460

H -5.251751 1.410704 -3.948337

O -6.699482 0.689551 -0.691552

H -7.648987 0.855823 -0.662778

H -6.558098 -0.081374 -0.128710

P1-1LIGAND2222eq+3H2O-CuIId

40

P1-1LIGAND2222eq+3H2O-CuIId SCF Done: -3832.86515809 A.U.

P -1.720631 2.085245 0.518577

O -1.012594 2.832977 1.704325

O -1.044260 0.652684 0.461013

C -1.508281 -0.442462 -0.400698

H -2.592827 -0.524697 -0.272158

H -1.296635 -0.157377 -1.434861

C 0.464051 2.756312 1.930273

H 0.944884 3.054248 0.995586

H 0.693692 1.711205 2.134016

C 0.784829 3.664165 3.069895

H 0.264994 3.362013 3.976340

H 1.855400 3.611740 3.264604

H 0.534469 4.698536 2.845290

C -0.784403 -1.685220 0.008893

H -1.001057 -1.947640 1.041870

H -1.100985 -2.512355 -0.624694

H 0.290996 -1.569156 -0.105640

S -3.688481 2.200764 0.923113

S -1.245721 2.945322 -1.344363

C -2.321063 4.414242 -1.209279

H -1.841099 5.210177 -1.777349

H -2.416118 4.741775 -0.175577

S -4.016688 4.227965 -1.866569

C -3.654318 3.965811 -3.654341

H -4.585526 3.569339 -4.058500

H -2.894437 3.183431 -3.715478

C -3.232287 5.239482 -4.347565

H -3.087259 5.030684 -5.407129

H -3.985363 6.019700 -4.261865

H -2.288224 5.627851 -3.968777

Cu -4.817907 2.153051 -1.098552

O -6.632213 2.459372 -2.206017

H -7.264701 3.183550 -2.131750

H -6.765143 2.061163 -3.074009

O -4.407620 0.721009 -2.724276

H -4.689448 -0.178631 -2.519252

H -3.718573 0.636997 -3.391641

O -5.912117 0.511125 -0.387646

H -6.871736 0.576875 -0.478811

H -5.746250 0.181554 0.505350

P1-1LIGAND2222eq+3H2O-FeIIIs

40

P1-1LIGAND2222eq+3H2O-FeIIIs SCF Done: -3455.61459356 A.U.

P -1.743508 1.437371 -0.234232

O -2.117822 2.296600 1.042384

O -0.676182 0.390090 0.159530

C 0.068678 -0.643456 -0.706138

H -0.715500 -1.244728 -1.165705

H 0.595123 -0.058376 -1.460361

C -1.033772 2.723356 2.060201

H -0.193969 3.091180 1.469743

H -0.760161 1.803568 2.572090

C -1.650831 3.751525 2.938421

H -2.505745 3.359603 3.488464

H -0.910408 4.049877 3.682525

H -1.928651 4.653535 2.392532

C 0.963427 -1.404125 0.201672

H 0.408280 -1.945690 0.963884

H 1.494405 -2.139670 -0.405188

H 1.709075 -0.765241 0.668995

S -3.525110 0.512244 -0.803875

S -0.992666 2.649611 -1.759289

C -2.166679 4.040981 -1.635234

H -1.664214 4.870409 -2.135964

H -2.340525 4.327878 -0.599427

S -3.819848 3.895896 -2.432571

C -3.406833 3.338127 -4.163223

H -4.386040 3.312463 -4.641255

H -3.016978 2.321134 -4.092403

C -2.462668 4.294977 -4.841973

H -2.362117 3.980403 -5.881980

H -2.835072 5.317706 -4.847289

H -1.461051 4.277745 -4.413876

Fe -5.025005 2.163887 -1.167108

O -4.684306 3.283930 0.560331

H -3.930035 3.047979 1.133879

H -5.093790 4.093570 0.897468

O -5.979954 1.467577 -2.906690

H -6.755484 1.841651 -3.350862

H -5.748933 0.661887 -3.391809

O -6.902105 1.859743 -0.337765

H -7.135185 2.038505 0.584183

H -7.618495 1.319102 -0.699739

P1-1LIGAND2222eq+3H2O-MnIIs

40

P1-1LIGAND2222eq+3H2O-MnIIs SCF Done: -3343.44276859 A.U.

P -1.850673 1.880212 0.481393

O -1.178445 2.792073 1.571426

O -1.045887 0.514002 0.510580

C -1.443061 -0.694131 -0.222534

H -2.536214 -0.756201 -0.199632

H -1.117648 -0.560305 -1.257714

C 0.303449 2.885229 1.724182

H 0.717455 3.107106 0.737568

H 0.645290 1.900181 2.039710

C 0.579167 3.954661 2.728416

H 0.130843 3.719552 3.691181

H 1.656391 4.031722 2.869692

H 0.214096 4.924178 2.395845

C -0.786852 -1.868437 0.431370

H -1.116846 -1.983633 1.461483

H -1.045596 -2.775398 -0.112925

H 0.296245 -1.769135 0.419188

S -3.800146 1.788764 0.903679

S -1.409258 2.653856 -1.446794

C -2.365822 4.226188 -1.346185

H -1.759096 4.985447 -1.836339

H -2.496255 4.518837 -0.305290

S -4.009766 4.215768 -2.124334

C -3.548661 4.133135 -3.913767

H -4.478059 3.862152 -4.414645

H -2.854891 3.297860 -4.030193

C -2.981675 5.431196 -4.427693

H -2.759765 5.327050 -5.489394

H -3.683479 6.254240 -4.312453

H -2.049167 5.703256 -3.934599

Mn -4.891737 1.936640 -1.261840

O -6.816044 2.928818 -1.012806

H -7.558722 2.578972 -0.507088

H -6.985573 3.872641 -1.117186

O -3.913637 0.802059 -2.827038

H -4.202638 0.015679 -3.304071

H -2.992412 0.966666 -3.069500

O -6.178337 0.136613 -0.997661

H -6.973363 -0.158697 -1.456525

H -6.023861 -0.505988 -0.295160

P1-1LIGAND2222eq+3H2O-NiIIt

40

P1-1LIGAND2222eq+3H2O-NiIIt SCF Done: -3700.70943581 A.U.

P -1.877775 1.903931 0.391898

O -1.274242 2.784445 1.544989

O -1.085720 0.530047 0.452232

C -1.438971 -0.658744 -0.330744

H -2.528982 -0.764096 -0.301616

H -1.124248 -0.472673 -1.362300

C 0.191135 2.826515 1.836795

H 0.703898 3.029338 0.893422

H 0.465686 1.831422 2.184409

C 0.406754 3.889932 2.861558

H -0.146202 3.678323 3.774055

H 1.466182 3.923407 3.112111

H 0.118070 4.871747 2.492007

C -0.734208 -1.834241 0.268457

H -1.050741 -2.002947 1.295345

H -0.965736 -2.726958 -0.310335

H 0.344414 -1.695144 0.251273

S -3.856597 1.850316 0.676183

S -1.320993 2.706886 -1.491078

C -2.350761 4.227180 -1.425970

H -1.794196 4.999214 -1.954474

H -2.476938 4.547748 -0.393020

S -4.012999 4.134757 -2.165484

C -3.606657 3.979218 -3.961507

H -4.551977 3.686222 -4.418492

H -2.916747 3.140327 -4.067742

C -3.064254 5.259821 -4.543578

H -2.896802 5.116907 -5.610653

H -3.758664 6.088710 -4.424369

H -2.107152 5.544933 -4.108809

Ni -4.925703 2.047769 -1.402967

O -6.632255 3.059865 -0.801837

H -7.263895 2.716815 -0.159106

H -6.679367 4.021630 -0.740484

O -3.833883 0.947440 -2.743151

H -3.997130 0.005314 -2.873023

H -2.874994 1.091820 -2.766472

O -6.038719 0.255080 -1.116037

H -6.896367 0.020645 -1.490402

H -5.922914 -0.290095 -0.328738

P1-1LIGAND2222eq+3H2Opir-CoIId

40

P1-1LIGAND2222eq+3H2Opir-CoIId SCF Done: -3575.15072509 A.U.

P -1.910350 2.231512 0.588001

O -1.255697 2.837253 1.876907

O -1.367816 0.741486 0.500858

C -1.757080 -0.196847 -0.558943

H -2.322730 -0.980267 -0.054981

H -2.435105 0.317035 -1.255524

C 0.182016 2.633505 2.232051

H 0.773802 2.970060 1.378021

H 0.320717 1.561429 2.366047

C 0.446405 3.421976 3.470782

H -0.179487 3.087578 4.295191

H 1.486677 3.275960 3.759457

H 0.287540 4.486304 3.312830

C -0.527809 -0.707301 -1.240558

H 0.142999 -1.180767 -0.526727

H -0.804279 -1.454117 -1.983402

H 0.011441 0.092462 -1.746512

S -3.891759 2.536531 0.826040

S -1.203641 3.100788 -1.198663

C -2.454899 4.406569 -1.392305

H -2.008639 5.191062 -2.001281

H -2.741801 4.831310 -0.433445

S -4.004472 3.884498 -2.218123

C -3.342493 3.291702 -3.840822

H -4.240306 3.003933 -4.387075

H -2.747571 2.396220 -3.645161

C -2.564117 4.350099 -4.582375

H -2.341537 3.983960 -5.583988

H -3.127213 5.275971 -4.688137

H -1.608286 4.572194 -4.110769

Co -4.809486 2.037369 -1.180992

O -5.370327 0.268753 -0.393829

H -5.324478 0.089117 0.553486

H -6.068050 -0.276717 -0.776296

O -5.835013 1.374091 -2.814184

H -6.671721 1.835676 -2.960963

H -5.530225 1.032900 -3.662007

O -6.813159 2.965165 -0.864693

H -7.436884 2.643788 -0.202734

H -6.924224 3.923749 -0.857979

P1-1LIGAND2222eq+3H2Opir-CoIIq

40

P1-1LIGAND2222eq+3H2Opir-CoIIq SCF Done: -3575.16158423 A.U.

P -1.939551 2.032482 0.608723

O -1.264458 2.838791 1.773483

O -1.321861 0.575970 0.672314

C -1.755501 -0.540713 -0.180999

H -2.315539 -1.206071 0.477033

H -2.436582 -0.154400 -0.948708

C 0.205538 2.792781 2.037478

H 0.710360 3.002387 1.091375

H 0.431715 1.772792 2.345685

C 0.496636 3.805885 3.093912

H -0.049469 3.590768 4.009727

H 1.560956 3.774489 3.323280

H 0.253477 4.813915 2.764851

C -0.546025 -1.191315 -0.773734

H 0.132356 -1.534012 0.004613

H -0.848589 -2.059131 -1.357671

H -0.007679 -0.510205 -1.431407

S -3.926383 2.238489 0.902283

S -1.339511 2.724869 -1.286029

C -2.327252 4.271076 -1.331986

H -1.727153 5.011352 -1.857374

H -2.515220 4.641624 -0.325797

S -3.944598 4.148691 -2.162272

C -3.429991 3.727752 -3.888689

H -4.381338 3.675818 -4.417900

H -2.988084 2.728771 -3.873811

C -2.509399 4.751938 -4.501899

H -2.365169 4.511901 -5.554765

H -2.920973 5.758145 -4.445954

H -1.521236 4.751704 -4.043687

Co -4.854147 2.071581 -1.200108

O -5.570914 0.079933 -0.795137

H -5.379856 -0.432732 -0.002040

H -6.068227 -0.488362 -1.393251

O -5.879238 1.665451 -3.024886

H -6.785965 1.991994 -3.084192

H -5.619626 1.372006 -3.905029

O -6.831251 2.808584 -0.711234

H -7.391268 2.305660 -0.107658

H -6.998042 3.735333 -0.501335

P1-1LIGAND2222eq+3H2Opir-CuIId

40

P1-1LIGAND2222eq+3H2Opir-CuIId SCF Done: -3832.86474736 A.U.

P -1.866430 2.110154 0.563026

O -1.254823 2.798232 1.834119

O -1.203261 0.672334 0.500412

C -1.553042 -0.349331 -0.499580

H -2.162197 -1.080678 0.032595

H -2.167788 0.116528 -1.278588

C 0.192223 2.684543 2.192608

H 0.768505 2.935364 1.298965

H 0.364737 1.639760 2.447560

C 0.441328 3.619558 3.328111

H -0.167309 3.365256 4.192845

H 1.487824 3.540289 3.620290

H 0.247722 4.653013 3.049277

C -0.289971 -0.931257 -1.049706

H 0.320543 -1.358711 -0.257220

H -0.531990 -1.728265 -1.751208

H 0.297502 -0.181254 -1.577347

S -3.858995 2.259119 0.827977

S -1.213127 3.004425 -1.228702

C -2.426595 4.356707 -1.341584

H -1.941458 5.169601 -1.880495

H -2.716951 4.720702 -0.358089

S -3.973361 3.964868 -2.235172

C -3.308433 3.346724 -3.842319

H -4.207069 3.102044 -4.407253

H -2.761870 2.423698 -3.636196

C -2.461851 4.372735 -4.554821

H -2.212477 3.992387 -5.544957

H -2.985245 5.318227 -4.686130

H -1.518817 4.561821 -4.044107

Cu -4.936866 2.077214 -1.206290

O -5.823741 0.324124 -0.534264

H -5.530087 -0.163449 0.244202

H -6.248882 -0.302448 -1.131170

O -6.107165 1.658567 -2.901331

H -6.945890 2.136994 -2.838773

H -5.979240 1.391151 -3.817414

O -6.994896 3.048669 -0.765172

H -7.572955 2.598794 -0.137467

H -7.166658 3.987782 -0.629428

P1-1LIGAND2222eq+3H2Opir-FeIIIs

40

P1-1LIGAND2222eq+3H2Opir-FeIIIs SCF Done: -3455.61603436 A.U.

P -1.789787 1.504522 -0.228795

O -2.080536 2.428175 1.024349

O -0.734036 0.445997 0.170459

C -0.166542 -0.733681 -0.635889

H -1.038508 -1.275761 -1.001883

H 0.376080 -0.277562 -1.463273

C -0.954258 2.832491 2.006660

H -0.149061 3.229644 1.388020

H -0.646310 1.899458 2.473032

C -1.540866 3.823214 2.946297

H -2.361564 3.401349 3.525228

H -0.767382 4.108967 3.660994

H -1.858962 4.736022 2.442676

C 0.690930 -1.519614 0.287300

H 0.125454 -1.931802 1.119699

H 1.101096 -2.356425 -0.280435

H 1.528802 -0.935803 0.661199

S -3.598829 0.650568 -0.755277

S -1.031782 2.633444 -1.826245

C -2.180694 4.040257 -1.789145

H -1.685101 4.824565 -2.364157

H -2.344297 4.412010 -0.778271

S -3.858903 3.852876 -2.550955

C -3.523781 3.030120 -4.190826

H -4.524425 2.930993 -4.612913

H -3.129724 2.036132 -3.971104

C -2.611115 3.864604 -5.050083

H -2.542843 3.379626 -6.025400

H -2.993460 4.870213 -5.213658

H -1.596729 3.923917 -4.658367

Fe -5.096532 2.364612 -1.092398

O -6.182299 1.174410 -2.384412

H -7.116099 1.309778 -2.604120

H -5.936376 0.303387 -2.730208

O -7.003398 3.322990 -0.888521

H -7.284923 4.152555 -1.300872

H -7.737423 3.048076 -0.319414

O -4.650187 3.338427 0.652307

H -5.206322 3.932841 1.174768

H -3.868365 3.080400 1.180839

P1-1LIGAND2222eq+3H2Opir-MnIIs

40

P1-1LIGAND2222eq+3H2Opir-MnIIs SCF Done: -3343.44323200 A.U.

P -2.021603 1.684141 0.516477

O -1.159377 2.737246 1.308313

O -1.255991 0.307220 0.667761

C -1.839398 -1.024896 0.434827

H -2.794427 -1.060728 0.964759

H -2.020022 -1.117623 -0.639620

C 0.333363 2.696818 1.313373

H 0.662185 2.574725 0.277751

H 0.615141 1.809483 1.878134

C 0.809162 3.968935 1.932544

H 0.435142 4.079906 2.947864

H 1.897321 3.952430 1.975944

H 0.511439 4.839205 1.350201

C -0.865082 -2.040434 0.939671

H -0.692662 -1.923905 2.007327

H -1.269811 -3.037305 0.771615

H 0.086877 -1.971714 0.417915

S -3.924733 1.769535 1.044637

S -1.812389 2.172565 -1.575195

C -2.173394 3.984939 -1.412190

H -1.555215 4.491975 -2.150656

H -1.864390 4.310756 -0.420104

S -3.913102 4.448773 -1.666291

C -3.912403 4.799231 -3.484442

H -4.969683 4.782716 -3.752159

H -3.430686 3.955253 -3.981529

C -3.268057 6.119331 -3.820320

H -3.322685 6.283190 -4.896172

H -3.769261 6.949560 -3.327859

H -2.213734 6.147768 -3.547171

Mn -4.650905 1.952604 -1.349021

O -5.406373 -0.123262 -1.236920

H -5.460667 -0.653210 -0.433164

H -5.678739 -0.693246 -1.964872

O -4.900642 1.507763 -3.514104

H -5.649674 1.818940 -4.036093

H -4.255776 1.171464 -4.146815

O -6.746617 2.631431 -1.287864

H -7.496504 2.048594 -1.118811

H -7.025684 3.505814 -0.990536

P1-1LIGAND2222eq+3H2Opir-NiIIt

40

P1-1LIGAND2222eq+3H2Opir-NiIIt SCF Done: -3700.71527773 A.U.

P -2.115275 1.665033 0.443895

O -1.234944 2.782329 1.115906

O -1.359279 0.309535 0.745919

C -1.925716 -1.043872 0.598340

H -2.908662 -1.039798 1.076252

H -2.041692 -1.229790 -0.473047

C 0.251004 2.670217 1.258025

H 0.656940 2.473393 0.262571

H 0.436974 1.804334 1.891077

C 0.734657 3.952059 1.849004

H 0.287538 4.133283 2.823716

H 1.813991 3.889388 1.981521

H 0.527125 4.802319 1.201740

C -0.979333 -2.004789 1.243074

H -0.868047 -1.796835 2.304894

H -1.372426 -3.014962 1.138310

H -0.000009 -1.974830 0.770550

S -4.032733 1.829346 0.854210

S -1.920643 1.943494 -1.711784

C -2.079107 3.783965 -1.704076

H -1.642334 4.138916 -2.635911

H -1.512111 4.186047 -0.867010

S -3.825100 4.317412 -1.599499

C -4.082218 4.986743 -3.303879

H -5.167803 5.038173 -3.399305

H -3.719619 4.238969 -4.010656

C -3.432243 6.334142 -3.491828

H -3.643032 6.696830 -4.497357

H -3.812041 7.068081 -2.784615

H -2.348583 6.288059 -3.387363

Ni -4.441890 1.965001 -1.546715

O -4.961749 -0.099476 -1.501579

H -4.916703 -0.675194 -0.730372

H -5.024078 -0.662016 -2.281716

O -4.853617 1.681536 -3.620665

H -5.690250 2.003496 -3.976492

H -4.253385 1.584678 -4.367762

O -6.525016 2.291867 -1.453379

H -7.067419 1.518044 -1.256687

H -6.920232 3.020864 -0.960096

P1-1LIGAND2222eq+3H2Opir-ZnII

40

P1-1LIGAND2222eq+3H2Opir-ZnII SCF Done: -3971.76216952 A.U.

P -1.712962 2.244755 0.020795

O -0.397675 2.530887 0.840222

O -1.862725 0.647135 -0.185110

C -0.694787 -0.166270 -0.643082

H -0.770526 -0.221124 -1.730005

H 0.214324 0.378972 -0.383383

C -0.233971 2.075691 2.255960

H 0.615952 1.395528 2.217871

H -1.123227 1.510687 2.545158

C 0.005798 3.267832 3.122184

H -0.857429 3.930909 3.140953

H 0.193374 2.932420 4.141589

H 0.876332 3.828884 2.789591

C -0.771512 -1.502731 0.020775

H -1.691161 -2.027837 -0.232637

H 0.061799 -2.116236 -0.319252

H -0.704184 -1.416231 1.103553

S -3.379758 2.922080 0.872060

S -1.290058 3.017975 -1.885151

C -2.338542 4.514863 -1.906766

H -1.838501 5.213211 -2.574946

H -2.399299 4.965572 -0.918591

S -4.060296 4.310966 -2.498186

C -3.817602 3.469128 -4.127911

H -4.836807 3.357518 -4.498743

H -3.410119 2.476141 -3.930928

C -2.960449 4.271407 -5.072086

H -2.957017 3.784226 -6.046569

H -3.336852 5.283496 -5.209902

H -1.922785 4.326202 -4.745146

Zn -4.888710 2.624968 -0.983231

O -4.375779 0.643057 -1.362295

H -3.562502 0.332474 -0.916824

H -5.015691 -0.077195 -1.343786

O -6.618202 2.048627 -2.107805

H -7.482025 2.408560 -1.875315

H -6.717101 1.597024 -2.952834

O -6.509825 3.490468 0.149279

H -6.787139 3.134628 1.002735

H -6.611420 4.447321 0.224968

P1-1LIGAND2222eq+3H2O-ZnII

40

P1-1LIGAND2222eq+3H2O-ZnII SCF Done: -3971.76731756 A.U.

P -1.784740 1.500439 -0.252483

O -2.101374 2.361546 1.062958

O -0.709584 0.439649 0.183095

C -0.224518 -0.685232 -0.669424

H -1.107241 -1.216536 -1.028217

H 0.298052 -0.238285 -1.516783

C -0.985845 2.850959 1.932194

H -0.188040 3.206200 1.276132

H -0.630155 1.984377 2.486487

C -1.543315 3.925835 2.807085

H -2.347139 3.550432 3.439822

H -0.759088 4.292798 3.467556

H -1.904717 4.775943 2.227508

C 0.664052 -1.532686 0.178784

H 0.125266 -1.950910 1.026140

H 1.036056 -2.359797 -0.424405

H 1.522076 -0.971484 0.542118

S -3.483394 0.677773 -0.924351

S -0.762071 2.763822 -1.596442

C -1.807710 4.262888 -1.566944

H -1.175666 5.036132 -2.001289

H -2.048679 4.558834 -0.546484

S -3.397170 4.277763 -2.498772

C -2.939501 3.395747 -4.059777

H -3.887706 3.320413 -4.592519

H -2.619057 2.390467 -3.780659

C -1.897333 4.146667 -4.845317

H -1.724012 3.628528 -5.787905

H -2.210828 5.162570 -5.078001

H -0.939483 4.187371 -4.326984

Zn -4.596690 2.678192 -1.212179

O -4.566076 3.602112 0.643405

H -3.913324 3.217368 1.252016

H -5.335091 3.874571 1.156601

O -6.521509 2.524064 -1.622740

H -7.014576 1.646576 -1.645012

H -7.072732 3.200195 -2.027256

O -7.634072 0.190665 -1.614681

H -8.227792 -0.107290 -0.918143

H -7.895922 -0.295850 -2.402788

P1-1LIGAND222+2OH2pq-CoIId

37

P1-1LIGAND222+2OH2pq-CoIId SCF Done: -3498.63863314 A.U.

Co -5.201696 2.076845 -1.460164

P -8.190711 1.947281 -1.129163

O -9.386154 2.930647 -0.949985

O -8.793718 0.488010 -0.977372

C -8.167625 -0.729840 -1.544104

H -7.798199 -0.478902 -2.539832

H -7.315774 -0.978782 -0.904103

C -10.698905 2.633196 -0.291531

H -10.476764 2.459422 0.763394

H -11.071036 1.712176 -0.739439

C -11.583147 3.810069 -0.528332

H -11.757834 3.970771 -1.589816

H -12.546084 3.618033 -0.056013

H -11.170264 4.717475 -0.093732

C -9.206113 -1.804422 -1.568094

H -10.045895 -1.529777 -2.202618

H -8.764939 -2.714510 -1.972273

H -9.579633 -2.029491 -0.570639

S -7.062870 2.377738 -2.718501

S -6.637250 2.290194 0.294683

C -6.830332 1.126155 1.874097

H -6.141286 1.643568 2.537369

H -6.459035 0.141563 1.602008

S -8.471072 1.120578 2.507674

C -9.005248 -0.611311 2.149266

H -8.556330 -1.235281 2.922141

H -8.579260 -0.893577 1.186281

C -10.510529 -0.702123 2.152375

H -10.811935 -1.742187 2.033518

H -10.938732 -0.353119 3.090765

H -10.952835 -0.128804 1.338291

O -3.446275 1.939177 -0.437563

H -3.069305 1.133200 -0.066466

H -3.073263 2.677033 0.060047

O -4.046873 1.904665 -3.078994

H -3.082997 1.905182 -3.038066

H -4.304597 1.933125 -4.007784

P1-1LIGAND222+2OH2pq-CoIIq

37

P1-1LIGAND222+2OH2pq-CoIIq SCF Done: -3498.66613333 A.U.

Co -5.219345 2.347367 -1.725316

P -7.957925 1.740246 -1.331530

O -9.041813 2.877907 -1.157269

O -8.820312 0.390731 -1.203050

C -8.244972 -0.909529 -1.546842

H -7.651386 -0.792185 -2.456426

H -7.564299 -1.194946 -0.736961

C -10.020646 2.874737 -0.062620

H -9.485138 2.662669 0.870585

H -10.720420 2.061727 -0.262197

C -10.686432 4.213707 -0.043086

H -11.181263 4.415284 -0.990717

H -11.442221 4.230032 0.740705

H -9.973480 5.010986 0.155213

C -9.364962 -1.885621 -1.733450

H -10.018686 -1.582651 -2.548293

H -8.956594 -2.865212 -1.977317

H -9.967510 -1.987135 -0.832230

S -7.023368 1.989720 -3.094923

S -6.508509 1.799093 0.138060

C -7.077264 -0.017398 2.447794

H -6.322405 0.661010 2.826286

H -6.779259 -0.797361 1.756442

S -8.569479 0.085933 3.094253

C -9.543412 -1.276363 2.317115

H -9.660772 -2.005683 3.120725

H -8.919711 -1.714888 1.538207

C -10.870497 -0.763689 1.811181

H -11.456884 -1.610746 1.457347

H -11.451672 -0.278091 2.593265

H -10.746018 -0.071892 0.979557

O -4.213563 4.070005 -1.302282

H -4.527525 4.692363 -0.635659

H -3.743958 4.592500 -1.964124

O -3.612341 1.453261 -2.611089

H -2.709034 1.417106 -2.276025

H -3.681440 0.790988 -3.308008

P1-1LIGAND222+2OH2pq-CuIId

37

P1-1LIGAND222+2OH2pq-CuIId SCF Done: -3756.37456445 A.U.

Cu -5.129122 2.219563 -1.461981

P -8.076219 1.648487 -1.349390

O -9.256469 2.668236 -1.197431

O -8.712777 0.196692 -1.266467

C -8.187937 -0.987188 -2.004478

H -8.318205 -0.777578 -3.065626

H -7.118770 -1.064678 -1.791265

C -10.312996 2.529539 -0.161320

H -9.798905 2.393830 0.798224

H -10.871852 1.623097 -0.400977

C -11.151405 3.763690 -0.199444

H -11.624566 3.889443 -1.170551

H -11.938697 3.674724 0.548266

H -10.567142 4.652017 0.028879

C -8.962697 -2.178055 -1.545057

H -10.027608 -2.053577 -1.730921

H -8.628581 -3.053361 -2.100101

H -8.808965 -2.379374 -0.485691

S -6.900284 2.089461 -2.886476

S -6.761790 1.851001 0.331947

C -7.004844 0.307132 1.422495

H -6.041936 0.206077 1.917627

H -7.199960 -0.553602 0.786543

S -8.301026 0.618689 2.604485

C -9.676388 -0.420710 1.965085

H -9.369156 -1.459400 2.104030

H -9.765042 -0.235544 0.892281

C -10.946836 -0.100342 2.714434

H -11.746440 -0.746647 2.355994

H -10.845267 -0.273176 3.784199

H -11.259971 0.932705 2.566162

O -3.596564 2.545716 -0.180200

H -2.725844 2.685522 -0.572894

H -3.618253 3.039416 0.647080

O -3.594848 2.430813 -2.907482

H -3.535640 3.232607 -3.442421

H -3.351515 1.708751 -3.500232

P1-1LIGAND222+2OH2pq-MnIId

37

P1-1LIGAND222+2OH2pq-MnIId SCF Done: -3266.82696524 A.U.

Mn -4.937133 2.372405 -1.394250

P -7.983969 2.022040 -1.370281

O -9.257955 2.877213 -1.078610

O -8.262804 0.588257 -1.936664

C -8.603495 -0.764902 -1.431657

H -8.173762 -1.407547 -2.196769

H -8.059849 -0.908419 -0.499660

C -10.667552 2.444143 -0.848112

H -10.671081 1.910858 0.103920

H -10.919696 1.757051 -1.655926

C -11.509908 3.675818 -0.838720

H -11.461968 4.200403 -1.790204

H -12.545548 3.383381 -0.668012

H -11.216665 4.354551 -0.041133

C -10.074383 -0.958810 -1.274922

H -10.610154 -0.735392 -2.195777

H -10.259595 -2.007150 -1.038313

H -10.477055 -0.359101 -0.460103

S -6.731094 3.008835 -2.621089

S -6.561983 2.145544 0.252192

C -6.833430 0.720922 1.404704

H -6.221629 0.963221 2.274921

H -6.490926 -0.210323 0.953017

S -8.607273 0.694802 1.785074

C -8.779123 -1.051788 2.354145

H -8.079358 -1.195733 3.176917

H -8.493034 -1.714759 1.534204

C -10.205441 -1.283826 2.794616

H -10.311891 -2.313063 3.133866

H -10.482829 -0.634100 3.622440

H -10.916369 -1.130505 1.983426

O -3.238369 2.135919 -0.262148

H -3.164426 1.747681 0.616824

H -2.441069 2.666232 -0.389193

O -3.688882 2.577379 -2.988108

H -2.817359 2.179214 -3.102844

H -3.977460 2.873526 -3.860446

P1-1LIGAND222+2OH2pq-MnIIs

37

P1-1LIGAND222+2OH2pq-MnIIs SCF Done: -3266.94375134 A.U.

Mn -5.158197 2.292099 -1.839381

P -7.955925 1.708113 -1.347923

O -9.002421 2.871288 -1.104830

O -8.849131 0.378829 -1.217406

C -8.295145 -0.926438 -1.578131

H -7.709315 -0.808702 -2.493030

H -7.611191 -1.226873 -0.776774

C -9.818272 2.957486 0.108608

H -9.140388 2.918381 0.968766

H -10.473168 2.083759 0.130156

C -10.585780 4.240817 0.050776

H -11.231684 4.270059 -0.823745

H -11.212975 4.328242 0.936591

H -9.919580 5.100094 0.020922

C -9.429616 -1.885752 -1.764562

H -10.087668 -1.565512 -2.569336

H -9.036197 -2.867729 -2.022934

H -10.024631 -1.990100 -0.858542

S -7.121518 1.974700 -3.154846

S -6.476809 1.666131 0.093901

C -7.318052 0.174764 2.532335

H -6.671225 0.959905 2.904405

H -6.875277 -0.661429 2.002752

S -8.890947 0.230762 2.958948

C -9.642749 -1.314167 2.282065

H -9.630152 -2.010103 3.122757

H -8.968948 -1.687070 1.510841

C -11.040468 -1.042138 1.782047

H -11.484332 -1.983749 1.461574

H -11.684602 -0.633810 2.558931

H -11.043137 -0.364441 0.929936

O -4.151679 4.168363 -1.585299

H -4.238282 4.728896 -0.805149

H -3.862023 4.749686 -2.298900

O -3.356934 1.420928 -2.560340

H -2.459672 1.650836 -2.292645

H -3.276424 0.717858 -3.215217

P1-1LIGAND222+2OH2pq-NiII

37

P1-1LIGAND222+2OH2pq-NiII SCF Done: -3624.20543268 A.U.

Ni -5.494895 2.178668 -0.935991

P -8.289149 1.415538 -1.122851

O -9.773572 1.814641 -0.810509

O -8.343679 -0.092802 -1.561361

C -9.499156 -0.684758 -2.317834

H -9.216441 -1.732584 -2.371146

H -10.372090 -0.578801 -1.675691

C -10.255421 3.192398 -0.489215

H -9.587441 3.898995 -0.984715

H -10.149162 3.291433 0.590706

C -11.669475 3.296851 -0.954885

H -12.305023 2.558514 -0.470961

H -12.048959 4.284509 -0.695552

H -11.747609 3.183064 -2.034372

C -9.658372 -0.043709 -3.657906

H -8.750915 -0.120189 -4.253985

H -10.455189 -0.551867 -4.199489

H -9.948265 1.005650 -3.576422

S -7.217803 2.729859 -2.202102

S -6.958497 1.407634 0.532005

C -6.816738 -0.508233 0.916102

H -5.961972 -0.549853 1.588474

H -6.597456 -0.974707 -0.038917

S -8.319556 -1.106336 1.597216

C -8.084435 -0.763619 3.394158

H -7.961506 0.313627 3.514564

H -7.160978 -1.260576 3.692431

C -9.277143 -1.281153 4.161356

H -9.129924 -1.088452 5.222540

H -10.200798 -0.788627 3.862039

H -9.402789 -2.355212 4.038115

O -3.948586 1.666583 0.203108

H -3.104344 1.611309 -0.264665

H -3.764739 2.105043 1.043236

O -4.139810 2.832464 -2.246457

H -3.914497 3.771944 -2.276539

H -4.208132 2.546731 -3.167160

P1-1LIGAND222+2OH2pq-NiIIt

37

P1-1LIGAND222+2OH2pq-NiIIt SCF Done: -3624.20801688 A.U.

Fe -5.050429 2.061225 -1.539297

P -7.859986 1.658041 -1.293166

O -8.894918 2.847765 -1.172076

O -8.789127 0.352762 -1.189298

C -8.271186 -0.973969 -1.530363

H -7.678856 -0.884502 -2.443527

H -7.598728 -1.284546 -0.723219

C -9.906434 2.896576 -0.108066

H -9.410098 2.661588 0.841503

H -10.638952 2.118244 -0.326931

C -10.506703 4.266255 -0.109760

H -10.962518 4.489807 -1.071917

H -11.284192 4.320822 0.650700

H -9.763034 5.029116 0.110161

C -9.432423 -1.901883 -1.706160

H -10.080192 -1.573361 -2.515891

H -9.066451 -2.897454 -1.952608

H -10.030716 -1.978749 -0.799804

S -6.817638 1.848911 -2.996477

S -6.471525 1.665007 0.234083

C -7.127835 -0.090204 2.575471

H -6.372619 0.579598 2.969564

H -6.820869 -0.890618 1.911687

S -8.643365 0.065940 3.149836

C -9.617129 -1.283439 2.350029

H -9.751205 -2.019777 3.144542

H -8.986507 -1.717309 1.573988

C -10.932107 -0.752172 1.831627

H -11.521598 -1.589963 1.461276

H -11.519370 -0.269094 2.610680

H -10.789798 -0.053446 1.008598

O -4.465593 3.971360 -1.265695

H -4.913331 4.525327 -0.615031

H -4.269287 4.538239 -2.021780

O -3.460417 1.748552 -2.791346

H -2.529317 1.910908 -2.602534

H -3.500063 1.228425 -3.601480

P1-1LIGAND222+2OH2pq-ZnII

7

P1-1LIGAND222+2OH2pq-ZnII SCF Done: -3895.27256071 A.U.

Zn -5.300138 1.998671 -1.753239

P -8.026158 1.542055 -1.495174

O -8.914377 2.367610 -0.448285

O -9.100937 0.528341 -2.087688

C -8.825295 -0.350459 -3.235928

H -8.401284 0.265151 -4.032224

H -8.075936 -1.077804 -2.915028

C -10.067111 3.165143 -0.926739

H -10.740577 2.488190 -1.453078

H -9.680749 3.904090 -1.632050

C -10.701654 3.793461 0.273102

H -10.006039 4.445738 0.798640

H -11.550184 4.398367 -0.041727

H -11.076426 3.039458 0.964968

C -10.115225 -0.993731 -3.635670

H -10.850574 -0.251058 -3.938577

H -9.938466 -1.654196 -4.482972

H -10.531473 -1.589988 -2.826193

S -7.149797 2.667052 -2.941597

S -6.581358 0.640987 -0.345977

C -8.204322 0.888892 2.229612

H -8.475471 1.740962 1.611651

H -7.246905 0.881293 2.740229

S -9.315489 -0.283011 2.418732

C -8.566546 -1.505683 3.581636

H -7.531241 -1.204783 3.734894

H -8.580590 -2.442932 3.023482

C -9.371333 -1.588233 4.858315

H -8.930188 -2.361242 5.486460

H -9.354291 -0.654056 5.415486

H -10.406871 -1.869935 4.676802

O -4.049557 3.524889 -1.210140

H -3.197578 3.445460 -0.767238

H -4.303267 4.454234 -1.190853

O -3.700766 0.975910 -2.542154

H -3.203613 1.282481 -3.309943

H -3.662731 0.012652 -2.554213

P1-1LIGAND222+2OH2td-CoIIq

37

P1-1LIGAND222+2OH2td-CoIIq SCF Done: -3498.64754159 A.U.

Co -5.118798 2.488396 -1.952732

P -8.071848 1.930715 -1.466672

O -9.288563 2.794957 -1.000958

O -8.443901 0.513138 -2.021335

C -8.712943 -0.835719 -1.461304

H -8.325542 -1.489405 -2.239662

H -8.104261 -0.948077 -0.565706

C -10.709709 2.383882 -0.802181

H -10.731080 1.828449 0.136358

H -10.969713 1.720854 -1.627609

C -11.529403 3.630269 -0.769123

H -11.467881 4.176293 -1.707699

H -12.570993 3.352909 -0.609481

H -11.228304 4.284599 0.045771

C -10.166567 -1.048906 -1.199313

H -10.767134 -0.859517 -2.087041

H -10.317265 -2.092695 -0.921267

H -10.522620 -0.431903 -0.375694

S -7.070904 2.926527 -2.934894

S -6.526910 1.992658 0.017549

C -6.819100 0.634961 1.241204

H -6.143938 0.888153 2.060257

H -6.534485 -0.326942 0.815129

S -8.564524 0.683210 1.729764

C -8.749102 -1.037894 2.365805

H -8.005353 -1.178005 3.149604

H -8.531209 -1.734102 1.552382

C -10.152168 -1.214134 2.897490

H -10.268133 -2.227999 3.277181

H -10.360498 -0.529751 3.717645

H -10.905933 -1.064391 2.125072

O -4.062838 3.983810 -1.117688

H -4.288258 4.401953 -0.277255

H -3.468210 4.587945 -1.580401

O -3.833165 1.017512 -2.440439

H -3.397766 0.392606 -1.849189

H -3.471846 0.869701 -3.323375

P1-1LIGAND222+2OH2td-MnIIs

37

P1-1LIGAND222+2OH2td-MnIIs SCF Done: -3266.95235035 A.U.

Mn -5.220489 1.954109 -2.013706

P -8.032548 1.576502 -1.448991

O -8.826324 2.613736 -0.524325

O -9.158436 0.481905 -1.737562

C -9.014737 -0.572039 -2.752448

H -8.739673 -0.091754 -3.693830

H -8.196153 -1.224053 -2.437898

C -10.056552 3.265008 -1.037076

H -10.080419 3.166369 -2.124211

H -9.923152 4.317945 -0.796250

C -11.259603 2.662315 -0.380314

H -11.218361 2.778358 0.702700

H -12.156953 3.172632 -0.727838

H -11.357462 1.604770 -0.623097

C -10.322995 -1.293282 -2.838185

H -11.125642 -0.620198 -3.133935

H -10.252944 -2.079828 -3.587518

H -10.586157 -1.756471 -1.888919

S -7.283141 2.360614 -3.153511

S -6.514966 0.882908 -0.263532

C -8.089066 1.348364 2.249983

H -8.224210 2.251086 1.663043

H -7.144888 1.173754 2.754491

S -9.381670 0.372241 2.425444

C -8.827338 -0.989954 3.541824

H -7.752136 -0.876469 3.670583

H -9.016746 -1.896838 2.966138

C -9.600142 -0.965525 4.840242

H -9.286139 -1.819700 5.438721

H -9.405554 -0.063538 5.416443

H -10.673521 -1.056336 4.684338

O -3.996675 3.699493 -1.872515

H -4.289123 4.592239 -2.090971

H -3.112388 3.785537 -1.498620

O -3.597126 0.805546 -2.785161

H -3.074406 1.020188 -3.566792

H -3.403029 -0.115572 -2.576557

P1-1LIGAND222+2OH2td-NiIIt

37

P1-1LIGAND222+2OH2td-NiIIt SCF Done: -3624.18320689 A.U.

Ni -4.965112 2.357493 -1.503213

P -7.913586 2.088169 -1.402159

O -9.222646 2.867231 -1.056510

O -8.124491 0.628348 -1.954991

C -8.421696 -0.713246 -1.399404

H -7.953020 -1.370808 -2.128977

H -7.889724 -0.805502 -0.454023

C -10.622590 2.379585 -0.893671

H -10.644820 1.826651 0.046816

H -10.821197 1.702500 -1.724506

C -11.507098 3.581527 -0.890079

H -11.442473 4.129184 -1.827452

H -12.537630 3.250532 -0.764357

H -11.266782 4.251063 -0.067473

C -9.885927 -0.969802 -1.262520

H -10.414649 -0.791735 -2.197232

H -10.028984 -2.019556 -1.003706

H -10.329402 -0.370810 -0.468917

S -6.800156 3.126641 -2.701021

S -6.543844 2.208307 0.269087

C -6.820712 0.801587 1.439734

H -6.201709 1.074890 2.295811

H -6.462007 -0.135423 1.012789

S -8.583805 0.730338 1.850548

C -8.722540 -1.043917 2.335504

H -8.015806 -1.210801 3.148209

H -8.425490 -1.664036 1.486487

C -10.142503 -1.323510 2.768162

H -10.226244 -2.367832 3.065120

H -10.431280 -0.715339 3.623063

H -10.858935 -1.151163 1.965659

O -4.036743 4.105572 -1.155420

H -3.366144 4.324561 -0.497135

H -4.388741 4.939907 -1.490325

O -4.843863 0.479856 -2.270471

H -4.054029 -0.075870 -2.273756

H -5.284488 0.339468 -3.119483

P1-1LIGAND222+2OH2pq-ZnII

37

P1-1LIGAND222+2OH2pq-ZnII SCF Done: -3895.27256071 A.U.

Zn -5.300138 1.998671 -1.753239

P -8.026158 1.542055 -1.495174

O -8.914377 2.367610 -0.448285

O -9.100937 0.528341 -2.087688

C -8.825295 -0.350459 -3.235928

H -8.401284 0.265151 -4.032224

H -8.075936 -1.077804 -2.915028

C -10.067111 3.165143 -0.926739

H -10.740577 2.488190 -1.453078

H -9.680749 3.904090 -1.632050

C -10.701654 3.793461 0.273102

H -10.006039 4.445738 0.798640

H -11.550184 4.398367 -0.041727

H -11.076426 3.039458 0.964968

C -10.115225 -0.993731 -3.635670

H -10.850574 -0.251058 -3.938577

H -9.938466 -1.654196 -4.482972

H -10.531473 -1.589988 -2.826193

S -7.149797 2.667052 -2.941597

S -6.581358 0.640987 -0.345977

C -8.204322 0.888892 2.229612

H -8.475471 1.740962 1.611651

H -7.246905 0.881293 2.740229

S -9.315489 -0.283011 2.418732

C -8.566546 -1.505683 3.581636

H -7.531241 -1.204783 3.734894

H -8.580590 -2.442932 3.023482

C -9.371333 -1.588233 4.858315

H -8.930188 -2.361242 5.486460

H -9.354291 -0.654056 5.415486

H -10.406871 -1.869935 4.676802

O -4.049557 3.524889 -1.210140

H -3.197578 3.445460 -0.767238

H -4.303267 4.454234 -1.190853

O -3.700766 0.975910 -2.542154

H -3.203613 1.282481 -3.309943

H -3.662731 0.012652 -2.554213

P1-1LIGAND2+2OH2pq-MnIId

37

P1-1LIGAND2+2OH2pq-MnIId SCF Done: -3266.84983362 A.U.

Mn -5.774193 2.027475 -1.223852

P -8.653714 1.455812 -1.563263

O -10.153636 1.879495 -1.766846

O -8.690795 -0.085006 -1.248981

C -9.755654 -1.034002 -1.734396

H -10.242236 -0.578562 -2.595113

H -9.179398 -1.895453 -2.062359

C -10.601674 3.170314 -2.371186

H -11.670120 3.008348 -2.485278

H -10.146362 3.233115 -3.359901

C -10.272933 4.332888 -1.491085

H -9.198009 4.512503 -1.428874

H -10.721020 5.231725 -1.912735

H -10.674562 4.203690 -0.487498

C -10.695173 -1.329373 -0.613770

H -11.244119 -0.442024 -0.301748

H -11.424174 -2.065604 -0.951444

H -10.175254 -1.754375 0.243446

S -7.329959 2.135744 -2.915131

S -7.650338 2.232278 0.172176

C -7.725527 0.916299 1.485023

H -7.610100 -0.054300 1.004388

H -8.711321 1.003811 1.939792

S -6.388736 1.235082 2.681360

C -6.044441 -0.506712 3.209965

H -5.758516 -1.079380 2.326934

H -6.982288 -0.901373 3.599177

C -4.964825 -0.499233 4.263903

H -4.784163 -1.521820 4.591908

H -4.022207 -0.105347 3.886663

H -5.255550 0.078863 5.138743

O -4.219464 1.905458 -2.515655

H -3.312950 1.650410 -2.305935

H -4.290941 1.929024 -3.477579

O -4.480285 1.902110 0.301882

H -3.621926 2.326456 0.418928

H -4.865785 1.705425 1.198940

P1-1LIGAND2+2OH2pq-MnIIs

37

P1-1LIGAND2+2OH2pq-MnIIs SCF Done: -3266.94614076 A.U.

Mn -5.141792 2.455407 -2.179138

P -7.725227 1.514818 -1.396020

O -8.524710 1.648904 -0.003939

O -8.609591 0.676997 -2.422361

C -8.813960 -0.772850 -2.335383

H -9.259390 -1.016031 -3.297026

H -7.829433 -1.243767 -2.283057

C -9.809806 2.388123 0.008646

H -10.519872 1.698420 0.465291

H -10.133488 2.562468 -1.019642

C -9.649560 3.660335 0.781055

H -8.942892 4.332618 0.297059

H -10.608656 4.172416 0.846310

H -9.305625 3.468801 1.797319

C -9.696677 -1.166046 -1.188011

H -10.650486 -0.641210 -1.220280

H -9.903802 -2.233839 -1.241454

H -9.221089 -0.969545 -0.224984

S -7.373187 3.287161 -2.286838

S -5.986752 0.555016 -0.896031

C -6.225533 -0.099030 1.975642

H -5.162298 0.072885 1.867656

H -6.645740 -1.015742 1.575638

S -7.079315 0.944818 2.890417

C -8.766311 0.198799 2.970402

H -9.031729 -0.036849 1.940299

H -9.388847 1.029633 3.303035

C -8.829858 -0.984183 3.906746

H -9.859006 -1.339047 3.944900

H -8.206667 -1.809698 3.565856

H -8.535431 -0.723370 4.921244

O -4.024350 1.928022 -3.934499

H -3.715708 1.039768 -4.149914

H -4.067658 2.402528 -4.773839

O -3.618557 3.814710 -1.590511

H -2.703892 3.804126 -1.895546

H -3.725451 4.618196 -1.068440

P1-1LIGAND2+2OH2pq-NiIIt

37

P1-1LIGAND2+2OH2pq-NiIIt SCF Done: -3624.21098723 A.U.

Ni -4.933843 2.306735 -1.718797

P -7.553399 1.566410 -1.221310

O -8.550722 1.586028 0.038021

O -8.277710 0.870805 -2.458546

C -8.630252 -0.558926 -2.499989

H -8.833709 -0.727809 -3.554733

H -7.738994 -1.128090 -2.224221

C -9.818819 2.353795 -0.064600

H -10.591029 1.642602 0.227824

H -9.987587 2.618524 -1.110927

C -9.756555 3.554646 0.826228

H -8.962425 4.236143 0.525022

H -10.700203 4.096006 0.772937

H -9.598469 3.273877 1.867106

C -9.811106 -0.887906 -1.636650

H -10.670629 -0.267746 -1.885537

H -10.095644 -1.926073 -1.801759

H -9.583597 -0.771901 -0.576708

S -6.960311 3.382445 -1.853012

S -5.905919 0.523044 -0.603194

C -6.138273 0.344030 2.359005

H -5.167143 0.823191 2.337778

H -6.210872 -0.697069 2.064311

S -7.385357 1.172534 2.999140

C -8.787709 -0.029898 2.956317

H -8.827676 -0.395970 1.929893

H -9.658425 0.603382 3.127304

C -8.653847 -1.119467 3.992834

H -9.534598 -1.758049 3.938880

H -7.780783 -1.746639 3.819478

H -8.599256 -0.720431 5.003611

O -4.512837 1.621892 -3.602170

H -4.287297 0.697755 -3.765256

H -5.127580 1.875816 -4.302739

O -3.433050 3.645281 -1.962490

H -2.910410 3.671563 -2.772762

H -3.316314 4.493231 -1.519685

P1-1LIGAND2+2OH2pq-ZnII

37

P1-1LIGAND2+2OH2pq-ZnII SCF Done: -3895.26559380 A.U.

Zn -5.227407 2.442181 -2.129047

P -7.716836 1.517264 -1.430925

O -8.511708 1.652202 -0.037759

O -8.609646 0.694377 -2.459130

C -8.818945 -0.756953 -2.386299

H -9.289373 -0.984365 -3.339764

H -7.834379 -1.230171 -2.365875

C -9.795255 2.396097 -0.021276

H -10.507421 1.707501 0.433653

H -10.119284 2.573913 -1.048978

C -9.629285 3.665582 0.754222

H -8.916052 4.334210 0.274796

H -10.585302 4.183824 0.816047

H -9.292000 3.470351 1.771966

C -9.672056 -1.164294 -1.221863

H -10.625379 -0.637665 -1.221451

H -9.883211 -2.230690 -1.286740

H -9.170438 -0.985370 -0.268569

S -7.344076 3.298863 -2.317785

S -5.974747 0.548724 -0.939829

C -6.180387 -0.045129 1.967184

H -5.120458 0.143458 1.851355

H -6.588753 -0.968866 1.570699

S -7.045500 0.991148 2.877272

C -8.725472 0.228510 2.963584

H -8.998290 0.003808 1.932831

H -9.350655 1.051120 3.311654

C -8.772294 -0.965890 3.886341

H -9.798879 -1.327671 3.929081

H -8.147411 -1.783669 3.530261

H -8.470895 -0.715117 4.901304

O -4.087169 1.947391 -3.782548

H -3.857510 1.031129 -3.979083

H -4.222645 2.379044 -4.635296

O -3.722598 3.640049 -1.449228

H -2.839609 3.654840 -1.836103

H -3.841022 4.464026 -0.964100

P1-1LIGAND2+2OH2td-CoIIq

37

P1-1LIGAND2+2OH2td-CoIIq SCF Done: -3498.67307157 A.U.

Co -5.689964 2.222209 -0.936386

P -8.396011 1.344866 -1.299619

O -9.336224 2.591208 -1.052844

O -9.212866 0.036063 -1.612755

C -10.550207 -0.273475 -1.008408

H -10.338861 -0.856388 -0.111904

H -11.011217 0.671264 -0.715192

C -10.123266 3.196904 -2.179289

H -10.554570 2.376658 -2.756924

H -9.405052 3.729211 -2.803648

C -11.157347 4.085220 -1.573602

H -10.706694 4.880881 -0.984490

H -11.732416 4.546913 -2.375400

H -11.848999 3.527876 -0.944916

C -11.340544 -1.028387 -2.024608

H -10.837229 -1.945980 -2.320406

H -12.301563 -1.298104 -1.587473

H -11.532932 -0.429808 -2.912878

S -6.956988 1.562403 -2.691149

S -7.423359 1.268761 0.593291

C -6.968440 -0.582433 0.636509

H -6.902351 -0.935193 -0.392384

H -7.810144 -1.058456 1.135598

S -5.428315 -0.962437 1.457635

C -5.823004 -0.566031 3.217670

H -6.708341 -1.150090 3.469252

H -6.072125 0.494260 3.283854

C -4.645609 -0.924690 4.091433

H -4.895911 -0.714919 5.129727

H -4.396607 -1.981842 4.020684

H -3.758340 -0.343400 3.845575

O -5.680164 4.161529 -0.401369

H -5.246259 4.880985 -0.876688

H -5.998521 4.524562 0.433694

O -4.122825 1.292167 -0.199197

H -4.277373 0.548352 0.442991

H -3.225324 1.232874 -0.549218

P1-1LIGAND2+2OH2td-CuIId

37

P1-1LIGAND2+2OH2td-CuIId SCF Done: -3756.37550647 A.U.

Cu -5.611101 2.256961 -0.888010

P -8.488124 1.557983 -1.210111

O -9.625313 2.531411 -0.728648

O -9.060616 0.158686 -1.646000

C -10.351103 -0.414365 -1.135285

H -10.057483 -1.369497 -0.703715

H -10.726048 0.240934 -0.347249

C -10.462512 3.337650 -1.687950

H -10.512031 2.791617 -2.632463

H -9.910751 4.264155 -1.839824

C -11.799628 3.532847 -1.057470

H -11.718341 4.041164 -0.099263

H -12.406356 4.154097 -1.715457

H -12.321747 2.587792 -0.916467

C -11.298696 -0.559014 -2.279621

H -10.878039 -1.175805 -3.070570

H -12.206278 -1.045310 -1.922429

H -11.583668 0.405391 -2.697743

S -7.165803 2.218428 -2.558938

S -7.316142 1.391244 0.579114

C -6.876392 -0.463928 0.623187

H -6.740106 -0.799484 -0.403875

H -7.750737 -0.945941 1.057879

S -5.397252 -0.790261 1.566899

C -6.036095 -0.681615 3.297523

H -6.816415 -1.437927 3.383649

H -6.484155 0.304504 3.430480

C -4.906872 -0.924121 4.268237

H -5.298551 -0.892974 5.283450

H -4.449988 -1.901420 4.123793

H -4.130878 -0.163923 4.194270

O -4.177694 3.339662 -1.915467

H -3.922490 3.118196 -2.820036

H -4.161371 4.304006 -1.864249

O -4.208626 1.936939 0.481566

H -4.280399 1.111394 1.011492

H -3.278163 2.104063 0.286470

P1-1LIGAND2+2OH2td-FeIIId

37

P1-1LIGAND2+2OH2td-FeIIId SCF Done: -3379.06482478 A.U.

Fe -5.833303 2.177097 -1.498862

P -8.729374 1.472007 -0.802774

O -9.981227 2.194410 -0.237075

O -8.984294 -0.068334 -0.734371

C -10.311354 -0.717188 -1.169643

H -10.088604 -1.767954 -1.007623

H -11.045160 -0.373872 -0.443282

C -10.407926 3.649866 -0.418867

H -9.512392 4.214550 -0.681170

H -10.731814 3.916462 0.583787

C -11.483466 3.711321 -1.446115

H -12.332746 3.085046 -1.180762

H -11.841782 4.740431 -1.496979

H -11.124164 3.443253 -2.439435

C -10.639772 -0.376692 -2.581009

H -9.859571 -0.684736 -3.274721

H -11.549699 -0.911517 -2.856907

H -10.855675 0.686303 -2.711696

S -7.851887 2.271389 -2.449071

S -6.921854 1.647523 0.455858

C -6.749732 -0.129877 1.075331

H -6.523227 -0.778771 0.227478

H -7.707411 -0.410240 1.512356

S -5.413015 -0.287115 2.282318

C -6.221542 -1.013653 3.742439

H -6.774527 -1.888043 3.370460

H -6.985753 -0.273545 4.029356

C -5.252095 -1.331986 4.845451

H -5.829490 -1.733526 5.678393

H -4.527182 -2.091434 4.556832

H -4.721394 -0.453690 5.208861

O -4.879764 2.751749 -3.213236

H -4.873261 2.201382 -4.009356

H -4.895665 3.666633 -3.530475

O -4.006177 2.402935 -0.741565

H -3.683502 2.558713 0.154753

H -3.286162 2.624633 -1.351512

P1-1LIGAND2+2OH2td-FeIIIs

37

P1-1LIGAND2+2OH2td-FeIIIs SCF Done: -3379.08087253 A.U.

Fe -5.833303 2.177097 -1.498862

P -8.729374 1.472007 -0.802774

O -9.981227 2.194410 -0.237075

O -8.984294 -0.068334 -0.734371

C -10.311354 -0.717188 -1.169643

H -10.088604 -1.767954 -1.007623

H -11.045160 -0.373872 -0.443282

C -10.407926 3.649866 -0.418867

H -9.512392 4.214550 -0.681170

H -10.731814 3.916462 0.583787

C -11.483466 3.711321 -1.446115

H -12.332746 3.085046 -1.180762

H -11.841782 4.740431 -1.496979

H -11.124164 3.443253 -2.439435

C -10.639772 -0.376692 -2.581009

H -9.859571 -0.684736 -3.274721

H -11.549699 -0.911517 -2.856907

H -10.855675 0.686303 -2.711696

S -7.851887 2.271389 -2.449071

S -6.921854 1.647523 0.455858

C -6.749732 -0.129877 1.075331

H -6.523227 -0.778771 0.227478

H -7.707411 -0.410240 1.512356

S -5.413015 -0.287115 2.282318

C -6.221542 -1.013653 3.742439

H -6.774527 -1.888043 3.370460

H -6.985753 -0.273545 4.029356

C -5.252095 -1.331986 4.845451

H -5.829490 -1.733526 5.678393

H -4.527182 -2.091434 4.556832

H -4.721394 -0.453690 5.208861

O -4.879764 2.751749 -3.213236

H -4.873261 2.201382 -4.009356

H -4.895665 3.666633 -3.530475

O -4.006177 2.402935 -0.741565

H -3.683502 2.558713 0.154753

H -3.286162 2.624633 -1.351512

P1-1LIGAND2+2OH2td-MnIId

37

P1-1LIGAND2+2OH2td-MnIId SCF Done: -3266.85103215 A.U.

Mn -5.587956 2.191900 -0.923530

P -8.542122 1.683411 -1.170122

O -9.799253 2.449318 -0.613867

O -8.937401 0.277044 -1.763230

C -10.232956 -0.438801 -1.521885

H -9.969466 -1.281147 -0.883543

H -10.891535 0.233464 -0.970575

C -10.693106 3.298251 -1.475191

H -10.952691 2.707591 -2.355921

H -10.093102 4.155197 -1.781744

C -11.878802 3.668618 -0.650724

H -11.589177 4.228760 0.235332

H -12.532352 4.301970 -1.249506

H -12.449028 2.792496 -0.348507

C -10.783822 -0.858118 -2.844341

H -10.088056 -1.498197 -3.381994

H -11.698926 -1.425478 -2.676175

H -11.031583 -0.003178 -3.470712

S -7.279971 2.657103 -2.394840

S -7.254620 1.434926 0.525783

C -7.114495 -0.442829 0.826233

H -7.078164 -0.929760 -0.146221

H -8.025078 -0.716110 1.356896

S -5.649575 -0.853486 1.758042

C -6.133850 -0.350381 3.469692

H -7.015276 -0.939795 3.721418

H -6.409182 0.704674 3.449813

C -4.988277 -0.619199 4.414292

H -5.293244 -0.357652 5.425987

H -4.704435 -1.669856 4.419706

H -4.108784 -0.023830 4.174267

O -4.223962 3.311315 -1.941202

H -4.413694 3.845972 -2.721683

H -3.350057 3.575542 -1.628771

O -4.089553 1.298369 0.077791

H -4.322161 0.627905 0.765737

H -3.210251 1.087525 -0.259540

P1-1LIGAND2+2OH2td-MnIIs

37

P1-1LIGAND2+2OH2td-MnIIs SCF Done: -3266.94619774 A.U.

Mn -5.588108 2.221744 -0.884940

P -8.430250 1.372228 -1.316791

O -9.463536 2.532649 -1.032582

O -9.152600 0.000693 -1.606589

C -10.439259 -0.392434 -0.949927

H -10.203519 -1.329010 -0.447159

H -10.690467 0.363947 -0.203336

C -10.258057 3.158157 -2.141730

H -10.346670 2.429062 -2.950013

H -9.661228 3.998943 -2.492661

C -11.578098 3.561373 -1.574944

H -11.460854 4.262737 -0.751757

H -12.158935 4.052967 -2.354587

H -12.147137 2.700961 -1.226693

C -11.487994 -0.550262 -2.002357

H -11.188731 -1.276713 -2.754478

H -12.404091 -0.909825 -1.534484

H -11.717000 0.393562 -2.495479

S -7.043004 1.678106 -2.720843

S -7.484221 1.310162 0.612091

C -7.017383 -0.540987 0.673649

H -6.952367 -0.905401 -0.351241

H -7.861555 -1.009734 1.175609

S -5.484017 -0.913844 1.507991

C -5.891778 -0.495426 3.260226

H -6.773656 -1.083392 3.514620

H -6.151105 0.563354 3.309032

C -4.717621 -0.830904 4.147010

H -4.977626 -0.610869 5.180782

H -4.456968 -1.886152 4.091643

H -3.834525 -0.243299 3.900677

O -4.904014 4.200699 -0.679938

H -5.154386 4.963352 -1.215590

H -4.237046 4.512920 -0.056046

O -3.927801 1.232190 -0.152407

H -4.126803 0.498852 0.483018

H -3.043553 1.093133 -0.512609

P1-1LIGAND2+2OH2td-NiIIt

37

P1-1LIGAND2+2OH2td-NiIIt SCF Done: -3624.21146915 A.U.

Ni -5.681575 2.138774 -1.042496

P -8.455015 1.418854 -1.336518

O -9.454880 2.599898 -1.039660

O -9.195988 0.061097 -1.623576

C -10.525100 -0.318724 -1.037352

H -10.293200 -0.913954 -0.154473

H -11.027275 0.598198 -0.725194

C -10.297025 3.205351 -2.131342

H -10.696122 2.383576 -2.729584

H -9.616855 3.794457 -2.746485

C -11.360373 4.017154 -1.473124

H -10.937191 4.809953 -0.860373

H -11.970375 4.481407 -2.247395

H -12.014062 3.403100 -0.857063

C -11.274543 -1.082167 -2.077690

H -10.730327 -1.970123 -2.390613

H -12.226061 -1.403138 -1.654642

H -11.486505 -0.471580 -2.953203

S -7.069274 1.718517 -2.762127

S -7.369690 1.361802 0.506362

C -6.985503 -0.507632 0.604843

H -6.927512 -0.889733 -0.413761

H -7.849657 -0.934235 1.111163

S -5.466985 -0.898974 1.454183

C -5.906438 -0.532540 3.211351

H -6.781855 -1.140538 3.439516

H -6.181483 0.520861 3.283255

C -4.737344 -0.873103 4.102958

H -5.013378 -0.683514 5.138706

H -4.459993 -1.922676 4.024277

H -3.860747 -0.266004 3.882440

O -5.117547 3.986693 -0.481570

H -5.495842 4.835995 -0.734603

H -4.448311 4.148693 0.193495

O -4.056941 1.459649 -0.064538

H -4.238206 0.687586 0.526016

H -3.206787 1.300547 -0.494020

P1-1LIGAND2+2OH2td-ZnII

37

P1-1LIGAND2+2OH2td-ZnII SCF Done: -3895.26630987 A.U.

Zn -5.762985 2.219599 -0.799615

P -8.468200 1.400169 -1.278856

O -9.477901 2.583062 -1.007430

O -9.208877 0.057322 -1.634582

C -10.543869 -0.328057 -1.070226

H -10.325129 -0.922279 -0.182849

H -11.056560 0.585797 -0.765537

C -10.270952 3.195026 -2.127690

H -10.682653 2.375969 -2.721064

H -9.560115 3.751657 -2.739114

C -11.324558 4.051335 -1.510345

H -10.892234 4.844147 -0.903968

H -11.904551 4.516357 -2.306716

H -12.008080 3.468483 -0.896199

C -11.273468 -1.094850 -2.122482

H -10.719709 -1.979644 -2.427447

H -12.230053 -1.420700 -1.715162

H -11.473796 -0.484460 -3.000857

S -7.024185 1.704223 -2.643425

S -7.551980 1.301318 0.665913

C -7.038356 -0.537981 0.667297

H -6.975831 -0.870779 -0.368528

H -7.863068 -1.043289 1.165332

S -5.483704 -0.896852 1.468621

C -5.872207 -0.539247 3.238863

H -6.745983 -1.142771 3.484760

H -6.138573 0.515317 3.326085

C -4.683529 -0.893985 4.098556

H -4.930989 -0.706330 5.141832

H -4.417855 -1.945563 4.007889

H -3.807417 -0.293691 3.858233

O -5.172307 4.121168 -0.446431

H -5.465082 4.922676 -0.895014

H -4.450399 4.363561 0.145700

O -4.079773 1.400827 -0.111019

H -4.244001 0.624810 0.484781

H -3.287034 1.221186 -0.632142

P1-LIGAND111

30

P1-LIGAND111 SCF Done: -1963.51362605 A.U.

P -1.988749 1.003385 0.524304

O -0.847398 0.981490 1.687490

O -1.972869 -0.561284 0.116767

C -0.766094 -1.213095 -0.316562

H -0.420010 -0.732545 -1.239185

H 0.007352 -1.084229 0.447812

C -0.684564 2.161625 2.512934

H -1.548471 2.231803 3.178372

H -0.674156 3.049093 1.872356

C 0.606393 2.023101 3.262908

H 1.450393 1.991856 2.572946

H 0.746300 2.880498 3.920468

H 0.616855 1.120159 3.871996

C -1.084014 -2.662969 -0.542019

H -1.862707 -2.776598 -1.294143

H -0.195753 -3.192053 -0.885638

H -1.427631 -3.133315 0.377756

S -3.733768 1.670103 0.934430

S -1.076587 2.096005 -1.062987

C 0.711918 1.832224 -0.774502

H 1.160211 1.532616 -1.722660

H 0.850193 1.036772 -0.041728

S 1.481448 3.366634 -0.161313

C 3.192135 2.737686 0.013863

H 3.572948 2.473258 -0.974967

H 3.161893 1.823938 0.612990

C 4.062299 3.784062 0.671129

H 5.085549 3.422419 0.770397

H 4.091208 4.703393 0.086968

H 3.697571 4.033850 1.667738

P1-LIGAND11

30

P1-LIGAND11 SCF Done: -1963.52188499 A.U.

P -1.918273 2.726840 0.163819

O -1.137994 2.581096 1.586130

O -1.690331 1.272871 -0.545320

C -0.349047 0.761607 -0.731058

H 0.367154 1.376578 -0.177998

H -0.345692 -0.233730 -0.281283

C -1.673075 1.658194 2.561363

H -1.670861 0.648727 2.136796

H -2.712691 1.928992 2.767104

C -0.817568 1.746200 3.790936

H -0.830293 2.753997 4.202752

H -1.189155 1.061777 4.552653

H 0.215692 1.481441 3.570617

C -0.020994 0.720645 -2.196921

H -0.016765 1.727680 -2.620392

H 0.963843 0.281012 -2.353869

H -0.751924 0.121181 -2.740047

S -3.786463 3.184505 0.157424

S -0.537769 4.089759 -0.656159

C -1.561231 4.858037 -1.993441

H -1.178843 5.873335 -2.065341

H -2.590820 4.897229 -1.633001

S -1.484820 4.143609 -3.647493

C -2.738956 2.818498 -3.488231

H -3.698188 3.279016 -3.246858

H -2.465313 2.174688 -2.651499

C -2.804035 2.028452 -4.774030

H -3.543105 1.232161 -4.688674

H -3.086278 2.654231 -5.620682

H -1.843157 1.567892 -5.006811

P1-LIGAND1

30

P1-LIGAND1 SCF Done: -1963.51358223 A.U.

P -1.776878 1.016821 0.341915

O -0.518196 1.735264 1.087986

O -1.336956 -0.528470 0.524796

C -0.067082 -1.010664 0.051656

H -0.026585 -0.883669 -1.036556

H 0.734551 -0.411221 0.495730

C -0.612295 3.148006 1.397577

H -1.356739 3.272178 2.187717

H -0.958800 3.687824 0.511080

C 0.750204 3.616138 1.813435

H 1.460161 3.512257 0.992053

H 0.712571 4.669332 2.089397

H 1.119561 3.051901 2.668884

C 0.045997 -2.457403 0.436638

H -0.761708 -3.039806 -0.003384

H 0.993200 -2.865208 0.085446

H 0.001929 -2.576713 1.517786

S -3.556013 1.381031 0.941620

S -1.507658 1.516961 -1.714009

C 0.291382 1.830261 -1.826198

H 0.663463 1.296180 -2.701489

H 0.785039 1.448860 -0.932049

S 0.615367 3.617432 -1.978146

C 2.444634 3.543880 -1.977017

H 2.780126 3.042615 -2.887198

H 2.760502 2.933087 -1.127263

C 3.018587 4.938780 -1.877995

H 4.107786 4.905257 -1.889455

H 2.698130 5.563491 -2.711412

H 2.707803 5.429357 -0.955333

P1-LIGAND2222

30

P1-LIGAND2222 SCF Done: -1963.51508490 A.U.

P -1.774890 0.788736 -0.311308

O -1.128916 1.727865 0.847919

O -1.248128 -0.679694 0.107269

C 0.111489 -1.136846 -0.051478

H 0.067462 -2.180209 0.258930

H 0.364935 -1.127506 -1.116257

C -1.902726 2.725089 1.549363

H -2.503121 2.218660 2.309863

H -2.588031 3.207162 0.848888

C -0.938418 3.707565 2.146869

H -0.368272 4.209281 1.366057

H -1.481094 4.462697 2.715161

H -0.238586 3.215069 2.821224

C 1.114668 -0.361732 0.759814

H 0.844097 -0.353074 1.814570

H 2.096077 -0.825302 0.659596

H 1.192425 0.671983 0.424398

S -3.685832 0.898484 -0.528558

S -0.606304 1.187686 -2.056006

C -1.667700 2.471979 -2.789979

H -2.686723 2.084360 -2.846512

H -1.273815 2.612998 -3.795375

S -1.692779 4.038924 -1.851747

C -3.463577 4.454629 -2.048855

H -3.626262 5.274232 -1.347313

H -4.037308 3.599319 -1.682870

C -3.860443 4.847426 -3.452588

H -4.918720 5.110632 -3.493839

H -3.284080 5.702510 -3.804299

H -3.702964 4.029952 -4.156893

P1-LIGAND222

P -1.850820 1.426414 0.218548

O -0.904203 2.214235 1.281579

O -1.213684 -0.064903 0.102394

C 0.158263 -0.367388 -0.216590

H 0.117309 -1.374825 -0.632124

H 0.511218 0.297439 -1.005731

C -1.062594 1.937813 2.690398

H -0.969168 0.860251 2.863213

H -2.070790 2.234041 2.992548

C -0.001121 2.709588 3.419397

H -0.094287 3.776804 3.223580

H -0.090741 2.548966 4.493286

H 0.995796 2.396124 3.109032

C 1.070713 -0.306132 0.978675

H 0.699469 -0.934798 1.787916

H 2.061540 -0.668254 0.699097

H 1.179299 0.717401 1.335103

S -3.731318 1.339384 0.588465

S -1.538680 2.578589 -1.526555

C 0.231693 2.630204 -2.008979

H 0.238664 3.418032 -2.764109

H 0.515616 1.696931 -2.497022

S 1.431474 2.995217 -0.688465

C 2.916761 2.343035 -1.534642

H 3.111997 2.929904 -2.434156

H 2.704774 1.316210 -1.848299

C 4.097171 2.380985 -0.591067

H 4.988290 1.985052 -1.077910

H 4.319902 3.397997 -0.269364

H 3.908273 1.782518 0.300637

P1-LIGAND22

30

P1-LIGAND22 SCF Done: -1963.51943919 A.U.

P -1.983323 2.776148 0.066773

O -1.190910 2.542906 1.468163

O -1.682583 1.429496 -0.807235

C -0.400870 1.145595 -1.415256

H -0.595398 0.259731 -2.020615

H -0.146152 1.967015 -2.093329

C -1.665429 1.507246 2.356710

H -1.770794 0.572973 1.795288

H -2.656963 1.789132 2.721721

C -0.667885 1.375614 3.469883

H -0.564554 2.313470 4.013485

H -0.990153 0.608128 4.172645

H 0.312228 1.096091 3.084444

C 0.694498 0.899898 -0.413030

H 0.411658 0.120108 0.295004

H 1.598363 0.576042 -0.928823

H 0.932348 1.801713 0.150665

S -3.880599 3.101060 0.132083

S -0.714846 4.314707 -0.601014

C -1.705711 4.942101 -2.029881

H -1.386458 5.976606 -2.130730

H -2.756951 4.930184 -1.736707

S -1.482337 4.140640 -3.636937

C -2.862101 2.932240 -3.616857

H -3.801367 3.485676 -3.580203

H -2.795626 2.330903 -2.711259

C -2.778287 2.060882 -4.848147

H -3.603877 1.349691 -4.861142

H -2.829975 2.646688 -5.766164

H -1.848414 1.491147 -4.868665

P1-LIGAND2

30

P1-LIGAND2 SCF Done: -1963.50873437 A.U.

P -1.748362 1.164710 0.173822

O -0.426985 2.058655 0.516347

O -1.373541 -0.321103 0.730504

C -0.233656 -1.068537 0.272919

H -0.498229 -2.109004 0.462441

H -0.136611 -0.955988 -0.812188

C -0.340631 2.759894 1.774298

H -0.264696 2.028798 2.586341

H -1.262776 3.327759 1.923180

C 0.865367 3.650517 1.703401

H 0.789544 4.317967 0.845016

H 0.944263 4.254181 2.606638

H 1.782534 3.068442 1.606300

C 1.037415 -0.697308 0.994227

H 0.904858 -0.771567 2.073260

H 1.839014 -1.377817 0.706081

H 1.355541 0.318895 0.761766

S -3.448371 1.733757 0.846587

S -1.586023 1.101029 -1.938441

C 0.167851 1.544878 -2.228889

H 0.444723 1.015341 -3.140354

H 0.784761 1.181375 -1.407691

S 0.437693 3.339882 -2.400249

C 2.185665 3.367143 -1.859076

H 2.786120 2.773182 -2.551104

H 2.232925 2.886081 -0.877383

C 2.690798 4.789469 -1.792864

H 3.729301 4.810403 -1.462807

H 2.643435 5.275810 -2.766995

H 2.106143 5.388660 -1.094784

P1-2LIGAND2222+1OH2-CoIIq

64

P1-2LIGAND2222+1OH2-CoIIq SCF Done: -5385.82703471 A.U.

P -1.902576 1.434391 -0.130425

O -1.095046 2.735477 0.253436

O -0.847000 0.254363 -0.029977

C -1.200248 -1.168938 -0.130452

H -2.016926 -1.355455 0.571674

H -1.554803 -1.348530 -1.149797

C 0.323435 2.926898 -0.147273

H 0.414803 2.658442 -1.203479

H 0.911790 2.222477 0.438848

C 0.672831 4.353253 0.127223

H 0.554044 4.592703 1.181637

H 1.714906 4.521418 -0.140847

H 0.060958 5.039907 -0.456105

C 0.023904 -1.965373 0.192497

H 0.370983 -1.759228 1.202665

H -0.211011 -3.026505 0.126593

H 0.829548 -1.754696 -0.507374

S -3.564674 1.250995 0.930305

S -2.397171 1.576114 -2.219063

C -2.692452 3.383885 -2.338334

H -3.023243 3.533823 -3.366178

H -1.764083 3.923680 -2.168303

S -3.999046 3.969594 -1.194363

C -4.989491 5.052360 -2.313767

H -5.883932 5.266183 -1.725338

H -5.295374 4.447760 -3.168664

C -4.253732 6.310662 -2.698927

H -4.901019 6.933482 -3.315669

H -3.960955 6.894212 -1.828427

H -3.360411 6.099637 -3.286011

Co -4.947881 1.670253 -1.049423

P -7.671450 2.073586 -3.463687

O -7.797075 3.655954 -3.580455

O -8.380087 1.512613 -4.765985

C -8.361713 0.092550 -5.162449

H -7.315373 -0.209342 -5.243889

H -8.836842 -0.482683 -4.364066

C -9.080504 4.297072 -3.951830

H -9.870202 3.845373 -3.345768

H -9.264602 4.056664 -4.998365

C -8.926885 5.763791 -3.705330

H -8.121263 6.182737 -4.306079

H -9.847659 6.275435 -3.981004

H -8.731452 5.975567 -2.654295

C -9.096096 -0.024040 -6.459408

H -8.616086 0.562866 -7.239514

H -9.098711 -1.066156 -6.774677

H -10.130163 0.299204 -6.360356

S -5.734864 1.595032 -3.333170

S -8.894856 1.438656 -1.878227

C -8.339626 2.659418 -0.621566

H -9.191118 2.793764 0.043079

H -8.137439 3.614514 -1.104720

S -6.879864 2.250893 0.380204

C -7.434111 0.695916 1.194267

H -6.562157 0.396727 1.777449

H -7.574557 -0.045995 0.407339

C -8.655210 0.874314 2.059195

H -8.861222 -0.055505 2.588245

H -8.520114 1.657024 2.803363

H -9.545644 1.103802 1.474386

O -5.395657 -0.486777 -1.038412

H -5.617219 -0.901016 -1.880562

H -4.919011 -1.141941 -0.516586

P1-2LIGAND2222+1OH2-CoIIqISOMER

64

P1-2LIGAND2222+1OH2-CoIIqCCC SCF Done: -5385.81704982 A.U.

P -2.054010 2.160742 0.312312

O -1.184078 3.393613 0.776310

O -1.484554 0.918485 1.122439

C -2.180238 -0.367234 1.242872

H -3.126550 -0.177621 1.755027

H -2.393855 -0.734305 0.233235

C 0.288750 3.291094 0.960005

H 0.699773 2.789852 0.079553

H 0.450398 2.657273 1.830945

C 0.812267 4.678791 1.136984

H 0.363335 5.164155 2.000806

H 1.888324 4.632417 1.298402

H 0.632098 5.293240 0.256410

C -1.289065 -1.295643 2.005254

H -1.076476 -0.904613 2.997948

H -1.785187 -2.257837 2.121691

H -0.348270 -1.461029 1.484547

S -3.986331 2.530493 0.629399

S -1.521660 1.730771 -1.697092

C -1.790720 3.441525 -2.343936

H -1.156381 3.523689 -3.224741

H -1.431415 4.149222 -1.598860

S -3.483504 3.905009 -2.790412

C -3.581333 3.187015 -4.484627

H -4.653632 3.137042 -4.678101

H -3.216286 2.159064 -4.431300

C -2.850884 4.010217 -5.514985

H -2.957336 3.549444 -6.496636

H -3.244130 5.023258 -5.571200

H -1.782220 4.076215 -5.311226

Co -5.232163 2.564270 -1.424635

P -8.411546 3.119708 -3.083693

O -9.606035 3.937919 -2.452394

O -8.697175 3.081746 -4.645728

C -7.686148 2.710988 -5.643553

H -6.940872 3.509203 -5.651036

H -7.202995 1.784267 -5.317079

C -11.015209 3.715766 -2.870737

H -11.172849 2.638001 -2.961184

H -11.119510 4.168949 -3.855764

C -11.891843 4.345946 -1.838243

H -11.687778 5.410119 -1.742053

H -12.932550 4.228953 -2.137339

H -11.768857 3.875861 -0.863879

C -8.380736 2.559435 -6.959505

H -8.869005 3.486403 -7.252909

H -7.649817 2.307182 -7.726188

H -9.125035 1.766647 -6.927447

S -6.693027 4.022328 -2.588146

S -8.592487 1.105456 -2.504302

C -8.590627 1.412391 -0.694034

H -9.154091 0.594745 -0.247708

H -9.118848 2.339992 -0.478300

S -6.966290 1.505005 0.107869

C -6.522733 -0.281104 0.144444

H -5.438700 -0.276469 0.277033

H -6.741133 -0.689024 -0.847210

C -7.223384 -1.045617 1.239705

H -6.918920 -2.091403 1.214970

H -6.982956 -0.644365 2.222120

H -8.306656 -1.026699 1.125556

O -4.626998 0.860118 -2.446862

H -3.658623 0.748517 -2.432817

H -5.023941 -0.016322 -2.386161

P1-2LIGAND2222+1OH2-CuIId

64

P1-2LIGAND2222+1OH2-CuIId SCF Done: -5643.52548686 A.U.

P -2.006603 1.347034 -0.275143

O -1.126778 2.450445 0.431382

O -1.163338 0.005814 -0.159869

C -1.679107 -1.313361 -0.546563

H -2.576912 -1.499651 0.047856

H -1.954983 -1.269186 -1.604996

C 0.341540 2.538946 0.229507

H 0.547952 2.394773 -0.834848

H 0.782344 1.709779 0.781555

C 0.775551 3.877143 0.733500

H 0.538810 3.996019 1.788546

H 1.854178 3.970675 0.617357

H 0.304955 4.687981 0.178785

C -0.600956 -2.317818 -0.289442

H -0.321344 -2.331779 0.761539

H -0.962868 -3.309716 -0.555485

H 0.284182 -2.110137 -0.886537

S -3.823414 1.264551 0.541106

S -2.074201 1.772322 -2.354876

C -2.259743 3.589589 -2.283410

H -2.492303 3.883665 -3.306883

H -1.310584 4.031500 -1.985759

S -3.573020 4.201474 -1.160988

C -4.153525 5.673149 -2.107341

H -5.019978 6.007125 -1.535103

H -4.514559 5.332213 -3.078792

C -3.094973 6.743965 -2.211062

H -3.515324 7.613411 -2.715745

H -2.743850 7.064164 -1.231823

H -2.235740 6.419462 -2.797090

Cu -5.163123 2.249605 -1.206365

P -7.981472 2.473972 -3.447004

O -8.798915 3.772478 -3.833366

O -8.267482 1.463010 -4.640490

C -7.583709 0.177673 -4.806569

H -6.530957 0.398871 -5.003182

H -7.659812 -0.375474 -3.866450

C -10.192837 3.684989 -4.336384

H -10.739986 2.999162 -3.684438

H -10.139883 3.252227 -5.334892

C -10.759790 5.067251 -4.326506

H -10.180699 5.737034 -4.958512

H -11.776810 5.035489 -4.714751

H -10.797942 5.479183 -3.319719

C -8.236518 -0.538435 -5.946943

H -8.166452 0.039464 -6.865943

H -7.737130 -1.492335 -6.109185

H -9.285237 -0.738957 -5.738620

S -6.082376 3.071304 -3.199708

S -8.857976 1.541374 -1.790507

C -8.507754 2.847715 -0.549852

H -9.352141 2.842945 0.137179

H -8.470875 3.826193 -1.026220

S -6.979357 2.661466 0.414651

C -7.282718 1.038936 1.233416

H -6.364638 0.865984 1.796154

H -7.345265 0.281944 0.448575

C -8.494260 1.036071 2.131465

H -8.540370 0.089157 2.668263

H -8.455991 1.834820 2.869837

H -9.425458 1.124358 1.572754

O -5.417875 0.190860 -2.007291

H -4.991144 0.064144 -2.863213

H -5.051160 -0.492768 -1.431752

P1-2LIGAND2222+1OH2-FeIIIs

64

P1-2LIGAND2222+1OH2-FeIIIs SCF Done: -5266.31392387 A.U.

P -2.485781 1.827544 0.238063

O -1.094984 2.549588 0.319391

O -2.766133 0.816776 1.402955

C -1.681555 -0.034868 2.038959

H -2.109208 -1.034264 2.008552

H -0.805832 0.001718 1.389414

C -0.833908 3.840010 1.067624

H 0.068299 3.612341 1.630373

H -1.659732 3.995159 1.763563

C -0.666093 4.944718 0.081582

H -1.582349 5.129415 -0.479478

H -0.419474 5.860332 0.619032

H 0.150684 4.743016 -0.609601

C -1.422242 0.462944 3.419397

H -2.324871 0.455541 4.025941

H -0.697816 -0.199315 3.893775

H -0.998194 1.465925 3.421083

S -4.092251 3.040306 0.054840

S -2.298512 0.653765 -1.525451

C -1.474714 1.866774 -2.641846

H -1.125841 1.288641 -3.496867

H -0.616981 2.287943 -2.121396

S -2.596219 3.192197 -3.208427

C -2.659559 2.880652 -5.036421

H -3.490899 3.508649 -5.359515

H -2.948110 1.838025 -5.173175

C -1.365490 3.241272 -5.718735

H -1.478662 3.075482 -6.790092

H -1.103320 4.286563 -5.570892

H -0.530867 2.623815 -5.388135

Fe -4.702447 2.187983 -2.067611

P -7.873362 3.263109 -3.360204

O -8.574160 3.587581 -1.991018

O -8.504695 4.043270 -4.550889

C -9.186928 3.555947 -5.800254

H -8.960079 4.349205 -6.507545

H -8.674424 2.645715 -6.115031

C -10.057137 3.835132 -1.858949

H -10.567984 2.989482 -2.324957

H -10.261428 4.738573 -2.432146

C -10.350136 3.976165 -0.404703

H -9.808927 4.812914 0.030934

H -11.415824 4.170102 -0.284646

H -10.112799 3.069103 0.148953

C -10.645398 3.368299 -5.555943

H -11.113028 4.284988 -5.201817

H -11.122359 3.102448 -6.499649

H -10.844707 2.558861 -4.853061

S -5.924100 3.852078 -3.158372

S -8.034439 1.179408 -3.746872

C -8.084852 0.574968 -2.014023

H -8.046061 -0.510269 -2.103084

H -9.037768 0.856798 -1.572298

S -6.760470 1.137470 -0.868355

C -6.144867 -0.474470 -0.189029

H -5.274983 -0.171489 0.398800

H -5.798859 -1.080664 -1.028510

C -7.178295 -1.174764 0.656385

H -6.744321 -2.089514 1.059808

H -7.501069 -0.559751 1.493663

H -8.056598 -1.468163 0.083075

O -4.823867 0.674517 -3.556372

H -4.423831 -0.203552 -3.589388

H -5.671883 0.631117 -4.037591

P1-2LIGAND2222+1OH2-MnIIs

64

P1-2LIGAND2222+1OH2-MnIIs SCF Done: -5154.11068753 A.U.

P -2.297303 1.247623 -0.775145

O -0.758494 1.097801 -0.421464

O -2.850625 -0.153626 -1.338632

C -1.968683 -1.077522 -2.104381

H -2.592200 -1.411857 -2.931814

H -1.135395 -0.502990 -2.511821

C -0.249499 0.876730 0.956216

H 0.545908 0.147539 0.816616

H -1.041738 0.421273 1.553053

C 0.239729 2.168148 1.530068

H -0.575738 2.877228 1.673544

H 0.687281 1.982424 2.505584

H 1.001308 2.618000 0.895375

C -1.521258 -2.198140 -1.220880

H -2.368151 -2.737902 -0.801688

H -0.930132 -2.902517 -1.804881

H -0.896273 -1.839415 -0.404624

S -3.458293 1.931566 0.658682

S -2.311404 2.461433 -2.496669

C -1.952230 4.066547 -1.655642

H -1.346579 4.653006 -2.343698

H -1.364900 3.884088 -0.757896

S -3.430644 5.022645 -1.193682

C -3.744544 5.907503 -2.786077

H -4.761467 6.285961 -2.679486

H -3.754689 5.159904 -3.582174

C -2.757835 7.018324 -3.040702

H -3.006616 7.513458 -3.978962

H -2.780111 7.765313 -2.250175

H -1.734825 6.654730 -3.135482

Mn -5.071446 2.950449 -1.113606

P -7.998682 2.635005 -3.013171

O -9.106375 3.232499 -3.979318

O -7.672365 1.118690 -3.491989

C -7.383691 0.886116 -4.925508

H -8.303387 1.076559 -5.480650

H -6.633029 1.615651 -5.239512

C -10.450837 2.622684 -4.091995

H -10.989183 2.877604 -3.177813

H -10.335777 1.536231 -4.132366

C -11.092701 3.179525 -5.321949

H -10.535644 2.914704 -6.218720

H -12.097615 2.770764 -5.415821

H -11.172293 4.262930 -5.268348

C -6.912396 -0.525984 -5.069336

H -7.653202 -1.232544 -4.700186

H -6.738471 -0.742914 -6.121963

H -5.972841 -0.691873 -4.541549

S -6.462447 3.879445 -3.037760

S -8.772559 2.139733 -1.131161

C -8.371860 3.679998 -0.215095

H -9.180165 3.820179 0.499492

H -8.354615 4.535848 -0.886719

S -6.787260 3.681515 0.683096

C -6.992082 2.215021 1.784781

H -6.043302 2.172700 2.320109

H -7.053098 1.330411 1.147495

C -8.161896 2.337009 2.727276

H -8.159127 1.490166 3.412440

H -8.112944 3.246876 3.322824

H -9.119207 2.316091 2.207029

O -5.511711 0.890225 -1.649857

H -4.880973 0.165950 -1.517400

H -6.194124 0.608720 -2.282101

P1-2LIGAND2222+1OH2-NiIIt

64

P1-2LIGAND2222+1OH2-NiIIt SCF Done: -5511.36068196 A.U.

P -2.104614 1.716053 0.381362

O -1.260502 3.036104 0.597414

O -1.444561 0.628107 1.327934

C -2.126925 -0.576200 1.818512

H -1.437009 -0.959610 2.565794

H -3.041671 -0.256628 2.320417

C 0.213807 3.011320 0.753512

H 0.626019 2.338659 -0.004091

H 0.419330 2.588440 1.736092

C 0.696583 4.418790 0.609351

H 0.258276 5.064892 1.366760

H 1.778377 4.441391 0.731286

H 0.460058 4.827345 -0.372882

C -2.386440 -1.558765 0.716336

H -1.471257 -1.810644 0.181719

H -2.788447 -2.479803 1.135761

H -3.123458 -1.178756 0.004234

S -4.038734 2.012025 0.684251

S -1.653935 1.053644 -1.599877

C -1.658466 2.672082 -2.438853

H -1.637924 2.443152 -3.503934

H -0.752621 3.210936 -2.170334

S -3.090606 3.772480 -2.094120

C -3.160565 4.687601 -3.694347

H -4.047462 5.313481 -3.590261

H -3.353423 3.966119 -4.489270

C -1.920356 5.512973 -3.934166

H -2.051305 6.099203 -4.843134

H -1.733296 6.206553 -3.116103

H -1.032357 4.898089 -4.076476

Ni -5.110066 2.414176 -1.464277

P -7.962109 2.590085 -3.631773

O -9.095554 3.231683 -2.736788

O -8.506451 2.671915 -5.116503

C -7.732274 2.278840 -6.306664

H -6.775994 2.804500 -6.265668

H -7.550399 1.203221 -6.239925

C -10.525031 2.884521 -2.918702

H -10.592184 1.811971 -3.122749

H -10.866980 3.423837 -3.801179

C -11.241007 3.286777 -1.669670

H -11.149479 4.355685 -1.489868

H -12.299406 3.050939 -1.769641

H -10.856076 2.756075 -0.799250

C -8.540521 2.643482 -7.510752

H -8.724813 3.714833 -7.552512

H -7.992683 2.359205 -8.407748

H -9.494873 2.121368 -7.520304

S -6.204530 3.470780 -3.347071

S -7.872533 0.515005 -3.174434

C -8.123487 0.583708 -1.366493

H -7.755631 -0.375106 -1.002211

H -9.190370 0.660638 -1.167618

S -7.294094 1.922690 -0.414630

C -7.135689 1.037142 1.195988

H -6.602786 1.749533 1.825941

H -6.483535 0.175773 1.039794

C -8.474320 0.670610 1.788112

H -8.321623 0.243301 2.778470

H -9.117719 1.541923 1.899927

H -9.002893 -0.077217 1.198242

O -4.798172 0.526489 -2.186905

H -3.859041 0.298072 -2.297202

H -5.276897 0.287771 -2.995014

P1-2LIGAND2222+1OH2-ZnII

64

P1-2LIGAND2222+1OH2-ZnII SCF Done: -5782.41109104 A.U.

P -2.030336 2.249814 0.258550

O -0.994839 3.340756 0.740325

O -1.576076 0.903819 0.973050

C -2.450700 -0.254439 1.174164

H -3.274022 0.070866 1.814981

H -2.857670 -0.549296 0.201084

C 0.454616 3.041235 0.870904

H 0.779906 2.566104 -0.058218

H 0.554875 2.323815 1.684695

C 1.151916 4.335021 1.138634

H 0.784904 4.800101 2.050881

H 2.216916 4.144245 1.262837

H 1.030380 5.035664 0.314323

C -1.633700 -1.339512 1.801037

H -1.228808 -1.019581 2.758688

H -2.264025 -2.209877 1.976740

H -0.811302 -1.642217 1.156368

S -3.862940 2.896017 0.673227

S -1.616174 1.859387 -1.785311

C -1.849654 3.595105 -2.381703

H -1.211619 3.693293 -3.258194

H -1.481746 4.279313 -1.618259

S -3.536922 4.089198 -2.809447

C -3.703885 3.266478 -4.446572

H -4.782273 3.151873 -4.568572

H -3.276569 2.263742 -4.354411

C -3.084134 4.050933 -5.575894

H -3.225701 3.520827 -6.517643

H -3.534192 5.037186 -5.670214

H -2.011293 4.184872 -5.442801

Zn -5.283480 2.726837 -1.292502

P -8.365952 3.070517 -3.058468

O -9.688522 3.723596 -2.491952

O -8.587506 2.980187 -4.630685

C -7.508632 2.840551 -5.609924

H -6.865230 3.718755 -5.514289

H -6.926955 1.948265 -5.352608

C -11.031453 3.324913 -2.987579

H -11.060161 2.232942 -3.030311

H -11.118982 3.717676 -4.000004

C -12.040633 3.896806 -2.046029

H -11.962211 4.980351 -1.992951

H -13.039104 3.648743 -2.403310

H -11.932764 3.487855 -1.042761

C -8.134024 2.733527 -6.964999

H -8.721311 3.619933 -7.194373

H -7.352428 2.644127 -7.717768

H -8.776541 1.858844 -7.038132

S -6.810816 4.170108 -2.471326

S -8.324213 1.051767 -2.444165

C -8.508409 1.391591 -0.642945

H -9.009791 0.521275 -0.223265

H -9.160856 2.253382 -0.509691

S -6.990218 1.695571 0.304596

C -6.374743 -0.025701 0.513103

H -5.322006 0.114544 0.762016

H -6.413805 -0.508547 -0.465088

C -7.119143 -0.792330 1.576661

H -6.698942 -1.793522 1.668550

H -7.049837 -0.305903 2.547417

H -8.175072 -0.913963 1.336406

O -4.725295 0.913133 -2.196379

H -3.774023 0.853560 -2.397798

H -5.214580 0.469124 -2.898883

P1-2LIGAND2222+2OH2-CoIId

67

P1-2LIGAND2222+2OH2-CoIId SCF Done: -5462.27399420 A.U.

P -2.209189 1.459020 0.152219

O -1.310026 2.716031 0.492773

O -1.591052 0.267815 0.996758

C -2.187382 -1.077110 1.074802

H -3.239366 -0.956052 1.341688

H -2.125134 -1.523537 0.079199

C 0.168598 2.632101 0.485318

H 0.468038 1.990379 -0.348129

H 0.458565 2.146688 1.416675

C 0.696482 4.025434 0.360774

H 0.365970 4.648866 1.188779

H 1.785007 3.999659 0.373176

H 0.380740 4.490522 -0.572695

C -1.417095 -1.854729 2.093584

H -1.480154 -1.390490 3.075440

H -1.833325 -2.858262 2.166534

H -0.369598 -1.945311 1.814181

S -4.133557 1.803900 0.519503

S -1.802890 0.907728 -1.864630

C -1.989687 2.517960 -2.711121

H -2.259583 2.277076 -3.739137

H -1.026114 3.024734 -2.696539

S -3.225832 3.683772 -2.011402

C -3.365996 4.831062 -3.447956

H -4.139159 5.529674 -3.124607

H -3.760612 4.271270 -4.297048

C -2.065909 5.533415 -3.753583

H -2.239539 6.290118 -4.517568

H -1.660830 6.036388 -2.876634

H -1.309199 4.855502 -4.145656

Co -5.218742 2.622778 -1.421110

P -8.053563 2.661450 -3.636435

O -9.217562 3.415185 -2.876075

O -8.487543 2.683172 -5.163626

C -7.643920 2.195936 -6.266054

H -6.627745 2.557000 -6.090010

H -7.645313 1.104579 -6.219443

C -10.635031 3.038234 -3.071926

H -10.673826 1.999784 -3.409659

H -11.007349 3.676850 -3.872093

C -11.343174 3.244571 -1.771412

H -11.265851 4.277386 -1.437719

H -12.399170 3.007319 -1.891219

H -10.937047 2.596264 -0.994927

C -8.225085 2.710521 -7.544702

H -8.221740 3.798039 -7.572195

H -7.629459 2.347470 -8.380886

H -9.245522 2.359757 -7.683453

S -6.297335 3.551450 -3.299142

S -8.105464 0.596928 -3.193045

C -7.840777 0.502420 -1.380158

H -7.085585 -0.266727 -1.226861

H -8.786143 0.203647 -0.930549

S -7.307150 2.041693 -0.554998

C -7.250758 1.410285 1.177577

H -6.708669 2.193407 1.709097

H -6.632049 0.512520 1.187363

C -8.626432 1.191144 1.757559

H -8.529528 0.900058 2.803021

H -9.229800 2.096078 1.720833

H -9.170544 0.392101 1.256177

O -5.636368 4.620312 -0.233956

H -5.125588 4.975263 0.501219

H -6.275733 5.299235 -0.474406

O -4.971621 0.623316 -2.418361

H -4.071233 0.263907 -2.400470

H -5.316903 0.467722 -3.306249

P1-2LIGAND2222+2OH2-CoIIq

67

P1-2LIGAND2222+2OH2-CoIIq SCF Done: -5462.29627995 A.U.

P -1.916517 1.910160 0.151193

O -0.676513 2.686833 0.750550

O -1.621812 0.375459 0.454975

C -2.657568 -0.658230 0.403638

H -3.510560 -0.276167 -0.167893

H -2.207317 -1.474777 -0.158514

C 0.707894 2.178843 0.588925

H 0.805108 1.781659 -0.424962

H 0.821656 1.359106 1.297381

C 1.638150 3.317736 0.853619

H 1.493748 3.722421 1.853030

H 2.665184 2.963109 0.779983

H 1.507304 4.119359 0.128515

C -3.045717 -1.059185 1.794584

H -3.477156 -0.221948 2.344529

H -3.781609 -1.862227 1.752135

H -2.186297 -1.426008 2.351748

S -3.594372 2.681997 0.835539

S -1.728003 1.992425 -1.969179

C -1.950215 3.813600 -2.121689

H -1.301438 4.137444 -2.933245

H -1.607150 4.295428 -1.207093

S -3.630279 4.414033 -2.459117

C -3.841229 3.831073 -4.189887

H -4.906282 3.977403 -4.369058

H -3.646117 2.754165 -4.201369

C -2.992022 4.581197 -5.185096

H -3.213236 4.224323 -6.190941

H -3.192579 5.650361 -5.158341

H -1.925139 4.429644 -5.025540

Co -5.315291 3.191725 -0.989749

P -8.406085 2.890945 -3.182768

O -9.854201 3.479255 -2.952054

O -8.373893 2.501375 -4.728505

C -7.144556 2.110160 -5.412605

H -6.323780 2.094784 -4.684826

H -7.312449 1.091505 -5.762586

C -11.061961 2.843959 -3.533584

H -11.017469 1.774914 -3.310038

H -11.001230 2.978599 -4.613020

C -12.252627 3.512625 -2.926683

H -12.256652 4.579746 -3.137718

H -13.157379 3.082119 -3.353515

H -12.288507 3.368215 -1.848440

C -6.870625 3.069489 -6.530728

H -6.711917 4.080689 -6.156204

H -5.980088 2.755653 -7.075082

H -7.698859 3.093590 -7.235610

S -7.056067 4.184672 -2.552121

S -8.286342 1.006065 -2.221158

C -8.491659 1.638774 -0.504473

H -9.060469 0.881286 0.031341

H -9.087660 2.549682 -0.525748

S -6.987392 1.990442 0.450574

C -6.362312 0.282748 0.715929

H -5.341832 0.444501 1.063096

H -6.301385 -0.193901 -0.265195

C -7.181407 -0.507571 1.704955

H -6.729161 -1.489174 1.845095

H -7.224941 -0.018221 2.675936

H -8.201894 -0.678029 1.363467

O -5.613276 4.965905 0.096280

H -5.076415 5.278948 0.833429

H -6.319747 5.605428 -0.050053

O -4.934168 1.372030 -1.990172

H -4.030510 1.208246 -2.313601

H -5.578864 0.964263 -2.585877

P1-2LIGAND2222+2OH2-CuIId

67

P1-2LIGAND2222+2OH2-CuIId SCF Done: -5719.98373031 A.U.

P -2.159646 1.478838 0.170565

O -1.245178 2.720669 0.528227

O -1.532152 0.262885 0.974221

C -2.143648 -1.074417 1.038495

H -3.192832 -0.945278 1.313247

H -2.092605 -1.508358 0.036566

C 0.231039 2.613194 0.564166

H 0.548832 1.996915 -0.281505

H 0.485475 2.090202 1.485607

C 0.780845 4.002592 0.509087

H 0.434961 4.597502 1.351676

H 1.868073 3.960843 0.550912

H 0.497877 4.509627 -0.413136

C -1.376078 -1.874498 2.042097

H -1.428563 -1.422650 3.030392

H -1.802016 -2.874663 2.104099

H -0.331064 -1.971652 1.755488

S -4.066323 1.838474 0.567645

S -1.785058 0.974717 -1.866630

C -1.862299 2.622517 -2.653385

H -2.051100 2.427134 -3.708687

H -0.889545 3.099067 -2.545110

S -3.125889 3.786143 -2.013323

C -3.269495 4.897820 -3.474328

H -4.087261 5.564279 -3.195662

H -3.602023 4.299651 -4.323748

C -1.994314 5.656335 -3.752793

H -2.169856 6.368437 -4.558363

H -1.658794 6.216822 -2.881812

H -1.185483 5.002270 -4.076009

Cu -5.195796 2.572210 -1.489522

P -8.037570 2.658956 -3.716046

O -9.183970 3.388888 -2.903651

O -8.535484 2.668965 -5.222506

C -7.739121 2.178281 -6.359062

H -6.757967 2.654852 -6.305807

H -7.616114 1.099528 -6.234988

C -10.605029 3.008949 -3.062155

H -10.652848 1.933716 -3.255319

H -10.968087 3.533464 -3.945360

C -11.316998 3.402602 -1.807507

H -11.240833 4.473131 -1.629089

H -12.372621 3.150689 -1.896523

H -10.914578 2.876832 -0.941690

C -8.480400 2.522407 -7.611702

H -8.605042 3.598252 -7.713494

H -7.916064 2.164010 -8.471206

H -9.460773 2.051234 -7.633453

S -6.282947 3.548309 -3.432454

S -8.046993 0.602063 -3.208237

C -8.110417 0.634664 -1.377938

H -7.570605 -0.256757 -1.060061

H -9.155765 0.559014 -1.082780

S -7.405539 2.093367 -0.527281

C -7.254923 1.377738 1.162599

H -6.729084 2.159424 1.712919

H -6.593566 0.512349 1.099147

C -8.591363 1.055153 1.785686

H -8.437387 0.737365 2.816426

H -9.250930 1.921266 1.801999

H -9.101880 0.240160 1.274848

O -5.614420 4.658124 -0.278101

H -5.122311 5.005055 0.473808

H -6.296302 5.311165 -0.468550

O -4.991208 0.438971 -2.315431

H -4.070142 0.152355 -2.398458

H -5.433958 0.202507 -3.140655

P1-2LIGAND2222+2OH2-FeIIIs

67

P1-2LIGAND2222+2OH2-FeIIIs SCF Done: -5342.78973590 A.U.

P -2.108693 1.553571 0.289192

O -1.286785 2.877715 0.466547

O -1.520620 0.490323 1.277949

C -2.020425 -0.899457 1.498474

H -3.090275 -0.818834 1.702325

H -1.864648 -1.439418 0.562179

C 0.207480 2.879793 0.690368

H 0.641700 2.154557 -0.001460

H 0.352656 2.528346 1.710249

C 0.682165 4.274791 0.467515

H 0.213979 4.971229 1.159287

H 1.757536 4.306893 0.641148

H 0.501129 4.611090 -0.552903

C -1.247924 -1.478686 2.635718

H -1.398484 -0.911473 3.551456

H -1.595734 -2.496530 2.808842

H -0.184434 -1.524668 2.413106

S -4.070348 1.940683 0.620720

S -1.781051 0.822602 -1.682275

C -1.659595 2.424452 -2.556360

H -1.657902 2.164621 -3.614519

H -0.710091 2.890229 -2.300892

S -2.976719 3.676624 -2.253473

C -3.081355 4.445708 -3.938142

H -3.879568 5.180366 -3.824308

H -3.424675 3.673752 -4.628490

C -1.781004 5.087006 -4.351623

H -1.927043 5.583410 -5.310847

H -1.452207 5.839992 -3.637792

H -0.980814 4.361348 -4.489997

Fe -5.238804 2.626392 -1.411058

P -8.112299 2.784087 -3.671916

O -9.189723 3.370042 -2.690923

O -8.619107 3.031314 -5.132391

C -7.943262 2.628424 -6.403224

H -6.944620 3.068240 -6.378886

H -7.866759 1.539560 -6.385995

C -10.672257 3.262242 -2.967566

H -10.864729 2.247519 -3.322977

H -10.871113 3.964372 -3.774955

C -11.382355 3.594455 -1.700304

H -11.144880 4.600120 -1.360793

H -12.455641 3.552409 -1.884669

H -11.155749 2.886142 -0.904423

C -8.778849 3.132700 -7.531345

H -8.860785 4.217003 -7.514043

H -8.304221 2.846419 -8.469357

H -9.775162 2.696644 -7.516376

S -6.324402 3.675707 -3.301508

S -7.973197 0.689818 -3.371152

C -8.370783 0.635209 -1.586418

H -8.136525 -0.386111 -1.288375

H -9.437388 0.808260 -1.461002

S -7.503702 1.787288 -0.436628

C -7.204832 0.639366 0.990475

H -6.643256 1.257548 1.691895

H -6.546246 -0.153760 0.631954

C -8.489798 0.131763 1.593528

H -8.247991 -0.478293 2.463731

H -9.131603 0.943895 1.929588

H -9.055020 -0.502282 0.911850

O -5.558066 4.454017 -0.361892

H -5.082722 4.740242 0.429218

H -6.200414 5.138917 -0.589407

O -4.926069 0.784884 -2.308163

H -4.043083 0.369256 -2.307357

H -5.463992 0.450831 -3.044725

P1-2LIGAND2222+2OH2-MnIIs

67

P1-2LIGAND2222+2OH2-MnIIs SCF Done: -5230.57604036 A.U.

P -1.908883 1.892900 0.086188

O -0.645801 2.642291 0.672600

O -1.636833 0.346948 0.348125

C -2.695326 -0.664769 0.355585

H -3.574364 -0.260752 -0.159186

H -2.296464 -1.486017 -0.237875

C 0.731132 2.140489 0.444837

H 0.790829 1.772453 -0.582725

H 0.870635 1.301132 1.125240

C 1.670744 3.272157 0.708211

H 1.562812 3.648003 1.723448

H 2.694461 2.920927 0.587042

H 1.513980 4.094457 0.011748

C -3.003984 -1.067051 1.765810

H -3.397924 -0.228817 2.341355

H -3.744905 -1.866417 1.765046

H -2.115044 -1.440421 2.269991

S -3.545886 2.666106 0.877426

S -1.781923 2.018709 -2.028573

C -1.895674 3.856420 -2.154934

H -1.247560 4.141847 -2.980994

H -1.490271 4.299826 -1.246533

S -3.537175 4.579268 -2.428496

C -3.844415 4.071142 -4.170220

H -4.888503 4.346311 -4.325179

H -3.782740 2.979782 -4.207914

C -2.932224 4.736957 -5.168936

H -3.222822 4.444043 -6.177785

H -2.991611 5.821561 -5.106049

H -1.890302 4.443550 -5.045090

Mn -5.297590 3.225877 -0.943655

P -8.403453 2.913969 -3.125100

O -9.874086 3.470323 -2.979703

O -8.273109 2.519816 -4.663945

C -7.006741 2.108866 -5.266790

H -6.203969 2.266620 -4.537388

H -7.096077 1.038485 -5.459876

C -11.032271 2.796597 -3.617565

H -10.978812 1.734142 -3.366425

H -10.909072 2.908590 -4.694217

C -12.270908 3.452306 -3.099590

H -12.285290 4.513583 -3.337418

H -13.138244 2.990742 -3.569581

H -12.369058 3.332736 -2.022368

C -6.781884 2.901621 -6.517597

H -6.693238 3.965976 -6.302473

H -5.865121 2.567075 -7.002712

H -7.598755 2.762755 -7.222552

S -7.135248 4.266142 -2.441100

S -8.307322 1.028321 -2.156071

C -8.568811 1.626823 -0.428432

H -9.151690 0.852717 0.066660

H -9.172288 2.532611 -0.454659

S -7.098432 1.976910 0.573229

C -6.464191 0.272497 0.845905

H -5.452505 0.441418 1.217514

H -6.373840 -0.203551 -0.134066

C -7.298754 -0.533518 1.808916

H -6.849224 -1.516052 1.950394

H -7.365813 -0.052159 2.782378

H -8.311349 -0.701177 1.443280

O -5.621678 5.048890 0.294782

H -5.024782 5.406194 0.961506

H -6.384459 5.636685 0.262050

O -5.021992 1.357190 -2.096659

H -4.190301 1.056630 -2.489768

H -5.756755 0.814992 -2.422659

P1-2LIGAND2222+2OH2-NiIIt

67

P1-2LIGAND2222+2OH2-NiIIt SCF Done: -5587.84995797 A.U.

P -1.928986 2.088203 0.149111

O -0.733715 2.773400 0.920541

O -1.704778 0.528946 0.377523

C -2.662536 -0.483816 -0.072511

H -3.599804 0.015033 -0.337377

H -2.237401 -0.925459 -0.975828

C 0.642889 2.219576 0.879065

H 0.879021 1.984693 -0.161976

H 0.624352 1.293003 1.451731

C 1.556735 3.249079 1.459740

H 1.281247 3.489639 2.484175

H 2.572256 2.855595 1.468660

H 1.556499 4.164612 0.871538

C -2.847763 -1.487154 1.023813

H -3.258647 -1.023492 1.920105

H -3.533835 -2.266293 0.692213

H -1.905997 -1.963985 1.287114

S -3.622858 2.891165 0.785031

S -1.610929 2.327839 -1.935337

C -1.939697 4.139519 -1.980384

H -1.250617 4.562380 -2.708262

H -1.710521 4.573576 -1.008485

S -3.627709 4.643844 -2.413719

C -3.699361 4.076354 -4.162664

H -4.734094 4.271705 -4.444306

H -3.553695 2.992023 -4.168775

C -2.723915 4.785580 -5.068408

H -2.898997 4.478192 -6.098909

H -2.841494 5.866442 -5.019859

H -1.687086 4.541566 -4.841419

Ni -5.251508 3.278314 -1.040638

P -8.354533 2.875218 -3.143233

O -9.752468 3.546329 -2.837628

O -8.447697 2.446215 -4.675125

C -7.327054 1.858335 -5.404289

H -6.429231 2.439617 -5.167945

H -7.191822 0.835718 -5.037837

C -11.027080 2.945794 -3.303377

H -11.002316 1.879820 -3.062868

H -11.050716 3.059919 -4.386537

C -12.137435 3.669240 -2.612750

H -12.121499 4.731696 -2.845511

H -13.090027 3.265031 -2.951863

H -12.085968 3.546063 -1.532353

C -7.658308 1.889469 -6.863819

H -7.798369 2.910042 -7.213748

H -6.844276 1.441926 -7.431709

H -8.563115 1.322445 -7.071809

S -6.911051 4.143645 -2.664348

S -8.238256 1.039322 -2.107632

C -8.396230 1.770961 -0.421564

H -8.985759 1.063712 0.158218

H -8.959522 2.701000 -0.485226

S -6.863421 2.120020 0.477630

C -6.235014 0.413340 0.734295

H -5.257462 0.587774 1.183617

H -6.070761 -0.031617 -0.249671

C -7.117377 -0.434373 1.616172

H -6.626418 -1.389241 1.804064

H -7.303958 0.038029 2.578745

H -8.076919 -0.663398 1.153629

O -5.744028 5.055065 0.059084

H -5.150222 5.498492 0.674925

H -6.349142 5.722218 -0.283981

O -4.809247 1.461244 -2.022627

H -3.949673 1.423300 -2.473528

H -5.483826 1.082228 -2.604502

P1-2LIGAND2222+2OH2-ZnII

67

P1-2LIGAND2222+2OH2-ZnII SCF Done: -5858.88492836 A.U.

P -1.920122 2.099063 0.228116

O -0.729957 2.798686 0.994237

O -1.712657 0.546137 0.510817

C -2.653429 -0.478918 0.055561

H -3.585436 0.008890 -0.246178

H -2.202380 -0.938337 -0.826096

C 0.645063 2.240991 0.977387

H 0.891804 1.986518 -0.056524

H 0.618333 1.325108 1.566648

C 1.555282 3.278775 1.549213

H 1.269908 3.537745 2.566456

H 2.569890 2.883758 1.575216

H 1.562618 4.184001 0.945348

C -2.866679 -1.459498 1.167245

H -3.308994 -0.979641 2.039722

H -3.535537 -2.251535 0.831134

H -1.930193 -1.922659 1.470585

S -3.612800 2.927931 0.830188

S -1.563497 2.249165 -1.855770

C -1.832484 4.068239 -2.008947

H -1.118930 4.417370 -2.752737

H -1.595178 4.551260 -1.062388

S -3.494595 4.603634 -2.499502

C -3.537273 3.955526 -4.219820

H -4.593184 4.006445 -4.486325

H -3.257833 2.898453 -4.179837

C -2.670348 4.738689 -5.173801

H -2.768265 4.328211 -6.178299

H -2.959880 5.786736 -5.209939

H -1.613831 4.687429 -4.913407

Zn -5.223383 3.275689 -1.068391

P -8.335264 2.920941 -3.213522

O -9.727629 3.553767 -2.807175

O -8.503894 2.538784 -4.749637

C -7.392619 2.140133 -5.612149

H -6.624713 2.915145 -5.543280

H -6.983001 1.201885 -5.223818

C -11.011169 2.950910 -3.242960

H -10.925277 1.865426 -3.147911

H -11.129044 3.200500 -4.296843

C -12.089487 3.522749 -2.380691

H -12.130165 4.606536 -2.464621

H -13.050945 3.124764 -2.701738

H -11.950368 3.257197 -1.333653

C -7.924773 1.985081 -7.002421

H -8.338470 2.921750 -7.369485

H -7.116831 1.687315 -7.668846

H -8.697032 1.220208 -7.046516

S -6.879640 4.185418 -2.776885

S -8.181334 1.046767 -2.242093

C -8.415811 1.703332 -0.532790

H -8.987025 0.946699 0.001176

H -9.023641 2.605990 -0.580739

S -6.933701 2.089817 0.435770

C -6.266749 0.399850 0.717647

H -5.294804 0.600548 1.169399

H -6.088863 -0.050922 -0.261651

C -7.136256 -0.453484 1.605964

H -6.639441 -1.406001 1.790006

H -7.318144 0.018815 2.569315

H -8.098684 -0.686723 1.151560

O -5.710070 5.154915 -0.052900

H -5.243572 5.579928 0.674694

H -6.425942 5.743020 -0.317752

O -4.774428 1.414516 -2.068305

H -3.901201 1.330371 -2.483644

H -5.439191 1.014867 -2.647018

P1-2LIGAND2222cis+1H2Oeq-CoIIq

64

P1-2LIGAND2222cis+1H2Oeq-CoIIq SCF Done: -5385.82055358 A.U.

P -1.552557 1.409914 0.433218

O -1.491212 2.947504 0.810386

O -0.268553 0.761693 1.091930

C 0.031515 -0.684626 1.066026

H -0.860990 -1.212304 1.408116

H 0.229863 -0.956173 0.026604

C -0.213858 3.701115 0.765166

H 0.304876 3.436980 -0.160836

H 0.386502 3.353030 1.604669

C -0.551112 5.154934 0.851617

H -1.074339 5.383691 1.777729

H 0.368803 5.737298 0.834254

H -1.169209 5.477919 0.014321

C 1.214235 -0.913675 1.950496

H 1.000802 -0.628222 2.978282

H 1.466273 -1.972803 1.939770

H 2.082820 -0.358433 1.602882

S -3.258823 0.581130 1.074571

S -1.268431 1.250225 -1.663563

C -2.109961 2.782942 -2.196790

H -2.132092 2.722668 -3.285144

H -1.511815 3.642977 -1.905156

S -3.814861 3.013858 -1.570140

C -4.644205 3.791668 -3.019611

H -5.690734 3.824045 -2.713436

H -4.566365 3.106961 -3.865852

C -4.104026 5.163375 -3.337190

H -4.668486 5.595166 -4.163186

H -4.189477 5.835970 -2.485702

H -3.059399 5.132665 -3.644855

Co -4.918955 0.982213 -0.533944

P -7.926882 0.010968 -0.189930

O -9.141134 -0.953806 -0.525531

O -8.506958 1.344757 0.496133

C -9.882243 1.840956 0.205972

H -9.761728 2.921331 0.155419

H -10.177810 1.478705 -0.779975

C -9.436639 -2.187012 0.248090

H -10.523964 -2.214574 0.266123

H -9.072323 -2.053268 1.268150

C -8.835364 -3.376653 -0.430126

H -7.745576 -3.335703 -0.417538

H -9.132084 -4.281620 0.098616

H -9.184021 -3.463502 -1.458095

C -10.819112 1.408253 1.288219

H -10.484749 1.752361 2.264815

H -11.803433 1.835854 1.101331

H -10.929823 0.325327 1.315943

S -6.404774 -0.719040 0.786511

S -7.339063 0.750172 -2.099501

C -6.487783 -0.785250 -2.653176

H -6.630523 -0.878899 -3.727684

H -6.937231 -1.649879 -2.168385

S -4.704429 -0.758977 -2.277167

C -4.099931 0.058664 -3.814912

H -3.097185 0.393955 -3.547465

H -4.720810 0.943660 -3.970411

C -4.089576 -0.866528 -5.005876

H -3.710071 -0.335157 -5.878168

H -3.452274 -1.731849 -4.836161

H -5.086041 -1.226350 -5.261612

O -6.013814 2.586500 0.362567

H -6.867333 2.380041 0.782370

H -5.533190 3.175103 0.956512

P1-2LIGAND2222cis+1H2Oeq-CuIId

64

P1-2LIGAND2222cis+1H2Oeq-CuIId SCF Done: -5643.52232131 A.U.

P -1.903327 2.424576 0.454631

O -0.676319 3.225156 1.038911

O -1.701838 0.918562 0.923841

C -2.831595 -0.016452 1.026576

H -3.447681 0.314794 1.864731

H -3.431771 0.064675 0.113650

C 0.696736 2.659842 1.057596

H 0.940316 2.359785 0.035249

H 0.666854 1.772035 1.688579

C 1.606965 3.721034 1.583649

H 1.324672 4.020500 2.590619

H 2.621794 3.327508 1.622158

H 1.610868 4.600525 0.943357

C -2.276378 -1.390910 1.220400

H -1.672138 -1.448822 2.123316

H -3.100598 -2.095761 1.322878

H -1.666965 -1.700404 0.373454

S -3.555765 3.390695 0.993921

S -1.718598 2.280140 -1.652100

C -2.212892 4.005215 -2.023009

H -1.766771 4.264019 -2.982320

H -1.812812 4.677014 -1.266022

S -4.008534 4.316430 -2.131454

C -4.326757 3.881753 -3.889342

H -5.388996 3.634474 -3.911513

H -3.760795 2.974008 -4.101174

C -3.980196 5.012782 -4.828144

H -4.194109 4.711041 -5.853075

H -4.561752 5.906699 -4.613768

H -2.924260 5.277305 -4.783731

Cu -5.051897 2.630637 -0.739762

P -7.448093 0.128281 0.311221

O -6.786900 -1.307955 0.190730

O -8.749880 -0.100425 1.184644

C -9.650079 0.986260 1.618452

H -9.034845 1.755119 2.090024

H -10.115423 1.401318 0.720536

C -7.611937 -2.517419 -0.042919

H -8.341957 -2.286182 -0.823846

H -8.152136 -2.715956 0.881988

C -6.681315 -3.622860 -0.426139

H -5.969634 -3.833557 0.369410

H -7.255713 -4.529665 -0.609313

H -6.127127 -3.386780 -1.334590

C -10.654697 0.394300 2.553846

H -10.174752 -0.024666 3.435709

H -11.338759 1.175617 2.881331

H -11.240508 -0.382435 2.067117

S -6.168500 1.451708 1.053427

S -8.162910 0.654782 -1.629124

C -6.783283 0.010964 -2.630386

H -6.992237 0.350003 -3.645092

H -6.813876 -1.076242 -2.602644

S -5.084506 0.538771 -2.165381

C -4.274046 0.335346 -3.811341

H -3.280606 0.758027 -3.656734

H -4.805985 0.961846 -4.528653

C -4.205656 -1.109003 -4.243798

H -3.649681 -1.179005 -5.178420

H -3.697794 -1.726627 -3.504869

H -5.192239 -1.532026 -4.427954

O -6.850078 3.653466 -1.486076

H -7.605671 3.045237 -1.505345

H -7.166275 4.463154 -1.068225

P1-2LIGAND2222cis+1H2Oeq-FeIIId

64

P1-2LIGAND2222cis+1H2Oeq-FeIIId SCF Done: -5266.28568188 A.U.

P -1.414578 1.252453 -0.070852

O -0.837494 2.465625 0.755997

O -0.612712 -0.088911 0.024268

C 0.828603 -0.297339 -0.375998

H 0.948331 -1.360880 -0.189495

H 0.874657 -0.118542 -1.450970

C -0.753867 2.481216 2.254230

H 0.293402 2.703864 2.447103

H -0.972789 1.477189 2.622668

C -1.684248 3.523579 2.779994

H -2.723586 3.279287 2.555155

H -1.584489 3.579307 3.863330

H -1.447115 4.506734 2.377055

C 1.771507 0.552957 0.407480

H 1.669491 0.384642 1.478388

H 2.788475 0.272423 0.133604

H 1.660044 1.614586 0.186887

S -3.293387 0.711553 0.619846

S -1.324979 1.937526 -2.033525

C -2.358876 3.420657 -1.780133

H -2.072875 4.138825 -2.548278

H -2.153349 3.867114 -0.808812

S -4.161686 3.167827 -1.925345

C -4.331453 3.073039 -3.768358

H -5.277246 2.554135 -3.934388

H -3.521818 2.431846 -4.123238

C -4.316308 4.439590 -4.406026

H -4.419767 4.322554 -5.484705

H -5.135144 5.064024 -4.056277

H -3.380284 4.969430 -4.234174

Fe -5.060308 1.382496 -0.604594

P -7.969955 0.606084 -0.036396

O -8.940478 -0.497605 -0.587183

O -8.734655 1.791130 0.642868

C -10.183097 2.153178 0.361685

H -10.143200 3.236002 0.276545

H -10.448704 1.721556 -0.603454

C -9.208726 -1.833187 0.075671

H -10.292227 -1.907862 0.028405

H -8.899773 -1.754331 1.118445

C -8.497583 -2.905087 -0.676284

H -7.414349 -2.788399 -0.619138

H -8.742586 -3.869420 -0.230949

H -8.814931 -2.941251 -1.717493

C -11.034478 1.671498 1.486161

H -10.715603 2.086350 2.439718

H -12.058153 2.001426 1.307725

H -11.049217 0.584557 1.554952

S -6.344344 0.053910 0.967730

S -7.134760 1.460128 -1.863764

C -6.579918 -0.095066 -2.673385

H -6.445031 0.117572 -3.733192

H -7.329713 -0.874976 -2.548379

S -4.985428 -0.557525 -1.893458

C -3.846539 -0.540164 -3.344973

H -2.860885 -0.625534 -2.885953

H -3.918373 0.435608 -3.822932

C -4.151733 -1.689850 -4.275278

H -3.421178 -1.683810 -5.084286

H -4.079853 -2.652685 -3.773549

H -5.136099 -1.608764 -4.735146

O -5.716530 2.954009 0.522458

H -6.004904 2.814431 1.436067

H -5.464790 3.881525 0.415611

P1-2LIGAND2222cis+1H2Oeq-FeIIIq

64

P1-2LIGAND2222cis+1H2Oeq-FeIIIq SCF Done: -5266.30108064 A.U.

P -1.593651 1.482136 0.472150

O -1.488208 3.025184 0.748509

O -0.422838 0.773097 1.226192

C -0.062685 -0.685553 1.193999

H -0.969248 -1.236389 1.448094

H 0.227262 -0.904609 0.164876

C -0.162015 3.749527 0.750202

H 0.410872 3.380442 -0.103650

H 0.335795 3.447511 1.669828

C -0.456513 5.209096 0.684593

H -1.048444 5.535736 1.536563

H 0.486596 5.754474 0.708395

H -0.972774 5.482780 -0.235203

C 1.045981 -0.889467 2.169209

H 0.739323 -0.641792 3.182783

H 1.328478 -1.941589 2.152530

H 1.924574 -0.305635 1.905268

S -3.417246 0.810803 1.118249

S -1.368456 1.124980 -1.597762

C -2.225288 2.604680 -2.213319

H -2.327859 2.458156 -3.288135

H -1.615552 3.487992 -2.029328

S -3.883138 2.983502 -1.498805

C -4.772890 3.712152 -2.951649

H -5.761298 3.927070 -2.543067

H -4.872656 2.937690 -3.714141

C -4.086433 4.955262 -3.462099

H -4.701211 5.387755 -4.251730

H -3.972496 5.707402 -2.683856

H -3.110491 4.747249 -3.898038

Fe -5.050147 1.132782 -0.473445

P -7.918967 0.225565 -0.164323

O -9.005557 -0.801331 -0.618747

O -8.550523 1.453180 0.602035

C -10.035240 1.814945 0.536337

H -10.007008 2.899565 0.598187

H -10.392398 1.518223 -0.449491

C -9.235717 -2.194284 -0.059734

H -10.317306 -2.282867 -0.118386

H -8.932118 -2.178754 0.986861

C -8.506243 -3.197278 -0.885029

H -7.423579 -3.079668 -0.811346

H -8.743792 -4.194158 -0.512628

H -8.817602 -3.161036 -1.927959

C -10.759852 1.165746 1.663588

H -10.342945 1.445082 2.628803

H -11.796442 1.503583 1.642554

H -10.772144 0.080378 1.574970

S -6.273729 -0.460767 0.757126

S -7.142282 1.115669 -1.953170

C -6.508214 -0.436378 -2.743957

H -6.618925 -0.311242 -3.820027

H -7.134797 -1.269103 -2.425984

S -4.767724 -0.743124 -2.313295

C -3.940324 -0.189998 -3.876407

H -2.883830 -0.204369 -3.608035

H -4.235197 0.846261 -4.058845

C -4.232013 -1.101747 -5.041017

H -3.675367 -0.754449 -5.911296

H -3.925762 -2.125490 -4.836899

H -5.284602 -1.106930 -5.322123

O -6.059245 2.831542 0.509493

H -6.927552 2.706643 0.925390

H -5.561987 3.426415 1.085828

P1-2LIGAND2222cis+1H2Oeq-FeIIIs

64

P1-2LIGAND2222cis+1H2Oeq-FeIIIs SCF Done: -5266.31717616 A.U.

P -1.311416 1.145510 0.244132

O -0.665704 2.548075 0.545115

O -0.560856 -0.117640 0.767742

C 0.859919 -0.527972 0.449405

H 0.910274 -1.500283 0.931019

H 0.905837 -0.660982 -0.631443

C -0.484896 3.107776 1.934335

H 0.595209 3.134129 2.064249

H -0.911129 2.396399 2.644358

C -1.125826 4.452709 1.984427

H -2.202228 4.390332 1.826721

H -0.957816 4.886524 2.969749

H -0.692199 5.129073 1.250254

C 1.854076 0.451811 0.975185

H 1.733686 0.610092 2.045902

H 2.851786 0.041091 0.820800

H 1.815234 1.406906 0.451315

S -3.169213 1.010306 1.125851

S -1.350361 1.081241 -1.844206

C -1.924337 2.783970 -2.182637

H -1.804772 2.919730 -3.257432

H -1.286151 3.492589 -1.659521

S -3.678459 3.126565 -1.744942

C -4.401495 3.749543 -3.341372

H -5.477294 3.668964 -3.173677

H -4.125591 3.035768 -4.117524

C -3.960198 5.156013 -3.650148

H -4.436715 5.474449 -4.577667

H -4.248256 5.856878 -2.869813

H -2.884152 5.229010 -3.801212

Fe -4.753029 1.087325 -0.544773

P -7.895565 -0.148764 -0.404089

O -8.897922 -1.349379 -0.324936

O -8.539952 1.228849 0.078314

C -9.981356 1.596437 -0.246484

H -9.914969 2.664624 -0.438795

H -10.246886 1.085366 -1.173336

C -9.184648 -2.143866 0.935469

H -9.302423 -1.423890 1.747197

H -8.292147 -2.744761 1.109043

C -10.405974 -2.955717 0.676337

H -10.267110 -3.634103 -0.162403

H -10.607888 -3.557951 1.562229

H -11.279235 -2.333717 0.491272

C -10.871242 1.253046 0.898208

H -10.538944 1.718626 1.823834

H -11.871107 1.629650 0.680437

H -10.958196 0.177717 1.043276

S -6.183579 -0.442963 0.630239

S -7.490133 0.144850 -2.439606

C -6.230375 -1.176548 -2.630771

H -6.312822 -1.551338 -3.650193

H -6.416143 -2.003049 -1.948276

S -4.506015 -0.613261 -2.383701

C -4.224662 0.196731 -4.025325

H -3.354284 0.830667 -3.853663

H -5.084482 0.843693 -4.212686

C -3.986162 -0.810906 -5.121833

H -3.802486 -0.277689 -6.054710

H -3.121345 -1.438236 -4.917609

H -4.846398 -1.456282 -5.293799

O -6.137558 2.651321 -0.161998

H -7.042003 2.430794 0.137367

H -5.891551 3.496395 0.236485

P1-2LIGAND2222cis+1H2Oeq-MnIIs

64

P1-2LIGAND2222cis+1H2Oeq-MnIIs SCF Done: -5154.09080652 A.U.

P -1.936698 2.512927 0.535498

O -0.666998 3.286429 1.059822

O -1.744516 1.001332 0.999132

C -2.883460 0.082991 1.111324

H -3.497045 0.421228 1.948819

H -3.486356 0.165450 0.198537

C 0.696733 2.705404 0.982288

H 0.868880 2.412075 -0.056633

H 0.697242 1.811766 1.605806

C 1.652879 3.750579 1.457107

H 1.439062 4.044578 2.482296

H 2.663002 3.344468 1.427288

H 1.626737 4.635811 0.825213

C -2.349709 -1.300300 1.303403

H -1.746228 -1.367098 2.206226

H -3.185285 -1.991823 1.405825

H -1.745188 -1.618235 0.456135

S -3.567523 3.470815 1.110326

S -1.828263 2.346749 -1.582089

C -2.192740 4.115352 -1.970993

H -1.630125 4.358696 -2.870334

H -1.831284 4.748148 -1.162878

S -3.944756 4.509530 -2.245344

C -4.124613 3.997919 -4.005598

H -5.197276 3.842380 -4.131345

H -3.636178 3.026849 -4.105113

C -3.579341 5.018882 -4.972858

H -3.719782 4.668698 -5.995120

H -4.085582 5.976590 -4.873493

H -2.511619 5.186938 -4.834729

Mn -5.066000 2.642110 -0.744697

P -7.545195 0.059922 0.350678

O -6.880264 -1.372754 0.191899

O -8.886325 -0.203507 1.152178

C -9.701470 0.854313 1.779659

H -9.020875 1.526823 2.305292

H -10.195097 1.403549 0.974489

C -7.698238 -2.577967 -0.075594

H -8.399223 -2.342309 -0.881780

H -8.273444 -2.781395 0.826774

C -6.756051 -3.683999 -0.429228

H -6.070475 -3.894940 0.388999

H -7.324587 -4.590450 -0.631371

H -6.171834 -3.448693 -1.318861

C -10.678894 0.187901 2.693655

H -10.170515 -0.362761 3.482114

H -11.303138 0.947404 3.161812

H -11.330116 -0.493506 2.150352

S -6.301553 1.363312 1.149763

S -8.195693 0.626068 -1.616767

C -6.825126 -0.061546 -2.598469

H -7.028054 0.250941 -3.622621

H -6.863146 -1.146601 -2.541590

S -5.109861 0.459230 -2.166997

C -4.325430 0.346816 -3.835948

H -3.326611 0.752889 -3.670325

H -4.863495 1.022037 -4.502292

C -4.279100 -1.068904 -4.353155

H -3.747358 -1.087420 -5.304142

H -3.758430 -1.732474 -3.664914

H -5.273548 -1.473645 -4.536944

O -6.889004 3.617383 -1.447133

H -7.668990 3.039067 -1.478339

H -7.198469 4.510228 -1.255330

P1-2LIGAND2222cis+1H2Oeq-NiII

64

P1-2LIGAND2222cis+1H2Oeq-NiII SCF Done: -5511.33893701 A.U.

P -1.919332 2.463898 0.428336

O -0.839146 3.343281 1.165804

O -1.660966 0.985203 0.951826

C -2.533074 -0.150848 0.638444

H -3.457847 -0.013112 1.201522

H -2.774410 -0.112179 -0.430355

C 0.570603 2.902468 1.313651

H 0.938240 2.646594 0.316460

H 0.560102 2.003402 1.929005

C 1.325867 4.029881 1.937894

H 0.921174 4.282576 2.915601

H 2.363695 3.727398 2.071313

H 1.311660 4.918517 1.310670

C -1.804435 -1.402856 1.012592

H -1.551307 -1.406428 2.070597

H -2.443067 -2.263240 0.817918

H -0.889862 -1.521357 0.434736

S -3.674875 3.374171 0.773841

S -1.481465 2.308161 -1.637312

C -2.228431 3.894117 -2.127943

H -1.824378 4.149789 -3.106491

H -1.952790 4.677169 -1.425033

S -4.057482 3.900780 -2.251956

C -4.283360 3.489987 -4.029445

H -5.309850 3.124188 -4.081598

H -3.607099 2.670286 -4.271318

C -4.066763 4.697689 -4.909672

H -4.230089 4.419417 -5.950362

H -4.755579 5.503640 -4.665420

H -3.051092 5.085668 -4.837755

Ni -4.904928 2.327697 -0.852996

P -7.412480 0.093014 0.302267

O -6.914189 -1.397322 0.115153

O -8.620572 0.009420 1.320333

C -9.334198 1.188708 1.858351

H -8.582003 1.928132 2.140536

H -9.944334 1.589454 1.045638

C -7.868395 -2.503264 -0.139084

H -8.665575 -2.123477 -0.784029

H -8.300902 -2.767202 0.825259

C -7.100643 -3.622417 -0.765417

H -6.305117 -3.972050 -0.110601

H -7.771945 -4.458967 -0.953079

H -6.662448 -3.322383 -1.717084

C -10.159124 0.728660 3.016427

H -9.536006 0.324005 3.811086

H -10.710152 1.577653 3.418290

H -10.881257 -0.026610 2.713849

S -5.932827 1.285964 0.938457

S -8.301060 0.728038 -1.519467

C -6.998700 0.275141 -2.714027

H -7.164679 0.918133 -3.578381

H -7.133300 -0.767163 -2.999039

S -5.264801 0.452687 -2.127609

C -4.379124 0.138660 -3.716815

H -3.362746 0.477322 -3.506739

H -4.803707 0.780658 -4.487719

C -4.412628 -1.322056 -4.096888

H -3.792136 -1.474744 -4.979608

H -4.022905 -1.955159 -3.301541

H -5.415298 -1.662262 -4.351088

O -6.841933 3.613230 -1.530292

H -7.676104 3.148252 -1.378540

H -6.990066 4.514238 -1.221466

P1-2LIGAND2222cis+1H2Oeq-NiIIt

64

P1-2LIGAND2222cis+1H2Oeq-NiIIt SCF Done: -5511.37218758 A.U.

P -1.914404 1.647411 0.436167

O -1.146979 2.397416 1.597009

O -1.206003 0.224975 0.347126

C -1.726125 -0.889876 -0.446279

H -2.780387 -1.021945 -0.181319

H -1.667225 -0.608339 -1.500851

C 0.332536 2.362028 1.698591

H 0.739586 2.533872 0.698661

H 0.600206 1.355128 2.017343

C 0.740338 3.414497 2.677551

H 0.295684 3.239472 3.654740

H 1.823063 3.391439 2.791951

H 0.459558 4.409886 2.338086

C -0.898636 -2.096406 -0.131409

H -0.960928 -2.353331 0.923913

H -1.260401 -2.945146 -0.709730

H 0.145894 -1.935928 -0.389995

S -3.858282 1.653015 0.880136

S -1.427876 2.561196 -1.401883

C -2.251060 4.158497 -1.054776

H -1.663679 4.930424 -1.548384

H -2.252007 4.367967 0.013822

S -3.969417 4.312042 -1.635327

C -3.708229 4.252144 -3.455829

H -4.699680 4.041918 -3.856839

H -3.070841 3.383781 -3.644145

C -3.123377 5.528335 -4.007721

H -3.018264 5.437650 -5.088229

H -3.760051 6.386699 -3.804566

H -2.131382 5.736759 -3.609051

Ni -5.238942 2.395997 -0.942152

P -8.182388 0.415019 -0.261048

O -8.012836 -1.031508 0.391137

O -9.702965 0.845648 -0.277762

C -10.809650 -0.141665 -0.398837

H -11.507702 0.341490 -1.078736

H -10.422756 -1.044506 -0.875353

C -7.584218 -1.247411 1.788904

H -8.222443 -2.058648 2.133473

H -7.808077 -0.353117 2.372485

C -6.129147 -1.598811 1.816481

H -5.517290 -0.766766 1.458714

H -5.821748 -1.814215 2.838994

H -5.927073 -2.484566 1.214477

C -11.397867 -0.403499 0.950947

H -11.741394 0.517591 1.417083

H -12.255351 -1.066869 0.845380

H -10.683442 -0.889202 1.614469

S -7.087275 1.833995 0.573316

S -7.750426 -0.015192 -2.285692

C -6.040412 -0.628644 -2.040078

H -5.908424 -1.488010 -2.694306

H -5.915057 -0.959115 -1.008248

S -4.699269 0.561120 -2.377660

C -5.021119 0.951617 -4.152644

H -4.176492 1.584770 -4.420962

H -5.923643 1.560651 -4.210366

C -5.094729 -0.274654 -5.027888

H -5.139058 0.036435 -6.070790

H -4.221673 -0.915123 -4.911916

H -5.989151 -0.867529 -4.840784

O -6.701053 3.185911 -2.338371

H -7.333050 2.476329 -2.546562

H -7.247152 3.919149 -2.024780

P1-2LIGAND2222cis+1H2Oeq-ZnII

64

P1-2LIGAND2222cis+1H2Oeq-ZnII SCF Done: -5782.41863873 A.U.

P -1.583383 2.212562 0.567970

O -0.737885 3.174146 1.488364

O -0.878696 0.792055 0.699417

C -1.468128 -0.452169 0.206957

H -2.513948 -0.483756 0.536662

H -1.445883 -0.421475 -0.885796

C 0.746467 3.161512 1.480938

H 1.074382 3.202235 0.439160

H 1.054609 2.208641 1.910311

C 1.206293 4.337604 2.279254

H 0.839076 4.288525 3.302083

H 2.294816 4.337485 2.312599

H 0.883050 5.276630 1.834583

C -0.667312 -1.588158 0.762253

H -0.699887 -1.599004 1.849491

H -1.074905 -2.530963 0.400768

H 0.371247 -1.528005 0.444062

S -3.466193 2.367880 1.227788

S -1.331957 2.718023 -1.453718

C -2.352925 4.256566 -1.426334

H -1.905807 4.929551 -2.155347

H -2.277063 4.724163 -0.447209

S -4.108510 4.046348 -1.826216

C -4.080134 4.325307 -3.649767

H -5.043079 3.943899 -3.990781

H -3.301221 3.686618 -4.072283

C -3.891811 5.776026 -4.016483

H -3.903328 5.881755 -5.100970

H -4.684223 6.399211 -3.607395

H -2.938056 6.170950 -3.667771

Zn -4.781468 1.649745 -0.582536

P -7.433131 -0.644241 -0.270092

O -8.246724 -1.921790 0.201522

O -8.468634 0.601229 -0.351506

C -9.784767 0.435523 -1.026451

H -9.878075 1.320828 -1.653487

H -9.736674 -0.438897 -1.679552

C -8.597508 -2.121656 1.631226

H -9.001508 -1.182264 2.016310

H -7.666455 -2.340502 2.155638

C -9.580055 -3.245268 1.695797

H -9.160857 -4.161692 1.286209

H -9.840969 -3.429125 2.737035

H -10.495960 -3.010286 1.156338

C -10.876219 0.324562 -0.009819

H -10.874724 1.174660 0.669630

H -11.839301 0.304086 -0.518725

H -10.792756 -0.592421 0.571132

S -5.894458 -0.136638 0.848045

S -6.984795 -1.090973 -2.282666

C -5.188053 -1.368455 -2.154241

H -4.934929 -2.060688 -2.954733

H -4.930395 -1.826668 -1.201484

S -4.096896 0.095609 -2.331203

C -4.685801 0.809149 -3.925132

H -4.012145 1.652893 -4.069582

H -5.691831 1.199765 -3.760634

C -4.620273 -0.167390 -5.071138

H -4.847817 0.355733 -5.999312

H -3.631951 -0.612152 -5.175491

H -5.353118 -0.967370 -4.976057

O -6.620092 2.589464 -0.904340

H -7.420334 2.120916 -0.593151

H -6.718569 3.522402 -0.680454

P1-2LIGAND2222cis+1OH2ax-CoIIq

64

P1-2LIGAND2222cis+1OH2ax-CoIIq SCF Done: -5385.82235113 A.U.

P -1.426534 1.549110 -0.726062

O -2.117553 1.182555 0.656083

O 0.075794 1.068081 -0.610355

C 1.022677 1.040165 -1.747461

H 0.558691 0.453319 -2.542382

H 1.151445 2.068099 -2.093311

C -1.468837 1.497032 1.950782

H -1.043284 2.501774 1.883141

H -0.655195 0.783192 2.074272

C -2.514981 1.379448 3.012532

H -2.945465 0.379965 3.032421

H -2.062594 1.571626 3.984174

H -3.318588 2.101052 2.868434

C 2.297878 0.438102 -1.253157

H 2.143796 -0.579204 -0.899670

H 3.014907 0.405866 -2.072040

H 2.731622 1.029882 -0.449925

S -2.421252 0.612067 -2.191967

S -1.281895 3.643763 -0.833432

C -2.992199 4.083885 -0.358899

H -2.959755 5.158199 -0.185362

H -3.274532 3.593554 0.571794

S -4.325895 3.726153 -1.551513

C -3.595336 4.342881 -3.126492

H -4.354543 4.105197 -3.873543

H -2.720026 3.726049 -3.337072

C -3.269697 5.813891 -3.096835

H -2.917856 6.126885 -4.079256

H -4.136484 6.421090 -2.842286

H -2.471279 6.039962 -2.390234

Co -4.662287 1.211016 -1.882357

P -7.237741 0.122755 0.357228

O -7.349730 -1.007751 1.451999

O -8.359919 1.182059 0.754974

C -8.483034 2.494413 0.121431

H -7.474998 2.906279 0.002574

H -8.924616 2.340603 -0.868485

C -8.664088 -1.534411 1.902798

H -9.263422 -1.739821 1.012293

H -9.141428 -0.737174 2.471463

C -8.399603 -2.758369 2.717130

H -7.777066 -2.532731 3.580163

H -9.347696 -3.151502 3.081116

H -7.916761 -3.536270 2.129038

C -9.350059 3.348819 0.992506

H -8.904461 3.487899 1.974951

H -9.473475 4.328159 0.533109

H -10.337367 2.909281 1.115567

S -5.359366 0.789415 0.356249

S -7.943957 -0.631582 -1.490523

C -6.532135 -1.762895 -1.864761

H -6.966734 -2.624508 -2.368116

H -6.085313 -2.106289 -0.933258

S -5.204420 -1.079586 -2.886383

C -6.064186 -0.935090 -4.510993

H -5.405590 -0.295549 -5.098735

H -6.993776 -0.381818 -4.355242

C -6.299632 -2.268984 -5.173601

H -6.766266 -2.112352 -6.145484

H -5.369239 -2.810309 -5.331818

H -6.971056 -2.906387 -4.599239

O -6.383848 1.903245 -2.914403

H -7.128389 1.284516 -2.804266

H -6.753004 2.793530 -2.859626

P1-2LIGAND2222cis+1OH2ax-CuIId

64

P1-2LIGAND2222cis+1OH2ax-CuIId SCF Done: -5643.52613044 A.U.

P -1.690661 2.318424 0.559124

O -0.732941 3.126279 1.514088

O -1.157499 0.819718 0.620282

C -1.873212 -0.296036 0.000602

H -2.937257 -0.192568 0.247935

H -1.755256 -0.203211 -1.082469

C 0.738353 2.922132 1.514803

H 1.079178 2.970767 0.477402

H 0.916115 1.918022 1.898737

C 1.334554 3.991626 2.370034

H 0.955332 3.940495 3.388367

H 2.414288 3.853727 2.406077

H 1.134871 4.983410 1.969657

C -1.290885 -1.568220 0.532362

H -1.415205 -1.639686 1.610622

H -1.797014 -2.417133 0.074463

H -0.231541 -1.643789 0.296313

S -3.551246 2.698420 1.173186

S -1.387349 2.868906 -1.455765

C -2.391935 4.398008 -1.412642

H -1.996874 5.052126 -2.188519

H -2.286306 4.901048 -0.454391

S -4.175604 4.185579 -1.726837

C -4.156864 4.086845 -3.561130

H -5.133576 3.672553 -3.810192

H -3.390488 3.357974 -3.836194

C -3.933417 5.433456 -4.208405

H -3.959493 5.320464 -5.291738

H -4.703045 6.149025 -3.926707

H -2.963552 5.860573 -3.956203

Cu -4.838531 2.053228 -0.739752

P -7.346890 -0.520456 0.178807

O -7.255324 -1.969971 0.796382

O -8.818864 -0.030264 0.534514

C -9.274315 1.350882 0.370748

H -8.450950 2.012261 0.659547

H -9.497161 1.493521 -0.691941

C -8.388062 -2.928718 0.719765

H -8.693639 -2.992441 -0.327647

H -9.204611 -2.500445 1.299964

C -7.904068 -4.233200 1.263329

H -7.579059 -4.134488 2.296855

H -8.721913 -4.951909 1.235916

H -7.083321 -4.634872 0.672518

C -10.486958 1.544490 1.226026

H -10.253096 1.396431 2.277977

H -10.859988 2.559854 1.101073

H -11.281515 0.857632 0.942431

S -5.805112 0.534819 0.872319

S -7.361198 -0.648943 -1.933883

C -5.571425 -1.041949 -2.135887

H -5.507216 -1.783612 -2.928761

H -5.188665 -1.488295 -1.219453

S -4.470356 0.341219 -2.552010

C -5.142241 0.811293 -4.207532

H -4.410599 1.528201 -4.573940

H -6.083140 1.342835 -4.053205

C -5.285897 -0.350844 -5.158167

H -5.511897 0.032215 -6.152715

H -4.370582 -0.936390 -5.231663

H -6.105467 -1.014853 -4.887643

O -6.776967 2.657502 -1.765346

H -7.332047 1.882062 -1.944773

H -7.372032 3.346521 -1.447032

P1-2LIGAND2222cis+1OH2ax-FeIIId

64

P1-2LIGAND2222cis+1OH2ax-FeIIId SCF Done: -5266.28563501 A.U.

P -1.393665 1.208141 -0.074461

O -0.863811 2.453021 0.738378

O -0.560004 -0.108604 0.064851

C 0.895273 -0.292017 -0.295754

H 1.038219 -1.344609 -0.067897

H 0.960317 -0.150960 -1.375034

C -0.812499 2.491770 2.236492

H 0.226061 2.735339 2.450011

H -1.022864 1.489900 2.615922

C -1.773982 3.524066 2.725383

H -2.802233 3.255887 2.477024

H -1.702714 3.596637 3.809912

H -1.545320 4.505923 2.314316

C 1.796971 0.610892 0.477426

H 1.675607 0.476791 1.551173

H 2.826894 0.348856 0.235766

H 1.662719 1.661058 0.217649

S -3.268962 0.628242 0.591251

S -1.289526 1.860907 -2.047785

C -2.310215 3.358136 -1.821619

H -2.030219 4.051371 -2.614550

H -2.083171 3.827943 -0.866460

S -4.119070 3.125543 -1.928386

C -4.339123 3.068425 -3.767797

H -5.296810 2.566445 -3.917567

H -3.548819 2.423559 -4.156725

C -4.320964 4.446878 -4.379111

H -4.452763 4.352186 -5.456921

H -5.121801 5.075505 -3.996608

H -3.373412 4.960296 -4.220821

Fe -5.032286 1.338206 -0.618535

P -7.949140 0.622948 -0.023104

O -8.982990 -0.433047 -0.550182

O -8.649472 1.838784 0.672546

C -10.099083 2.231401 0.447277

H -10.056608 3.315039 0.515472

H -10.362532 1.942276 -0.570398

C -9.259989 -1.782711 0.077390

H -10.344957 -1.839845 0.039939

H -8.939984 -1.736323 1.118638

C -8.575287 -2.845963 -0.710781

H -7.489288 -2.752073 -0.661193

H -8.833260 -3.817702 -0.289431

H -8.904249 -2.848451 -1.749139

C -10.955302 1.599142 1.490881

H -10.637627 1.874821 2.494069

H -11.977937 1.953226 1.359311

H -10.973258 0.513583 1.404181

S -6.324322 -0.000287 0.938853

S -7.117636 1.456521 -1.861359

C -6.600682 -0.116062 -2.664164

H -6.498644 0.076121 -3.731494

H -7.353499 -0.886519 -2.501898

S -4.988030 -0.585794 -1.929702

C -3.887420 -0.541238 -3.410594

H -2.887501 -0.604354 -2.980028

H -3.996115 0.433487 -3.883609

C -4.193917 -1.695039 -4.335338

H -3.487373 -1.670905 -5.165102

H -4.086832 -2.657212 -3.838837

H -5.192789 -1.634507 -4.766408

O -5.654842 2.904134 0.531553

H -5.935488 2.763989 1.447370

H -5.393413 3.829076 0.425446

P1-2LIGAND2222cis+1OH2ax-FeIIIs

64

P1-2LIGAND2222cis+1OH2ax-FeIIIs SCF Done: -5266.31971279 A.U.

P -1.406486 1.582306 -0.644411

O -2.134079 1.242590 0.712154

O 0.051679 1.029758 -0.580272

C 1.091425 1.061789 -1.672717

H 0.666270 0.505558 -2.508666

H 1.218685 2.108656 -1.951982

C -1.442760 1.450407 2.039232

H -0.958788 2.428721 2.003257

H -0.681639 0.674821 2.099117

C -2.482943 1.345520 3.101662

H -2.976956 0.375892 3.081353

H -1.997629 1.448781 4.071767

H -3.229617 2.134723 3.023202

C 2.330305 0.446589 -1.119448

H 2.167482 -0.587434 -0.824276

H 3.092254 0.455713 -1.898566

H 2.715098 1.009343 -0.272058

S -2.489853 0.706373 -2.146011

S -1.265975 3.659683 -0.872860

C -2.984902 4.102290 -0.465549

H -2.963534 5.180698 -0.309584

H -3.314040 3.627503 0.458229

S -4.295293 3.776032 -1.720572

C -3.459496 4.260986 -3.303015

H -4.199112 4.016758 -4.067378

H -2.607249 3.590382 -3.427834

C -3.073849 5.716947 -3.320610

H -2.655766 5.953585 -4.299085

H -3.924846 6.374908 -3.156320

H -2.304106 5.949507 -2.585408

Fe -4.713766 1.264772 -1.792888

P -7.333903 0.131524 0.372986

O -7.328796 -1.023964 1.422519

O -8.379505 1.203128 0.850225

C -8.697475 2.490445 0.184404

H -7.744557 2.992855 -0.008539

H -9.180564 2.244279 -0.764666

C -8.601681 -1.617785 2.002970

H -9.252684 -1.845636 1.156993

H -9.044310 -0.823816 2.602140

C -8.207078 -2.816828 2.791559

H -7.528134 -2.559169 3.601176

H -9.108200 -3.239314 3.236782

H -7.753206 -3.585121 2.169412

C -9.590011 3.265533 1.096665

H -9.099781 3.486324 2.042070

H -9.847617 4.210440 0.619665

H -10.515870 2.729526 1.292737

S -5.368503 0.738909 0.354069

S -8.030427 -0.540860 -1.501413

C -6.638715 -1.676271 -1.911595

H -7.083538 -2.506123 -2.460175

H -6.194194 -2.074795 -1.000684

S -5.296145 -0.991397 -2.930334

C -6.140785 -0.813191 -4.570879

H -5.486039 -0.143895 -5.129029

H -7.086325 -0.292496 -4.400762

C -6.326316 -2.142282 -5.258806

H -6.787219 -1.965787 -6.230673

H -5.379579 -2.650363 -5.427140

H -6.988646 -2.811795 -4.711863

O -6.415007 1.945491 -2.900504

H -7.216371 1.393912 -2.885588

H -6.690968 2.846613 -3.114505

P1-2LIGAND2222cis+1OH2ax-MnIId

64

P1-2LIGAND2222cis+1OH2ax-MnIId SCF Done: -5154.00189046 A.U.

P -1.526483 2.044963 0.349894

O -0.672464 2.875547 1.385031

O -0.750661 0.665414 0.200436

C -1.332403 -0.531530 -0.414033

H -2.268836 -0.744873 0.110345

H -1.563530 -0.297642 -1.456450

C 0.812049 2.894429 1.338716

H 1.107057 3.168647 0.322618

H 1.147914 1.877707 1.540455

C 1.277518 3.877677 2.362472

H 0.946200 3.596587 3.359807

H 2.366435 3.897057 2.362855

H 0.923109 4.883122 2.144619

C -0.334209 -1.638403 -0.282739

H -0.111912 -1.847493 0.761370

H -0.738910 -2.545179 -0.729810

H 0.593826 -1.396550 -0.796578

S -3.392197 1.949412 1.087575

S -1.383358 2.915191 -1.560483

C -2.412347 4.381587 -1.182947

H -2.037429 5.198101 -1.797255

H -2.309754 4.669278 -0.138237

S -4.192196 4.198987 -1.523000

C -4.167035 4.143978 -3.363197

H -5.154762 3.762402 -3.621765

H -3.426805 3.388445 -3.638772

C -3.887655 5.487775 -3.988135

H -3.918471 5.395216 -5.073157

H -4.625340 6.231782 -3.694579

H -2.899722 5.868065 -3.731198

Mn -5.025400 2.171075 -0.568007

P -7.710912 -0.221627 0.319378

O -7.526844 -1.596007 1.073176

O -9.260382 0.109195 0.461586

C -9.850352 1.397001 0.084604

H -9.239787 2.186981 0.531946

H -9.800041 1.475855 -1.006500

C -8.532697 -2.685485 0.973683

H -8.795891 -2.801955 -0.080826

H -9.412372 -2.347169 1.519686

C -7.917775 -3.915364 1.557201

H -7.632583 -3.759701 2.595449

H -8.647450 -4.723320 1.526850

H -7.041637 -4.232525 0.994944

C -11.261681 1.420914 0.580857

H -11.297865 1.334684 1.664442

H -11.725778 2.365411 0.301173

H -11.847104 0.615211 0.143359

S -6.411679 1.096841 1.055096

S -7.471843 -0.556664 -1.767004

C -5.672922 -0.954044 -1.728264

H -5.522300 -1.779730 -2.420606

H -5.390603 -1.286788 -0.729824

S -4.548151 0.408132 -2.168475

C -5.082975 0.729037 -3.909368

H -4.367638 1.475305 -4.251089

H -6.066119 1.201566 -3.882991

C -5.060927 -0.499255 -4.784139

H -5.234860 -0.203127 -5.818049

H -4.101272 -1.012481 -4.744769

H -5.846295 -1.209002 -4.527743

O -6.731250 2.679774 -1.756878

H -7.248929 1.869971 -1.923073

H -7.354953 3.329841 -1.410000

P1-2LIGAND2222cis+1OH2ax-MnIIs

64

P1-2LIGAND2222cis+1OH2ax-MnIIs SCF Done: -5154.09431871 A.U.

P -1.578438 2.224172 0.549148

O -0.580160 3.164481 1.328756

O -0.907608 0.782798 0.595715

C -1.603785 -0.444133 0.204916

H -2.598280 -0.430484 0.665174

H -1.719999 -0.426618 -0.882440

C 0.889636 3.075267 1.145642

H 1.091599 3.067555 0.071217

H 1.201376 2.120681 1.568583

C 1.496331 4.250199 1.841205

H 1.247767 4.252902 2.900117

H 2.580027 4.194755 1.750192

H 1.169013 5.190437 1.401789

C -0.778742 -1.602807 0.671057

H -0.672517 -1.599199 1.753595

H -1.263626 -2.533775 0.381212

H 0.211993 -1.585683 0.221667

S -3.376415 2.435957 1.358705

S -1.540084 2.723679 -1.509315

C -2.372902 4.356650 -1.334650

H -1.928781 5.016210 -2.077570

H -2.173535 4.773128 -0.349219

S -4.175301 4.344983 -1.578647

C -4.239797 4.259596 -3.419715

H -5.241972 3.889816 -3.638730

H -3.528338 3.488891 -3.725957

C -3.964818 5.588742 -4.077457

H -4.021988 5.479912 -5.160035

H -4.688283 6.343476 -3.776395

H -2.969023 5.965276 -3.846186

Mn -4.881284 1.997092 -0.579671

P -7.608057 -0.510235 0.244262

O -7.585860 -2.001489 0.763031

O -9.103338 -0.022639 0.488030

C -9.527305 1.376363 0.421793

H -8.754847 1.986110 0.899584

H -9.595170 1.645788 -0.637803

C -8.724015 -2.925604 0.529694

H -8.965379 -2.892922 -0.535997

H -9.569817 -2.532794 1.093002

C -8.297335 -4.282845 0.985776

H -8.031229 -4.279400 2.040393

H -9.125054 -4.977549 0.850531

H -7.450715 -4.652726 0.410682

C -10.852149 1.491542 1.108352

H -10.775568 1.211394 2.156570

H -11.197016 2.523109 1.058235

H -11.600114 0.862827 0.630107

S -6.133954 0.492011 1.089327

S -7.480644 -0.570711 -1.888036

C -5.736302 -1.147163 -1.986263

H -5.691374 -1.866983 -2.800355

H -5.471329 -1.663635 -1.064507

S -4.435722 0.098852 -2.270734

C -4.858573 0.694968 -3.966927

H -4.046790 1.388511 -4.186797

H -5.783423 1.271609 -3.904142

C -4.938005 -0.408912 -4.990450

H -5.043158 0.031530 -5.981196

H -4.042028 -1.027145 -4.996575

H -5.804627 -1.051646 -4.841859

O -6.759117 2.606550 -1.623843

H -7.335518 1.858080 -1.859282

H -7.330168 3.363447 -1.447622

P1-2LIGAND2222cis+1OH2ax-NiII

64

P1-2LIGAND2222cis+1OH2ax-NiII SCF Done: -5511.33893787 A.U.

P -1.921732 2.462897 0.434926

O -0.843115 3.340833 1.176423

O -1.665540 0.983424 0.957278

C -2.536521 -0.152265 0.639693

H -3.463187 -0.015171 1.199840

H -2.774281 -0.112406 -0.429881

C 0.566065 2.899244 1.327124

H 0.936021 2.644650 0.330472

H 0.553721 1.999327 1.941206

C 1.320293 4.025463 1.954804

H 0.913219 4.277061 2.931801

H 2.357613 3.722264 2.090509

H 1.308154 4.914932 1.328720

C -1.809231 -1.404745 1.014826

H -1.559677 -1.409608 2.073686

H -2.447313 -2.264817 0.816997

H -0.892721 -1.522695 0.439929

S -3.677823 3.373664 0.776781

S -1.478557 2.309542 -1.629779

C -2.224852 3.895702 -2.120622

H -1.818211 4.152526 -3.097809

H -1.951439 4.678263 -1.416315

S -4.053455 3.901547 -2.249891

C -4.273610 3.490096 -4.027957

H -5.299873 3.124124 -4.083231

H -3.596450 2.670357 -4.267247

C -4.054362 4.697410 -4.908051

H -4.214296 4.418629 -5.949139

H -4.744058 5.503387 -4.666404

H -3.038977 5.085568 -4.833089

Ni -4.904005 2.327935 -0.853314

P -7.415020 0.093776 0.295777

O -6.917008 -1.396706 0.108905

O -8.624991 0.010143 1.311570

C -9.339144 1.189134 1.849496

H -8.587217 1.928199 2.133326

H -9.948029 1.590645 1.036231

C -7.871584 -2.501905 -0.147201

H -8.667464 -2.121334 -0.793272

H -8.305760 -2.765872 0.816385

C -7.103780 -3.621479 -0.772699

H -6.309292 -3.971721 -0.116942

H -7.775434 -4.457554 -0.961242

H -6.664269 -3.321722 -1.723828

C -10.165817 0.728361 3.006027

H -9.543923 0.322933 3.801247

H -10.717197 1.577179 3.407788

H -10.887726 -0.026470 2.701829

S -5.936144 1.285905 0.935562

S -8.300432 0.730260 -1.526898

C -6.996172 0.279163 -2.720170

H -7.160174 0.924172 -3.583403

H -7.130799 -0.762453 -3.007690

S -5.263445 0.454238 -2.129825

C -4.374199 0.139515 -3.716893

H -3.358237 0.478104 -3.504710

H -4.796935 0.781339 -4.488985

C -4.407087 -1.321365 -4.096372

H -3.785443 -1.474424 -4.978216

H -4.018402 -1.954056 -3.300189

H -5.409413 -1.661780 -4.351669

O -6.840274 3.613873 -1.534816

H -7.675530 3.150936 -1.382877

H -6.987096 4.516075 -1.228849

P1-2LIGAND2222cis+1OH2ax-NiIIt

64

P1-2LIGAND2222cis+1OH2ax-NiIIt SCF Done: -5511.37218841 A.U.

P -1.912741 1.644141 0.434447

O -1.143759 2.392104 1.595579

O -1.205549 0.221232 0.343158

C -1.726976 -0.892229 -0.451304

H -2.781075 -1.024045 -0.185521

H -1.668981 -0.609261 -1.505562

C 0.335719 2.355122 1.696233

H 0.742352 2.528634 0.696412

H 0.602737 1.347340 2.012750

C 0.745106 3.405153 2.677161

H 0.300851 3.228458 3.654237

H 1.827872 3.380772 2.790884

H 0.465088 4.401525 2.339983

C -0.899803 -2.099596 -0.138894

H -0.961299 -2.358010 0.916104

H -1.262407 -2.947345 -0.718131

H 0.144573 -1.939135 -0.398133

S -3.856234 1.650514 0.880022

S -1.426988 2.559504 -1.402939

C -2.249293 4.156789 -1.053958

H -1.662112 4.928805 -1.547692

H -2.249144 4.365794 0.014732

S -3.968127 4.311497 -1.632950

C -3.708185 4.255248 -3.453747

H -4.699906 4.045831 -3.854522

H -3.070932 3.387297 -3.644442

C -3.123819 5.532686 -4.003270

H -3.019248 5.444286 -5.084019

H -3.760525 6.390502 -3.797934

H -2.131650 5.740418 -3.604680

Ni -5.238932 2.395764 -0.940172

P -8.182475 0.416642 -0.258166

O -8.013413 -1.031073 0.391543

O -9.702671 0.848795 -0.271874

C -10.810569 -0.137106 -0.392786

H -11.509461 0.348120 -1.070367

H -10.425571 -1.039389 -0.871865

C -7.582263 -1.249693 1.788059

H -8.220690 -2.060783 2.132611

H -7.803935 -0.356116 2.373534

C -6.127525 -1.602756 1.812130

H -5.515449 -0.770697 1.454732

H -5.818312 -1.820434 2.833611

H -5.927672 -2.487588 1.208018

C -11.396613 -0.401077 0.957526

H -11.738223 0.519423 1.426238

H -12.255035 -1.063259 0.852117

H -10.681582 -0.888977 1.618809

S -7.084713 1.833108 0.577045

S -7.753970 -0.010452 -2.284086

C -6.044311 -0.626574 -2.042148

H -5.914275 -1.484473 -2.698699

H -5.918088 -0.959774 -1.011290

S -4.702145 0.562371 -2.378504

C -5.024664 0.956272 -4.152599

H -4.179509 1.589008 -4.420284

H -5.926526 1.566401 -4.208848

C -5.100130 -0.268431 -5.029894

H -5.144478 0.044444 -6.072261

H -4.227864 -0.910243 -4.915373

H -5.995252 -0.860437 -4.843395

O -6.701058 3.189452 -2.334268

H -7.335897 2.482300 -2.541862

H -7.243911 3.925450 -2.021549

P1-2LIGAND2222cis+1OH2ax-ZnII

64

P1-2LIGAND2222cis+1OH2ax-ZnII SCF Done: -5782.41863886 A.U.

P -1.582845 2.211483 0.557691

O -0.730527 3.182163 1.462211

O -0.876946 0.792712 0.698473

C -1.468507 -0.457230 0.222809

H -2.515140 -0.481486 0.550396

H -1.443612 -0.441918 -0.870139

C 0.753836 3.165358 1.446970

H 1.076141 3.195154 0.403114

H 1.061663 2.215719 1.883664

C 1.221383 4.347567 2.231562

H 0.860183 4.309040 3.256965

H 2.310093 4.344694 2.258506

H 0.898173 5.283351 1.780068

C -0.671805 -1.587054 0.796219

H -0.707487 -1.582763 1.883403

H -1.080439 -2.533875 0.446559

H 0.367761 -1.533570 0.480182

S -3.461174 2.373577 1.228641

S -1.346053 2.699256 -1.470408

C -2.356565 4.245356 -1.442744

H -1.910007 4.912249 -2.177684

H -2.269964 4.715980 -0.465949

S -4.116043 4.043737 -1.828528

C -4.101780 4.323690 -3.652238

H -5.065289 3.938281 -3.987153

H -3.323441 3.688218 -4.080487

C -3.921049 5.775321 -4.018991

H -3.940722 5.882098 -5.103265

H -4.712596 6.395463 -3.603652

H -2.966241 6.173093 -3.676450

Zn -4.785067 1.649336 -0.572428

P -7.436715 -0.638401 -0.265289

O -8.248804 -1.918881 0.200864

O -8.473863 0.605473 -0.344970

C -9.789809 0.439166 -1.020282

H -9.881546 1.322087 -1.650917

H -9.742755 -0.438101 -1.669579

C -8.601487 -2.124354 1.629348

H -9.004932 -1.186188 2.017959

H -7.671234 -2.346326 2.153855

C -9.585247 -3.247212 1.688010

H -9.166459 -4.162376 1.275184

H -9.847712 -3.435134 2.728124

H -10.500180 -3.009044 1.148323

C -10.881597 0.333818 -0.003476

H -10.879640 1.186979 0.672100

H -11.844572 0.311703 -0.512510

H -10.798894 -0.580478 0.581843

S -5.901076 -0.132551 0.858008

S -6.982980 -1.076031 -2.278520

C -5.188897 -1.368248 -2.142107

H -4.936748 -2.060687 -2.942681

H -4.939560 -1.830407 -1.189080

S -4.087154 0.088923 -2.310082

C -4.657503 0.803785 -3.909929

H -3.977548 1.643512 -4.047902

H -5.663068 1.199554 -3.754605

C -4.586676 -0.173465 -5.055010

H -4.801800 0.350809 -5.985493

H -3.600100 -0.624283 -5.149576

H -5.325321 -0.968883 -4.967055

O -6.622078 2.591947 -0.895771

H -7.423678 2.125505 -0.585107

H -6.717936 3.525042 -0.671306

P1-2LIGAND2222cis+2OH2-CoIIq

67

P1-2LIGAND2222cis+2OH2-CoIIq SCF Done: -5462.28958756 A.U.

P -2.114759 1.493396 0.121301

O -1.580972 2.989530 0.172717

O -1.283060 0.724777 1.228490

C -1.519128 -0.686689 1.591371

H -2.571817 -0.782087 1.864744

H -1.326171 -1.292985 0.702938

C -0.137250 3.292662 0.316261

H 0.416279 2.653338 -0.377517

H 0.145350 3.017445 1.331610

C 0.034968 4.750512 0.032725

H -0.526521 5.357375 0.741026

H 1.086764 5.015964 0.127598

H -0.288246 5.004224 -0.976354

C -0.597110 -1.023792 2.718434

H -0.790659 -0.398912 3.587627

H -0.754980 -2.061702 3.007635

H 0.445062 -0.911326 2.427087

S -4.079568 1.433634 0.381666

S -1.492968 0.672131 -1.745228

C -1.486384 2.199838 -2.762430

H -1.345987 1.841468 -3.781483

H -0.621554 2.795199 -2.481528

S -2.970657 3.274528 -2.717905

C -3.273557 3.512673 -4.521390

H -4.200911 4.087228 -4.533864

H -3.494772 2.534695 -4.952730

C -2.147530 4.231311 -5.218997

H -2.410224 4.385118 -6.265259

H -1.955299 5.206318 -4.774683

H -1.217938 3.662519 -5.206497

Co -5.062138 2.108445 -1.751028

P -7.921278 3.727686 -3.503091

O -7.473257 5.227404 -3.772500

O -9.247413 3.547275 -4.354251

C -9.932924 2.256553 -4.537876

H -9.187800 1.530012 -4.867753

H -10.320671 1.949153 -3.563210

C -8.461048 6.327820 -3.790981

H -9.161497 6.172617 -2.965070

H -9.013329 6.242084 -4.726244

C -7.705071 7.613130 -3.675598

H -7.005916 7.735372 -4.500232

H -8.405733 8.446214 -3.703710

H -7.149971 7.672559 -2.739521

C -11.022305 2.464034 -5.541103

H -10.619404 2.781476 -6.500525

H -11.552706 1.525090 -5.691633

H -11.741367 3.205115 -5.198676

S -6.486973 2.435877 -3.887275

S -8.604234 3.673426 -1.469643

C -7.338923 4.726762 -0.672592

H -7.427349 4.526584 0.394877

H -7.587717 5.768229 -0.865189

S -5.599312 4.462609 -1.197903

C -4.678273 4.938312 0.326498

H -3.652415 4.652726 0.081846

H -5.012295 4.286876 1.134718

C -4.805282 6.404793 0.651354

H -4.183447 6.638630 1.515083

H -4.477380 7.031672 -0.176322

H -5.825864 6.684136 0.910369

O -6.817747 0.995461 -1.015785

H -6.859656 0.559384 -0.156995

H -7.640792 1.507480 -1.097636

O -4.592741 0.316348 -2.764196

H -3.727535 -0.074724 -2.562214

H -5.247865 -0.391864 -2.744342

P1-2LIGAND2222cis+2OH2-CuIId

67

P1-2LIGAND2222cis+2OH2-CuIId SCF Done: -5719.99642019 A.U.

P -2.555447 1.899085 -0.022702

O -1.855794 3.229422 0.500874

O -2.176914 0.808590 1.067974

C -2.667937 -0.577855 1.034868

H -3.756239 -0.540769 0.950888

H -2.258006 -1.052177 0.139144

C -0.495393 3.214410 1.088077

H 0.158518 2.651732 0.416256

H -0.562302 2.677468 2.033405

C -0.073433 4.639526 1.253847

H -0.757886 5.178324 1.906975

H 0.915906 4.673352 1.707396

H -0.017787 5.159515 0.297522

C -2.209346 -1.250464 2.289421

H -2.616183 -0.760244 3.171426

H -2.552611 -2.283687 2.288327

H -1.123803 -1.258027 2.360025

S -4.504536 2.169836 -0.261048

S -1.497587 1.285353 -1.765960

C -1.172302 2.932186 -2.535132

H -0.292627 2.773324 -3.156919

H -0.908864 3.637609 -1.750301

S -2.465108 3.704846 -3.547113

C -2.331106 2.722509 -5.102449

H -3.249612 2.960370 -5.639280

H -2.388757 1.669605 -4.824055

C -1.099472 3.057670 -5.903973

H -1.090895 2.461902 -6.816142

H -1.075122 4.107126 -6.189091

H -0.177963 2.828483 -5.368921

Cu -4.703087 3.006377 -2.549976

P -7.924136 4.045889 -3.145930

O -8.081224 5.609319 -3.361262

O -9.357098 3.448292 -3.474658

C -9.637564 2.015341 -3.646482

H -8.835201 1.590761 -4.253697

H -9.607214 1.555379 -2.655864

C -9.279519 6.338605 -2.891647

H -9.522533 5.985718 -1.884968

H -10.097781 6.064296 -3.556819

C -8.956842 7.798007 -2.934682

H -8.703987 8.115602 -3.943955

H -9.825245 8.368528 -2.609322

H -8.125256 8.045780 -2.275647

C -10.982092 1.894448 -4.291046

H -10.992716 2.361851 -5.273507

H -11.228351 0.840933 -4.415367

H -11.757249 2.349222 -3.677847

S -6.432013 3.318239 -4.226172

S -7.651654 3.725514 -1.061520

C -6.507218 5.101591 -0.712958

H -6.129572 4.904624 0.290110

H -7.051689 6.044176 -0.725695

S -5.069191 5.281626 -1.847277

C -3.762114 5.731612 -0.630862

H -2.841101 5.675519 -1.215076

H -3.730782 4.944690 0.123444

C -3.971558 7.109101 -0.048914

H -3.140383 7.347175 0.614452

H -4.011419 7.876149 -0.820015

H -4.882422 7.170554 0.545233

O -6.688524 -0.338218 -2.908202

H -6.854256 -0.849036 -2.109823

H -6.983217 -0.903686 -3.629578

O -4.459052 1.124091 -3.314235

H -3.712170 0.681101 -2.881831

H -5.255188 0.554949 -3.162729

P1-2LIGAND2222cis+2OH2-FeIIId

67

P1-2LIGAND2222cis+2OH2-FeIIId SCF Done: -5342.76369788 A.U.

P -2.001345 1.575252 0.057821

O -1.322914 2.991891 0.085212

O -1.360573 0.713769 1.193343

C -1.652654 -0.719143 1.538749

H -2.720271 -0.770940 1.755299

H -1.426871 -1.307392 0.647500

C 0.172046 3.155664 0.226651

H 0.644060 2.396160 -0.400862

H 0.392924 2.941413 1.270641

C 0.503791 4.550599 -0.180894

H 0.004533 5.281309 0.452027

H 1.577713 4.699021 -0.071748

H 0.246907 4.743512 -1.221873

C -0.794407 -1.077826 2.703496

H -1.014561 -0.457982 3.569445

H -0.997440 -2.113900 2.972958

H 0.263133 -0.998886 2.462337

S -4.016506 1.795007 0.284716

S -1.572635 0.611401 -1.778379

C -1.763543 2.015801 -2.932103

H -1.920832 1.557257 -3.908506

H -0.837194 2.587890 -2.946877

S -3.114248 3.239973 -2.628215

C -3.375912 3.805091 -4.376154

H -4.221769 4.488287 -4.285775

H -3.703082 2.939096 -4.952319

C -2.144787 4.470727 -4.936421

H -2.389053 4.868387 -5.921460

H -1.807709 5.303142 -4.320918

H -1.316932 3.777370 -5.076189

Fe -5.050493 2.238748 -1.724473

P -7.911740 3.711381 -3.471592

O -7.468357 5.196087 -3.748495

O -9.121193 3.400586 -4.414986

C -9.843302 2.094003 -4.559447

H -9.088358 1.350400 -4.817642

H -10.268616 1.862613 -3.581037

C -8.470850 6.310131 -3.883235

H -9.250950 6.142979 -3.136491

H -8.903861 6.200514 -4.875944

C -7.736551 7.594071 -3.691959

H -6.956066 7.722830 -4.438463

H -8.439292 8.419284 -3.802166

H -7.294184 7.665718 -2.698514

C -10.880612 2.275115 -5.615289

H -10.434995 2.529675 -6.574268

H -11.418254 1.334984 -5.734890

H -11.603810 3.039271 -5.339834

S -6.338776 2.473415 -3.734785

S -8.667408 3.608115 -1.480261

C -7.392186 4.569397 -0.593213

H -7.446377 4.248778 0.447220

H -7.641607 5.628196 -0.656014

S -5.645368 4.443891 -1.177915

C -4.755824 5.061460 0.330841

H -3.707537 4.933580 0.054146

H -4.978607 4.374623 1.147295

C -5.110198 6.495371 0.632730

H -4.474253 6.838829 1.448907

H -4.936328 7.153905 -0.216659

H -6.139145 6.612482 0.968495

O -6.655660 1.238620 -0.900387

H -6.630052 0.901855 0.005580

H -7.536564 1.640656 -1.033272

O -4.625935 0.453829 -2.566405

H -3.786468 0.033333 -2.297984

H -5.329316 -0.207531 -2.492082

P1-2LIGAND2222cis+2OH2-FeIIIs

67

P1-2LIGAND2222cis+2OH2-FeIIIs SCF Done: -5342.79390416 A.U.

P -1.864564 1.536967 0.063481

O -1.165360 2.941738 0.155964

O -1.168618 0.594107 1.101303

C -1.528916 -0.825900 1.408210

H -2.591590 -0.830570 1.655850

H -1.361448 -1.395116 0.491743

C 0.327773 3.072906 0.340591

H 0.803490 2.369712 -0.346748

H 0.528789 2.759211 1.363251

C 0.681504 4.495865 0.073408

H 0.178146 5.169090 0.763851

H 1.754913 4.618611 0.215058

H 0.446851 4.791236 -0.948408

C -0.659108 -1.271695 2.534693

H -0.823561 -0.672427 3.427312

H -0.905672 -2.305361 2.774321

H 0.393925 -1.234847 2.265695

S -3.851771 1.757100 0.396650

S -1.507074 0.720022 -1.863794

C -1.582549 2.259515 -2.843595

H -1.521594 1.931175 -3.880853

H -0.712757 2.869262 -2.609639

S -3.057740 3.361680 -2.677486

C -3.405688 3.770999 -4.455040

H -4.319146 4.364388 -4.385205

H -3.650890 2.834316 -4.957974

C -2.273897 4.525386 -5.101088

H -2.565959 4.782400 -6.119389

H -2.046605 5.451735 -4.577095

H -1.362041 3.934797 -5.177196

Fe -5.104297 2.231567 -1.629606

P -7.994804 3.677385 -3.464616

O -7.464895 5.109333 -3.845045

O -9.216681 3.366424 -4.391471

C -9.982648 2.081339 -4.477690

H -9.256574 1.307402 -4.730094

H -10.392041 1.892016 -3.483606

C -8.418601 6.240760 -4.142598

H -9.202782 6.212849 -3.382213

H -8.858400 6.012147 -5.111698

C -7.625526 7.503065 -4.131058

H -6.844827 7.490976 -4.888472

H -8.292508 8.334424 -4.357523

H -7.175481 7.695016 -3.157720

C -11.038054 2.261800 -5.515632

H -10.607064 2.474196 -6.491361

H -11.605659 1.335191 -5.595091

H -11.732203 3.055052 -5.247832

S -6.492528 2.332528 -3.642108

S -8.719953 3.744772 -1.468866

C -7.505778 4.914411 -0.760170

H -7.666775 4.862393 0.316481

H -7.739117 5.917660 -1.110330

S -5.706996 4.649120 -1.084534

C -4.994645 5.092107 0.573779

H -3.932855 4.875872 0.442508

H -5.398528 4.381797 1.296482

C -5.252545 6.530854 0.937043

H -4.760662 6.741783 1.886825

H -4.851578 7.219593 0.196081

H -6.310900 6.745894 1.078522

O -6.703376 1.488731 -0.431171

H -6.720841 0.740431 0.178449

H -7.617461 1.781378 -0.595206

O -4.652183 0.319820 -2.454134

H -3.776095 -0.083871 -2.329297

H -5.182204 -0.259374 -3.015886

P1-2LIGAND2222cis+2OH2-MnIId

67

P1-2LIGAND2222cis+2OH2-MnIId SCF Done: -5230.48243777 A.U.

P -2.088316 1.522757 0.106474

O -1.483495 2.990409 0.076302

O -1.245310 0.753935 1.205335

C -1.536320 -0.622940 1.650032

H -2.588202 -0.659837 1.939361

H -1.379939 -1.284719 0.794816

C -0.022883 3.228790 0.050031

H 0.433241 2.492253 -0.617176

H 0.343419 3.048212 1.060119

C 0.190262 4.636693 -0.407306

H -0.287234 5.346056 0.266153

H 1.256842 4.855443 -0.419942

H -0.199709 4.794645 -1.412511

C -0.613947 -0.935454 2.784110

H -0.771969 -0.257894 3.620439

H -0.807378 -1.949641 3.130193

H 0.427862 -0.879033 2.476156

S -4.041520 1.547766 0.411684

S -1.570895 0.571973 -1.727732

C -1.795756 1.935679 -2.924310

H -1.969334 1.441896 -3.880586

H -0.871529 2.507564 -2.978359

S -3.171080 3.128889 -2.617363

C -3.449257 3.671953 -4.361126

H -4.309430 4.337444 -4.270919

H -3.770062 2.799853 -4.932166

C -2.247646 4.361193 -4.955915

H -2.503611 4.741090 -5.944499

H -1.922273 5.206010 -4.350252

H -1.402610 3.686194 -5.087419

Mn -5.112372 2.134925 -1.714361

P -7.869505 3.666639 -3.499457

O -7.319877 5.126454 -3.794758

O -9.159735 3.528499 -4.409899

C -9.932946 2.280351 -4.548156

H -9.237225 1.493101 -4.844364

H -10.345292 2.036996 -3.565635

C -8.227004 6.293259 -3.849874

H -8.990622 6.170238 -3.076364

H -8.718501 6.264727 -4.821730

C -7.391994 7.518200 -3.652248

H -6.641184 7.614285 -4.433826

H -8.028469 8.400769 -3.688731

H -6.885651 7.505911 -2.686842

C -11.000657 2.522283 -5.566355

H -10.572930 2.775827 -6.534074

H -11.591069 1.615205 -5.685701

H -11.671122 3.321318 -5.257049

S -6.499791 2.273418 -3.766506

S -8.633871 3.705653 -1.497088

C -7.350723 4.717947 -0.683828

H -7.481848 4.547900 0.384949

H -7.540776 5.766727 -0.903646

S -5.597553 4.363760 -1.146619

C -4.746035 4.917933 0.393881

H -3.705933 4.648864 0.198563

H -5.094794 4.289674 1.213886

C -4.924289 6.391727 0.656331

H -4.331288 6.678911 1.523887

H -4.594739 6.996284 -0.187518

H -5.958271 6.651227 0.881531

O -6.768544 1.166644 -0.889984

H -6.765519 0.836916 0.016606

H -7.612972 1.638296 -1.017760

O -4.665703 0.286974 -2.502102

H -3.862921 -0.148692 -2.169074

H -5.379426 -0.363188 -2.500693

P1-2LIGAND2222cis+2OH2-MnIIs

67

P1-2LIGAND2222cis+2OH2-MnIIs SCF Done: -5230.56855461 A.U.

P -1.911718 1.521049 0.135673

O -1.170266 2.923482 0.193946

O -1.086702 0.590349 1.121046

C -1.486973 -0.781765 1.470562

H -2.539740 -0.757209 1.759801

H -1.379877 -1.393463 0.571201

C 0.306963 3.014072 0.242788

H 0.717060 2.341999 -0.516525

H 0.615455 2.649096 1.221831

C 0.671744 4.445344 0.011146

H 0.247602 5.088565 0.779396

H 1.754935 4.550259 0.046770

H 0.330647 4.796266 -0.962231

C -0.595224 -1.244062 2.578590

H -0.706240 -0.617589 3.461138

H -0.862746 -2.263451 2.851830

H 0.449054 -1.241243 2.274094

S -3.826006 1.697844 0.574434

S -1.529656 0.713634 -1.807838

C -1.548557 2.252158 -2.805760

H -1.465836 1.906379 -3.835411

H -0.661816 2.829406 -2.558152

S -3.004018 3.362336 -2.678685

C -3.406291 3.637880 -4.458909

H -4.338928 4.203190 -4.409952

H -3.640891 2.667240 -4.898942

C -2.326438 4.382018 -5.200791

H -2.643851 4.549234 -6.229793

H -2.122552 5.351550 -4.750102

H -1.391520 3.823752 -5.244006

Mn -5.140372 2.156753 -1.604716

P -7.992748 3.666353 -3.488538

O -7.450138 5.107130 -3.885043

O -9.314594 3.489477 -4.347857

C -10.059398 2.223080 -4.448383

H -9.350975 1.446789 -4.744138

H -10.447137 1.987609 -3.454027

C -8.379893 6.239798 -4.093546

H -9.097649 6.249236 -3.267924

H -8.926091 6.037968 -5.014374

C -7.557497 7.486681 -4.164105

H -6.850070 7.445362 -4.989773

H -8.212959 8.340823 -4.327237

H -7.003639 7.658409 -3.241724

C -11.151983 2.421110 -5.449951

H -10.748794 2.666651 -6.430239

H -11.723653 1.498971 -5.541802

H -11.834718 3.210287 -5.142112

S -6.630250 2.268372 -3.749525

S -8.680435 3.838003 -1.466469

C -7.465451 5.054963 -0.828854

H -7.644736 5.083114 0.245314

H -7.703890 6.028402 -1.250741

S -5.682222 4.728329 -1.116057

C -4.979471 5.117183 0.546070

H -3.930871 4.833154 0.439670

H -5.428334 4.431506 1.266469

C -5.140943 6.566083 0.927867

H -4.666617 6.742419 1.892890

H -4.676549 7.228395 0.199421

H -6.187185 6.853356 1.030969

O -6.837549 1.379955 -0.437141

H -6.882825 0.800074 0.329784

H -7.707087 1.795427 -0.554921

O -4.624549 0.216724 -2.514206

H -3.742966 -0.160373 -2.366969

H -5.202606 -0.482753 -2.836217

P1-2LIGAND2222cis+2OH2-NiIIt

67

P1-2LIGAND2222cis+2OH2-NiIIt SCF Done: -5587.84570165 A.U.

P -2.115412 1.664351 0.304903

O -1.415096 3.090105 0.334179

O -1.390914 0.811644 1.429093

C -1.976839 -0.368145 2.081431

H -1.280787 -0.569312 2.891817

H -2.934087 -0.067585 2.510343

C 0.053805 3.227006 0.453160

H 0.520285 2.476546 -0.191452

H 0.310689 2.996194 1.486329

C 0.403129 4.627607 0.062179

H -0.076376 5.351157 0.718434

H 1.480185 4.766313 0.141106

H 0.108718 4.842622 -0.964687

C -2.104736 -1.518398 1.129415

H -1.148342 -1.763289 0.669304

H -2.455284 -2.400474 1.662892

H -2.832596 -1.304146 0.342378

S -4.072594 1.822298 0.514230

S -1.512515 0.759285 -1.526995

C -1.544485 2.209681 -2.644282

H -1.521243 1.780135 -3.645617

H -0.637267 2.788608 -2.485179

S -2.966945 3.368815 -2.542509

C -3.166537 3.767105 -4.332236

H -4.063560 4.387814 -4.349569

H -3.406414 2.836347 -4.848923

C -1.966572 4.474642 -4.908185

H -2.167665 4.734611 -5.946888

H -1.742045 5.395049 -4.371487

H -1.074135 3.849766 -4.905033

Ni -5.049237 2.283149 -1.729108

P -7.760423 3.725752 -3.712426

O -7.303453 5.216717 -4.020608

O -9.010465 3.472314 -4.657380

C -9.476471 2.141130 -5.070901

H -10.230508 2.368714 -5.819950

H -8.641970 1.634604 -5.558182

C -8.299234 6.292822 -4.222156

H -9.061910 6.199680 -3.443433

H -8.773170 6.111405 -5.186200

C -7.568547 7.596273 -4.167976

H -6.811817 7.656768 -4.947354

H -8.274605 8.410680 -4.322711

H -7.085410 7.748280 -3.203254

C -10.027500 1.362694 -3.914629

H -10.821997 1.908558 -3.407476

H -10.443457 0.421482 -4.270243

H -9.244638 1.117787 -3.192188

S -6.289672 2.429228 -3.904655

S -8.605901 3.785734 -1.744956

C -7.435404 4.926226 -0.927044

H -7.644759 4.830198 0.138144

H -7.661184 5.942575 -1.242297

S -5.642508 4.639937 -1.206864

C -4.969255 5.165731 0.425785

H -3.911658 4.907028 0.350484

H -5.405341 4.520874 1.189646

C -5.185650 6.633444 0.695801

H -4.712361 6.900233 1.640307

H -4.750689 7.256501 -0.083922

H -6.241476 6.886962 0.787106

O -6.757484 1.255455 -0.960863

H -6.785646 0.949411 -0.046421

H -7.580112 1.758276 -1.108199

O -4.524543 0.406596 -2.527537

H -3.708313 0.016020 -2.173435

H -5.216827 -0.262446 -2.464040

P1-2LIGAND2222cis+2OH2-ZnII

67

P1-2LIGAND2222cis+2OH2-ZnII SCF Done: -5858.87895433 A.U.

P -1.951700 1.515442 0.100608

O -1.209791 2.914644 0.205161

O -1.164588 0.565843 1.097458

C -1.577216 -0.813014 1.408446

H -2.634176 -0.788469 1.681463

H -1.459044 -1.404342 0.496899

C 0.266667 3.000296 0.291385

H 0.692984 2.333263 -0.463264

H 0.548811 2.626386 1.274978

C 0.641698 4.432036 0.080804

H 0.199288 5.070948 0.842363

H 1.723818 4.533173 0.146429

H 0.328648 4.791388 -0.898924

C -0.705053 -1.304953 2.519032

H -0.825009 -0.696853 3.413186

H -0.983532 -2.328079 2.766130

H 0.343549 -1.302991 2.229875

S -3.880854 1.690422 0.476725

S -1.515372 0.739055 -1.841299

C -1.543665 2.287504 -2.822820

H -1.462339 1.951892 -3.855978

H -0.659170 2.866519 -2.570820

S -2.996268 3.403066 -2.686722

C -3.381803 3.703947 -4.465699

H -4.312375 4.272451 -4.416645

H -3.616491 2.738944 -4.917825

C -2.291401 4.452762 -5.187090

H -2.600771 4.636333 -6.215643

H -2.086225 5.415121 -4.721770

H -1.359192 3.890267 -5.232099

Zn -5.079766 2.226500 -1.696359

P -7.955150 3.694043 -3.488010

O -7.490450 5.174919 -3.829623

O -9.277924 3.486143 -4.337935

C -9.970486 2.192069 -4.465874

H -9.231955 1.452383 -4.780691

H -10.345018 1.919498 -3.475800

C -8.477699 6.269738 -3.963621

H -9.178146 6.201154 -3.126070

H -9.031023 6.088077 -4.884325

C -7.719231 7.558273 -3.984284

H -7.026500 7.593853 -4.822470

H -8.419481 8.385058 -4.093360

H -7.157369 7.712246 -3.063508

C -11.073617 2.367677 -5.459911

H -10.683953 2.650898 -6.435467

H -11.607373 1.425081 -5.570703

H -11.786810 3.121342 -5.132941

S -6.537849 2.365575 -3.803724

S -8.633968 3.746489 -1.451517

C -7.428975 4.934638 -0.750163

H -7.577358 4.876668 0.327672

H -7.693019 5.933911 -1.088699

S -5.649069 4.669160 -1.114825

C -4.895535 5.098190 0.512766

H -3.843110 4.847048 0.369881

H -5.294223 4.409531 1.258568

C -5.092142 6.546855 0.880541

H -4.580245 6.754780 1.819734

H -4.685831 7.214321 0.122637

H -6.142287 6.797288 1.028800

O -6.732201 1.212649 -0.756239

H -6.708999 0.826230 0.126707

H -7.576888 1.690704 -0.838491

O -4.586644 0.372051 -2.643066

H -3.727701 -0.018434 -2.412746

H -5.240659 -0.336465 -2.652216

P1-2LIGAND2222cistrans+1H2Oeq-CoIId

P -2.274741 1.546368 0.244221

O -1.219592 2.687654 0.530122

O -1.809291 0.325135 1.148033

C -2.585754 -0.911938 1.295065

H -3.576619 -0.634126 1.664656

H -2.695598 -1.362026 0.303601

C 0.233003 2.399336 0.630370

H 0.505657 1.755484 -0.210486

H 0.379571 1.841672 1.554621

C 0.950067 3.710197 0.618795

H 0.639670 4.337095 1.451763

H 2.020207 3.531705 0.713205

H 0.781438 4.255261 -0.308787

C -1.845022 -1.801638 2.242379

H -1.734967 -1.333041 3.217864

H -2.399074 -2.729809 2.373769

H -0.857498 -2.050702 1.859634

S -4.130595 2.194094 0.601580

S -2.019223 0.857802 -1.744791

C -1.909346 2.467226 -2.591311

H -1.950430 2.231433 -3.654488

H -0.964040 2.953320 -2.358636

S -3.287256 3.617490 -2.192563

C -3.560145 4.392103 -3.843492

H -4.498899 4.932930 -3.714159

H -3.732375 3.592301 -4.564878

C -2.425311 5.305384 -4.235369

H -2.663478 5.785027 -5.184235

H -2.260286 6.088958 -3.498514

H -1.490268 4.763991 -4.375803

Co -5.046122 2.236261 -1.543110

P -6.117496 -0.113246 -3.804062

O -7.681814 -0.308491 -3.929943

O -5.508697 -0.897742 -5.042723

C -4.080415 -0.896924 -5.384852

H -3.751441 0.144800 -5.428120

H -3.546001 -1.401807 -4.575525

C -8.270972 -1.627694 -4.269395

H -7.779310 -2.383523 -3.650244

H -8.024843 -1.822254 -5.312552

C -9.741894 -1.537038 -4.022153

H -10.197684 -0.761890 -4.634302

H -10.206218 -2.487244 -4.282233

H -9.963180 -1.329896 -2.976228

C -3.927674 -1.601783 -6.695342

H -4.477893 -1.091716 -7.483027

H -2.875469 -1.619053 -6.974338

H -4.275729 -2.630580 -6.633918

S -5.644363 1.829248 -3.758623

S -5.444627 -1.177855 -2.097902

C -6.718590 -0.640054 -0.913592

H -6.359473 -0.971876 0.060437

H -7.665673 -1.127719 -1.136029

S -7.000481 1.175009 -0.853717

C -7.325335 1.383366 0.948448

H -7.334725 2.466356 1.079446

H -6.465579 0.988764 1.491237

C -8.627735 0.747021 1.366505

H -8.805142 0.954768 2.421152

H -9.472476 1.138952 0.803075

H -8.612107 -0.336406 1.252770

O -6.249268 4.119513 -1.301842

H -7.027698 4.358725 -1.815893

H -6.085238 4.850874 -0.697251

P1-2LIGAND2222cistrans+1H2Oeq-CoIIq

64

P1-2LIGAND2222cistrans+1H2Oeq-CoIIq SCF Done: -5385.83154445 A.U.

P -1.712082 2.043934 0.236445

O -1.173610 2.794407 1.516219

O -0.754470 0.787052 0.067646

C -1.035577 -0.314402 -0.861017

H -2.024149 -0.708832 -0.611377

H -1.069918 0.098490 -1.873399

C 0.276691 3.034015 1.723343

H 0.661971 3.516866 0.821569

H 0.747583 2.056912 1.828637

C 0.418629 3.885855 2.942336

H 0.002528 3.393781 3.818879

H 1.477095 4.063168 3.127437

H -0.067576 4.851119 2.817174

C 0.049499 -1.331201 -0.695459

H 0.074111 -1.717882 0.321165

H -0.131563 -2.165821 -1.370877

H 1.024759 -0.912037 -0.933984

S -3.649003 1.667844 0.560981

S -1.327851 3.165898 -1.499606

C -2.614997 4.454210 -1.283615

H -2.193906 5.379690 -1.671289

H -2.843282 4.599967 -0.228917

S -4.198679 4.141723 -2.133600

C -3.611433 3.870739 -3.862297

H -4.520346 3.583781 -4.388651

H -2.950830 3.002127 -3.846705

C -2.953923 5.088872 -4.458404

H -2.718412 4.891696 -5.503622

H -3.600502 5.963742 -4.421619

H -2.013206 5.336172 -3.967327

Co -4.909252 1.939505 -1.413185

P -6.166673 -0.686936 -3.354033

O -6.974625 0.239226 -4.384037

O -6.008174 -2.064029 -4.117776

C -5.247916 -3.215790 -3.587115

H -4.224935 -2.876691 -3.413831

H -5.694428 -3.496555 -2.630519

C -8.165199 -0.289459 -5.101729

H -8.726475 -0.914607 -4.403398

H -7.785332 -0.915803 -5.907366

C -8.951417 0.884518 -5.590009

H -8.358374 1.512228 -6.253621

H -9.812321 0.532335 -6.155889

H -9.328851 1.490392 -4.765537

C -5.331995 -4.312258 -4.599114

H -4.896822 -4.008716 -5.548898

H -4.777288 -5.175392 -4.234305

H -6.361861 -4.621366 -4.765105

S -4.427944 0.163828 -2.886570

S -7.454966 -1.140501 -1.753001

C -8.058618 0.536381 -1.335765

H -9.005904 0.368295 -0.825797

H -8.249800 1.109644 -2.239896

S -7.026763 1.597962 -0.267339

C -6.726198 0.479885 1.165664

H -6.123230 1.090993 1.837354

H -6.101981 -0.343663 0.813042

C -7.995838 0.008130 1.827057

H -7.742991 -0.542023 2.732481

H -8.640609 0.837556 2.112945

H -8.565640 -0.673253 1.195934

O -6.571566 2.691223 -3.091248

H -6.764802 2.146233 -3.869541

H -7.080320 3.504368 -3.181608

P1-2LIGAND2222cistrans+1H2Oeq-CuIId

64

P1-2LIGAND2222cistrans+1H2Oeq-CuIId SCF Done: -5643.52860886 A.U.

P -2.362495 1.379386 0.190891

O -1.299019 2.471049 0.613581

O -1.907336 0.055645 0.941202

C -2.677961 -1.193795 0.928373

H -3.725345 -0.940464 1.116340

H -2.595480 -1.618245 -0.075776

C 0.148832 2.156059 0.688795

H 0.412113 1.562746 -0.191507

H 0.288822 1.538967 1.575281

C 0.884468 3.455065 0.757619

H 0.583045 4.033680 1.627947

H 1.952265 3.257165 0.838581

H 0.721990 4.058689 -0.134251

C -2.104585 -2.093649 1.976685

H -2.187109 -1.649460 2.966272

H -2.650922 -3.035555 1.980945

H -1.058023 -2.314115 1.778159

S -4.201889 2.002836 0.625410

S -2.092343 0.956078 -1.867495

C -1.910274 2.667646 -2.477814

H -1.824102 2.568864 -3.559115

H -0.993284 3.098421 -2.080973

S -3.298197 3.811781 -2.105891

C -3.573571 4.566453 -3.764106

H -4.534588 5.070818 -3.653812

H -3.704466 3.754259 -4.480680

C -2.469280 5.518849 -4.152345

H -2.705724 5.966596 -5.117227

H -2.354452 6.323855 -3.429348

H -1.508870 5.015240 -4.257994

Cu -5.147228 2.303137 -1.537316

P -5.989468 -0.137378 -3.767665

O -7.505875 -0.395156 -4.139155

O -5.166006 -1.013485 -4.805318

C -3.714262 -0.927773 -4.998302

H -3.438785 0.130284 -4.971109

H -3.242327 -1.437780 -4.154707

C -8.002481 -1.752618 -4.474815

H -7.613158 -2.447789 -3.725345

H -7.575962 -2.013641 -5.442643

C -9.495247 -1.688404 -4.491329

H -9.850375 -0.966904 -5.223765

H -9.891466 -2.665946 -4.762386

H -9.899110 -1.421528 -3.515699

C -3.388928 -1.574310 -6.307624

H -3.873032 -1.058826 -7.134209

H -2.312583 -1.538174 -6.468882

H -3.693890 -2.618325 -6.318890

S -5.597903 1.814958 -3.813544

S -5.624730 -1.023974 -1.877469

C -7.130019 -0.431091 -1.027502

H -7.079607 -0.861212 -0.028254

H -8.007750 -0.820003 -1.539780

S -7.319921 1.389936 -0.866666

C -7.514206 1.555317 0.957872

H -7.388612 2.625609 1.128762

H -6.673394 1.042638 1.427399

C -8.852594 1.052138 1.440492

H -8.925742 1.203929 2.516928

H -9.679277 1.580742 0.970558

H -8.981391 -0.014299 1.256659

O -6.270647 4.346229 -1.191962

H -7.069172 4.660595 -1.629507

H -6.028626 5.035790 -0.564262

P1-2LIGAND2222cistrans+1H2Oeq-FeIIId

64

P1-2LIGAND2222cistrans+1H2Oeq-FeIIId SCF Done: -5266.27715683 A.U.

P -1.979662 1.565925 0.133247

O -1.923948 2.337292 1.493840

O -1.261744 0.187356 0.333931

C -0.960579 -0.811110 -0.733615

H -1.396887 -1.732322 -0.352532

H -1.497644 -0.513175 -1.639042

C -0.665201 2.407413 2.341942

H 0.152704 2.669238 1.667988

H -0.515441 1.398228 2.721746

C -0.905955 3.420018 3.406129

H -1.749235 3.147702 4.036481

H -0.020051 3.462753 4.039776

H -1.067126 4.414773 2.996138

C 0.518826 -0.894993 -0.924465

H 1.021326 -1.159409 0.003462

H 0.738836 -1.674949 -1.652683

H 0.939992 0.036635 -1.300561

S -4.037660 1.334857 -0.173707

S -0.980678 2.546313 -1.406932

C -2.011918 4.060650 -1.376577

H -1.376901 4.875276 -1.722891

H -2.336117 4.297160 -0.364022

S -3.489805 4.028803 -2.435384

C -2.744281 3.808216 -4.119564

H -3.617715 3.768697 -4.771258

H -2.264220 2.827908 -4.132530

C -1.806440 4.930385 -4.482055

H -1.509124 4.803818 -5.523110

H -2.273043 5.909487 -4.391728

H -0.890208 4.919270 -3.893529

Fe -5.037929 2.335177 -1.827615

P -5.842891 -0.486825 -3.699034

O -7.287337 0.052877 -4.029206

O -5.334522 -1.414405 -4.847062

C -4.677345 -2.763152 -4.775832

H -3.963442 -2.721830 -5.594283

H -4.129395 -2.815710 -3.833031

C -8.376897 -0.869211 -4.519201

H -8.375107 -1.748993 -3.870802

H -8.083169 -1.165890 -5.525306

C -9.659781 -0.110987 -4.476693

H -9.623180 0.771446 -5.111535

H -10.454840 -0.754205 -4.853250

H -9.927851 0.184740 -3.463365

C -5.707011 -3.831390 -4.933786

H -6.256163 -3.718420 -5.866510

H -5.204277 -4.798225 -4.965359

H -6.407653 -3.853783 -4.099498

S -4.595383 1.167596 -3.634198

S -5.825791 -1.521605 -1.880743

C -7.104731 -0.557671 -0.996899

H -6.973728 -0.828741 0.050511

H -8.091993 -0.872932 -1.328724

S -7.074709 1.280226 -1.131494

C -7.340304 1.769015 0.642093

H -7.208415 2.852734 0.622803

H -6.520391 1.337468 1.217785

C -8.703258 1.365693 1.141990

H -8.812919 1.722206 2.166351

H -9.506232 1.803722 0.552696

H -8.836302 0.284853 1.167832

O -6.384357 3.740384 -2.433186

H -7.274722 3.528701 -2.747993

H -6.179637 4.642472 -2.717637

P1-2LIGAND2222cistrans+1H2Oeq-FeIIIq

64

P1-2LIGAND2222cistrans+1H2Oeq-FeIIIq SCF Done: -5266.30168825 A.U.

P -2.205428 1.444836 0.232274

O -1.355160 2.744318 0.433868

O -1.618750 0.334914 1.168306

C -2.084632 -1.078479 1.281996

H -3.147480 -1.036837 1.530160

H -1.955340 -1.531907 0.296454

C 0.147505 2.725283 0.622303

H 0.555054 2.020411 -0.105852

H 0.309560 2.336620 1.625896

C 0.629024 4.122906 0.437542

H 0.179415 4.799484 1.160497

H 1.707643 4.139719 0.592795

H 0.434307 4.494232 -0.568063

C -1.261174 -1.740196 2.335572

H -1.386555 -1.258692 3.302535

H -1.585849 -2.775782 2.431332

H -0.206283 -1.745847 2.071802

S -4.159161 1.882516 0.634644

S -2.028129 0.783194 -1.774909

C -1.859405 2.410161 -2.581362

H -1.913420 2.207029 -3.651122

H -0.897146 2.858999 -2.339317

S -3.178334 3.607641 -2.125465

C -3.469624 4.474713 -3.736739

H -4.369483 5.061382 -3.547521

H -3.700044 3.714853 -4.484828

C -2.286954 5.335493 -4.112248

H -2.533060 5.866893 -5.032049

H -2.060626 6.080264 -3.352113

H -1.391160 4.749355 -4.311773

Fe -5.035188 2.159947 -1.506833

P -6.070537 -0.254806 -3.796446

O -7.634502 -0.186532 -3.867838

O -5.563959 -1.044500 -5.050772

C -4.149157 -1.375941 -5.391286

H -3.594044 -0.435108 -5.369724

H -3.784206 -2.036259 -4.601403

C -8.488959 -1.361537 -4.289576

H -8.082055 -2.250198 -3.801152

H -8.350292 -1.450176 -5.365351

C -9.890440 -1.048970 -3.891677

H -10.250270 -0.143193 -4.374450

H -10.531832 -1.871103 -4.208460

H -9.996139 -0.944322 -2.812457

C -4.151284 -2.023836 -6.735167

H -4.540086 -1.355713 -7.500200

H -3.126003 -2.279083 -7.000339

H -4.732533 -2.942919 -6.733445

S -5.357264 1.664060 -3.761236

S -5.477471 -1.316416 -2.066185

C -6.800378 -0.763100 -0.944067

H -6.502642 -1.105850 0.046823

H -7.747260 -1.225015 -1.220637

S -7.080182 1.054471 -0.893861

C -7.470364 1.318726 0.899981

H -7.493206 2.405481 0.993522

H -6.625057 0.937881 1.475894

C -8.785016 0.679684 1.275114

H -8.993002 0.920065 2.318087

H -9.613073 1.057948 0.679007

H -8.759551 -0.406096 1.197691

O -6.228369 4.003644 -1.346824

H -7.081289 4.168582 -1.767809

H -6.015257 4.800251 -0.845225

P1-2LIGAND2222cistrans+1H2Oeq-FeIIIs

64

P1-2LIGAND2222cistrans+1H2Oeq-FeIIIs SCF Done: -5266.32789206 A.U.

P -1.912936 0.979597 -0.656737

O -2.811082 2.102818 0.080428

O -1.281004 0.050398 0.422777

C 0.151252 -0.429562 0.553286

H 0.020382 -1.466898 0.849431

H 0.596026 -0.400587 -0.442086

C -2.228175 2.830312 1.264115

H -1.224669 3.156276 0.980260

H -2.143493 2.087612 2.057471

C -3.127832 3.967103 1.615428

H -4.131204 3.625031 1.869547

H -2.727509 4.467255 2.496980

H -3.182848 4.706868 0.818543

C 0.869497 0.401028 1.560040

H 0.371369 0.381043 2.527644

H 1.866700 -0.017073 1.700009

H 0.995047 1.432917 1.232631

S -3.285778 0.025900 -1.811318

S -0.399505 1.927681 -1.734243

C -1.323197 3.443588 -2.214489

H -0.590716 4.028135 -2.771732

H -1.616606 4.011812 -1.334305

S -2.819093 3.285603 -3.242963

C -2.258425 2.228843 -4.652636

H -3.157297 2.156182 -5.266740

H -2.041223 1.239788 -4.243706

C -1.097391 2.832465 -5.399047

H -0.885881 2.213421 -6.270728

H -1.312224 3.838178 -5.755269

H -0.185945 2.854825 -4.803054

Fe -4.602483 1.961363 -1.896440

P -7.208096 0.126863 -3.619621

O -8.616737 0.673362 -4.011611

O -6.811862 -0.985109 -4.652181

C -5.518182 -1.718395 -4.719664

H -4.764480 -0.978190 -4.998622

H -5.302665 -2.095921 -3.716862

C -9.768886 -0.230639 -4.421772

H -9.846947 -1.003124 -3.654812

H -9.461898 -0.679419 -5.364883

C -10.980887 0.625721 -4.531222

H -10.853382 1.409615 -5.273954

H -11.810358 -0.004184 -4.853654

H -11.255204 1.071377 -3.577465

C -5.671546 -2.806756 -5.729792

H -5.912963 -2.406238 -6.711760

H -4.728990 -3.347065 -5.809525

H -6.439942 -3.518052 -5.435614

S -6.037067 1.824801 -3.684943

S -7.195156 -0.805765 -1.748155

C -7.592714 0.656953 -0.711457

H -8.235162 0.296715 0.091081

H -8.149167 1.403850 -1.276055

S -6.162721 1.493667 0.060422

C -5.461472 0.123455 1.089379

H -4.513221 0.534901 1.437440

H -5.237409 -0.696386 0.403342

C -6.369817 -0.277536 2.222386

H -5.862802 -1.032470 2.823230

H -6.612421 0.557714 2.876230

H -7.298133 -0.729003 1.875155

O -4.974519 3.997379 -1.278188

H -5.582422 4.226638 -0.562060

H -4.978080 4.746864 -1.889575

P1-2LIGAND2222cistrans+1H2Oeq-MnIId

64

P1-2LIGAND2222cistrans+1H2Oeq-MnIId SCF Done: -5154.00462662 A.U.

P -2.499256 1.995023 0.471268

O -1.268032 2.971858 0.327307

O -1.976604 0.861817 1.453530

C -2.850251 -0.163809 2.038256

H -3.618152 0.353645 2.617357

H -3.331448 -0.700494 1.212903

C 0.145461 2.528067 0.455898

H 0.283824 1.653578 -0.185022

H 0.285377 2.220614 1.491178

C 1.005222 3.685221 0.063529

H 0.829696 4.543148 0.708607

H 2.051817 3.399628 0.159842

H 0.832551 3.986037 -0.969206

C -1.998593 -1.059209 2.880902

H -1.514688 -0.499852 3.678610

H -2.624202 -1.823179 3.339718

H -1.235165 -1.557682 2.287491

S -4.214781 2.859662 0.851954

S -2.781663 1.031787 -1.467537

C -2.103767 2.376152 -2.524574

H -2.238245 2.022858 -3.547595

H -1.050335 2.560522 -2.321576

S -3.174662 3.830106 -2.229335

C -3.314821 4.500027 -3.945632

H -4.092131 5.260649 -3.861276

H -3.696858 3.705151 -4.587372

C -2.012485 5.078545 -4.438939

H -2.159187 5.510553 -5.428174

H -1.643447 5.865237 -3.783488

H -1.236595 4.318888 -4.532291

Mn -4.878873 2.246791 -1.460744

P -6.068844 -0.125348 -3.922807

O -7.649831 -0.102572 -3.847800

O -5.724212 -0.801804 -5.317008

C -4.351327 -0.953852 -5.822248

H -3.901125 0.041263 -5.850844

H -3.797329 -1.570110 -5.108545

C -8.461058 -1.300279 -4.172884

H -7.949751 -2.178095 -3.767991

H -8.475645 -1.381479 -5.259086

C -9.819735 -1.093341 -3.584222

H -10.298024 -0.207810 -3.997500

H -10.446775 -1.952508 -3.817085

H -9.777624 -0.991221 -2.500228

C -4.433133 -1.589047 -7.173694

H -5.000007 -0.970253 -7.865954

H -3.428090 -1.711474 -7.574131

H -4.895964 -2.572217 -7.121613

S -5.310063 1.722681 -3.747394

S -5.328294 -1.472611 -2.471634

C -6.407702 -1.024887 -1.074661

H -5.965537 -1.518816 -0.209288

H -7.404742 -1.426802 -1.241597

S -6.577785 0.775414 -0.716453

C -6.854131 0.692770 1.105650

H -6.940698 1.742115 1.390240

H -5.943808 0.305570 1.565641

C -8.084970 -0.094395 1.479524

H -8.244727 -0.027288 2.555127

H -8.976241 0.292671 0.987735

H -7.992743 -1.152729 1.237194

O -6.445958 3.607544 -1.386023

H -7.303663 3.318727 -1.723358

H -6.351731 4.536394 -1.624336

P1-2LIGAND2222cistrans+1H2Oeq-MnIIs

64

P1-2LIGAND2222cistrans+1H2Oeq-MnIIs SCF Done: -5154.10125463 A.U.

P -2.051627 1.808828 -0.256725

O -0.947125 2.552372 0.590215

O -1.745793 0.254251 -0.080835

C -2.754161 -0.760622 -0.377715

H -3.540658 -0.670697 0.375685

H -3.197484 -0.527554 -1.352696

C 0.478442 2.137435 0.556664

H 0.778552 2.064779 -0.491741

H 0.528360 1.146182 1.006668

C 1.259218 3.162806 1.312245

H 0.921533 3.236466 2.343661

H 2.308213 2.870814 1.322079

H 1.187990 4.144423 0.848114

C -2.086618 -2.099249 -0.360482

H -1.629265 -2.296131 0.606827

H -2.828314 -2.876034 -0.543580

H -1.319374 -2.172478 -1.129187

S -3.821722 2.443531 0.388649

S -1.698960 2.126953 -2.307524

C -2.120000 3.922731 -2.334283

H -1.414596 4.391231 -3.016850

H -1.967231 4.353286 -1.346010

S -3.805478 4.347474 -2.853726

C -3.783760 3.792499 -4.612546

H -4.800782 3.989698 -4.951303

H -3.651945 2.707709 -4.604306

C -2.760199 4.503521 -5.459091

H -2.883535 4.201123 -6.498580

H -2.874244 5.584987 -5.413463

H -1.737828 4.252193 -5.178851

Mn -5.403994 2.691893 -1.486633

P -6.594283 -0.048660 -3.422781

O -7.944414 -0.553557 -4.062870

O -5.441501 -0.839838 -4.187873

C -4.049923 -0.397065 -4.168853

H -3.894643 0.174555 -5.085630

H -3.903092 0.286787 -3.322592

C -8.188758 -1.989907 -4.351816

H -7.910046 -2.560447 -3.462012

H -7.520636 -2.262801 -5.168305

C -9.633428 -2.134436 -4.703351

H -9.891771 -1.530757 -5.570422

H -9.835192 -3.176467 -4.947101

H -10.278964 -1.852290 -3.873909

C -3.165740 -1.602512 -4.084578

H -3.306268 -2.255031 -4.943624

H -2.121556 -1.290387 -4.068705

H -3.366304 -2.179707 -3.182256

S -6.565177 1.933975 -3.540491

S -6.420257 -0.779539 -1.455450

C -7.830525 0.170327 -0.730456

H -8.281688 -0.482365 0.014235

H -8.572304 0.359291 -1.504225

S -7.423882 1.752560 0.053641

C -6.764703 1.167113 1.674251

H -6.293743 2.053326 2.100308

H -5.966916 0.453161 1.457847

C -7.832745 0.609576 2.579561

H -7.383555 0.315167 3.527631

H -8.606957 1.344411 2.789936

H -8.310700 -0.278435 2.166601

O -6.479348 4.570492 -1.066032

H -7.395955 4.648555 -0.778099

H -6.154200 5.468035 -1.198942

P1-2LIGAND2222cistrans+1H2Oeq-NiII

64

P1-2LIGAND2222cistrans+1H2Oeq-NiII SCF Done: -5511.34709669 A.U.

P -2.326563 1.391802 0.188467

O -1.307528 2.535660 0.577136

O -1.877285 0.135683 1.049079

C -2.612279 -1.135767 1.083547

H -3.654739 -0.905613 1.319490

H -2.567449 -1.573781 0.082643

C 0.147908 2.272113 0.703077

H 0.453514 1.661739 -0.151233

H 0.282127 1.687395 1.612252

C 0.839027 3.595946 0.751545

H 0.496980 4.189213 1.596718

H 1.910009 3.434954 0.865520

H 0.681031 4.167411 -0.161927

C -1.965936 -2.005797 2.114162

H -2.009848 -1.548852 3.100450

H -2.489189 -2.959710 2.159015

H -0.925725 -2.205313 1.866241

S -4.201590 2.013494 0.537691

S -1.982474 0.818021 -1.818240

C -1.935915 2.470461 -2.583777

H -1.981506 2.287766 -3.656999

H -1.001994 2.971621 -2.335463

S -3.331088 3.577321 -2.117010

C -3.620126 4.436956 -3.720002

H -4.557141 4.969032 -3.549438

H -3.796771 3.678027 -4.482919

C -2.487248 5.370233 -4.070248

H -2.736198 5.902792 -4.987540

H -2.313494 6.111600 -3.292785

H -1.554449 4.837618 -4.252438

Ni -5.061073 2.186759 -1.573121

P -6.025280 -0.139247 -3.797279

O -7.569367 -0.336262 -4.071265

O -5.294042 -0.946435 -4.952861

C -3.844206 -0.903146 -5.184783

H -3.558325 0.148450 -5.273234

H -3.351758 -1.327185 -4.304881

C -8.135743 -1.661002 -4.427125

H -7.704796 -2.402143 -3.748245

H -7.801629 -1.882630 -5.440112

C -9.621791 -1.556261 -4.311246

H -10.016092 -0.791094 -4.976350

H -10.068975 -2.508772 -4.591966

H -9.931955 -1.326342 -3.293006

C -3.561334 -1.682652 -6.429561

H -4.072486 -1.251757 -7.287912

H -2.491146 -1.665745 -6.629635

H -3.867414 -2.721278 -6.324457

S -5.590702 1.818601 -3.760663

S -5.512047 -1.165752 -2.022529

C -6.836995 -0.537518 -0.940335

H -6.550625 -0.842409 0.065654

H -7.785373 -1.000883 -1.206313

S -7.064134 1.288520 -0.961514

C -7.437902 1.590274 0.816979

H -7.398233 2.677298 0.894305

H -6.620016 1.177732 1.409234

C -8.785487 1.034169 1.206638

H -8.990247 1.293014 2.244859

H -9.588024 1.445001 0.596863

H -8.820500 -0.052299 1.134755

O -6.278724 4.392451 -1.102225

H -7.019706 4.800965 -1.563007

H -6.009492 5.043103 -0.444363

P1-2LIGAND2222cistrans+1H2Oeq-NiIIt

64

P1-2LIGAND2222cistrans+1H2Oeq-NiIIt SCF Done: -5511.38211949 A.U.

P -1.985265 2.197707 0.372788

O -1.250977 3.162740 1.384518

O -1.314847 0.775299 0.618079

C -1.807082 -0.456211 -0.002606

H -2.869315 -0.549293 0.242731

H -1.714940 -0.349961 -1.087185

C 0.223878 3.141392 1.544547

H 0.665942 3.159031 0.544935

H 0.475456 2.195586 2.023738

C 0.601964 4.332118 2.364291

H 0.124755 4.307018 3.341581

H 1.680361 4.328724 2.516202

H 0.335085 5.262873 1.867385

C -0.989483 -1.590724 0.529984

H -1.083994 -1.672066 1.610636

H -1.334120 -2.524530 0.088455

H 0.062688 -1.470009 0.280570

S -3.938969 2.357129 0.736456

S -1.427912 2.657755 -1.600868

C -2.346429 4.237859 -1.734108

H -1.767588 4.877264 -2.397800

H -2.415269 4.728721 -0.764990

S -4.034096 4.110159 -2.399411

C -3.658990 3.515701 -4.100679

H -4.602433 3.101510 -4.458860

H -2.968469 2.676690 -3.988292

C -3.126085 4.601751 -5.000606

H -2.944663 4.190926 -5.993242

H -3.825180 5.429369 -5.102549

H -2.177126 5.002997 -4.647133

Ni -5.227654 2.296975 -1.259053

P -6.047301 -0.405372 -3.288792

O -6.834883 0.656947 -4.212659

O -5.753257 -1.607273 -4.276055

C -5.022959 -2.831874 -3.881839

H -4.038613 -2.522305 -3.525224

H -5.570615 -3.292778 -3.056587

C -7.933788 0.198600 -5.107189

H -8.507320 -0.560448 -4.570676

H -7.453602 -0.264866 -5.967491

C -8.754801 1.396295 -5.464165

H -8.155079 2.158046 -5.961333

H -9.541728 1.100307 -6.156084

H -9.239460 1.831705 -4.589594

C -4.951510 -3.716836 -5.083831

H -4.416324 -3.235170 -5.899185

H -4.417208 -4.628844 -4.822066

H -5.944090 -3.997834 -5.429562

S -4.422713 0.443781 -2.524325

S -7.432790 -1.206175 -1.925493

C -8.076111 0.325583 -1.153804

H -8.968059 -0.004136 -0.623222

H -8.379898 1.045595 -1.911165

S -7.020104 1.235297 0.022452

C -6.353674 -0.128387 1.057027

H -5.691035 0.384246 1.753912

H -5.737904 -0.750012 0.403674

C -7.434635 -0.908672 1.760416

H -6.975639 -1.633905 2.431075

H -8.077856 -0.264885 2.357563

H -8.057761 -1.471868 1.065589

O -6.834916 2.867749 -2.586779

H -6.910767 2.353830 -3.410923

H -7.094402 3.776706 -2.774865

P1-2LIGAND2222cistrans+1H2Oeq-ZnII

64

P1-2LIGAND2222cistrans+1H2Oeq-ZnII SCF Done: -5782.42059460 A.U.

P -2.171418 2.315465 0.144597

O -1.246798 3.002166 1.221111

O -1.903468 0.751607 0.325723

C -2.698118 -0.261708 -0.357566

H -3.742580 0.073885 -0.374795

H -2.352498 -0.317079 -1.393414

C 0.191364 2.660921 1.348505

H 0.631352 2.715768 0.349533

H 0.242287 1.631054 1.701654

C 0.799195 3.636392 2.303075

H 0.323537 3.582315 3.279934

H 1.854012 3.396277 2.429046

H 0.727472 4.657146 1.933519

C -2.539078 -1.553430 0.382501

H -2.884563 -1.463837 1.411078

H -3.128446 -2.328329 -0.107155

H -1.500779 -1.877805 0.396048

S -4.022213 2.967685 0.529434

S -1.511059 2.706078 -1.805320

C -2.227472 4.395667 -2.014211

H -1.516744 4.946429 -2.626938

H -2.304150 4.893570 -1.050100

S -3.858605 4.455292 -2.798740

C -3.434683 3.954670 -4.520295

H -4.406743 3.791827 -4.985798

H -2.942681 2.981119 -4.463495

C -2.615702 4.984192 -5.256123

H -2.446692 4.646449 -6.278158

H -3.119443 5.947703 -5.301003

H -1.633936 5.133476 -4.807911

Zn -5.203423 2.496465 -1.466730

P -5.948336 -0.466636 -3.434094

O -7.201656 0.021340 -4.270406

O -5.331369 -1.676067 -4.254466

C -4.033686 -2.289951 -3.925910

H -3.272438 -1.514263 -4.034751

H -4.066457 -2.605469 -2.879159

C -8.169080 -0.955835 -4.837198

H -8.421220 -1.670876 -4.049716

H -7.647051 -1.484615 -5.633642

C -9.352220 -0.184527 -5.324212

H -9.066731 0.540929 -6.083002

H -10.065411 -0.874662 -5.772377

H -9.857798 0.336489 -4.512733

C -3.824582 -3.438988 -4.859272

H -3.814722 -3.107131 -5.895140

H -2.863031 -3.903290 -4.645594

H -4.597678 -4.194741 -4.739771

S -4.637717 1.029587 -3.228586

S -6.648752 -1.359545 -1.661914

C -7.815446 -0.038427 -1.143947

H -8.579916 -0.544846 -0.558003

H -8.295553 0.399002 -2.018538

S -7.142896 1.329216 -0.158236

C -6.434220 0.424840 1.281387

H -6.001902 1.218846 1.888551

H -5.612712 -0.189018 0.904384

C -7.456947 -0.380873 2.040262

H -6.992889 -0.795139 2.934761

H -8.302144 0.227234 2.357696

H -7.834832 -1.225120 1.464269

O -6.720101 3.959522 -1.717988

H -7.527012 4.013377 -1.192861

H -6.512980 4.855755 -2.009483

P1-2LIGAND222+2OH2-CoIId

67

P1-2LIGAND222+2OH2-CoIId SCF Done: -5462.26733327 A.U.

P -2.013004 3.190399 -0.935915

O -2.104416 4.721129 -0.527996

O -0.461223 2.954670 -1.143063

C 0.088454 1.660126 -1.586645

H 0.219377 1.053137 -0.690665

H -0.650703 1.176047 -2.231906

C -0.932195 5.625139 -0.329210

H -0.248467 5.463145 -1.160532

H -0.455340 5.314616 0.599710

C -1.472477 7.016467 -0.281197

H -2.167364 7.152383 0.546465

H -0.648128 7.713292 -0.135612

H -1.969232 7.282467 -1.214068

C 1.370628 1.937328 -2.304081

H 2.084875 2.440177 -1.655432

H 1.814904 0.997431 -2.627723

H 1.202250 2.555767 -3.184820

S -3.017299 1.950333 0.240559

S -3.136369 2.940969 -2.718576

C -3.193701 4.722652 -3.395068

H -3.672306 5.312253 -2.616242

H -3.853671 4.630790 -4.257485

S -1.609029 5.408872 -3.800930

C -1.256654 4.568066 -5.398827

H -2.099189 4.763970 -6.062841

H -1.206632 3.493553 -5.210506

C 0.037536 5.093542 -5.972297

H 0.246171 4.598646 -6.919288

H -0.013045 6.163955 -6.164196

H 0.881879 4.907279 -5.309840

Co -4.812783 2.466131 -1.178489

P -7.738945 2.153882 -1.219858

O -8.911836 3.126983 -0.800162

O -8.439310 0.740482 -1.441620

C -8.098525 -0.257818 -2.468152

H -7.617292 0.267154 -3.296255

H -7.374258 -0.939489 -2.017565

C -9.902368 2.714287 0.219842

H -9.351247 2.289009 1.067852

H -10.513100 1.930627 -0.231092

C -10.694329 3.923183 0.599959

H -11.206701 4.344872 -0.262176

H -11.447393 3.638720 1.333406

H -10.063814 4.690117 1.044887

C -9.362830 -0.946388 -2.874897

H -10.071978 -0.249441 -3.316346

H -9.133038 -1.708828 -3.617904

H -9.836568 -1.439391 -2.027827

S -6.592735 2.889352 -2.649394

S -6.411777 2.013817 0.429684

C -6.463932 0.181676 0.982278

H -5.453061 0.047687 1.363745

H -6.598697 -0.418749 0.085248

S -7.651952 -0.151304 2.258999

C -9.019342 -0.946215 1.317080

H -8.607982 -1.823920 0.815338

H -9.373014 -0.250328 0.556750

C -10.118670 -1.320016 2.283328

H -10.928264 -1.811540 1.746360

H -9.767853 -2.007978 3.050665

H -10.533762 -0.443333 2.779765

O -4.895890 4.741680 -0.604089

H -4.249350 5.123258 0.003319

H -5.721904 5.214119 -0.453754

O -4.848472 0.168417 -1.854605

H -4.113894 -0.369861 -1.535974

H -4.894010 -0.012762 -2.800614

P1-2LIGAND222+2OH2-CoIIq

67

P1-2LIGAND222+2OH2-CoIIq SCF Done: -5462.27769538 A.U.

P -1.989223 3.161568 -1.212513

O -2.429361 4.657793 -0.841944

O -0.412866 3.277849 -1.309503

C 0.439136 2.135775 -1.687366

H 0.388495 1.407877 -0.876103

H 0.014472 1.690009 -2.591735

C -1.472895 5.711022 -0.389077

H -0.683634 5.760546 -1.136916

H -1.056276 5.377011 0.560768

C -2.251578 6.980414 -0.276873

H -3.046950 6.904964 0.463796

H -1.583479 7.778889 0.043081

H -2.678470 7.273257 -1.235686

C 1.819904 2.664637 -1.909880

H 2.219669 3.119595 -1.006081

H 2.478071 1.844687 -2.192611

H 1.833987 3.403010 -2.710032

S -2.728366 1.780872 0.008369

S -2.914359 2.748417 -3.053496

C -3.065424 4.473738 -3.840996

H -3.783388 5.021445 -3.235181

H -3.504461 4.242037 -4.811196

S -1.567126 5.419488 -3.972783

C -0.703733 4.495759 -5.306595

H -1.383884 4.430392 -6.156700

H -0.513247 3.483471 -4.940422

C 0.576345 5.207289 -5.674991

H 1.095781 4.650627 -6.453048

H 0.383215 6.207564 -6.058565

H 1.252460 5.296648 -4.825684

Co -4.759222 1.981709 -1.349284

P -7.713067 1.527674 -1.209327

O -8.586983 2.812873 -0.849154

O -8.643209 0.232033 -1.261018

C -9.364490 -0.157227 -2.493178

H -8.617355 -0.296951 -3.277247

H -9.781085 -1.127420 -2.232285

C -9.597739 2.763113 0.230101

H -9.056956 2.828477 1.176820

H -10.096010 1.792593 0.181800

C -10.544671 3.900691 0.016502

H -11.060349 3.810419 -0.937663

H -11.292987 3.897456 0.807621

H -10.030526 4.859249 0.044864

C -10.411361 0.845087 -2.873258

H -9.975367 1.809120 -3.140124

H -10.955580 0.482440 -3.744168

H -11.134087 0.994101 -2.072058

S -6.654915 1.931425 -2.844521

S -6.467979 1.192721 0.442602

C -6.367968 -0.741305 0.644368

H -5.306565 -0.948803 0.543034

H -6.918111 -1.130795 -0.209356

S -6.955405 -1.315166 2.199147

C -8.762153 -1.219747 1.899382

H -8.993724 -1.856571 1.043892

H -8.988452 -0.185741 1.623357

C -9.517391 -1.644841 3.135990

H -10.589195 -1.585087 2.954118

H -9.289154 -2.672992 3.411584

H -9.288666 -1.007712 3.988762

O -5.105576 4.034803 -0.849754

H -4.347085 4.570669 -0.562398

H -5.895606 4.377602 -0.418611

O -4.412847 -0.109214 -1.917407

H -3.519445 -0.471937 -1.876581

H -4.786780 -0.402701 -2.757084

P1-2LIGAND222+2OH2-CuIId

67

P1-2LIGAND222+2OH2-CuIId SCF Done: -5719.97875779 A.U.

P -1.951163 3.269479 -1.076242

O -2.136706 4.805986 -0.719840

O -0.382824 3.117027 -1.249763

C 0.246750 1.835007 -1.610188

H 0.115389 1.159641 -0.763354

H -0.292178 1.429812 -2.471603

C -1.022296 5.758233 -0.447246

H -0.278004 5.625775 -1.230390

H -0.593428 5.473896 0.513174

C -1.625041 7.124916 -0.443928

H -2.380242 7.232135 0.333558

H -0.845135 7.859181 -0.247302

H -2.068811 7.363930 -1.410162

C 1.683877 2.111249 -1.917328

H 2.198189 2.534811 -1.057166

H 2.180619 1.178498 -2.179713

H 1.784404 2.795833 -2.757988

S -2.844353 1.992267 0.146997

S -2.984091 2.925716 -2.894037

C -3.115976 4.667456 -3.622133

H -3.672259 5.252369 -2.892519

H -3.719992 4.511515 -4.515950

S -1.556958 5.450920 -3.964259

C -1.018303 4.530992 -5.461893

H -1.804390 4.633829 -6.210748

H -0.933577 3.475974 -5.192232

C 0.297322 5.086388 -5.953268

H 0.617254 4.539135 -6.838146

H 0.214507 6.136573 -6.227904

H 1.082598 4.992005 -5.204365

Cu -4.766904 2.330220 -1.244583

P -7.708246 1.870678 -1.166032

O -8.955159 2.806210 -0.858811

O -8.185139 0.348370 -1.236171

C -8.657359 -0.276241 -2.486539

H -7.913141 -0.070022 -3.259405

H -8.633490 -1.338754 -2.252696

C -9.902889 2.445476 0.213350

H -9.322871 2.075794 1.067852

H -10.518942 1.628708 -0.168000

C -10.701359 3.663891 0.549489

H -11.242588 4.031653 -0.319753

H -11.429574 3.413699 1.319426

H -10.067455 4.461607 0.931268

C -10.024253 0.202760 -2.869089

H -10.033775 1.267038 -3.107047

H -10.357594 -0.332116 -3.757365

H -10.748316 0.009418 -2.078617

S -6.672362 2.587157 -2.685358

S -6.453760 1.875165 0.536263

C -6.400856 0.082314 1.164931

H -5.405426 0.038413 1.603603

H -6.453500 -0.565636 0.293443

S -7.615186 -0.277804 2.415487

C -8.914900 -1.171157 1.471428

H -8.423411 -1.964842 0.906684

H -9.370393 -0.481090 0.760366

C -9.929095 -1.721627 2.447276

H -10.702501 -2.262369 1.904222

H -9.475855 -2.414683 3.153598

H -10.416766 -0.930487 3.015260

O -4.938629 4.677481 -0.834610

H -4.317627 5.143734 -0.260905

H -5.766716 5.167745 -0.792075

O -4.792161 -0.008731 -1.765334

H -4.025723 -0.546393 -1.535176

H -5.006509 -0.259433 -2.670936

P1-2LIGAND222+2OH2-FeIIId

67

P1-2LIGAND222+2OH2-FeIIId SCF Done: -5342.49868944 A.U.

P -1.903873 2.894134 -1.252015

O -1.377329 4.186899 -0.500416

O -0.748017 1.899272 -1.794177

C 0.578478 1.742780 -2.632140

H 0.868087 0.750335 -2.228405

H 0.288582 1.650993 -3.688109

C -0.015117 4.657815 -0.318552

H 0.447149 4.850753 -1.294519

H 0.576337 3.896835 0.215143

C -0.111401 5.924088 0.482775

H -0.577524 5.733894 1.455571

H 0.901381 6.308906 0.639691

H -0.703923 6.676103 -0.051095

C 1.684667 2.663254 -2.280356

H 1.841957 2.685626 -1.199243

H 2.582422 2.266832 -2.766058

H 1.509299 3.681846 -2.626943

S -3.084607 1.827378 -0.047837

S -3.319572 3.525522 -2.728234

C -2.816467 4.602157 -4.159638

H -3.509212 5.472314 -4.086494

H -3.114075 4.011888 -5.058869

S -1.024382 5.098666 -4.202087

C -1.047897 6.140497 -5.705436

H -1.758396 6.969755 -5.558704

H -1.387201 5.536389 -6.562456

C 0.357507 6.666165 -5.934188

H 0.335121 7.290248 -6.832682

H 0.699634 7.272289 -5.086247

H 1.069274 5.848001 -6.088470

Fe -4.828146 2.307399 -1.456206

P -7.872600 1.712407 -1.733998

O -9.069543 2.623025 -1.258290

O -8.313547 0.311327 -2.382300

C -9.176827 -0.963390 -2.038488

H -9.294440 -1.332836 -3.078058

H -8.534791 -1.649943 -1.468244

C -10.499017 2.351836 -1.272139

H -10.720114 1.475576 -0.651617

H -10.832605 2.154211 -2.303454

C -11.163967 3.578541 -0.717464

H -10.939252 4.454261 -1.335393

H -12.245327 3.408501 -0.714292

H -10.826719 3.773863 0.306751

C -10.531458 -0.688204 -1.512456

H -11.050757 0.043321 -2.135944

H -11.074194 -1.638116 -1.541700

H -10.506075 -0.319037 -0.478358

S -6.601000 2.737834 -2.874502

S -6.546548 1.427612 -0.107010

C -7.007056 0.520803 1.447207

H -6.739370 1.245526 2.260110

H -6.244710 -0.303275 1.496652

S -8.766305 -0.102534 1.486654

C -8.897281 -0.937345 3.106975

H -8.667146 -0.195457 3.890984

H -8.135188 -1.736162 3.138611

C -10.295279 -1.512988 3.300629

H -10.335106 -2.003577 4.277856

H -11.053857 -0.723567 3.270536

H -10.527765 -2.250680 2.524888

O -5.166133 4.020320 -0.471588

H -5.066599 4.112156 0.481130

H -5.443123 4.834180 -0.902946

O -4.500535 0.606441 -2.433426

H -3.619838 0.280332 -2.652034

H -5.203933 0.022693 -2.739379

P1-2LIGAND222+2OH2-FeIIIs

67

P1-2LIGAND222+2OH2-FeIIIs SCF Done: -5342.78378401 A.U.

P -2.152504 3.235516 -1.207496

O -2.787347 4.682077 -1.014195

O -0.601589 3.471975 -1.174769

C 0.426297 2.386174 -1.214677

H 0.267455 1.772383 -0.327519

H 0.222992 1.793803 -2.109584

C -2.028103 5.849239 -0.417102

H -1.105384 5.936730 -0.987584

H -1.799383 5.574752 0.611867

C -2.913958 7.041910 -0.533261

H -3.834946 6.925107 0.036678

H -2.389195 7.904853 -0.123605

H -3.152567 7.268450 -1.571467

C 1.764544 3.045040 -1.243192

H 1.931859 3.649106 -0.354250

H 2.532603 2.272959 -1.269958

H 1.885414 3.668395 -2.127136

S -2.877230 1.830728 0.014487

S -2.884626 2.609556 -3.100849

C -3.132154 4.276510 -4.016479

H -3.907440 4.806431 -3.469289

H -3.499394 3.958023 -4.991436

S -1.681811 5.291012 -4.122246

C -0.792825 4.526891 -5.538772

H -1.484609 4.531552 -6.383238

H -0.589914 3.487309 -5.267547

C 0.471134 5.295850 -5.835029

H 0.978494 4.830932 -6.678422

H 0.263994 6.328611 -6.108157

H 1.162035 5.292599 -4.993643

Fe -4.742026 1.627129 -1.568920

P -7.695348 1.263804 -1.464774

O -8.180630 2.757318 -1.195937

O -8.869392 0.214594 -1.399643

C -9.731778 -0.132100 -2.587249

H -9.056471 -0.499754 -3.360949

H -10.318465 -0.962250 -2.203206

C -9.191588 3.079550 -0.134267

H -9.339774 2.187256 0.476930

H -10.108182 3.290724 -0.681571

C -8.687796 4.244673 0.651600

H -8.529020 5.114856 0.015448

H -9.432884 4.524301 1.395508

H -7.767011 4.003499 1.183860

C -10.558644 1.032189 -3.022197

H -9.949503 1.857833 -3.395090

H -11.202692 0.715594 -3.842346

H -11.204793 1.388897 -2.221974

S -6.572316 1.260336 -3.117616

S -6.511549 0.807582 0.229763

C -6.605897 -1.110550 0.384076

H -5.621156 -1.470163 0.098816

H -7.361978 -1.421155 -0.335101

S -6.973825 -1.625189 2.040007

C -8.793721 -1.419253 2.075871

H -9.202003 -2.072881 1.300924

H -8.999479 -0.383852 1.786316

C -9.334547 -1.748902 3.445758

H -10.416765 -1.633244 3.439144

H -9.119590 -2.777309 3.729408

H -8.934687 -1.089206 4.213427

O -5.416374 3.621959 -1.164652

H -4.851931 4.339347 -0.835696

H -6.338661 3.916466 -1.209526

O -4.104198 -0.410997 -1.945498

H -3.217510 -0.723995 -1.723902

H -4.404904 -0.934887 -2.699366

P1-2LIGAND222+2OH2-MnIId

67

P1-2LIGAND222+2OH2-MnIId SCF Done: -5230.45490546 A.U.

P -1.886367 3.173563 -1.118772

O -1.749626 4.612523 -0.468676

O -0.389114 2.793517 -1.493873

C -0.039069 1.532925 -2.169026

H -0.230311 0.723038 -1.462506

H -0.707529 1.421697 -3.027924

C -0.456040 5.305325 -0.209964

H 0.172022 5.159476 -1.086007

H -0.003370 4.809707 0.648625

C -0.776754 6.741948 0.042785

H -1.436460 6.864703 0.900179

H 0.147646 7.276735 0.257278

H -1.234629 7.209814 -0.828405

C 1.396407 1.619208 -2.579678

H 2.043259 1.770230 -1.717853

H 1.690617 0.687667 -3.060169

H 1.558804 2.430526 -3.287691

S -2.962202 1.895113 -0.026016

S -3.165442 3.373430 -2.793570

C -2.958558 5.230002 -3.181868

H -3.259144 5.751892 -2.276191

H -3.692300 5.384244 -3.972936

S -1.313256 5.722291 -3.625774

C -1.166593 4.959637 -5.291042

H -1.984678 5.341362 -5.903660

H -1.298619 3.881216 -5.173953

C 0.179194 5.287285 -5.894251

H 0.258657 4.832795 -6.880405

H 0.315711 6.360576 -6.015363

H 1.000741 4.906161 -5.288304

Mn -4.836368 2.772614 -1.215872

P -7.884355 2.437011 -1.321896

O -9.207274 3.191100 -0.906893

O -8.207527 1.128809 -2.146220

C -8.429782 -0.308735 -1.916256

H -7.971791 -0.771498 -2.789308

H -7.873056 -0.604325 -1.029595

C -10.541390 2.591511 -0.695022

H -10.439140 1.836466 0.085888

H -10.826233 2.104191 -1.628192

C -11.471948 3.696587 -0.311949

H -11.531526 4.452151 -1.092546

H -12.468967 3.284262 -0.165329

H -11.163084 4.171585 0.616787

C -9.880830 -0.639501 -1.799130

H -10.448399 -0.272581 -2.653046

H -9.987961 -1.723712 -1.774529

H -10.308324 -0.242980 -0.879416

S -6.687804 3.630749 -2.409866

S -6.510356 2.230750 0.322333

C -6.670471 0.533359 1.012894

H -5.970876 0.538543 1.852566

H -6.339194 -0.200677 0.277138

S -8.383331 0.275478 1.548701

C -8.360853 -1.552760 1.756840

H -7.562420 -1.795117 2.458568

H -8.119998 -2.009228 0.793502

C -9.704797 -2.012169 2.272770

H -9.695255 -3.092825 2.403386

H -9.937822 -1.565423 3.237996

H -10.511265 -1.770289 1.580938

O -4.656842 4.658753 -0.300174

H -4.164861 4.773031 0.522245

H -5.459406 5.190508 -0.230881

O -5.087358 0.923041 -2.206824

H -4.277631 0.427106 -2.384810

H -5.529751 1.032299 -3.060284

P1-2LIGAND222+2OH2-MnIIs

67

P1-2LIGAND222+2OH2-MnIIs SCF Done: -5230.55596971 A.U.

P -2.054172 3.118586 -1.330314

O -2.730092 4.536421 -1.009457

O -0.505669 3.457934 -1.367582

C 0.515102 2.408921 -1.546824

H 0.477478 1.763715 -0.667910

H 0.237271 1.820922 -2.425877

C -1.974394 5.692603 -0.452561

H -1.122401 5.859062 -1.109505

H -1.622314 5.403796 0.537976

C -2.918480 6.850418 -0.424737

H -3.772905 6.662301 0.225093

H -2.399723 7.725130 -0.034937

H -3.274401 7.097654 -1.424738

C 1.834488 3.092783 -1.711968

H 2.081296 3.692833 -0.838762

H 2.614238 2.343029 -1.834745

H 1.841720 3.734387 -2.591258

S -2.586538 1.626034 -0.145628

S -2.809382 2.596099 -3.224751

C -3.053594 4.287686 -4.065346

H -3.902108 4.756124 -3.572339

H -3.327542 3.999472 -5.079756

S -1.675292 5.407837 -4.015113

C -0.495450 4.556842 -5.134020

H -1.012833 4.348236 -6.071317

H -0.227678 3.603823 -4.670534

C 0.717675 5.429967 -5.351912

H 1.433181 4.914262 -5.990333

H 0.455522 6.367164 -5.839737

H 1.221205 5.668187 -4.415344

Mn -4.709717 1.490932 -1.553253

P -7.749101 1.310447 -1.340254

O -8.131563 2.834618 -0.981549

O -9.047224 0.396471 -1.219678

C -9.947882 0.163443 -2.372103

H -9.333681 -0.194673 -3.200629

H -10.577729 -0.652262 -2.025219

C -9.006739 3.138088 0.176698

H -8.379052 3.097760 1.069216

H -9.757964 2.349890 0.245459

C -9.610705 4.486673 -0.054134

H -10.217713 4.499579 -0.957159

H -10.251585 4.742433 0.788183

H -8.848044 5.259565 -0.136088

C -10.724877 1.395334 -2.721414

H -10.075386 2.200064 -3.069562

H -11.418635 1.165594 -3.528914

H -11.310958 1.750996 -1.874967

S -6.792794 1.270659 -3.064961

S -6.563286 0.687594 0.281246

C -6.715576 -1.242434 0.213568

H -5.796103 -1.587833 -0.249781

H -7.570464 -1.424339 -0.434991

S -6.885836 -1.958316 1.816081

C -8.647189 -1.555112 2.144822

H -9.244282 -2.004443 1.349412

H -8.750282 -0.468852 2.079478

C -9.049163 -2.068434 3.506805

H -10.093014 -1.826010 3.698428

H -8.944852 -3.149781 3.576229

H -8.453527 -1.618338 4.299457

O -5.345871 3.537637 -1.078899

H -4.749115 4.253896 -0.814488

H -6.261049 3.852138 -1.101874

O -4.174266 -0.572710 -2.145201

H -3.300699 -0.960694 -2.016674

H -4.583528 -1.045114 -2.879550

P1-2LIGAND222+2OH2-NiII

67

P1-2LIGAND222+2OH2-NiII SCF Done: -5586.38695906 A.U.

P -2.183426 2.834612 -1.289720

O -1.552475 4.178632 -0.482349

O -0.932630 1.925600 -1.983975

C 0.222195 2.044241 -2.859138

H 0.448170 1.104339 -2.385024

H -0.238711 1.869526 -3.814260

C -0.199490 4.625284 -0.338329

H 0.219374 4.821820 -1.308214

H 0.380400 3.865018 0.154052

C -0.186430 5.909612 0.499373

H -0.606458 5.713849 1.468651

H 0.826140 6.250235 0.611241

H -0.766227 6.668091 0.006535

C 1.345957 3.075803 -2.740821

H 1.626806 3.188197 -1.710367

H 2.193090 2.735696 -3.306995

H 1.015905 4.022175 -3.124964

S -3.316288 1.749951 -0.045745

S -3.507635 3.471804 -2.748914

C -2.887060 4.503898 -4.081288

H -3.443068 5.426750 -4.082469

H -3.048921 3.989646 -5.014199

S -1.356447 4.811789 -4.000929

C -0.819207 5.847331 -5.356025

H -1.354779 6.779316 -5.328975

H -1.016988 5.350160 -6.288772

C 0.687672 6.114346 -5.223491

H 1.011542 6.736754 -6.036602

H 0.887131 6.612180 -4.292225

H 1.224670 5.184213 -5.251599

Ni -4.901937 2.280435 -1.470454

P -7.818053 1.739259 -1.792765

O -9.137613 2.695974 -1.348112

O -8.351654 0.255611 -2.409885

C -9.206548 -0.868087 -2.063364

H -9.131485 -1.018450 -3.126690

H -8.501444 -1.500818 -1.555372

C -10.543185 2.427847 -1.412034

H -10.772779 1.566657 -0.811976

H -10.827969 2.238875 -2.431800

C -11.306251 3.646588 -0.879322

H -11.075764 4.507930 -1.478549

H -12.361657 3.451976 -0.924636

H -11.020940 3.834655 0.139242

C -10.645548 -0.783486 -1.551420

H -11.185924 -0.042906 -2.110655

H -11.117372 -1.740278 -1.675876

H -10.647638 -0.516463 -0.510340

S -6.505978 2.755852 -2.909766

S -6.616944 1.415342 -0.143374

C -7.075465 0.546049 1.356571

H -6.907761 1.204862 2.194099

H -6.437900 -0.319022 1.452685

S -8.448305 -0.017208 1.485656

C -8.349797 -0.775329 3.101103

H -8.118214 -0.021587 3.832740

H -7.572526 -1.519250 3.097386

C -9.694342 -1.435036 3.446657

H -9.630706 -1.889114 4.417949

H -10.471014 -0.692827 3.450998

H -9.926203 -2.188037 2.716285

O -5.217270 3.871212 -0.574138

H -5.127315 3.918842 0.380969

H -5.472995 4.660355 -1.058429

O -4.588893 0.702208 -2.385378

H -3.690673 0.413384 -2.565798

H -5.321996 0.159808 -2.687165

P1-2LIGAND222+2OH2-NiIIt

67

P1-2LIGAND222+2OH2-NiIIt SCF Done: -5587.81425551 A.U.

P -1.757402 2.965141 -1.333995

O -1.687699 4.316819 -0.490955

O -0.254652 2.740947 -1.779099

C 0.154073 1.646332 -2.676481

H 0.119985 0.723900 -2.095338

H -0.579909 1.582300 -3.485117

C -0.437941 5.081467 -0.200404

H 0.152828 5.097928 -1.114189

H 0.092672 4.520955 0.568245

C -0.858477 6.444211 0.242703

H -1.477440 6.407383 1.138133

H 0.028599 7.028871 0.482397

H -1.396383 6.971695 -0.544624

C 1.523435 1.971852 -3.180726

H 2.237196 2.053139 -2.363765

H 1.861583 1.177811 -3.844548

H 1.524095 2.906219 -3.740481

S -2.697953 1.492889 -0.410236

S -3.032142 3.334027 -2.992245

C -2.900577 5.208394 -3.228934

H -3.283907 5.657785 -2.315096

H -3.594456 5.393435 -4.049054

S -1.264157 5.820245 -3.558559

C -1.029927 5.270350 -5.297551

H -1.871231 5.651284 -5.877750

H -1.065007 4.178548 -5.313061

C 0.287334 5.789959 -5.822844

H 0.424555 5.462858 -6.852180

H 0.323140 6.877852 -5.815548

H 1.132230 5.418937 -5.243884

Ni -4.715246 2.572535 -1.303486

P -7.814011 2.270183 -1.165111

O -9.157123 2.924871 -0.659413

O -8.108295 0.951635 -2.003338

C -8.205467 -0.463851 -1.618870

H -7.617787 -0.978530 -2.377617

H -7.712959 -0.594653 -0.656794

C -10.521640 2.401156 -0.877218

H -10.661111 1.593649 -0.156953

H -10.567079 1.989478 -1.886487

C -11.473738 3.533683 -0.666746

H -11.307466 4.334163 -1.384233

H -12.491051 3.167526 -0.799156

H -11.390360 3.939229 0.339265

C -9.627663 -0.927437 -1.600793

H -10.127589 -0.714309 -2.544099

H -9.647612 -2.007494 -1.453871

H -10.186707 -0.470228 -0.786410

S -6.727737 3.545033 -2.234594

S -6.481014 2.033628 0.477814

C -6.939442 0.581351 1.519450

H -6.406687 0.766797 2.453274

H -6.561215 -0.334799 1.065520

S -8.736996 0.520378 1.751336

C -8.903555 -1.258265 2.191112

H -8.300061 -1.439345 3.080493

H -8.495843 -1.855662 1.371880

C -10.359962 -1.573529 2.439711

H -10.465463 -2.623966 2.706212

H -10.764708 -0.983661 3.260316

H -10.972300 -1.394457 1.555994

O -4.424735 4.417236 -0.270938

H -3.605852 4.581582 0.220738

H -5.155931 4.801003 0.227109

O -5.110777 0.779276 -2.384150

H -4.363497 0.206887 -2.596920

H -5.619035 0.892070 -3.198338

P1-2LIGAND222+2OH2-ZnII

67

P1-2LIGAND222+2OH2-ZnII SCF Done: -5858.87757590 A.U.

P -2.788961 3.453501 -2.338862

O -2.467767 4.717521 -1.399541

O -1.487287 2.560867 -2.202983

C -1.280681 1.351206 -3.021461

H -1.904446 0.564690 -2.594288

H -1.630348 1.567449 -4.034823

C -1.391551 4.698953 -0.363744

H -0.478203 4.390280 -0.869493

H -1.670374 3.949821 0.377683

C -1.306168 6.081943 0.193766

H -2.229580 6.382342 0.688797

H -0.512790 6.116488 0.939049

H -1.060894 6.807196 -0.581375

C 0.178245 1.027054 -2.979585

H 0.514217 0.834417 -1.962809

H 0.365417 0.132403 -3.571331

H 0.771491 1.839704 -3.395590

S -4.429050 2.416552 -1.812986

S -3.022497 4.225756 -4.252178

C -1.793917 5.684579 -4.205813

H -2.236081 6.427016 -3.547025

H -1.839474 6.039042 -5.235225

S -0.129782 5.366186 -3.675331

C 0.553157 4.535387 -5.165028

H 0.329337 5.168383 -6.024301

H 0.027265 3.586205 -5.293544

C 2.041191 4.333533 -5.002308

H 2.439815 3.822696 -5.877337

H 2.565966 5.282276 -4.905788

H 2.280347 3.726795 -4.129515

Zn -6.050300 4.076666 -1.935758

P -8.315877 2.488846 -0.145958

O -9.834867 2.043359 -0.043254

O -7.480616 1.511204 -1.117804

C -7.294062 0.082038 -0.803939

H -6.325763 -0.149540 -1.244699

H -7.218812 -0.020667 0.282495

C -10.744000 2.004338 -1.206167

H -10.202599 1.561711 -2.045603

H -10.993265 3.037481 -1.451858

C -11.935525 1.196744 -0.800259

H -12.444531 1.645665 0.050646

H -12.641230 1.152367 -1.628303

H -11.653721 0.177909 -0.538593

C -8.404857 -0.747033 -1.371381

H -8.503154 -0.606558 -2.446820

H -8.195541 -1.801025 -1.190864

H -9.353667 -0.519870 -0.885763

S -8.119303 4.310402 -1.001854

S -7.655616 2.388003 1.803571

C -9.060409 1.441315 2.669080

H -9.971621 1.999540 2.480009

H -8.769400 1.564831 3.711937

S -9.315715 -0.263374 2.237373

C -7.870672 -1.027778 3.073444

H -8.007361 -0.925784 4.150185

H -6.984666 -0.454256 2.787184

C -7.747249 -2.475549 2.662536

H -6.897632 -2.935625 3.164402

H -8.634007 -3.048006 2.930149

H -7.594789 -2.576925 1.587474

O -4.990803 5.830318 -1.514090

H -4.067125 5.758632 -1.218365

H -5.382859 6.611673 -1.110496

O -6.287542 4.707012 -3.895211

H -5.486757 4.761331 -4.438368

H -7.025932 4.487055 -4.472602

P1-2LIGAND2222pq-CoIIq

61

P1-2LIGAND2222pq-CoIIq SCF Done: -5309.36414472 A.U.

P -2.455642 1.500992 -0.276568

O -1.466732 1.362876 0.940529

O -2.198635 0.218455 -1.177880

C -3.048362 -0.143108 -2.316679

H -4.090454 -0.001464 -2.011909

H -2.828910 0.554739 -3.129244

C -0.046190 0.953042 0.758511

H 0.371745 1.562703 -0.046479

H -0.062222 -0.088710 0.440408

C 0.647134 1.158825 2.064837

H 0.190347 0.564851 2.853376

H 1.684811 0.843108 1.965166

H 0.641692 2.205008 2.363371

C -2.741157 -1.561687 -2.681848

H -2.969992 -2.240914 -1.863363

H -3.339643 -1.852035 -3.544625

H -1.693998 -1.680540 -2.951984

S -4.303752 1.695279 0.485989

S -1.879254 3.084174 -1.541148

C -2.263185 4.499028 -0.435570

H -1.506424 5.257051 -0.629220

H -2.186482 4.204540 0.609741

S -3.909948 5.250654 -0.667603

C -3.725203 5.948669 -2.369964

H -4.748628 6.171362 -2.671600

H -3.364470 5.138167 -3.007733

C -2.840080 7.167484 -2.407914

H -2.812783 7.553516 -3.426421

H -3.210807 7.959874 -1.761113

H -1.811293 6.946465 -2.126282

Co -5.227674 3.255242 -0.936576

P -7.374697 2.038418 -3.375931

O -8.307199 3.071027 -4.123926

O -7.410689 0.719264 -4.254972

C -6.498546 -0.417835 -4.066262

H -5.487856 -0.051164 -4.262879

H -6.561650 -0.735850 -3.021250

C -9.637313 2.675034 -4.663098

H -10.126067 2.042152 -3.917965

H -9.443242 2.080186 -5.554578

C -10.394854 3.930503 -4.947428

H -9.870229 4.555473 -5.666873

H -11.363628 3.670846 -5.372032

H -10.571618 4.509045 -4.041964

C -6.917016 -1.496071 -5.014361

H -6.863129 -1.155931 -6.046238

H -6.250339 -2.350288 -4.904821

H -7.930365 -1.833452 -4.807783

S -5.553876 2.870684 -3.191510

S -8.293734 1.472814 -1.569679

C -8.594076 3.160043 -0.912265

H -9.511457 3.098753 -0.329422

H -8.764164 3.855762 -1.732918

S -7.294679 3.875876 0.144094

C -7.409860 2.775679 1.625492

H -6.519001 3.027964 2.201260

H -7.296326 1.747872 1.273002

C -8.674185 2.989040 2.416600

H -8.651377 2.354044 3.301496

H -8.776669 4.020080 2.748871

H -9.568338 2.716444 1.857112

P1-2LIGAND2222pq-CuIId

61

P1-2LIGAND2222pq-CuIId SCF Done: -5567.06628518 A.U.

P -2.542000 1.485535 0.073020

O -1.581935 1.607143 1.320226

O -2.414942 -0.019960 -0.418375

C -3.455252 -0.762274 -1.129429

H -3.108471 -1.791119 -1.071439

H -4.382227 -0.675676 -0.557696

C -0.232613 0.981015 1.333263

H 0.265541 1.250322 0.398360

H -0.389305 -0.097106 1.350539

C 0.483466 1.490029 2.541052

H -0.048788 1.233450 3.454463

H 1.469405 1.029479 2.586295

H 0.618146 2.569185 2.503473

C -3.617743 -0.293041 -2.544719

H -2.669490 -0.298813 -3.080997

H -4.298528 -0.967578 -3.063647

H -4.042853 0.714822 -2.592588

S -4.353428 2.097847 0.675379

S -1.769764 2.600061 -1.529638

C -2.008969 4.255599 -0.773660

H -1.202070 4.887356 -1.141224

H -1.924183 4.196359 0.309927

S -3.589448 5.075353 -1.157032

C -3.289822 5.575272 -2.904243

H -4.273053 5.894363 -3.251221

H -3.018335 4.673986 -3.459516

C -2.267145 6.677157 -3.034791

H -2.189543 6.963359 -4.083092

H -2.549204 7.561801 -2.467976

H -1.271791 6.367585 -2.718256

Cu -5.164235 3.210641 -1.172232

P -7.198016 2.074797 -3.536681

O -8.582502 2.828785 -3.442098

O -7.246377 1.272722 -4.899934

C -6.151719 0.417836 -5.395184

H -5.213848 0.961691 -5.255776

H -6.141599 -0.478222 -4.769684

C -9.861489 2.167539 -3.814730

H -9.863994 1.165536 -3.376063

H -9.855376 2.069034 -4.899271

C -10.970450 3.032559 -3.310845

H -10.926647 4.025696 -3.752477

H -11.924430 2.584403 -3.585215

H -10.945374 3.133137 -2.226490

C -6.431669 0.105786 -6.830185

H -6.450931 1.009181 -7.435523

H -5.645341 -0.542371 -7.214239

H -7.380156 -0.414790 -6.943348

S -5.678646 3.370683 -3.422253

S -7.124267 0.633456 -1.977955

C -8.054522 1.543264 -0.695209

H -7.930661 0.945365 0.207297

H -9.108734 1.573867 -0.962590

S -7.495010 3.257403 -0.348333

C -7.510556 3.249409 1.495789

H -7.016736 4.185811 1.758523

H -6.873767 2.429248 1.831684

C -8.910585 3.168582 2.053427

H -8.863964 3.211774 3.140983

H -9.530651 3.995284 1.712428

H -9.405773 2.234438 1.789702

P1-2LIGAND2222pq-FeIIIs

61

P1-2LIGAND2222pq-FeIIIs SCF Done: -5189.84243032 A.U.

P -2.349817 1.073910 -0.217708

O -1.637911 1.564665 1.082172

O -1.675915 -0.258343 -0.677755

C -2.075076 -1.144236 -1.816089

H -3.133432 -1.372389 -1.672746

H -1.942896 -0.556851 -2.726943

C -0.139040 1.403734 1.319188

H 0.358847 1.763578 0.417013

H 0.019688 0.332129 1.422345

C 0.194039 2.184072 2.541085

H -0.355220 1.826661 3.409010

H 1.257149 2.052963 2.745196

H 0.013602 3.249530 2.410990

C -1.199948 -2.351019 -1.767311

H -1.336406 -2.906912 -0.842579

H -1.467062 -3.006484 -2.595530

H -0.150497 -2.087412 -1.877371

S -4.342039 0.914827 0.247914

S -2.079763 2.468839 -1.785502

C -2.441361 3.997649 -0.824906

H -1.831656 4.787327 -1.262947

H -2.139941 3.865410 0.213585

S -4.183258 4.533635 -0.851956

C -4.264198 5.452443 -2.470042

H -5.333549 5.488507 -2.686847

H -3.791662 4.811735 -3.217253

C -3.642412 6.820166 -2.370554

H -3.737438 7.312708 -3.338544

H -4.136807 7.443683 -1.629106

H -2.578678 6.782689 -2.139227

Fe -5.289346 2.310631 -1.300420

P -7.231786 1.799384 -3.644491

O -8.020042 3.154219 -3.598953

O -7.812607 0.957811 -4.820385

C -7.321087 -0.366726 -5.357051

H -6.263056 -0.228123 -5.581362

H -7.441424 -1.083413 -4.543865

C -9.337780 3.415628 -4.323523

H -10.005935 2.593366 -4.066371

H -9.099422 3.369234 -5.383853

C -9.819513 4.748805 -3.871382

H -9.110416 5.537635 -4.112808

H -10.748686 4.970048 -4.397490

H -10.036126 4.770574 -2.804358

C -8.146662 -0.688262 -6.554432

H -8.029834 0.057658 -7.336971

H -7.807053 -1.644112 -6.953063

H -9.199811 -0.790151 -6.302832

S -5.226177 2.082949 -3.631299

S -7.561814 0.808358 -1.740509

C -8.482623 2.105488 -0.827546

H -8.872691 1.625886 0.070233

H -9.313000 2.488541 -1.415168

S -7.343435 3.460874 -0.377895

C -7.227731 3.290222 1.477940

H -6.363603 3.908511 1.725166

H -6.976665 2.249304 1.687579

C -8.487908 3.753003 2.158948

H -8.346334 3.660011 3.236197

H -8.713695 4.794663 1.942682

H -9.353450 3.144829 1.899675

P1-2LIGAND2222pq-MnIIs

61

P1-2LIGAND2222pq-MnIIs SCF Done: -5077.63073756 A.U.

P -2.273072 1.692625 -0.393705

O -1.114428 1.647647 0.671760

O -2.056932 0.404711 -1.301566

C -3.026578 -0.028674 -2.309923

H -4.021461 0.007666 -1.853015

H -2.997950 0.692841 -3.131501

C 0.287980 1.325071 0.294284

H 0.563260 1.979641 -0.536616

H 0.289247 0.292675 -0.053569

C 1.134899 1.536145 1.505974

H 0.816989 0.896810 2.326653

H 2.165717 1.282785 1.262698

H 1.112654 2.572080 1.837607

C -2.646396 -1.408747 -2.747314

H -2.677065 -2.110258 -1.916326

H -3.345913 -1.752642 -3.508693

H -1.649040 -1.424460 -3.181634

S -3.998389 1.807661 0.602167

S -1.960346 3.273286 -1.760640

C -2.286029 4.700662 -0.645633

H -1.558551 5.468179 -0.902258

H -2.128368 4.420179 0.394751

S -3.958760 5.418878 -0.760593

C -3.953748 6.061609 -2.495510

H -4.988068 6.370079 -2.650333

H -3.761011 5.216905 -3.160365

C -2.985463 7.197424 -2.698328

H -3.093858 7.580757 -3.712380

H -3.171327 8.019190 -2.009699

H -1.947464 6.884404 -2.590873

Mn -5.256182 3.242462 -0.900248

P -7.467252 1.864882 -3.205942

O -8.509509 2.879808 -3.824409

O -7.587745 0.547121 -4.079141

C -6.627109 -0.564847 -4.020938

H -5.649038 -0.158941 -4.290667

H -6.589214 -0.923946 -2.988445

C -9.874487 2.446406 -4.231258

H -10.294293 1.855294 -3.413086

H -9.749905 1.801071 -5.099677

C -10.662854 3.680850 -4.524552

H -10.202655 4.264287 -5.318912

H -11.660670 3.393943 -4.853515

H -10.770506 4.310842 -3.642960

C -7.094753 -1.620049 -4.971745

H -7.141314 -1.240608 -5.990250

H -6.393623 -2.453456 -4.954095

H -8.074825 -2.000267 -4.692036

S -5.649587 2.698179 -3.229722

S -8.175714 1.271178 -1.301180

C -8.639527 2.924401 -0.651793

H -9.523280 2.770006 -0.035363

H -8.913003 3.585077 -1.473391

S -7.393205 3.797144 0.353291

C -7.301910 2.690231 1.835713

H -6.439011 3.070599 2.383277

H -7.053674 1.688399 1.477485

C -8.557579 2.719694 2.667289

H -8.411521 2.099062 3.550741

H -8.800124 3.725988 3.002612

H -9.418833 2.314121 2.137468

P1-2LIGAND2222pq-NiII

61

P1-2LIGAND2222pq-NiII SCF Done: -5434.88187396 A.U.

P -2.858638 1.413299 -0.162464

O -1.510871 2.058325 0.344552

O -2.708101 -0.141236 0.106043

C -3.766613 -1.126949 -0.163692

H -4.678945 -0.769987 0.321556

H -3.927779 -1.150821 -1.244970

C -0.186514 1.449935 0.049393

H -0.174807 1.169772 -1.007342

H -0.120904 0.543792 0.650151

C 0.855356 2.461908 0.398131

H 0.800723 2.739306 1.448414

H 1.840344 2.035418 0.213980

H 0.761537 3.363154 -0.205961

C -3.304700 -2.444562 0.371980

H -3.141898 -2.398694 1.446564

H -4.065820 -3.198208 0.176507

H -2.383491 -2.765129 -0.109760

S -4.417258 2.256454 0.788099

S -2.967695 1.602505 -2.268515

C -2.484303 3.353692 -2.426386

H -2.742537 3.628560 -3.448410

H -1.411845 3.461090 -2.275418

S -3.352426 4.491492 -1.272788

C -3.495439 5.992185 -2.337298

H -4.174949 6.637045 -1.778876

H -3.988926 5.701835 -3.265259

C -2.152175 6.641184 -2.564180

H -2.295746 7.554937 -3.139963

H -1.666764 6.911410 -1.628360

H -1.476232 6.005514 -3.134939

Ni -5.358279 3.467227 -0.888105

P -7.363854 2.611536 -3.384064

O -8.890745 3.000651 -3.312995

O -7.122176 2.121536 -4.873171

C -5.798827 1.771931 -5.411634

H -5.136388 2.620596 -5.222725

H -5.430230 0.908149 -4.851579

C -9.947637 2.143879 -3.913764

H -9.735769 1.108020 -3.634962

H -9.848822 2.240647 -4.994049

C -11.262575 2.630918 -3.398591

H -11.431462 3.671161 -3.667939

H -12.059552 2.035357 -3.841607

H -11.333397 2.534590 -2.316303

C -5.964302 1.480295 -6.868853

H -6.343719 2.347861 -7.404467

H -4.997104 1.216662 -7.294204

H -6.641109 0.644158 -7.030659

S -6.232379 4.187966 -2.855079

S -7.023088 0.898628 -2.190997

C -7.882979 1.416348 -0.667654

H -7.562388 0.703547 0.091745

H -8.960796 1.350594 -0.804957

S -7.469812 3.112923 -0.092599

C -7.506032 2.859251 1.734248

H -7.076110 3.781603 2.126682

H -6.827904 2.039923 1.975533

C -8.909323 2.626654 2.237862

H -8.885207 2.555139 3.324657

H -9.578312 3.443456 1.974354

H -9.334679 1.695627 1.865060

P1-2LIGAND2222pq-NiIIt

61

P1-2LIGAND2222pq-NiIIt SCF Done: -5434.90093925 A.U.

P -2.634306 1.498766 -0.759338

O -1.887524 1.624706 0.629218

O -2.178812 0.100840 -1.354543

C -2.801930 -0.536125 -2.523912

H -3.841492 -0.742278 -2.257839

H -2.786360 0.179363 -3.351263

C -0.447473 1.276304 0.770382

H 0.086027 1.704095 -0.082160

H -0.386461 0.190394 0.712472

C 0.018740 1.822752 2.080045

H -0.551017 1.407185 2.908405

H 1.064206 1.553366 2.223704

H -0.053381 2.908712 2.112722

C -2.025295 -1.777287 -2.828669

H -2.038168 -2.466083 -1.986746

H -2.472705 -2.281943 -3.683734

H -0.992152 -1.546906 -3.078770

S -4.610897 1.626544 -0.439198

S -1.819037 2.907788 -2.091343

C -2.055086 4.394234 -1.051467

H -1.248164 5.076933 -1.311978

H -1.954771 4.145783 0.004597

S -3.628605 5.292406 -1.246927

C -3.597185 5.687526 -3.050139

H -4.560419 6.169242 -3.220354

H -3.596496 4.735899 -3.585214

C -2.442620 6.577541 -3.430683

H -2.542945 6.854503 -4.479569

H -2.421341 7.495482 -2.846479

H -1.479893 6.077549 -3.329771

Ni -5.400871 3.769163 -0.798173

P -7.878178 2.517805 -3.005959

O -9.087486 3.207197 -3.747167

O -7.545807 1.233002 -3.882806

C -6.366642 0.394745 -3.661242

H -5.493996 1.054081 -3.608156

H -6.483331 -0.098717 -2.691973

C -10.247957 2.422053 -4.243898

H -10.555202 1.743470 -3.443846

H -9.887526 1.832837 -5.086323

C -11.317016 3.393870 -4.622028

H -10.974481 4.078165 -5.395232

H -12.170553 2.843203 -5.015127

H -11.656288 3.972725 -3.765458

C -6.287096 -0.579341 -4.795343

H -6.180443 -0.067226 -5.749301

H -5.421751 -1.226982 -4.660382

H -7.171357 -1.211543 -4.835945

S -6.435179 3.902655 -2.867109

S -8.503488 1.705014 -1.171287

C -8.687442 3.274680 -0.251476

H -9.519725 3.140951 0.436820

H -8.931638 4.097254 -0.921760

S -7.232301 3.792432 0.718859

C -7.066632 2.379747 1.892550

H -6.101092 2.551154 2.368202

H -6.979162 1.475083 1.287167

C -8.196010 2.313746 2.887929

H -8.003598 1.500540 3.586910

H -8.289381 3.232155 3.463972

H -9.155180 2.102853 2.416103

P1-2LIGAND2222pq-ZnII

61

P1-2LIGAND2222pq-ZnII SCF Done: -5705.96071795 A.U.

P -2.732878 1.226332 -0.182683

O -2.024678 0.814464 1.163208

O -2.344209 0.090996 -1.222497

C -2.942434 -0.000474 -2.560316

H -3.604776 -0.866029 -2.517063

H -3.553475 0.891521 -2.739870

C -0.622277 0.316608 1.184015

H -0.018577 1.006857 0.589430

H -0.630042 -0.656693 0.694630

C -0.192584 0.251522 2.612819

H -0.831536 -0.414794 3.188143

H 0.823875 -0.137219 2.656769

H -0.196947 1.234172 3.080334

C -1.851167 -0.156782 -3.572333

H -1.243016 -1.033708 -3.361227

H -2.288658 -0.283347 -4.561975

H -1.202746 0.717481 -3.601484

S -4.681303 1.431111 0.242820

S -1.808165 2.915438 -1.030590

C -2.367383 4.201472 0.148433

H -1.534192 4.888612 0.280497

H -2.599766 3.763265 1.118173

S -3.831405 5.166685 -0.363667

C -3.273646 5.833349 -1.993545

H -4.166172 6.325489 -2.380925

H -3.057043 4.979778 -2.638928

C -2.106952 6.778262 -1.872037

H -1.896192 7.208861 -2.850219

H -2.312441 7.597962 -1.186172

H -1.197052 6.274141 -1.547916

Zn -5.362009 3.350859 -0.922539

P -7.313063 2.370759 -3.613724

O -8.109887 3.232256 -4.665904

O -7.184528 0.931089 -4.268342

C -6.392799 -0.151391 -3.667951

H -5.501576 -0.234992 -4.291132

H -6.083173 0.150374 -2.660889

C -9.305470 2.691328 -5.368588

H -9.961675 2.254389 -4.611679

H -8.948081 1.898647 -6.025032

C -9.939799 3.824555 -6.105654

H -9.252952 4.260874 -6.827389

H -10.805143 3.449319 -6.650240

H -10.281528 4.604776 -5.428395

C -7.215817 -1.401057 -3.658227

H -7.536630 -1.666852 -4.663284

H -6.620965 -2.225754 -3.267252

H -8.097356 -1.293193 -3.027933

S -5.597980 3.346023 -3.256371

S -8.505490 1.972803 -1.925238

C -8.630070 3.666956 -1.236145

H -9.640394 3.769231 -0.845025

H -8.496428 4.413419 -2.017941

S -7.455560 4.073155 0.100066

C -7.846344 2.753665 1.332494

H -7.063504 2.867917 2.082668

H -7.696329 1.792233 0.836400

C -9.226908 2.894671 1.919074

H -9.364539 2.137212 2.689584

H -9.378077 3.868645 2.380169

H -10.010463 2.736809 1.178571

P1-2LIGAND2222td-CoIIq

61

P1-2LIGAND2222td-CoIIq SCF Done: -5309.36270332 A.U.

P -2.868796 1.136078 0.279681

O -2.372452 2.421427 1.061586

O -2.267391 -0.098128 1.068188

C -2.772939 -1.475665 1.003125

H -2.207530 -1.979456 1.782884

H -3.824393 -1.456982 1.294962

C -0.986765 2.517809 1.591359

H -0.301468 2.159774 0.817222

H -0.928459 1.837933 2.440206

C -0.754790 3.945789 1.965794

H -1.457639 4.272164 2.729217

H 0.251505 4.050656 2.368549

H -0.842805 4.610605 1.106725

C -2.556730 -2.083092 -0.350046

H -1.510734 -2.034876 -0.648910

H -2.846943 -3.132479 -0.328292

H -3.164051 -1.589486 -1.113481

S -4.869478 1.121290 0.169984

S -1.945062 1.172466 -1.629882

C -1.760354 2.980844 -1.816733

H -1.237412 3.113271 -2.763098

H -1.135252 3.374219 -1.019797

S -3.296525 3.991437 -1.867002

C -3.313766 4.530298 -3.640176

H -4.331263 4.895201 -3.789146

H -3.191066 3.634327 -4.250718

C -2.281318 5.587696 -3.931608

H -2.352396 5.878685 -4.979226

H -2.431538 6.478138 -3.324707

H -1.264928 5.230060 -3.767760

Co -5.133844 2.440858 -1.724181

P -7.109834 0.583674 -3.633085

O -7.966865 0.788334 -4.937265

O -6.957680 -0.986527 -3.455723

C -5.985038 -1.593648 -2.538324

H -5.021259 -1.106661 -2.716728

H -6.304964 -1.366774 -1.517390

C -9.173478 -0.034452 -5.230789

H -9.816167 0.000012 -4.347829

H -8.826551 -1.056949 -5.376641

C -9.820528 0.545470 -6.445000

H -9.146877 0.528211 -7.298833

H -10.695979 -0.051786 -6.695828

H -10.149413 1.568805 -6.275669

C -5.951106 -3.062776 -2.817156

H -5.638056 -3.264382 -3.839328

H -5.238749 -3.539271 -2.145272

H -6.924089 -3.520134 -2.651728

S -5.403539 1.615074 -3.860909

S -8.208516 1.144604 -1.922515

C -8.291253 2.954302 -2.227186

H -9.261585 3.288020 -1.864234

H -8.238838 3.164989 -3.294242

S -6.991382 3.944798 -1.416950

C -7.456007 3.740227 0.360863

H -6.569482 4.076289 0.899059

H -7.558504 2.667413 0.539034

C -8.686903 4.522847 0.737711

H -8.878130 4.391813 1.802333

H -8.566001 5.587635 0.549817

H -9.577126 4.180018 0.211825

P1-2LIGAND2222td-CuIId

61

P1-2LIGAND2222td-CuIId SCF Done: -5567.06894769 A.U.

P -2.242686 2.369860 -0.098405

O -1.285434 2.933301 1.016861

O -2.243166 0.791831 0.133119

C -3.127073 -0.107920 -0.602399

H -4.155012 0.251256 -0.468863

H -2.877553 -0.038748 -1.665925

C 0.047751 2.331325 1.286429

H 0.573584 2.261686 0.330675

H -0.131759 1.326145 1.667118

C 0.746007 3.214316 2.267477

H 0.188695 3.289515 3.198651

H 1.721246 2.785095 2.493478

H 0.902086 4.213885 1.867764

C -2.923393 -1.487123 -0.054726

H -3.183183 -1.536805 1.000457

H -3.552834 -2.192841 -0.594920

H -1.890395 -1.807184 -0.174037

S -3.986844 3.329255 0.141709

S -1.408674 2.556970 -2.018546

C -2.011211 4.230444 -2.440074

H -1.268536 4.670425 -3.103530

H -2.095127 4.853854 -1.552289

S -3.626790 4.276498 -3.279899

C -3.254527 3.311809 -4.801613

H -4.216254 3.268416 -5.312334

H -2.995764 2.297019 -4.488920

C -2.185271 3.944575 -5.657532

H -2.107772 3.387627 -6.590516

H -2.417739 4.977981 -5.907355

H -1.202088 3.911454 -5.189736

Cu -5.103147 2.996264 -1.846104

P -7.389599 0.510108 -3.050961

O -8.375232 0.794903 -4.250602

O -7.263939 -1.069998 -2.975049

C -6.296092 -1.775467 -2.125875

H -5.301329 -1.411847 -2.398207

H -6.496612 -1.503088 -1.085571

C -9.657631 0.055491 -4.400044

H -10.073561 -0.103680 -3.401957

H -9.401406 -0.911137 -4.832072

C -10.549126 0.874800 -5.274778

H -10.096352 1.051380 -6.247965

H -11.483111 0.337001 -5.431920

H -10.788603 1.834436 -4.819035

C -6.462082 -3.241713 -2.371772

H -6.265756 -3.492285 -3.411925

H -5.757055 -3.793752 -1.751719

H -7.465154 -3.574410 -2.113768

S -5.669714 1.465310 -3.460708

S -8.323652 1.040392 -1.248338

C -8.508700 2.825314 -1.620249

H -9.435888 3.143665 -1.146658

H -8.608326 2.983889 -2.693473

S -7.176507 3.918054 -1.038110

C -7.263767 3.650658 0.783567

H -6.494952 4.320511 1.168979

H -6.941978 2.625884 0.979907

C -8.618884 3.961710 1.367073

H -8.553432 3.911871 2.453119

H -8.960774 4.960972 1.102624

H -9.378399 3.240880 1.066207

P1-2LIGAND2222td-FeIIIs

61

P1-2LIGAND2222td-FeIIIs SCF Done: -5189.84469268 A.U.

P -2.686323 1.142306 -0.209724

O -1.635061 1.926408 0.654688

O -2.637072 -0.418907 -0.102818

C -1.369808 -1.244509 -0.222483

H -1.763884 -2.199411 -0.558919

H -0.769720 -0.804127 -1.020192

C -1.704988 2.046054 2.167022

H -1.891016 1.042028 2.550765

H -2.566176 2.682440 2.372121

C -0.412311 2.625096 2.626663

H -0.237980 3.613078 2.206299

H -0.454189 2.729197 3.710985

H 0.430662 1.980172 2.388523

C -0.657456 -1.324578 1.085106

H -1.310309 -1.686129 1.877548

H 0.159665 -2.039269 0.984916

H -0.214253 -0.372803 1.374972

S -4.611697 1.699045 0.210243

S -2.164170 1.619764 -2.189213

C -2.080227 3.453438 -2.049883

H -1.317354 3.790392 -2.750924

H -1.778044 3.741361 -1.044780

S -3.658334 4.263865 -2.457578

C -3.585062 4.280135 -4.321542

H -4.614362 4.492785 -4.613284

H -3.344204 3.262723 -4.636981

C -2.615958 5.311387 -4.836751

H -2.644946 5.290776 -5.926527

H -2.877254 6.317310 -4.516266

H -1.586129 5.107858 -4.546099

Fe -5.198593 2.342833 -1.922860

P -7.302602 0.248261 -3.259477

O -8.255870 0.630207 -4.432979

O -7.440663 -1.285794 -2.990071

C -6.664521 -2.105829 -2.007326

H -5.608889 -1.931249 -2.225596

H -6.906066 -1.714676 -1.016690

C -9.554871 -0.108954 -4.761724

H -10.176323 -0.041936 -3.867854

H -9.263905 -1.143693 -4.930644

C -10.139414 0.558116 -5.955132

H -9.473253 0.507099 -6.813128

H -11.056972 0.029957 -6.216996

H -10.402178 1.595755 -5.759641

C -7.078712 -3.526991 -2.190155

H -6.847302 -3.885673 -3.190477

H -6.530604 -4.142319 -1.477360

H -8.140383 -3.661568 -1.996467

S -5.417714 0.888014 -3.703201

S -7.916318 1.237649 -1.464347

C -8.387791 2.860433 -2.194334

H -9.231368 3.240808 -1.619327

H -8.696778 2.732525 -3.231227

S -7.018995 4.060876 -2.133410

C -7.207285 4.750802 -0.410621

H -6.243180 5.225076 -0.222440

H -7.309521 3.899229 0.265171

C -8.355612 5.721841 -0.323895

H -8.397342 6.110046 0.694220

H -8.230312 6.566411 -0.997483

H -9.319178 5.254899 -0.523680

P1-2LIGAND2222td-MnIIs

61

P1-2LIGAND2222td-MnIIs SCF Done: -5077.63081625 A.U.

P -2.338636 1.965277 -0.040917

O -1.289323 2.645137 0.917226

O -2.108037 0.400208 0.122379

C -3.021291 -0.597924 -0.438635

H -4.031825 -0.348018 -0.099794

H -2.989136 -0.508330 -1.528755

C 0.119998 2.173516 0.994746

H 0.498286 2.099029 -0.027985

H 0.091674 1.176906 1.434113

C 0.875005 3.159178 1.824526

H 0.456999 3.235749 2.825829

H 1.907244 2.823995 1.916142

H 0.881861 4.147594 1.369303

C -2.574698 -1.945016 0.036939

H -2.609469 -2.012450 1.122062

H -3.235551 -2.709267 -0.371003

H -1.562996 -2.166807 -0.295768

S -4.145470 2.656687 0.444809

S -1.804460 2.296024 -2.058496

C -2.093492 4.111939 -2.136634

H -1.287793 4.529855 -2.737000

H -2.036731 4.550454 -1.141486

S -3.686771 4.616668 -2.863235

C -3.470204 4.032144 -4.605284

H -4.470471 4.117023 -5.030157

H -3.220928 2.969218 -4.564412

C -2.455596 4.841033 -5.370908

H -2.417165 4.483451 -6.399309

H -2.712730 5.897838 -5.394367

H -1.449734 4.740257 -4.964586

Mn -5.132029 2.827137 -1.770174

P -7.179638 0.276685 -2.935681

O -8.133372 0.638120 -4.143328

O -7.265217 -1.299750 -2.789852

C -6.374678 -2.097305 -1.934556

H -5.348407 -1.875776 -2.238742

H -6.518436 -1.766995 -0.901922

C -9.465887 -0.007068 -4.302079

H -9.957632 -0.004032 -3.325697

H -9.276895 -1.039154 -4.594052

C -10.215989 0.768710 -5.334830

H -9.688344 0.775372 -6.286122

H -11.186646 0.300991 -5.493003

H -10.388711 1.797333 -5.021653

C -6.733077 -3.536788 -2.124398

H -6.598407 -3.843558 -3.159468

H -6.085192 -4.152691 -1.502458

H -7.762256 -3.730779 -1.830521

S -5.343107 0.968494 -3.313315

S -8.069514 1.004861 -1.156341

C -8.528135 2.681442 -1.750961

H -9.465330 2.932532 -1.257218

H -8.706405 2.657288 -2.825199

S -7.339708 4.029631 -1.432197

C -7.454350 4.171420 0.411225

H -6.618684 4.822752 0.668884

H -7.246092 3.183018 0.827050

C -8.773163 4.735416 0.871529

H -8.754365 4.842029 1.955579

H -8.966642 5.716401 0.442694

H -9.612731 4.082931 0.634199

P1-2LIGAND2222td-NiIIt

61

P1-2LIGAND2222td-NiIIt SCF Done: -5434.90784640 A.U.

P -2.898861 1.271685 0.463316

O -2.082284 2.530142 0.962858

O -2.267735 0.037321 1.228271

C -2.942053 -1.253814 1.425845

H -2.288378 -1.767635 2.126004

H -3.895700 -1.057361 1.918316

C -0.630591 2.449950 1.283336

H -0.128801 1.956256 0.446294

H -0.538286 1.813223 2.161762

C -0.152622 3.846024 1.514941

H -0.681829 4.311279 2.343534

H 0.907157 3.821941 1.764140

H -0.275801 4.469550 0.630135

C -3.099635 -1.991397 0.131172

H -2.143114 -2.127799 -0.371527

H -3.524846 -2.975903 0.320618

H -3.780879 -1.462977 -0.541594

S -4.858694 1.514184 0.671615

S -2.446947 1.041337 -1.621177

C -2.041105 2.779322 -2.042395

H -1.830380 2.761317 -3.111485

H -1.151673 3.098241 -1.504802

S -3.417960 3.951166 -1.723113

C -3.584765 4.770393 -3.371337

H -4.541073 5.290245 -3.298257

H -3.691980 3.987406 -4.123198

C -2.442516 5.710913 -3.659856

H -2.610868 6.191614 -4.622950

H -2.359167 6.492272 -2.907235

H -1.486393 5.191898 -3.723623

Ni -5.095236 2.228998 -1.561513

P -7.001095 0.630287 -3.763464

O -7.828733 0.848262 -5.084912

O -6.757585 -0.940058 -3.671813

C -5.783060 -1.521933 -2.747679

H -4.796294 -1.148899 -3.038512

H -6.009926 -1.157716 -1.738411

C -8.958626 -0.040885 -5.468475

H -9.623246 -0.118872 -4.604507

H -8.530127 -1.021239 -5.674402

C -9.620518 0.572279 -6.658367

H -8.927098 0.667009 -7.491166

H -10.440584 -0.070508 -6.975484

H -10.031649 1.553206 -6.428760

C -5.888772 -3.011339 -2.852089

H -5.682437 -3.346978 -3.866132

H -5.159159 -3.475295 -2.190335

H -6.877760 -3.360266 -2.562663

S -5.394041 1.807764 -3.842260

S -8.185864 1.007506 -2.057864

C -8.253225 2.837041 -2.199802

H -9.227114 3.146597 -1.825064

H -8.177431 3.141450 -3.241892

S -6.964673 3.732460 -1.274716

C -7.573852 3.513256 0.451001

H -6.730065 3.834001 1.062253

H -7.706253 2.440853 0.613284

C -8.820452 4.309907 0.741743

H -9.092883 4.173332 1.787726

H -8.670588 5.374250 0.572817

H -9.674349 3.984779 0.148589

P1-2LIGAND2222td-ZnII

61

P1-2LIGAND2222td-ZnII SCF Done: -5705.96150401 A.U.

P -2.718248 1.511965 0.018273

O -2.144967 2.388218 1.201969

O -2.196227 0.039552 0.289160

C -2.722123 -1.155807 -0.387202

H -3.769595 -1.251469 -0.091789

H -2.674009 -0.986439 -1.467112

C -0.733827 2.250319 1.653871

H -0.105490 2.146965 0.765675

H -0.684564 1.325454 2.227197

C -0.402359 3.462503 2.461380

H -1.065322 3.561459 3.318100

H 0.616813 3.372261 2.834501

H -0.459959 4.371921 1.865082

C -1.892558 -2.322921 0.044330

H -1.941625 -2.462915 1.122008

H -2.270125 -3.227420 -0.430769

H -0.851762 -2.200498 -0.247558

S -4.713488 1.693018 0.011776

S -1.727513 2.070558 -1.756591

C -2.065138 3.869812 -1.676185

H -1.185153 4.367624 -2.078996

H -2.185138 4.187891 -0.640871

S -3.520520 4.476060 -2.595573

C -3.085496 3.978796 -4.323453

H -3.981115 4.235386 -4.889894

H -2.981622 2.891877 -4.336383

C -1.860435 4.684887 -4.842424

H -1.718637 4.422552 -5.890255

H -1.954190 5.767305 -4.781121

H -0.953762 4.383406 -4.318553

Zn -5.229552 2.868189 -1.956463

P -7.388978 0.676979 -3.435261

O -8.313148 0.676082 -4.711215

O -7.192015 -0.857090 -3.068380

C -6.239523 -1.320501 -2.056872

H -5.313644 -0.750864 -2.186528

H -6.654384 -1.081401 -1.074007

C -9.520896 -0.186817 -4.804882

H -10.108542 -0.029321 -3.897004

H -9.169336 -1.217801 -4.823634

C -10.253479 0.205651 -6.045910

H -9.635805 0.066292 -6.930456

H -11.134644 -0.426089 -6.148397

H -10.584561 1.241328 -6.007382

C -6.047077 -2.791774 -2.254661

H -5.624074 -3.008735 -3.233375

H -5.367824 -3.176207 -1.494837

H -6.988676 -3.327727 -2.155635

S -5.742158 1.701892 -3.930785

S -8.425744 1.421090 -1.760930

C -8.486221 3.197350 -2.218615

H -9.457031 3.569978 -1.898970

H -8.415938 3.321501 -3.298185

S -7.192613 4.249162 -1.476418

C -7.524451 3.998751 0.323123

H -6.654671 4.442865 0.807658

H -7.492753 2.922835 0.510249

C -8.815642 4.631887 0.772143

H -8.912095 4.515936 1.850943

H -8.848055 5.696168 0.547988

H -9.688794 4.158115 0.324846

P1-2LIGAND2222td-ZnIIt

61

P1-2LIGAND2222td-ZnIIt SCF Done: -5705.86554726 A.U.

P -2.896446 1.001158 0.361222

O -2.369277 2.306575 1.125317

O -2.461538 -0.203715 1.336229

C -2.877113 -1.585370 1.106102

H -2.455439 -2.119873 1.954406

H -3.966904 -1.622119 1.182143

C -1.022668 2.367723 1.733539

H -0.299440 2.015252 0.994468

H -1.021908 1.675888 2.575872

C -0.780085 3.785848 2.139393

H -1.522479 4.122819 2.859837

H 0.199484 3.859757 2.609400

H -0.793059 4.454315 1.280180

C -2.377584 -2.113433 -0.208230

H -1.297163 -2.010213 -0.294518

H -2.618944 -3.171476 -0.297565

H -2.844708 -1.592746 -1.048378

S -4.925005 1.108924 0.180551

S -2.265186 0.898330 -3.005179

C -2.187648 2.635795 -3.396943

H -2.705314 2.834802 -4.338806

H -1.135794 2.898557 -3.533315

S -2.896292 3.732902 -2.080037

C -3.379064 5.217215 -3.075536

H -3.941331 5.828624 -2.368753

H -4.069441 4.882152 -3.852109

C -2.184291 5.948183 -3.631505

H -2.522944 6.841967 -4.154323

H -1.500367 6.262688 -2.845394

H -1.630310 5.347354 -4.351460

Zn -4.965625 2.498370 -1.685131

P -7.033178 0.568595 -3.601271

O -7.811311 0.647764 -4.967147

O -6.888538 -0.979236 -3.286512

C -6.067790 -1.507798 -2.188964

H -5.092127 -1.011820 -2.236768

H -6.549075 -1.224261 -1.249570

C -9.036289 -0.159462 -5.222795

H -9.719197 0.009124 -4.386458

H -8.727916 -1.204250 -5.224305

C -9.598176 0.287824 -6.531898

H -8.885501 0.138225 -7.339932

H -10.485535 -0.303613 -6.753300

H -9.889800 1.335558 -6.508130

C -5.972400 -2.990043 -2.362993

H -5.484108 -3.250810 -3.299792

H -5.387035 -3.408913 -1.545324

H -6.955817 -3.454887 -2.339264

S -5.335958 1.607301 -3.873433

S -8.212878 1.287325 -2.012319

C -8.119505 3.087584 -2.368393

H -9.097212 3.500514 -2.128445

H -7.928233 3.263204 -3.425645

S -6.861678 4.028478 -1.434886

C -7.434181 3.758283 0.300731

H -6.613962 4.149813 0.902623

H -7.470248 2.678746 0.462580

C -8.743209 4.442092 0.596861

H -8.986827 4.301538 1.649383

H -8.698105 5.512588 0.406396

H -9.570807 4.024326 0.024229

P1-2LIGAND2222trans+1H2Oax-CoIId

64

P1-2LIGAND2222trans+1H2Oax-CoIId SCF Done: -5385.81250053 A.U.

P -2.311096 1.640554 0.221843

O -1.245739 2.779943 0.475401

O -1.864463 0.445626 1.169441

C -2.646343 -0.785500 1.336397

H -3.653242 -0.494941 1.649150

H -2.711244 -1.277988 0.361368

C 0.203802 2.481233 0.588484

H 0.472721 1.811433 -0.233129

H 0.342261 1.948748 1.528742

C 0.932970 3.784666 0.541875

H 0.624515 4.438564 1.354621

H 2.000996 3.599385 0.646593

H 0.773656 4.303732 -0.402135

C -1.948450 -1.631035 2.354086

H -1.884656 -1.120713 3.312759

H -2.506039 -2.555030 2.499246

H -0.943702 -1.892166 2.028686

S -4.160367 2.321275 0.550983

S -2.048577 0.882022 -1.741091

C -1.933171 2.460221 -2.644024

H -1.969285 2.186485 -3.698128

H -0.987337 2.951512 -2.424897

S -3.306888 3.630696 -2.295809

C -3.568433 4.340009 -3.977870

H -4.503351 4.892985 -3.874534

H -3.744317 3.512802 -4.666713

C -2.425323 5.228395 -4.402045

H -2.657110 5.671467 -5.370055

H -2.256676 6.039439 -3.696312

H -1.493919 4.674989 -4.517885

Co -5.084591 2.308772 -1.590271

P -6.122236 -0.134923 -3.750307

O -7.669566 -0.386028 -3.954353

O -5.416016 -0.967139 -4.904862

C -3.983280 -0.876546 -5.212725

H -3.765375 0.168229 -5.449096

H -3.425217 -1.155914 -4.313774

C -8.201873 -1.739190 -4.252748

H -7.721009 -2.445957 -3.570626

H -7.896701 -1.978671 -5.270697

C -9.685515 -1.683810 -4.084068

H -10.132246 -0.954320 -4.755870

H -10.107050 -2.659998 -4.319579

H -9.966611 -1.434370 -3.062084

C -3.708603 -1.794758 -6.361210

H -4.285915 -1.510068 -7.238077

H -2.652507 -1.741774 -6.620754

H -3.941591 -2.826672 -6.108099

S -5.720579 1.823260 -3.780479

S -5.509117 -1.099099 -1.966753

C -6.818276 -0.499155 -0.852338

H -6.503349 -0.807293 0.144394

H -7.765729 -0.975388 -1.096766

S -7.063010 1.322704 -0.855484

C -7.367956 1.605473 0.939941

H -7.331832 2.691693 1.033544

H -6.520970 1.195064 1.490950

C -8.692150 1.037509 1.386827

H -8.850470 1.282440 2.436551

H -9.524586 1.449119 0.819253

H -8.723862 -0.048265 1.303126

O -6.243965 4.227582 -1.402687

H -7.019604 4.473303 -1.917896

H -6.041711 4.980563 -0.837482

P1-2LIGAND2222trans+1H2Oax-CoIIq

64

P1-2LIGAND2222trans+1H2Oax-CoIIq SCF Done: -5385.82729344 A.U.

P -2.079822 1.438233 -0.276227

O -1.516367 2.504525 0.748046

O -1.332549 0.084057 0.080501

C -1.676958 -1.220020 -0.499683

H -2.757574 -1.352823 -0.396824

H -1.423386 -1.184977 -1.562823

C -0.059446 2.631555 1.014539

H 0.459931 2.615114 0.052687

H 0.230739 1.749293 1.583636

C 0.147983 3.907526 1.763658

H -0.406278 3.911928 2.699694

H 1.206018 4.012294 1.999707

H -0.149899 4.773951 1.175228

C -0.896614 -2.263898 0.234410

H -1.151011 -2.273976 1.291908

H -1.128554 -3.244238 -0.179078

H 0.174199 -2.101738 0.132516

S -4.072932 1.307887 -0.031875

S -1.426092 1.942009 -2.203953

C -1.943311 3.710870 -2.146924

H -1.223717 4.245714 -2.763076

H -1.855059 4.082373 -1.127073

S -3.615040 4.097118 -2.723619

C -3.487861 3.647304 -4.505168

H -4.485882 3.856835 -4.890141

H -3.341751 2.566367 -4.549551

C -2.427608 4.415679 -5.250965

H -2.492404 4.174577 -6.311395

H -2.552974 5.492004 -5.147671

H -1.419193 4.152417 -4.932848

Co -5.344823 2.569236 -1.494553

P -6.652090 -0.219643 -3.376008

O -7.854568 -0.392533 -4.383390

O -5.575293 -1.304676 -3.821348

C -4.177879 -1.253520 -3.387817

H -4.079049 -0.516780 -2.582009

H -3.975762 -2.241680 -2.974732

C -8.401281 -1.730281 -4.725854

H -8.579576 -2.264602 -3.789255

H -7.626549 -2.251715 -5.287217

C -9.650570 -1.513705 -5.515604

H -9.451100 -0.951308 -6.425172

H -10.059382 -2.481800 -5.801840

H -10.406315 -0.987782 -4.935782

C -3.306619 -0.923621 -4.561838

H -3.542165 0.064468 -4.956328

H -2.259606 -0.927511 -4.259784

H -3.427887 -1.652068 -5.360610

S -6.035800 1.683943 -3.540487

S -7.189010 -0.850396 -1.457371

C -8.277603 0.567302 -1.018255

H -9.080196 0.158631 -0.407747

H -8.721897 0.996685 -1.914478

S -7.466766 1.911423 -0.111192

C -7.083780 1.072575 1.482529

H -6.450360 1.789669 2.005120

H -6.463684 0.204529 1.248741

C -8.313748 0.715617 2.278150

H -8.011768 0.284255 3.231976

H -8.930816 1.587377 2.486063

H -8.931512 -0.029511 1.777807

O -6.118162 4.437412 -1.042652

H -5.749812 5.288199 -1.310172

H -6.983032 4.599037 -0.645975

P1-2LIGAND2222trans+1H2Oax-CoIIqISOMER

64

P1-2LIGAND2222trans+1H2Oax-CoIIqISOMER SCF Done: -5385.82979709 A.U.

P -1.939379 1.381793 -0.600072

O -2.870320 2.496955 0.111729

O -1.151714 0.703797 0.596624

C -0.161842 -0.378218 0.387286

H -0.655837 -1.168391 -0.181712

H 0.652374 0.035686 -0.211690

C -2.308062 3.370458 1.172679

H -1.340659 3.741279 0.824963

H -2.139372 2.733062 2.040418

C -3.288666 4.466511 1.440166

H -4.255997 4.066515 1.745437

H -2.918571 5.086853 2.255115

H -3.427746 5.105896 0.569909

C 0.299361 -0.835737 1.732722

H -0.524324 -1.235185 2.320756

H 1.035744 -1.627394 1.604019

H 0.771169 -0.027187 2.286894

S -3.175018 0.210054 -1.605506

S -0.473963 2.414460 -1.698893

C -1.495411 3.779469 -2.386044

H -0.789350 4.352562 -2.985295

H -1.858887 4.418872 -1.582930

S -2.950617 3.372070 -3.396144

C -2.269655 2.155725 -4.594403

H -3.148429 1.867106 -5.172715

H -1.948965 1.287310 -4.015444

C -1.172869 2.724823 -5.456402

H -0.873462 1.982775 -6.195695

H -1.493777 3.617149 -5.990730

H -0.282405 2.968772 -4.877690

Co -4.611187 2.206843 -1.880721

P -7.045307 0.134101 -3.572485

O -8.503531 0.482965 -4.065723

O -6.576288 -1.071477 -4.494875

C -5.205984 -1.600878 -4.491897

H -4.527347 -0.757283 -4.648124

H -5.013488 -2.025307 -3.502969

C -9.505036 -0.581505 -4.334092

H -9.503741 -1.260239 -3.477444

H -9.157056 -1.123600 -5.212843

C -10.823898 0.088127 -4.542603

H -10.787130 0.781524 -5.379997

H -11.573054 -0.669837 -4.766927

H -11.146524 0.626865 -3.653881

C -5.111953 -2.623699 -5.579319

H -5.317635 -2.185006 -6.553401

H -4.104454 -3.036357 -5.598573

H -5.806909 -3.443639 -5.411338

S -5.951085 1.809330 -3.755922

S -7.091891 -0.718301 -1.657507

C -7.550007 0.779250 -0.694264

H -8.201217 0.435207 0.106818

H -8.117200 1.472804 -1.312932

S -6.160621 1.702060 0.024461

C -5.445330 0.436172 1.149308

H -4.490269 0.871362 1.447408

H -5.220531 -0.436160 0.533098

C -6.326088 0.117143 2.330179

H -5.817562 -0.598284 2.975714

H -6.551833 1.001072 2.923615

H -7.267917 -0.343391 2.033430

O -5.155359 4.233292 -1.437465

H -5.849218 4.445680 -0.800806

H -5.208574 4.898506 -2.134716

P1-2LIGAND2222trans+1H2Oax-CuIId

64

P1-2LIGAND2222trans+1H2Oax-CuIId SCF Done: -5643.53351157 A.U.

P -2.111537 1.921048 0.241126

O -1.078566 2.912647 0.913433

O -1.793573 0.489269 0.861675

C -2.786880 -0.541883 1.135635

H -2.236783 -1.272896 1.723507

H -3.561190 -0.103919 1.769128

C 0.343104 2.537165 1.118493

H 0.704874 2.084896 0.191418

H 0.358602 1.783993 1.905549

C 1.084477 3.782181 1.482513

H 0.683414 4.231108 2.388642

H 2.128641 3.532455 1.665045

H 1.050480 4.518279 0.681296

C -3.344142 -1.127809 -0.127898

H -2.548266 -1.485198 -0.780651

H -3.989115 -1.975224 0.105422

H -3.947859 -0.396738 -0.676886

S -3.925305 2.700582 0.528297

S -1.601389 1.701393 -1.785899

C -1.895367 3.463010 -2.209430

H -1.228212 3.701567 -3.035818

H -1.621253 4.089755 -1.362403

S -3.585113 3.912057 -2.713360

C -3.510294 3.450944 -4.495100

H -4.549117 3.478145 -4.819777

H -3.182829 2.410197 -4.541566

C -2.641517 4.384279 -5.301801

H -2.670498 4.081401 -6.347938

H -2.992333 5.412540 -5.243198

H -1.596060 4.363493 -4.995521

Cu -5.182005 2.419874 -1.453139

P -6.521245 0.076067 -3.720980

O -7.721692 -0.143620 -4.718512

O -5.289727 -0.669319 -4.405153

C -3.929459 -0.580060 -3.880549

H -3.375469 0.025956 -4.599137

H -3.943692 -0.039967 -2.924903

C -8.015485 -1.472250 -5.313480

H -8.027563 -2.202033 -4.499837

H -7.190570 -1.705895 -5.986013

C -9.330195 -1.366255 -6.014612

H -9.300940 -0.611045 -6.796999

H -9.559533 -2.323924 -6.480069

H -10.135435 -1.126016 -5.323558

C -3.363234 -1.959711 -3.745498

H -3.389443 -2.486358 -4.697328

H -2.322197 -1.897740 -3.429321

H -3.911263 -2.549407 -3.010820

S -6.408980 2.045576 -3.420597

S -6.796877 -1.013844 -1.944094

C -7.903686 0.181953 -1.106739

H -8.540945 -0.399883 -0.443299

H -8.536964 0.686967 -1.832533

S -7.081473 1.462720 -0.112614

C -6.502984 0.433037 1.296214

H -5.845750 1.100394 1.851609

H -5.888928 -0.365173 0.872079

C -7.632346 -0.084683 2.153768

H -7.215142 -0.624099 3.003702

H -8.247615 0.723826 2.543422

H -8.278576 -0.783511 1.624072

O -6.301909 4.443252 -1.111375

H -7.251216 4.593834 -1.056931

H -5.895732 5.287784 -0.890156

P1-2LIGAND2222trans+1H2Oax-FeIIId

64

P1-2LIGAND2222trans+1H2Oax-FeIIId SCF Done: -5266.27685690 A.U.

P -2.048072 1.372668 0.110689

O -1.725732 2.805743 0.677300

O -1.321760 0.303054 0.987173

C -0.693518 -0.993535 0.568494

H -1.142995 -1.721057 1.239980

H -1.014726 -1.208014 -0.453035

C -0.312025 3.204341 1.027451

H 0.328098 2.887522 0.200188

H -0.055503 2.630629 1.916883

C -0.305024 4.678177 1.249157

H -0.970654 4.962068 2.061405

H 0.703622 4.981930 1.528146

H -0.579036 5.228757 0.350409

C 0.786765 -0.876674 0.713917

H 1.065447 -0.618219 1.733399

H 1.237775 -1.842215 0.484807

H 1.208770 -0.143686 0.027401

S -4.095508 1.098076 0.279993

S -1.436131 1.168549 -1.879457

C -1.765571 2.866998 -2.466989

H -1.777829 2.781494 -3.553419

H -0.952591 3.524738 -2.165051

S -3.327062 3.688592 -1.930248

C -3.863590 4.497694 -3.515445

H -4.849440 4.896489 -3.267715

H -3.993927 3.701035 -4.249889

C -2.904019 5.572809 -3.955186

H -3.301453 6.041432 -4.855585

H -2.784004 6.351075 -3.204301

H -1.921874 5.177043 -4.210843

Fe -5.125964 2.265546 -1.271006

P -6.119124 -0.418819 -3.619049

O -7.029875 0.172357 -4.756393

O -5.231215 -1.575220 -4.183512

C -5.029620 -2.965759 -3.666197

H -4.010692 -3.185987 -3.974065

H -5.065521 -2.921661 -2.575986

C -7.794754 -0.719729 -5.708081

H -8.337763 -1.442373 -5.094967

H -7.040245 -1.237274 -6.299335

C -8.688984 0.154480 -6.518128

H -8.123111 0.887035 -7.089207

H -9.229582 -0.472788 -7.226882

H -9.424814 0.667348 -5.902210

C -6.039577 -3.889015 -4.263454

H -5.996809 -3.874834 -5.350722

H -5.818479 -4.906397 -3.940756

H -7.054638 -3.659600 -3.938376

S -4.913768 1.236628 -3.166393

S -7.223826 -1.158348 -2.017138

C -8.091799 0.422341 -1.678061

H -9.059395 0.158473 -1.253620

H -8.268653 0.976919 -2.599034

S -7.260333 1.538997 -0.511935

C -7.205519 0.505160 1.027649

H -6.706467 1.159579 1.743248

H -6.546381 -0.340385 0.823171

C -8.576869 0.086998 1.491241

H -8.475515 -0.397335 2.462357

H -9.251020 0.932292 1.616064

H -9.041171 -0.641562 0.828154

O -5.682672 3.982670 -0.308418

H -6.548650 4.148522 0.090225

H -5.057506 4.609406 0.081739

P1-2LIGAND2222trans+1H2Oax-FeIIIq

64

P1-2LIGAND2222trans+1H2Oax-FeIIIq SCF Done: -5266.30171163 A.U.

P -2.201517 1.566085 0.211284

O -1.370884 2.883382 0.374504

O -1.601910 0.496143 1.184939

C -2.030773 -0.926421 1.324666

H -3.095244 -0.908146 1.567942

H -1.885346 -1.395470 0.348616

C 0.130723 2.895451 0.571779

H 0.554421 2.171624 -0.127860

H 0.292130 2.545404 1.589637

C 0.590854 4.293301 0.340686

H 0.125463 4.987487 1.036484

H 1.667953 4.333207 0.502211

H 0.397462 4.625985 -0.678597

C -1.194283 -1.544759 2.394182

H -1.335349 -1.046203 3.350357

H -1.492821 -2.586046 2.511001

H -0.138636 -1.528763 2.133781

S -4.162087 1.990776 0.597687

S -2.009841 0.840378 -1.772209

C -1.860183 2.441433 -2.630683

H -1.910065 2.203148 -3.693171

H -0.903604 2.909123 -2.402564

S -3.191531 3.640734 -2.217129

C -3.482870 4.452622 -3.857520

H -4.387988 5.038286 -3.691936

H -3.703597 3.666281 -4.580799

C -2.304815 5.309675 -4.255146

H -2.551027 5.809804 -5.192375

H -2.087405 6.079650 -3.517879

H -1.403847 4.724313 -4.432700

Fe -5.042300 2.211894 -1.547644

P -6.074565 -0.290877 -3.740622

O -7.638200 -0.221849 -3.820407

O -5.567023 -1.137778 -4.956367

C -4.154483 -1.495790 -5.278880

H -3.614815 -0.553483 -5.393862

H -3.760757 -2.041789 -4.418261

C -8.493182 -1.412903 -4.193216

H -8.089082 -2.279949 -3.665478

H -8.351430 -1.548359 -5.263747

C -9.895662 -1.081634 -3.814215

H -10.252578 -0.197657 -4.337700

H -10.536797 -1.916222 -4.097016

H -10.005482 -0.929723 -2.740998

C -4.175438 -2.315663 -6.525428

H -4.591618 -1.758808 -7.361886

H -3.152199 -2.589378 -6.779813

H -4.741953 -3.233972 -6.388875

S -5.356115 1.626124 -3.782203

S -5.491184 -1.281221 -1.965225

C -6.820317 -0.682205 -0.874987

H -6.531792 -0.990780 0.129692

H -7.767480 -1.148955 -1.142206

S -7.090881 1.137531 -0.891381

C -7.476623 1.470331 0.892142

H -7.485854 2.559867 0.947355

H -6.635603 1.099451 1.480578

C -8.798096 0.860176 1.290462

H -8.999907 1.136010 2.325892

H -9.622990 1.229454 0.684498

H -8.785923 -0.227752 1.247242

O -6.232494 4.062469 -1.451437

H -7.086937 4.213702 -1.874418

H -6.015497 4.876432 -0.980221

P1-2LIGAND2222trans+1H2Oax-FeIIIs

64

P1-2LIGAND2222trans+1H2Oax-FeIIIs SCF Done: -5266.32709992 A.U.

P -1.845808 1.200423 -0.636495

O -2.839610 2.239742 0.107978

O -1.214541 0.262823 0.436643

C 0.231538 -0.163080 0.599617

H 0.133757 -1.210562 0.871843

H 0.703177 -0.097752 -0.381515

C -2.363778 2.923448 1.363735

H -1.390698 3.367014 1.140570

H -2.225764 2.130960 2.099476

C -3.384586 3.931880 1.770042

H -4.355843 3.469594 1.949080

H -3.069071 4.389121 2.707447

H -3.486402 4.728925 1.035546

C 0.886792 0.674303 1.643156

H 0.370860 0.603393 2.598823

H 1.901058 0.303303 1.793473

H 0.965374 1.719679 1.345387

S -3.119711 0.240289 -1.889538

S -0.345439 2.279354 -1.600375

C -1.325546 3.775383 -2.039515

H -0.604542 4.412809 -2.552178

H -1.662831 4.292795 -1.143574

S -2.787003 3.600130 -3.110483

C -2.142093 2.653818 -4.563232

H -3.026449 2.556034 -5.194322

H -1.869574 1.660155 -4.201954

C -1.010659 3.366110 -5.257335

H -0.750767 2.807229 -6.156132

H -1.281521 4.374330 -5.564832

H -0.109766 3.411960 -4.646690

Fe -4.517525 2.124735 -1.869192

P -6.848757 0.150260 -3.772014

O -8.249836 0.575747 -4.331550

O -6.072713 -0.805794 -4.740060

C -5.711608 -2.247670 -4.560974

H -4.925910 -2.379371 -5.300007

H -5.278310 -2.348821 -3.563845

C -9.267892 -0.391346 -4.881900

H -9.501846 -1.088950 -4.075511

H -8.772005 -0.921286 -5.695242

C -10.442737 0.408686 -5.329409

H -10.171622 1.116225 -6.109488

H -11.183277 -0.275801 -5.742864

H -10.908076 0.942327 -4.503913

C -6.890162 -3.134892 -4.787728

H -7.309773 -2.998338 -5.782410

H -6.566118 -4.172921 -4.711987

H -7.670657 -2.987479 -4.039685

S -5.941923 1.996522 -3.667007

S -6.974202 -0.826533 -1.920807

C -7.488463 0.598219 -0.883768

H -8.161767 0.197083 -0.127782

H -8.033631 1.339648 -1.465823

S -6.130593 1.464714 -0.016316

C -5.394050 0.088621 0.980359

H -4.524552 0.559880 1.440935

H -5.032781 -0.657372 0.269107

C -6.352638 -0.474391 1.997013

H -5.824286 -1.209546 2.603927

H -6.743467 0.288129 2.667874

H -7.190108 -0.996732 1.536225

O -5.039068 4.111952 -1.186532

H -5.690661 4.277063 -0.491671

H -5.051414 4.889004 -1.762045

P1-2LIGAND2222trans+1H2Oax-MnIId

64

P1-2LIGAND2222trans+1H2Oax-MnIId SCF Done: -5153.99960129 A.U.

P -2.139246 1.191606 -0.368109

O -1.450289 2.475930 0.240514

O -1.345505 -0.045647 0.230618

C -1.715249 -1.444713 -0.025695

H -2.796610 -1.535357 0.109421

H -1.469258 -1.657983 -1.069582

C 0.016759 2.679011 0.165653

H 0.373060 2.245574 -0.772791

H 0.446036 2.113812 0.992052

C 0.271156 4.148930 0.260389

H -0.108686 4.556784 1.194525

H 1.344176 4.331297 0.225021

H -0.191804 4.686134 -0.566791

C -0.941440 -2.302837 0.923933

H -1.187840 -2.068509 1.957113

H -1.186667 -3.348632 0.745537

H 0.130181 -2.180348 0.782656

S -4.106772 1.150232 0.087203

S -1.788180 1.096404 -2.442392

C -2.140058 2.809194 -2.963984

H -2.483222 2.723859 -3.995166

H -1.214813 3.380907 -2.928482

S -3.399414 3.755877 -1.999841

C -3.778439 5.048935 -3.267443

H -4.491841 5.692791 -2.751054

H -4.281415 4.562398 -4.107004

C -2.565933 5.836315 -3.700443

H -2.882302 6.663505 -4.334643

H -2.035118 6.257968 -2.848064

H -1.869098 5.238313 -4.284918

Mn -5.274426 2.554070 -1.306282

P -6.357421 -0.318412 -3.329139

O -7.450038 -0.275949 -4.471186

O -5.414664 -1.537975 -3.721122

C -4.188800 -1.840656 -2.975559

H -3.899899 -0.947758 -2.409475

H -4.442554 -2.634895 -2.270596

C -8.125026 -1.511624 -4.944668

H -8.374717 -2.113859 -4.067481

H -7.395068 -2.052778 -5.545592

C -9.331286 -1.095958 -5.721916

H -9.059957 -0.469652 -6.568969

H -9.827851 -1.985311 -6.107646

H -10.044048 -0.557336 -5.100107

C -3.138074 -2.250314 -3.959913

H -2.898259 -1.435196 -4.641083

H -2.227397 -2.532415 -3.432140

H -3.459841 -3.108391 -4.546298

S -5.423145 1.478731 -3.317395

S -7.207242 -0.917271 -1.509832

C -8.174910 0.604272 -1.192086

H -9.068632 0.301826 -0.650010

H -8.488980 1.058945 -2.130242

S -7.314565 1.891719 -0.233126

C -7.133392 1.055103 1.401113

H -6.521598 1.751886 1.974222

H -6.537412 0.155462 1.234773

C -8.451677 0.769568 2.072671

H -8.263291 0.361881 3.065214

H -9.053554 1.668504 2.192017

H -9.041886 0.026949 1.536776

O -6.466378 4.179305 -1.845269

H -7.387943 4.188927 -1.550527

H -6.450111 4.592034 -2.716808

P1-2LIGAND2222trans+1H2Oax-MnIIs

64

P1-2LIGAND2222trans+1H2Oax-MnIIs SCF Done: -5154.10180179 A.U.

P -2.074775 1.391837 -0.213432

O -1.455940 2.437677 0.800875

O -1.305637 0.031938 0.072221

C -1.683374 -1.254451 -0.524524

H -2.761351 -1.379251 -0.388192

H -1.465644 -1.196979 -1.595369

C 0.010797 2.548307 1.005448

H 0.487164 2.549242 0.021428

H 0.320251 1.652211 1.542061

C 0.260934 3.806490 1.771953

H -0.250601 3.793618 2.731981

H 1.329162 3.900452 1.961822

H -0.058051 4.687270 1.216598

C -0.889142 -2.323113 0.157453

H -1.108960 -2.356658 1.222298

H -1.143845 -3.291067 -0.271507

H 0.179285 -2.167662 0.024684

S -4.039801 1.262508 0.091600

S -1.465389 1.927611 -2.159054

C -1.933722 3.702986 -2.079863

H -1.193271 4.231628 -2.676419

H -1.855837 4.060075 -1.053710

S -3.585573 4.154820 -2.684555

C -3.473937 3.649529 -4.455206

H -4.462910 3.886176 -4.848229

H -3.369942 2.562808 -4.467807

C -2.379929 4.354389 -5.213641

H -2.444220 4.082462 -6.266625

H -2.466643 5.437212 -5.145140

H -1.385286 4.064229 -4.875951

Mn -5.384876 2.570044 -1.502550

P -6.709208 -0.155581 -3.432560

O -7.910045 -0.482532 -4.402680

O -5.565324 -1.185576 -3.844361

C -4.169984 -1.032514 -3.441057

H -4.100264 -0.273963 -2.650634

H -3.895415 -1.994052 -3.007151

C -8.358441 -1.876082 -4.645649

H -8.554319 -2.332014 -3.671889

H -7.528832 -2.400301 -5.119800

C -9.576070 -1.809175 -5.509037

H -9.360057 -1.328750 -6.460830

H -9.919254 -2.822360 -5.713775

H -10.385564 -1.271572 -5.020046

C -3.342435 -0.681910 -4.640987

H -3.645905 0.277484 -5.059922

H -2.290573 -0.618104 -4.363221

H -3.437854 -1.438131 -5.417263

S -6.306522 1.783716 -3.647357

S -7.159790 -0.746172 -1.468562

C -8.325592 0.608078 -1.029613

H -9.107895 0.155912 -0.423503

H -8.785879 1.019645 -1.926067

S -7.597290 1.991445 -0.104668

C -7.179991 1.150034 1.482489

H -6.532987 1.861500 1.995869

H -6.569541 0.279992 1.229806

C -8.396010 0.792989 2.298758

H -8.077487 0.339986 3.237100

H -8.996867 1.668066 2.536959

H -9.034715 0.066049 1.798026

O -6.180234 4.584036 -1.071859

H -5.780330 5.430951 -1.301467

H -7.050135 4.777752 -0.703145

P1-2LIGAND2222trans+1H2Oax-NiII

64

P1-2LIGAND2222trans+1H2Oax-NiII SCF Done: -5511.34723823 A.U.

P -2.365041 1.524786 0.196030

O -1.338388 2.682177 0.517669

O -1.925161 0.314980 1.125661

C -2.668587 -0.948760 1.216520

H -3.706840 -0.702563 1.455302

H -2.640180 -1.424680 0.232132

C 0.116940 2.422351 0.650027

H 0.418024 1.772447 -0.176353

H 0.254350 1.880533 1.585118

C 0.809890 3.746058 0.633812

H 0.471101 4.379212 1.450919

H 1.880984 3.589177 0.752228

H 0.650261 4.274199 -0.305172

C -2.015530 -1.784128 2.271290

H -2.044147 -1.289828 3.240027

H -2.544609 -2.731804 2.358941

H -0.979758 -2.000137 2.018734

S -4.235510 2.174700 0.509050

S -2.027665 0.836524 -1.775327

C -1.963246 2.444983 -2.628521

H -2.007024 2.207071 -3.690770

H -1.025675 2.950157 -2.403970

S -3.349620 3.587903 -2.224577

C -3.620964 4.371167 -3.870626

H -4.555618 4.916779 -3.733681

H -3.795933 3.577113 -4.597384

C -2.479225 5.279736 -4.255154

H -2.717532 5.768675 -5.199282

H -2.307887 6.056621 -3.512719

H -1.548158 4.733771 -4.403701

Ni -5.091173 2.242412 -1.609915

P -6.026031 -0.186154 -3.742549

O -7.552161 -0.431281 -4.069936

O -5.229995 -1.022265 -4.834643

C -3.769632 -0.982189 -4.974806

H -3.465187 0.068148 -4.947438

H -3.339846 -1.491775 -4.107826

C -8.074189 -1.786453 -4.378212

H -7.635358 -2.485230 -3.660501

H -7.714548 -2.040560 -5.374710

C -9.564466 -1.721877 -4.292533

H -9.969060 -0.996258 -4.994447

H -9.977850 -2.698001 -4.542380

H -9.900343 -1.461789 -3.289816

C -3.416257 -1.652850 -6.264281

H -3.860239 -1.136143 -7.112492

H -2.334680 -1.645290 -6.390011

H -3.746926 -2.689059 -6.273358

S -5.643605 1.782516 -3.773714

S -5.550433 -1.128933 -1.914123

C -6.893799 -0.454833 -0.885030

H -6.635788 -0.736486 0.135099

H -7.840789 -0.914615 -1.162210

S -7.098834 1.374341 -0.955455

C -7.459871 1.726032 0.816724

H -7.397654 2.813497 0.869744

H -6.648352 1.310893 1.415728

C -8.817180 1.207910 1.224532

H -9.010149 1.492143 2.258415

H -9.613792 1.623907 0.610609

H -8.876657 0.121257 1.174599

O -6.319831 4.451153 -1.277547

H -7.073537 4.810176 -1.758609

H -6.072917 5.143613 -0.654576

P1-2LIGAND2222trans+1H2Oax-NiIIt

64

P1-2LIGAND2222trans+1H2Oax-NiIIt SCF Done: -5511.36936257 A.U.

P -2.528432 1.973237 0.433170

O -1.234383 2.875777 0.398373

O -2.128399 0.731889 1.341738

C -3.107790 -0.248828 1.824882

H -3.830509 0.290011 2.441805

H -3.628320 -0.663114 0.953421

C 0.139043 2.311351 0.466013

H 0.207855 1.506087 -0.270361

H 0.253945 1.880748 1.459568

C 1.090015 3.431765 0.198381

H 0.982245 4.224666 0.934925

H 2.109722 3.053907 0.257242

H 0.945717 3.856187 -0.794046

C -2.363298 -1.295990 2.591063

H -1.838062 -0.860440 3.438383

H -3.068279 -2.030978 2.976004

H -1.643628 -1.814125 1.960796

S -4.176185 2.947047 0.860911

S -2.777614 1.136396 -1.553879

C -2.204168 2.580259 -2.534391

H -2.297889 2.259944 -3.572417

H -1.164274 2.800737 -2.303939

S -3.284304 4.027686 -2.261490

C -3.519416 4.606168 -3.996839

H -4.344069 5.316111 -3.919562

H -3.868987 3.756711 -4.585080

C -2.274526 5.241732 -4.563517

H -2.481391 5.606177 -5.569317

H -1.941005 6.086604 -3.964423

H -1.451216 4.531840 -4.640657

Ni -5.018602 2.417957 -1.419067

P -6.036447 -0.104241 -3.820810

O -7.574932 -0.338395 -4.106607

O -5.283714 -0.854533 -5.005883

C -3.838029 -0.762230 -5.219946

H -3.581993 0.299205 -5.286441

H -3.338843 -1.190581 -4.345113

C -8.086745 -1.655582 -4.557572

H -7.654764 -2.423663 -3.910197

H -7.715606 -1.807880 -5.570462

C -9.578487 -1.603462 -4.484808

H -9.976568 -0.812365 -5.116668

H -9.985218 -2.550618 -4.835887

H -9.928176 -1.445154 -3.465822

C -3.513743 -1.508135 -6.476086

H -4.028344 -1.077058 -7.332282

H -2.442286 -1.454550 -6.662256

H -3.788885 -2.557418 -6.394329

S -5.658467 1.844574 -3.704708

S -5.497290 -1.227049 -2.109107

C -6.846000 -0.704482 -0.998376

H -6.671743 -1.249686 -0.071684

H -7.800333 -1.019071 -1.415401

S -6.954405 1.085632 -0.612531

C -7.070569 1.049656 1.223141

H -7.018107 2.104931 1.493939

H -6.167157 0.571904 1.605298

C -8.340080 0.402709 1.716717

H -8.373951 0.453730 2.804418

H -9.224381 0.906593 1.331100

H -8.402707 -0.650144 1.442859

O -6.490925 3.971347 -1.235941

H -7.261158 4.010920 -1.814477

H -6.305622 4.872757 -0.951210

P1-2LIGAND2222trans+1H2Oax-ZnII

64

P1-2LIGAND2222trans+1H2Oax-ZnII SCF Done: -5782.42056301 A.U.

P -2.263115 2.389924 0.153026

O -1.354917 3.071682 1.246180

O -2.092771 0.822537 0.413863

C -2.877667 -0.174753 -0.299587

H -3.915079 0.179513 -0.364601

H -2.488356 -0.242697 -1.319506

C 0.056140 2.664407 1.456139

H 0.544710 2.652707 0.478477

H 0.036692 1.649986 1.854468

C 0.670465 3.649186 2.396927

H 0.149047 3.662353 3.351606

H 1.704526 3.362135 2.582497

H 0.669065 4.654898 1.981817

C -2.778483 -1.465727 0.452686

H -3.151550 -1.357728 1.469763

H -3.376382 -2.227181 -0.047104

H -1.750649 -1.819186 0.500313

S -4.088475 3.161045 0.423735

S -1.493097 2.648730 -1.775817

C -2.120069 4.356077 -2.109507

H -1.368654 4.823498 -2.742683

H -2.180529 4.918585 -1.180590

S -3.730946 4.452504 -2.929425

C -3.274359 3.932536 -4.637325

H -4.231135 3.704793 -5.107694

H -2.729996 2.989395 -4.549768

C -2.504348 4.985994 -5.392759

H -2.289009 4.625550 -6.398273

H -3.068390 5.912089 -5.482060

H -1.546713 5.216403 -4.926734

Zn -5.245827 2.572808 -1.550379

P -5.909672 -0.482974 -3.398646

O -7.199525 -0.159216 -4.257873

O -5.218742 -1.708628 -4.130036

C -3.900892 -2.247844 -3.760694

H -3.205962 -1.406146 -3.702705

H -3.996280 -2.699040 -2.769553

C -8.101860 -1.238162 -4.739558

H -8.305204 -1.905335 -3.897971

H -7.549636 -1.791620 -5.497924

C -9.334130 -0.583706 -5.273907

H -9.099020 0.096452 -6.089679

H -10.004383 -1.350648 -5.659197

H -9.867303 -0.033174 -4.500413

C -3.509839 -3.247035 -4.802189

H -3.434082 -2.784259 -5.783790

H -2.536837 -3.665491 -4.549340

H -4.224207 -4.065852 -4.853917

S -4.718851 1.123877 -3.333966

S -6.538797 -1.255102 -1.542202

C -7.798421 0.026080 -1.160093

H -8.547339 -0.473384 -0.548985

H -8.278715 0.358287 -2.079049

S -7.239395 1.512665 -0.279903

C -6.585385 0.788977 1.284678

H -6.246091 1.661308 1.841193

H -5.701875 0.201040 1.025488

C -7.612491 -0.007011 2.047290

H -7.191702 -0.304715 3.007070

H -8.511992 0.573075 2.246340

H -7.898024 -0.923817 1.532288

O -6.643902 4.120890 -1.909610

H -7.513468 4.216796 -1.505318

H -6.367984 4.996825 -2.204959

P1-2LIGAND2222trans+1H2Oeq-CoIId

64

P1-2LIGAND2222trans+1H2Oeq-CoIId SCF Done: -5385.81250049 A.U.

P -2.311058 1.640549 0.221832

O -1.245709 2.779966 0.475312

O -1.864406 0.445635 1.169440

C -2.646356 -0.785438 1.336526

H -3.653209 -0.494801 1.649353

H -2.711372 -1.277954 0.361517

C 0.203825 2.481256 0.588441

H 0.472758 1.811431 -0.233148

H 0.342279 1.948807 1.528721

C 0.932993 3.784687 0.541778

H 0.624536 4.438622 1.354495

H 2.001020 3.599412 0.646501

H 0.773672 4.303706 -0.402257

C -1.948421 -1.630979 2.354175

H -1.884472 -1.120619 3.312818

H -2.506073 -2.554918 2.499446

H -0.943734 -1.892216 2.028668

S -4.160329 2.321263 0.550957

S -2.048541 0.881954 -1.741097

C -1.933158 2.460130 -2.644067

H -1.969286 2.186377 -3.698167

H -0.987321 2.951435 -2.424976

S -3.306873 3.630606 -2.295847

C -3.568427 4.339924 -3.977901

H -4.503324 4.892933 -3.874544

H -3.744358 3.512723 -4.666740

C -2.425297 5.228271 -4.402102

H -2.657092 5.671355 -5.370106

H -2.256602 6.039308 -3.696373

H -1.493915 4.674833 -4.517970

Co -5.084575 2.308732 -1.590288

P -6.122288 -0.134959 -3.750303

O -7.669601 -0.386052 -3.954413

O -5.416009 -0.967167 -4.904828

C -3.983281 -0.876575 -5.212664

H -3.765316 0.168247 -5.448790

H -3.425238 -1.156175 -4.313772

C -8.201969 -1.739204 -4.252806

H -7.721030 -2.446010 -3.570781

H -7.896918 -1.978618 -5.270807

C -9.685588 -1.683807 -4.083958

H -10.132379 -0.954268 -4.755669

H -10.107163 -2.659974 -4.319488

H -9.966577 -1.434432 -3.061929

C -3.708615 -1.794529 -6.361357

H -4.285852 -1.509591 -7.238192

H -2.652498 -1.741559 -6.620822

H -3.941684 -2.826488 -6.108503

S -5.720672 1.823225 -3.780480

S -5.509180 -1.099133 -1.966768

C -6.818319 -0.499172 -0.852343

H -6.503428 -0.807334 0.144394

H -7.765778 -0.975385 -1.096790

S -7.063032 1.322701 -0.855453

C -7.367905 1.605411 0.939995

H -7.331670 2.691623 1.033663

H -6.520954 1.194880 1.490962

C -8.692148 1.037558 1.386878

H -8.850416 1.282429 2.436624

H -9.524555 1.449301 0.819357

H -8.723982 -0.048206 1.303097

O -6.243918 4.227556 -1.402625

H -7.019453 4.473419 -1.917922

H -6.041632 4.980478 -0.837351

P1-2LIGAND2222trans+1H2Oeq-CoIIq

64

P1-2LIGAND2222trans+1H2Oeq-CoIIq SCF Done: -5385.82992653 A.U.

P -1.960962 1.413467 -0.538850

O -2.961123 2.425588 0.232106

O -1.167107 0.676972 0.618285

C -0.136029 -0.350274 0.341615

H -0.606512 -1.129230 -0.261658

H 0.652961 0.125493 -0.245135

C -2.464303 3.240072 1.370652

H -1.519455 3.698727 1.068430

H -2.262151 2.543578 2.184261

C -3.512951 4.247264 1.716537

H -4.454904 3.765797 1.980371

H -3.185050 4.821133 2.581907

H -3.688146 4.945813 0.899944

C 0.361054 -0.856438 1.656614

H -0.439061 -1.315086 2.233808

H 1.123900 -1.612837 1.479605

H 0.810529 -0.059259 2.244912

S -3.120552 0.270943 -1.663888

S -0.529175 2.599664 -1.518480

C -1.611038 3.969386 -2.098920

H -0.927554 4.628740 -2.632050

H -2.016631 4.513476 -1.247206

S -3.028521 3.583161 -3.167393

C -2.268621 2.532073 -4.469885

H -3.119412 2.276236 -5.102808

H -1.926820 1.617052 -3.982359

C -1.175039 3.232668 -5.233899

H -0.835529 2.591115 -6.046364

H -1.516009 4.169623 -5.670571

H -0.304338 3.436181 -4.611128

Co -4.650849 2.202496 -1.783382

P -6.973036 0.163609 -3.677682

O -8.424011 0.510781 -4.192304

O -6.446512 -0.967364 -4.661496

C -5.067586 -1.473660 -4.637495

H -4.401484 -0.615624 -4.764959

H -4.887795 -1.912314 -3.652113

C -9.392787 -0.552729 -4.564030

H -9.416216 -1.279073 -3.747819

H -8.998218 -1.038315 -5.456176

C -10.713600 0.106788 -4.792200

H -10.653882 0.846426 -5.587631

H -11.438831 -0.649204 -5.090055

H -11.083006 0.589801 -3.889720

C -4.932660 -2.476189 -5.739511

H -5.130086 -2.025628 -6.709823

H -3.916302 -2.866817 -5.747409

H -5.612864 -3.313226 -5.597327

S -5.919830 1.874335 -3.717777

S -7.050616 -0.821838 -1.828503

C -7.550065 0.594470 -0.771106

H -8.218433 0.188113 -0.014953

H -8.106375 1.329199 -1.351075

S -6.187310 1.473164 0.050780

C -5.437464 0.121562 1.047510

H -4.550218 0.591569 1.475383

H -5.090661 -0.639509 0.345868

C -6.363197 -0.426427 2.103127

H -5.828502 -1.159172 2.706563

H -6.731750 0.350488 2.770580

H -7.220638 -0.942958 1.672240

O -5.306016 4.160618 -1.203503

H -6.008293 4.302399 -0.556834

H -5.384239 4.868311 -1.855069

P1-2LIGAND2222trans+1H2Oeq-CoIIqISOMER

64

P1-2LIGAND2222trans+1H2Oeq-CoIIqCCCCCC SCF Done: -5385.82759617 A.U.

P -2.196356 2.349258 0.089507

O -1.324207 3.035231 1.210116

O -1.924437 0.785173 0.266762

C -2.726026 -0.222105 -0.417057

H -3.765356 0.129104 -0.448263

H -2.366723 -0.288159 -1.447979

C 0.105965 2.693956 1.408685

H 0.597972 2.758488 0.434906

H 0.140133 1.660988 1.754602

C 0.661972 3.660889 2.402768

H 0.135311 3.597931 3.352563

H 1.708718 3.419786 2.582353

H 0.609420 4.684900 2.039130

C -2.586996 -1.508740 0.336861

H -2.947764 -1.406420 1.358916

H -3.172219 -2.287882 -0.150632

H -1.550707 -1.838534 0.370040

S -4.083604 2.923445 0.414777

S -1.452432 2.762596 -1.824089

C -2.205314 4.428110 -2.050207

H -1.499225 5.001907 -2.646849

H -2.321114 4.925658 -1.089567

S -3.821233 4.437549 -2.868280

C -3.362783 3.920207 -4.575497

H -4.327553 3.781546 -5.063975

H -2.898201 2.934609 -4.501911

C -2.499148 4.924666 -5.294210

H -2.331320 4.587373 -6.316538

H -2.967236 5.906169 -5.339048

H -1.517135 5.034019 -4.835234

Co -5.277175 2.660338 -1.607578

P -5.949304 -0.477585 -3.403464

O -7.197581 -0.159863 -4.323533

O -5.216941 -1.701402 -4.098175

C -3.895544 -2.204353 -3.694778

H -3.185971 -1.381122 -3.807686

H -3.949467 -2.481001 -2.638286

C -8.082751 -1.242879 -4.827435

H -8.293535 -1.918328 -3.994398

H -7.512653 -1.784719 -5.581126

C -9.312215 -0.595616 -5.376672

H -9.070069 0.095029 -6.181598

H -9.967787 -1.365328 -5.781290

H -9.864000 -0.058498 -4.606793

C -3.568425 -3.371854 -4.570204

H -3.535686 -3.080778 -5.617745

H -2.588916 -3.761563 -4.297455

H -4.294934 -4.172525 -4.450488

S -4.788708 1.163278 -3.309173

S -6.642567 -1.240846 -1.574248

C -7.845886 0.103062 -1.206157

H -8.626746 -0.364141 -0.609563

H -8.296337 0.457054 -2.132086

S -7.214655 1.550764 -0.320305

C -6.628484 0.781532 1.247739

H -6.258178 1.630523 1.820625

H -5.767118 0.158179 0.996518

C -7.703939 0.026757 1.985371

H -7.317687 -0.290292 2.953409

H -8.583350 0.642819 2.165626

H -8.015409 -0.876781 1.462118

O -6.629970 4.220285 -1.730169

H -7.504541 4.249283 -1.324021

H -6.407751 5.114033 -2.019083

P1-2LIGAND2222trans+1H2Oeq-CoIIs

64

P1-2LIGAND2222trans+1H2Oeq-CoIIq SCF Done: -5385.74301133 A.U.

P -1.803738 2.131422 0.253846

O -0.877599 3.414285 0.406890

O -1.296170 1.189115 1.461425

C -1.972094 -0.041510 1.838920

H -2.868745 0.245401 2.396768

H -2.285533 -0.562235 0.926309

C -0.411943 3.956206 1.707238

H 0.221192 3.196569 2.163819

H -1.296122 4.099530 2.332751

C 0.314534 5.231050 1.428684

H -0.334460 5.969688 0.962654

H 0.674801 5.645355 2.369787

H 1.176493 5.063798 0.786205

C -1.029697 -0.864131 2.662731

H -0.715621 -0.323070 3.552767

H -1.527850 -1.777865 2.982979

H -0.145374 -1.141589 2.093340

S -3.805612 2.733378 0.781621

S -3.142518 0.419876 -1.777110

C -2.485276 1.458228 -3.117740

H -3.023017 1.241460 -4.042264

H -1.417538 1.296882 -3.250330

S -2.848151 3.188878 -2.644006

C -3.254291 3.990828 -4.249983

H -3.650492 4.963396 -3.956443

H -4.062848 3.418713 -4.705863

C -2.041856 4.108109 -5.141246

H -2.324386 4.625924 -6.057582

H -1.242612 4.676316 -4.669905

H -1.648360 3.133732 -5.428923

Co -4.742991 2.200859 -1.235092

P -6.804286 0.054648 -3.865170

O -8.216583 0.367611 -4.503626

O -6.112356 -0.929358 -4.906827

C -4.749202 -1.439728 -4.745797

H -4.089238 -0.574339 -4.638986

H -4.707650 -2.016516 -3.817912

C -9.045508 -0.703452 -5.110501

H -9.088559 -1.533782 -4.400999

H -8.523794 -1.035314 -6.007563

C -10.389849 -0.118922 -5.399509

H -10.315133 0.724649 -6.082381

H -11.011692 -0.878376 -5.871366

H -10.890676 0.207929 -4.490296

C -4.423780 -2.265724 -5.949994

H -4.477529 -1.674730 -6.861804

H -3.412219 -2.657663 -5.857084

H -5.102738 -3.110828 -6.041738

S -5.925476 1.806524 -3.520686

S -7.040782 -1.157808 -2.163101

C -7.735116 0.113835 -1.049413

H -8.438700 -0.399004 -0.395583

H -8.284666 0.868265 -1.610053

S -6.545437 0.997983 0.008544

C -5.707808 -0.404549 0.853973

H -5.018502 0.103115 1.529293

H -5.118301 -0.934315 0.101008

C -6.658051 -1.316203 1.588544

H -6.084966 -2.045022 2.160652

H -7.290863 -0.770608 2.286671

H -7.296093 -1.883639 0.911780

O -5.914031 3.998199 -1.446624

H -6.716617 4.072674 -1.976165

H -5.877798 4.761028 -0.858980

P1-2LIGAND2222trans+1H2Oeq-CuIId

64

P1-2LIGAND2222trans+1H2Oeq-CuIId SCF Done: -5643.53526818 A.U.

P -2.309270 2.119382 0.194444

O -1.283277 2.861322 1.132385

O -2.292969 0.611579 0.714409

C -3.167756 -0.427457 0.183793

H -3.763672 -0.764035 1.032550

H -3.853190 0.014095 -0.553829

C 0.051496 2.288974 1.445717

H 0.524322 2.023595 0.496598

H -0.121318 1.380469 2.022169

C 0.815324 3.326127 2.202042

H 0.308071 3.595356 3.125931

H 1.793885 2.923731 2.460741

H 0.966760 4.225250 1.608428

C -2.338021 -1.534206 -0.393363

H -1.651593 -1.933750 0.350271

H -2.983800 -2.351043 -0.716028

H -1.754082 -1.196851 -1.250153

S -4.007368 3.160553 0.300173

S -1.565409 1.949273 -1.767870

C -2.043547 3.620456 -2.354595

H -1.321556 3.889063 -3.124428

H -1.966603 4.341825 -1.544547

S -3.706884 3.756063 -3.073358

C -3.401307 2.856790 -4.651382

H -4.388722 2.537012 -4.984181

H -2.832176 1.962565 -4.386446

C -2.696944 3.712841 -5.675537

H -2.541604 3.128618 -6.582427

H -3.282675 4.590358 -5.939941

H -1.716207 4.047609 -5.338921

Cu -5.323707 2.588570 -1.567715

P -6.624637 0.023442 -3.506383

O -7.809440 -0.394996 -4.455149

O -5.341200 -0.697322 -4.131501

C -4.027238 -0.573857 -3.518380

H -3.924304 0.443195 -3.110788

H -3.985311 -1.274314 -2.680625

C -8.015892 -1.799411 -4.892677

H -7.971751 -2.432237 -4.002759

H -7.181554 -2.050129 -5.547369

C -9.339884 -1.861757 -5.581217

H -9.368033 -1.201576 -6.445307

H -9.507819 -2.879603 -5.930683

H -10.154324 -1.597573 -4.909938

C -2.993261 -0.876652 -4.558874

H -3.078091 -0.203324 -5.410487

H -1.998215 -0.766378 -4.128497

H -3.089842 -1.897187 -4.923851

S -6.641583 2.015916 -3.437784

S -6.791607 -0.869496 -1.605879

C -7.935299 0.356663 -0.863176

H -8.503372 -0.179886 -0.104837

H -8.629972 0.731274 -1.610866

S -7.148120 1.789793 -0.063830

C -6.488496 0.958219 1.439450

H -5.740114 1.649270 1.824885

H -5.965725 0.063067 1.093374

C -7.563864 0.654817 2.454036

H -7.109130 0.183624 3.325140

H -8.068667 1.558159 2.789886

H -8.317068 -0.035895 2.076606

O -6.393581 4.648298 -1.458081

H -7.335178 4.806522 -1.334826

H -5.985503 5.520382 -1.453987

P1-2LIGAND2222trans+1H2Oeq-FeIIId

64

P1-2LIGAND2222trans+1H2Oeq-FeIIId SCF Done: -5266.27689982 A.U.

P -2.045071 1.377098 0.107659

O -1.716159 2.810422 0.670046

O -1.308052 0.307368 0.974915

C -0.681403 -0.987181 0.547491

H -1.118050 -1.715572 1.226413

H -1.017657 -1.203819 -0.468759

C -0.299394 3.209138 1.007145

H 0.332701 2.895525 0.172561

H -0.033292 2.633008 1.892159

C -0.291580 4.682319 1.233334

H -0.948848 4.963003 2.053429

H 0.719654 4.986286 1.502512

H -0.575673 5.235374 0.339266

C 0.800527 -0.865045 0.670202

H 1.093745 -0.604459 1.685102

H 1.251338 -1.829316 0.435392

H 1.209646 -0.131503 -0.023482

S -4.090674 1.102782 0.302462

S -1.459807 1.175730 -1.890572

C -1.790733 2.878163 -2.464866

H -1.807767 2.800318 -3.551782

H -0.976351 3.533628 -2.161994

S -3.349956 3.696761 -1.915705

C -3.904029 4.502315 -3.496441

H -4.890458 4.894774 -3.240985

H -4.035074 3.705131 -4.230162

C -2.954860 5.583603 -3.943620

H -3.361743 6.049274 -4.841332

H -2.834571 6.362886 -3.193856

H -1.971946 5.194183 -4.205975

Fe -5.138115 2.268869 -1.238500

P -6.133508 -0.427561 -3.570397

O -7.039800 0.147591 -4.720092

O -5.218787 -1.578982 -4.103473

C -5.106202 -2.997279 -3.635518

H -4.086479 -3.252870 -3.911216

H -5.183333 -2.989284 -2.546719

C -7.781517 -0.746849 -5.685775

H -8.476148 -1.337760 -5.085845

H -7.031588 -1.402079 -6.128752

C -8.460796 0.132665 -6.678260

H -7.746665 0.729901 -7.240546

H -8.994287 -0.501032 -7.386683

H -9.189570 0.789013 -6.207920

C -6.130164 -3.853959 -4.303116

H -6.035674 -3.818333 -5.386581

H -5.973281 -4.888350 -3.996879

H -7.147658 -3.584307 -4.018291

S -4.943603 1.243495 -3.136652

S -7.239891 -1.158700 -1.965957

C -8.102992 0.424772 -1.628743

H -9.070855 0.164717 -1.202627

H -8.279000 0.979847 -2.549491

S -7.264574 1.537918 -0.463956

C -7.199298 0.499232 1.071964

H -6.697121 1.152280 1.786639

H -6.539945 -0.344552 0.860832

C -8.567435 0.077579 1.541860

H -8.460044 -0.409778 2.510809

H -9.242023 0.921549 1.673125

H -9.034484 -0.649361 0.878881

O -5.691864 3.984780 -0.271610

H -6.554549 4.147106 0.135527

H -5.065088 4.612709 0.113958

P1-2LIGAND2222trans+1H2Oeq-FeIIIq

64

P1-2LIGAND2222trans+1H2Oeq-FeIIIq SCF Done: -5266.31310522 A.U.

P -2.278472 2.371869 0.693484

O -1.548179 3.696348 0.298840

O -1.615625 1.855176 2.012728

C -2.061820 0.720849 2.882317

H -3.138151 0.838118 3.022122

H -1.856888 -0.191950 2.318892

C -0.114271 4.031656 0.671397

H 0.500238 3.193117 0.338707

H -0.097186 4.081769 1.758078

C 0.214377 5.321000 0.003780

H -0.438582 6.123800 0.338397

H 1.236404 5.592002 0.268631

H 0.163148 5.245308 -1.081301

C -1.289506 0.803233 4.155355

H -1.493469 1.728989 4.688596

H -1.590695 -0.024600 4.796542

H -0.219681 0.715684 3.980628

S -4.292052 2.577776 0.722442

S -1.962210 0.916178 -0.879508

C -1.714664 2.069599 -2.281337

H -1.631459 1.435928 -3.164846

H -0.791888 2.631015 -2.145015

S -3.138014 3.207192 -2.454564

C -3.330148 3.301085 -4.289634

H -4.260432 3.859484 -4.407348

H -3.487949 2.286730 -4.658094

C -2.158943 4.002622 -4.935379

H -2.354989 4.088363 -6.004146

H -2.015959 5.008186 -4.545279

H -1.228930 3.446429 -4.824965

Fe -4.629291 1.500587 -1.322522

P -6.481087 -0.386364 -3.730284

O -7.331815 0.951070 -3.895260

O -6.512596 -1.120820 -5.110436

C -5.937051 -2.481292 -5.414638

H -4.858738 -2.386383 -5.286621

H -6.336889 -3.166713 -4.665388

C -8.699369 0.914182 -4.547628

H -9.206459 0.019331 -4.181898

H -8.509241 0.813471 -5.614256

C -9.399518 2.178178 -4.179538

H -8.858076 3.056292 -4.529761

H -10.376063 2.191793 -4.662959

H -9.573427 2.253970 -3.105730

C -6.341864 -2.833388 -6.804539

H -5.959110 -2.117155 -7.527718

H -5.919521 -3.808765 -7.045582

H -7.422305 -2.906128 -6.904277

S -4.587566 0.156277 -3.188797

S -7.406962 -1.659278 -2.354835

C -7.559951 -0.493244 -0.965150

H -8.241065 -0.978665 -0.265132

H -8.017210 0.444737 -1.277311

S -6.070800 -0.034066 0.008687

C -5.166819 -1.636192 0.146569

H -4.250290 -1.358856 0.671031

H -4.902668 -1.949114 -0.865698

C -5.968426 -2.670627 0.897441

H -5.350950 -3.558792 1.030897

H -6.272114 -2.324990 1.883727

H -6.855155 -2.983539 0.347720

O -6.363681 2.636009 -1.879538

H -6.845925 2.436787 -2.700900

H -6.673877 3.483911 -1.538642

P1-2LIGAND2222trans+1H2Oeq-FeIIIs

64

P1-2LIGAND2222trans+1H2Oeq-FeIIIs SCF Done: -5266.32727502 A.U.

P -1.878121 1.496884 -0.585709

O -2.904158 2.452320 0.209627

O -1.161945 0.610860 0.485325

C 0.076771 -0.228845 0.257101

H -0.206106 -0.987543 -0.472816

H 0.827299 0.437147 -0.172074

C -2.425862 3.155556 1.460807

H -1.461487 3.609535 1.223190

H -2.270737 2.368787 2.197771

C -3.456820 4.151875 1.868133

H -4.416126 3.678836 2.078736

H -3.128114 4.630397 2.790526

H -3.588440 4.935449 1.123862

C 0.482578 -0.790823 1.574731

H -0.291053 -1.427776 1.997578

H 1.370704 -1.404040 1.422281

H 0.739647 -0.009171 2.286099

S -3.104596 0.484486 -1.850364

S -0.454779 2.670414 -1.554294

C -1.515695 4.120001 -1.955392

H -0.840224 4.796918 -2.479398

H -1.854206 4.613145 -1.046191

S -2.991564 3.885562 -2.993729

C -2.329122 3.011556 -4.483487

H -3.215592 2.908567 -5.110757

H -2.022343 2.014835 -4.159666

C -1.227155 3.782279 -5.162453

H -0.966930 3.268726 -6.087920

H -1.528938 4.794842 -5.423643

H -0.318085 3.826090 -4.563905

Fe -4.620086 2.281069 -1.780801

P -6.733237 0.207441 -3.784129

O -8.159128 0.492219 -4.368835

O -5.832047 -0.617013 -4.766531

C -5.297378 -2.005919 -4.615323

H -4.517594 -2.033274 -5.371805

H -4.833950 -2.064523 -3.628031

C -9.041321 -0.572459 -4.971610

H -9.179985 -1.334397 -4.201915

H -8.478673 -0.990830 -5.806362

C -10.311960 0.085372 -5.387292

H -10.137393 0.861167 -6.128952

H -10.953902 -0.670153 -5.840213

H -10.845560 0.509701 -4.539823

C -6.364707 -3.027429 -4.834278

H -6.820255 -2.925884 -5.817242

H -5.916001 -4.019629 -4.785342

H -7.141036 -2.989282 -4.068280

S -6.042974 2.136239 -3.578553

S -6.787225 -0.847995 -1.972845

C -7.450513 0.475771 -0.889664

H -8.095493 -0.017977 -0.164731

H -8.054249 1.186591 -1.451927

S -6.196065 1.430481 0.036808

C -5.352093 0.089741 0.994288

H -4.524761 0.614353 1.474514

H -4.930516 -0.599530 0.259245

C -6.264295 -0.581736 1.987553

H -5.680895 -1.293247 2.571799

H -6.714250 0.124569 2.682191

H -7.058378 -1.151636 1.506928

O -5.262039 4.199206 -1.032305

H -5.912883 4.308462 -0.325967

H -5.309836 4.998841 -1.573986

P1-2LIGAND2222trans+1H2Oeq-FeIIIo

64

P1-2LIGAND2222trans+1H2Oeq-FeIIIo SCF Done: -5266.23418242 A.U.

P -1.593774 1.525979 -0.249980

O -2.120605 2.653165 0.786260

O -0.678785 0.601423 0.660493

C 0.102905 -0.578398 0.187207

H -0.598139 -1.237383 -0.329184

H 0.841338 -0.200743 -0.521613

C -1.297039 3.113415 1.961076

H -0.327144 3.424241 1.573082

H -1.166365 2.240351 2.597802

C -2.048430 4.224571 2.614529

H -3.023867 3.896785 2.973273

H -1.485891 4.564285 3.483718

H -2.162438 5.084289 1.954224

C 0.727063 -1.211914 1.386736

H -0.022743 -1.564908 2.090979

H 1.310685 -2.071474 1.058806

H 1.402928 -0.526815 1.893506

S -3.334041 0.442489 -0.822648

S -0.767500 1.238986 -3.251179

C -1.981889 2.323805 -3.962517

H -2.743793 1.717053 -4.465990

H -1.481613 2.926867 -4.727621

S -2.784425 3.491554 -2.780757

C -3.525257 4.717897 -3.970551

H -4.211568 5.287696 -3.342384

H -4.116874 4.141763 -4.684472

C -2.487428 5.592564 -4.622381

H -2.999229 6.310387 -5.263899

H -1.905155 6.153877 -3.895138

H -1.805286 5.034121 -5.262266

Fe -4.665283 2.097826 -1.668296

P -6.342268 -0.191332 -3.843243

O -7.539089 0.035128 -4.834225

O -5.310494 -1.256230 -4.363257

C -5.051023 -2.629145 -3.833228

H -4.085365 -2.865724 -4.272562

H -4.930045 -2.539988 -2.751516

C -8.348604 -1.075729 -5.443973

H -8.788759 -1.633507 -4.614903

H -7.641704 -1.710953 -5.978214

C -9.365081 -0.447885 -6.335730

H -8.897945 0.121585 -7.135763

H -9.957315 -1.240750 -6.792002

H -10.043630 0.198270 -5.783400

C -6.124438 -3.588092 -4.230282

H -6.226499 -3.651131 -5.311776

H -5.857158 -4.581756 -3.870520

H -7.089187 -3.335925 -3.787330

S -5.508652 1.681471 -3.801052

S -6.976587 -0.838064 -1.953749

C -7.891330 0.686570 -1.484928

H -8.731347 0.364482 -0.871341

H -8.288762 1.192725 -2.363615

S -6.927400 1.902261 -0.535765

C -6.589493 0.962432 1.026712

H -5.850142 1.583433 1.534935

H -6.097166 0.031353 0.736489

C -7.830666 0.748138 1.853299

H -7.549428 0.254451 2.783517

H -8.322408 1.684155 2.109986

H -8.554230 0.099008 1.362190

O -4.488198 3.666646 -0.239499

H -5.051192 4.405367 0.021500

H -3.676628 3.670165 0.302984

P1-2LIGAND2222trans+1H2Oeq-MnIId

64

P1-2LIGAND2222trans+1H2Oeq-MnIId SCF Done: -5153.99956977 A.U.

P -2.134465 1.203093 -0.380799

O -1.437294 2.499184 0.192956

O -1.342108 -0.021277 0.245267

C -1.718408 -1.426217 0.034985

H -2.794824 -1.513849 0.206579

H -1.505248 -1.666310 -1.010235

C 0.030651 2.691771 0.109345

H 0.381118 2.237870 -0.821624

H 0.459615 2.140316 0.945159

C 0.294192 4.161659 0.174408

H -0.078572 4.589794 1.102327

H 1.368084 4.336826 0.130263

H -0.169760 4.685503 -0.660699

C -0.914062 -2.259615 0.981375

H -1.126555 -1.997218 2.015381

H -1.165483 -3.309584 0.839580

H 0.152438 -2.142238 0.801867

S -4.099929 1.182986 0.083611

S -1.793232 1.057691 -2.453507

C -2.133149 2.761542 -3.010947

H -2.476777 2.656773 -4.040162

H -1.204108 3.327696 -2.987468

S -3.385904 3.738108 -2.067873

C -3.764477 4.998861 -3.367864

H -4.475461 5.657283 -2.866777

H -4.270397 4.491643 -4.193173

C -2.551185 5.772100 -3.823425

H -2.867189 6.583974 -4.477373

H -2.017006 6.213512 -2.983268

H -1.857310 5.157969 -4.394588

Mn -5.264766 2.569767 -1.329906

P -6.402457 -0.334900 -3.276345

O -7.491619 -0.313840 -4.422363

O -5.473367 -1.575049 -3.633700

C -4.248076 -1.871100 -2.884609

H -3.962475 -0.976729 -2.319009

H -4.502422 -2.663551 -2.178094

C -8.167532 -1.558894 -4.870049

H -8.502684 -2.093054 -3.977409

H -7.413470 -2.160239 -5.377002

C -9.296907 -1.159463 -5.762681

H -8.940550 -0.601387 -6.625834

H -9.795417 -2.057162 -6.125827

H -10.033302 -0.557348 -5.233851

C -3.194757 -2.281301 -3.866064

H -2.957188 -1.467924 -4.550195

H -2.283516 -2.559337 -3.337185

H -3.513801 -3.142292 -4.449684

S -5.453584 1.454339 -3.315160

S -7.256249 -0.875423 -1.441079

C -8.190301 0.672514 -1.152072

H -9.091206 0.400204 -0.606123

H -8.492756 1.117663 -2.098579

S -7.300405 1.956651 -0.217151

C -7.109309 1.135126 1.423333

H -6.496273 1.838787 1.986652

H -6.511916 0.235499 1.262109

C -8.423444 0.852156 2.104025

H -8.229061 0.458608 3.101117

H -9.029492 1.749521 2.214114

H -9.011632 0.099386 1.580223

O -6.445164 4.197218 -1.891290

H -7.360442 4.230704 -1.579281

H -6.439531 4.586186 -2.773799

P1-2LIGAND2222trans+1H2Oeq-MnIIs

64

P1-2LIGAND2222trans+1H2Oeq-MnIIs SCF Done: -5154.10179711 A.U.

P -2.071688 1.408591 -0.228737

O -1.438726 2.468789 0.761888

O -1.310824 0.049275 0.080341

C -1.697029 -1.246295 -0.490016

H -2.776422 -1.359610 -0.354830

H -1.474696 -1.213622 -1.560873

C 0.030177 2.570291 0.955337

H 0.498743 2.564211 -0.032427

H 0.338208 1.674433 1.493199

C 0.294069 3.829966 1.714785

H -0.209018 3.823464 2.679328

H 1.364451 3.918668 1.894727

H -0.025055 4.710346 1.158898

C -0.914166 -2.306770 0.217512

H -1.138774 -2.316601 1.281809

H -1.174606 -3.281336 -0.192635

H 0.155992 -2.162437 0.086199

S -4.035857 1.300952 0.089691

S -1.470833 1.903551 -2.187713

C -1.925338 3.684141 -2.135762

H -1.183620 4.197089 -2.744232

H -1.839486 4.056974 -1.115798

S -3.576854 4.138811 -2.738263

C -3.480521 3.610527 -4.503467

H -4.468732 3.855532 -4.893490

H -3.391233 2.522400 -4.504804

C -2.382140 4.292792 -5.276039

H -2.456677 4.010736 -6.325704

H -2.454121 5.377353 -5.218506

H -1.389334 3.992956 -4.941444

Mn -5.379757 2.581107 -1.528190

P -6.711622 -0.179634 -3.403676

O -7.915273 -0.519693 -4.365731

O -5.571687 -1.220309 -3.799250

C -4.175943 -1.066970 -3.397335

H -4.103661 -0.299653 -2.615443

H -3.904916 -2.024649 -2.952800

C -8.367945 -1.915729 -4.585071

H -8.562692 -2.355606 -3.603730

H -7.540870 -2.449667 -5.052730

C -9.587499 -1.859160 -5.446562

H -9.372495 -1.394159 -6.406205

H -9.933867 -2.874469 -5.634717

H -10.394423 -1.311798 -4.964125

C -3.346376 -0.732630 -4.600498

H -3.641777 0.226201 -5.026486

H -2.293899 -0.675430 -4.323746

H -3.448463 -1.494078 -5.370782

S -6.303430 1.754019 -3.655580

S -7.159880 -0.733881 -1.428353

C -8.322093 0.630277 -1.010874

H -9.101420 0.190987 -0.391539

H -8.787210 1.022917 -1.913317

S -7.590789 2.034288 -0.119579

C -7.173944 1.236499 1.490297

H -6.534009 1.966567 1.986246

H -6.555812 0.364722 1.264239

C -8.390398 0.893623 2.311895

H -8.072126 0.470938 3.264328

H -8.999098 1.770568 2.521916

H -9.021172 0.147000 1.830491

O -6.181353 4.604754 -1.153700

H -5.777966 5.447407 -1.392634

H -7.053900 4.805647 -0.795185

P1-2LIGAND2222trans+1H2Oeq-NiII

64

P1-2LIGAND2222trans+1H2Oeq-NiII SCF Done: -5511.34719198 A.U.

P -2.353130 1.543385 0.224773

O -1.348187 2.713064 0.569169

O -1.914420 0.334919 1.156893

C -2.645603 -0.937210 1.230748

H -3.686328 -0.703443 1.470458

H -2.611457 -1.401532 0.240930

C 0.108800 2.474494 0.720242

H 0.430638 1.831391 -0.103671

H 0.241893 1.932424 1.655832

C 0.781842 3.808505 0.716269

H 0.423363 4.434150 1.530800

H 1.853646 3.667526 0.847395

H 0.625542 4.336685 -0.223294

C -1.987024 -1.778573 2.277233

H -2.021659 -1.295234 3.251280

H -2.508133 -2.731631 2.353534

H -0.948980 -1.982896 2.024231

S -4.236773 2.168890 0.510639

S -1.975522 0.863603 -1.740311

C -1.927501 2.470020 -2.597886

H -1.966841 2.226939 -3.659188

H -0.996211 2.986294 -2.372256

S -3.326602 3.600249 -2.202594

C -3.583002 4.392090 -3.846745

H -4.521813 4.932286 -3.717067

H -3.746043 3.601639 -4.580167

C -2.441466 5.308227 -4.213562

H -2.672766 5.803526 -5.156093

H -2.280290 6.080164 -3.463709

H -1.506850 4.767181 -4.357502

Ni -5.066466 2.238352 -1.619037

P -6.008323 -0.168549 -3.775950

O -7.552753 -0.383060 -4.036659

O -5.279061 -1.009807 -4.908136

C -3.831878 -0.962648 -5.156171

H -3.573749 0.075519 -5.379478

H -3.321769 -1.261734 -4.235754

C -8.107680 -1.723342 -4.351402

H -7.668115 -2.439718 -3.651694

H -7.773458 -1.972390 -5.357973

C -9.594390 -1.629808 -4.235115

H -9.997924 -0.889549 -4.922288

H -10.032440 -2.594991 -4.485409

H -9.904812 -1.372109 -3.223627

C -3.538145 -1.885178 -6.296278

H -4.071777 -1.581704 -7.194394

H -2.471415 -1.860766 -6.513276

H -3.809512 -2.910971 -6.056858

S -5.581462 1.790851 -3.797710

S -5.487079 -1.134489 -1.969463

C -6.821447 -0.484367 -0.912117

H -6.535896 -0.754212 0.104000

H -7.764131 -0.965420 -1.166843

S -7.066920 1.338342 -0.991930

C -7.460846 1.687654 0.773781

H -7.430610 2.776723 0.821754

H -6.645534 1.298256 1.384908

C -8.807859 1.131969 1.166015

H -9.024354 1.417509 2.194835

H -9.607702 1.519528 0.537748

H -8.833750 0.043682 1.123637

O -6.318979 4.428696 -1.285859

H -7.065600 4.789461 -1.776568

H -6.085164 5.116321 -0.652518

P1-2LIGAND2222trans+1H2Oeq-NiIIt

64

P1-2LIGAND2222trans+1H2Oeq-NiIIt SCF Done: -5511.36934726 A.U.

P -2.528234 1.978883 0.424076

O -1.228880 2.873877 0.394311

O -2.138285 0.738142 1.338210

C -3.124109 -0.234602 1.823596

H -3.849170 0.312025 2.430899

H -3.640044 -0.654822 0.952278

C 0.140242 2.302653 0.497958

H 0.236327 1.527428 -0.266898

H 0.213026 1.831978 1.477270

C 1.103765 3.428889 0.313494

H 0.970022 4.191960 1.076763

H 2.118941 3.043490 0.397225

H 1.000303 3.893226 -0.665787

C -2.388440 -1.277546 2.603924

H -1.867889 -0.836577 3.451347

H -3.098455 -2.007055 2.990005

H -1.665961 -1.803274 1.983217

S -4.174028 2.960573 0.842290

S -2.775791 1.135433 -1.560311

C -2.199633 2.573670 -2.547130

H -2.293593 2.248490 -3.583634

H -1.159314 2.793428 -2.317977

S -3.277584 4.024175 -2.281999

C -3.509081 4.595902 -4.020061

H -4.332829 5.307343 -3.946931

H -3.858994 3.744583 -4.605374

C -2.262441 5.227397 -4.587453

H -2.467248 5.587725 -5.595153

H -1.928843 6.074434 -3.991496

H -1.439929 4.516133 -4.660093

Ni -5.014788 2.420477 -1.436078

P -6.034050 -0.109095 -3.830220

O -7.572966 -0.341470 -4.115454

O -5.282529 -0.865045 -5.012391

C -3.837153 -0.773857 -5.229215

H -3.581405 0.287172 -5.302937

H -3.336550 -1.196595 -4.352463

C -8.087196 -1.659611 -4.560698

H -7.657144 -2.425587 -3.909556

H -7.715763 -1.817385 -5.572638

C -9.578876 -1.604178 -4.489117
[truncated: 114,518 more chars]
