# Supplementary figures and images for: Pesticides Curbing Soil Fertility: Effect of Complexation of Free Metal Ions
Source: Front Chem. 2017 Jul 4;5:43. doi: 10.3389/fchem.2017.00043 (PMC5495828; doi:10.3389/fchem.2017.00043)

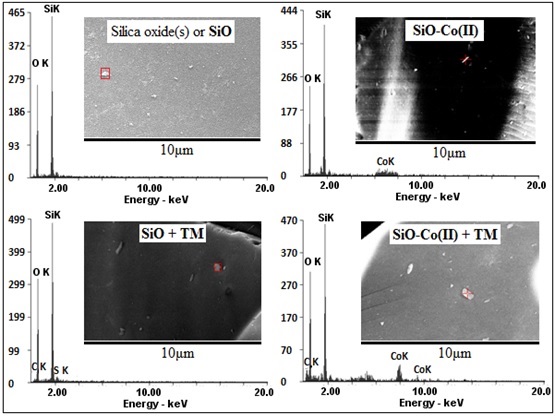

Supplement: Supplementary file 6 [file Image1.jpg]

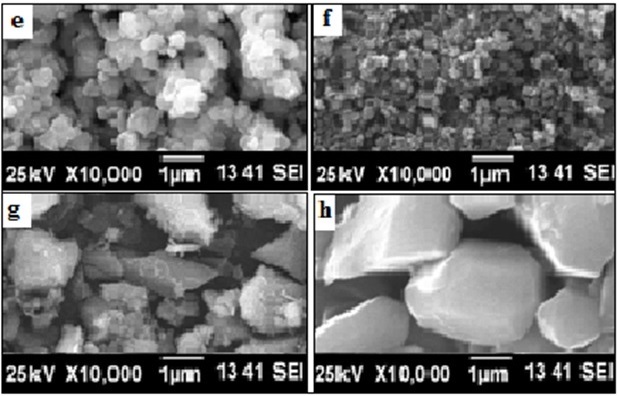

Supplement: Supplementary file 7 [file Image2.jpg]

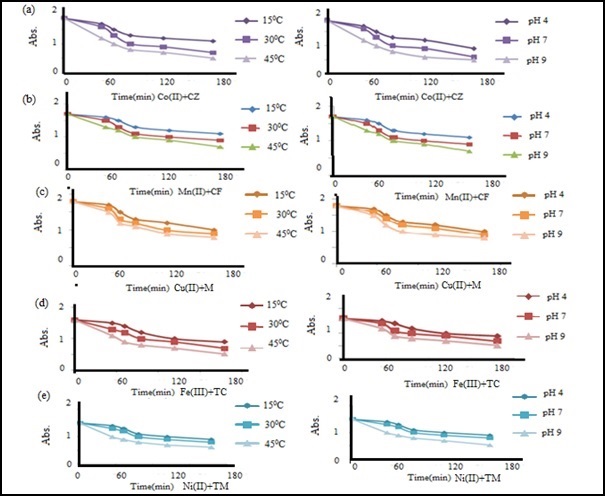

Supplement: Supplementary file 8 [file Image3.jpg]
